# The protein-protein interaction between connective tissue growth factor and annexin A2 is relevant to pannus formation in rheumatoid arthritis

**DOI:** 10.1186/s13075-021-02656-y

**Published:** 2021-10-26

**Authors:** Guoyu Yin, Chenglin Yang, Gan Wu, Xinxin Yu, Qingqing Tian, Daoxing Chen, Ben Cao, Lin Zhao, Nannan Xu, Shengwei Jin, Wei Zhang, Jianguang Wang

**Affiliations:** 1 Department of Anesthesia and Critical Care, School of the Second Clinical Medical Sciences, Wenzhou Medical University, Wenzhou, 325035 China; 2 Department of Biochemistry, School of Basic Medical Sciences, Wenzhou Medical University, Wenzhou, 325035 Zhejiang Province China; 3 Qiushi Academy for Advanced Studies, Zhejiang University, Hangzhou, China; 4 Department of Medicinal Chemistry, School of Pharmaceutical Sciences, Wenzhou Medical University, Wenzhou, China

**Keywords:** Rheumatoid arthritis (RA), Pannus formation, Annexin A2 (ANXA2), Connective tissue growth factor (CTGF)

## Abstract

**Background:**

Connective tissue growth factor (CTGF)-induced angiogenesis is a crucial factor in rheumatoid arthritis (RA), but CTGF-interacting protein and related molecular mechanism of their interaction have not been fully elucidated.

**Methods:**

CTGF-interacting proteins were identified through the LC-MS/MS analysis of the Co-IP products from fibroblast-like synoviocyte (FLS) lysates, and the interaction between CTGF and annexin A2 (ANXA2) was further confirmed through Co-IP and BiFC assay. The binding domain, mutant, mechanism, and angiogenesis function were assessed by homology modeling, molecular docking, MTT, cell scratch, tube formation, and chick chorioallantoic membrane (CAM) assays. Additionally, severe combined immunodeficiency (SCID) mouse co-implantation model was constructed to confirm the effect of ANXA2/CTGF-TSP1 in the process of RA in vivo.

**Results:**

ANXA2 was identified and verified as an interaction partner of CTGF for the first time by Co-IP and LC-MS/MS analysis. Co-localization of CTGF and ANXA2 was observed in RA-FLS, and direct interaction of the TSP-1 domain of CTGF and ANXA2 was determined in HEK293T cells. The spatial conformation and stable combination of the ANXA2/CTGF-TSP1 complex were assessed by homology modeling in the biomimetic environment. The function of the ANXA2/CTGF-TSP1 complex was proved on promoting FLS proliferation, migration, and angiogenesis in vitro and deteriorating FLS invasion and joint damage in SCID mice.

**Conclusions:**

TSP-1 is the essential domain in CTGF/ANXA2 interaction and contributes to FLS migration and pannus formation, inducing the process of RA.

**Supplementary Information:**

The online version contains supplementary material available at 10.1186/s13075-021-02656-y.

## Introduction

Rheumatoid arthritis (RA) is a chronic autoimmune inflammatory disorder with a multifactorial etiology, characterized by persistent synovitis and pannus formation [1]. Pannus, an indisputable sign of invasive synovial tissue, enhances synovial vascularity through synovium thickening and angiogenesis [2–5]. As a key component thereof, the overabundant fibroblast-like synoviocyte (FLS) secret matrix metalloproteinases (MMPs) [6–8], chemokines [9], and cytokines (e.g., IL-6, IL-18) thereby modulate growth, inflammation, angiogenesis, and cell recruitment [3]. Furthermore, pannus angiogenesis adversely affects the biomechanical properties of the cartilage, which ultimately leads to cartilage destruction and joint damage [10]. However, the effect of abnormal angiogenesis and FLS migration in the mechanism of pannus formation is still poorly understood.

Connective tissue growth factor (CTGF), also known as CCN family protein 2 (CCN2), is composed of four domains, including insulin-like growth factor binding protein-like (IGFBP), von Willebrand factor type C repeat (VWC), thrombospondin type 1 repeat (TSP1), and C-terminal cystine-knot (CT) modules [11]. It is a cysteine-rich matricellular protein involved in many biological events, including cell adhesion, proliferation, and angiogenesis. Some evidence revealed the involvement of CTGF in the onset of RA [12, 13]. In our previous proteomic study and the subsequent validation experiments, CTGF was found to be significantly elevated in FLS of 50 RA patients compared with that of 50 healthy controls [14]. Our previous study unveiled the diagnostic value of CTGF in RA, with a high sensitivity and a high specificity, which made it helpful on early diagnosis and distinguishing diagnosis [15]. A study by Nozawa et al. showed that anti-CTGF mAb treatment prevented the progression of arthritis in collagen-induced arthritis (CIA) mice through the suppressive effects on T cell proliferation and Th17 differentiation, indicating that CTGF may become a new target for the treatment of RA [16]. However, the expression of CTGF in the lesion region (synovial tissue) and its function in pannus formation remain unclear. Moreover, we identified the elevated expression of CTGF in RA synovial tissues and validated that overexpressed CTGF could enhance the proliferation and migration of human umbilical vein endothelial cells (HUVECs) by MTT and transwell assays [14]. But the effect of CTGF on FLS proliferation and migration and the molecular mechanism of CTGF-enhanced pannus formation require more investigations in vivo.

Annexin A2 (ANXA2) is a slightly curve-shaped protein consisting of a highly conserved core domain of four homologous repeats capable of binding calcium, phospholipids, heparin, and F-actin and a unique N-terminal interaction domain with the binding sites of S100A10, tissue plasminogen activator (tPA), and phosphorylation sites [17]. Many studies showed that ANXA2 could promote angiogenesis. The mechanisms for ANXA2 regulation of retinal and corneal angiogenesis have been detailed [18, 19], but there are few studies that focus on the mechanism of ANXA2 on RA pannus angiogenesis. Yi et al. demonstrated that ANXA2 could promote angiogenesis [20], but only focused on the interaction of exogenous AXNA2 and AXNA2R in HUVEC. To this end, endogenous ANXA2 may also play a critical role in the angiogenic response, since it can modulate many biological processes, such as vesicle trafficking and fusion [21]. Besides, the agents interacting with ANXA2 are subject to an ongoing discussion, and the function of their interaction in RA remains unanswered. Therefore, focusing on ANXA2-interacting agents may reveal novel mechanisms that conspire to ANXA2 in pathologic angiogenesis.

In this study, proteins that interacted with CTGF in FLS derived from RA patients were identified. The protein complex from co-immunoprecipitation (Co-IP) using anti-CTGF mAb was subjected to LC-MS/MS analysis. The interaction of ANXA2 and the TSP1 domain of CTGF was identified and further confirmed through Co-IP and bimolecular fluorescence complementation (BiFc) assay. Additionally, the combination of the TSP1 domain and ANXA2 was also verified using homology modeling and molecular docking. The high stability of the complex was successfully assessed by molecular dynamic simulation. We then explored the effect of the interaction of CTGF and ANXA2 in vitro and further verified it using severe combined immunodeficiency (SCID) mouse model in vivo, thereby shedding light into the mechanism of pannus formation in the process of RA.

## Material and methods

### Patients and samples

Synovial tissues were obtained from RA patients who underwent synovectomy or joint replacement surgeries and normal subjects who received high amputations at the First Affiliated Hospital of Wenzhou Medical University from March 2017 to December 2019. RA diagnosis was based on the 2010 ACR criteria. More clinical details of the patients were provided in Supplementary Table S1.

### Cell culture and co-immunoprecipitation

Synovial tissue was isolated enzymatically according to a previously described method [22], digested with 5 mg/ml collagenase (Sigma) and 1.5 mg/ml DNase (Sigma), and passed through a wire mesh to prepare isolated cells. FLS derived from synovial tissues were cultured with DMEM supplemented with 10% fetal bovine serum in a humidified atmosphere of 5% CO_2_ at 37 °C. FLSs were lysed in the RIPA lysis buffer containing protease inhibitors on the ice for 30 min followed by being centrifuged at 5000 rpm for 10 min. The supernatant containing the protein complex was incubated with anti-CTGF mAb or anti-ANXA2 mAb and that from HEK293T cells with anti-ANXA2 mAb or anti-FLAG mAb at 4 °C with gentle shaking. After incubating the supernatant with protein A agarose beads at 4 °C for 4 h with gentle shaking, the mixture was placed in a magnetic field for 5 min. The separated beads were then washed with PBS followed by being boiled in 2× SDS loading buffer for 5 min. The protein mixtures in the supernatant were further analyzed by western blotting.

### LC-MS/MS analysis

Proteins in gel pieces from Co-IP were analyzed by Nano-LC-MS/MS analysis which was performed on a Q Exactive mass spectrometer (Thermo Scientific) coupled to Easy nLC (Thermo Fisher Scientific). For more details of the analysis, please refer to the supplementary method. MS/MS spectra were searched using MASCOT engine (Matrix Science, London, UK; version 2.2) against a nonredundant International Protein Index arabidopsis sequence database v3.85 (released in September 2011; 39,679 sequences) from the European Bioinformatics Institute (http://www.ebi.ac.uk/).

### Bimolecular fluorescence complementation (BiFC) assay

All constructs for BiFC assays were prepared in pBiFC-VC155 and pBiFC-VN155 (I152L) vector and were co-transfected into HEK293T cell; specifically, CTGF-full length and CTGF-∆TSP1 were ligated into the pBiFC-VC155 vector, and ANXA2-full length was ligated into the pBiFC-VN155 (I152L) vector. The co-transfection of bFosDetaZIPVC155 and pBiFC-bJunVN173 vector was used as a negative control and the co-transfection of pBiFC-bFosVC155, and the pBiFC-bJunVN173 vector was used as a positive control according to a previous study [23]. The primer sequences were listed as Supplementary Table S2. The co-transfected HEK293T cells were cultured in a 6-well plate. After 48 h of cultivation, fluorescent images were obtained using Leica DMIRE2 inverted fluorescence microscope.

### Lentiviral transfection, MTT assay, and scratch assay

RA FLS was derived as mentioned above, and knockdown (KD) of CTGF in RA FLS was performed using shRNA. Then, the CTGF-KD FLS were divided into five groups and infected with lentiviruses containing Flag-CTGF-full length, Flag-CTGF-∆TSP1, ANXA2-shRNA+Flag-CTGF-full length, ANXA2-shRNA+Flag-CTGF-∆TSP1, and control. Briefly, GV309 lentivirus vector and poly-brene (50 μg/ml; Sigma-Aldrich) were added in a 6-well plate containing 5 × 10^4^ CTGF-KD FLS cells. After 16 h, the transfection medium was replaced by RPMI 1640 containing 10% fetal bovine serum. Then, the cells were digested for 3-(4,5-dimethylthiazol-2-yl)-2,5-diphenyltetrazolium bromide (MTT) and scratch assay.

In the MTT assay, cells were seeded onto 96-well plates and cultured in the DMEM supplemented with 10% FBS until 90% confluence. FLS were then incubated with MTT for 4 h. After the centrifugation at 3000 rpm for 10 min, DMSO was added into the wells to dissolve the formazan followed by the detection at the absorbance of 490 nm using Bio-Rad iMark™ microplate reader.

In the scratch assay, FLS were seeded onto 6-well plates. After 24 h, each well was scratched using a pipette tip in the middle and photographed every 12 h using an inverted microscope.

### Tube formation assay

DMEM medium and Matrigel were equally mixed firstly. Twenty-four-well plates were coated with 200 μl Matrigel (BD, Oxford, UK) per well, which were then kept in a 37 °C incubator for 1 h to solidify. HUVECs were first serum-starved for 24 h and then resuspended in DMEM after centrifugation. Next, lentiviruses containing Flag-CTGF-full length, Flag-CTGF-∆TSP1, and ANXA2-shRNA+Flag-CTGF-full length were administered to HUVECs. The HUVECs were then added to the Matrigel-coated wells (1.0 × 10^4^ cells/well in 200 μl of DMEM), and the pictures of each well were observed and photographed after 6 h.

### The chick chorioallantoic membrane (CAM) assay

The 36 fertilized eggs were incubated at 37 °C with a humidity of 40~60%. On the 7th day of incubation, silicone rings were placed on the CAM surface. All the eggs were randomly divided into 3 groups. Next, lentiviruses containing Flag-CTGF-full length, Flag-CTGF-∆TSP1, and ANXA2-shRNA+Flag-CTGF-full length were administered to eggs. The vessels were observed through a stereomicroscope.

### FLS-cartilage-SCID model construction

FLS was prepared as mentioned above. The normal human cartilage was cut into 5–8 mm^3^ pieces, which were obtained from the patients undergoing knee surgery for traumatic injuries. Then, the sterile sponge soaked with 5 × 10^5^ FLS prepared above and a piece of cartilage were inserted under the skin altogether at the left flank of a 4-week-old SCID mouse under sterile conditions. After 60 days, mice were sacrificed, and the implants were removed and embedded in Tissue-Tek OCT compound (Miles, Elkhart, IN) and snap-frozen in liquid nitrogen immediately for HE staining. At the same time, the knee joints of all the SCID mice were dissected and maintained in 4% paraformaldehyde for 24 h followed by the decalcification in the disodium EDTA (pH = 7.2~7.3) for 4~5 weeks (the decalcification solution was changed once a week). The decalcified joint tissues were embedded in paraffin and cut into 5 μm sheets.

### Hematoxylin and eosin (H&E) staining of implant and knee joint

After fixation, the implant sections and the mouse knee joint sections were stained using standard hematoxylin, and high-resolution images were captured on a Nikon photomicroscope.

### Statistical analysis

Statistical analysis was conducted in GraphPad Prism 8 (GraphPad Software, La Jolla, CA), and the data were presented as the mean ± *SD*. The Shapiro-Wilk method was used to determine whether the data were normally distributed and the homogeneity of variance was tested by the Levene method. The differences between the two groups of data which met the normal distribution and homogeneity of variance were analyzed using the unpaired, two-tailed Student’s *t*-tests. One-way analysis of variance test with post hoc contrasts by Tukey test was applied to compare the means of multigroups.

## Results

### The expression of CTGF increased in RA patients

**Fig. 1:**
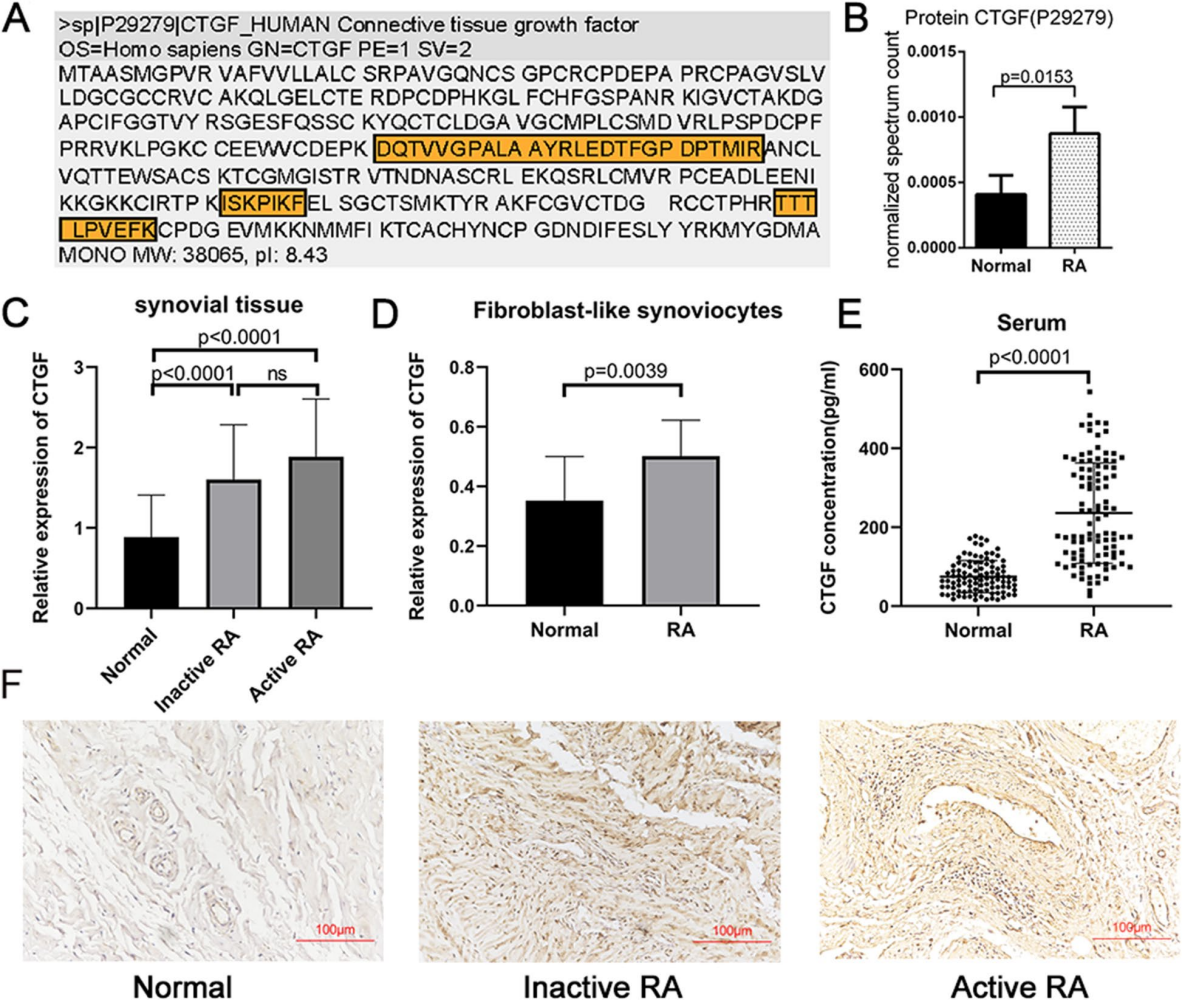
Overexpression of CTGF in RA patients. **A** The different expression of CTGF in FLSs of RA patients (*n* = 50) and HCs (*n* = 10) were identified by automated 2D-Nano-LC-ESI-MS/MS. The amino acid coverage of identified CTGF sequence is shown with the appraisal sequence in yellow. **B** The normalized spectrum count of CTGF in RA patients and HCs. **C** The level of CTGF in the synovial tissues of inactive RA patients (DAS28 < 3.32, *n* = 45), active RA patients (DAS28 > 3.32, *n* = 47), and HCs (*n* = 50) were detected by qPCR. **D** The level of CTGF in the FLS of RA patients (*n* = 16) and HCs (*n* = 16) was detected by qPCR. **E** The concentration of the serum CTGF from RA patients (*n* = 100) and HCs (*n* = 100) was determined by ELISA. **F** The expression of the CTGF of synovial tissue sections from RA patients and health controls were determined by IHC with a magnification of × 200

The proteins extracted from FLS of synovial tissues in RA patients and healthy controls (HCs) were analyzed by the automated 2D-Nano-LC-ESI-MS/MS, which subsequently identified human CTGF with high amino acid coverage (Fig. 1A). The analysis of the normalized spectrum showed that CTGF was overexpressed in FLS derived from RA patients compared with HCs (Fig. 1B). Furthermore, the mRNA expression of CTGF detected by qPCR was higher in inactive and active RA patients than in healthy controls. However, no significant difference was observed between inactive RA and active RA (Fig. 1C). As expected, compared with HCs, the overexpression of CTGF was further validated by qPCR in RA FLS (Fig. 1D). In addition, the concentration of CTGF was higher in RA serum determined by ELISA compared with HCs (Fig. 1E). The overexpression of CTGF on synovial tissue sections from RA patients was validated by immunohistochemical (IHC) staining, most distributed around the blood vessels (Fig. 1F). Considering all the findings above, we claimed that CTGF expression increased in RA patients.

### Identification of ANXA2 as a partner interacting with CTGF

**Fig. 2:**
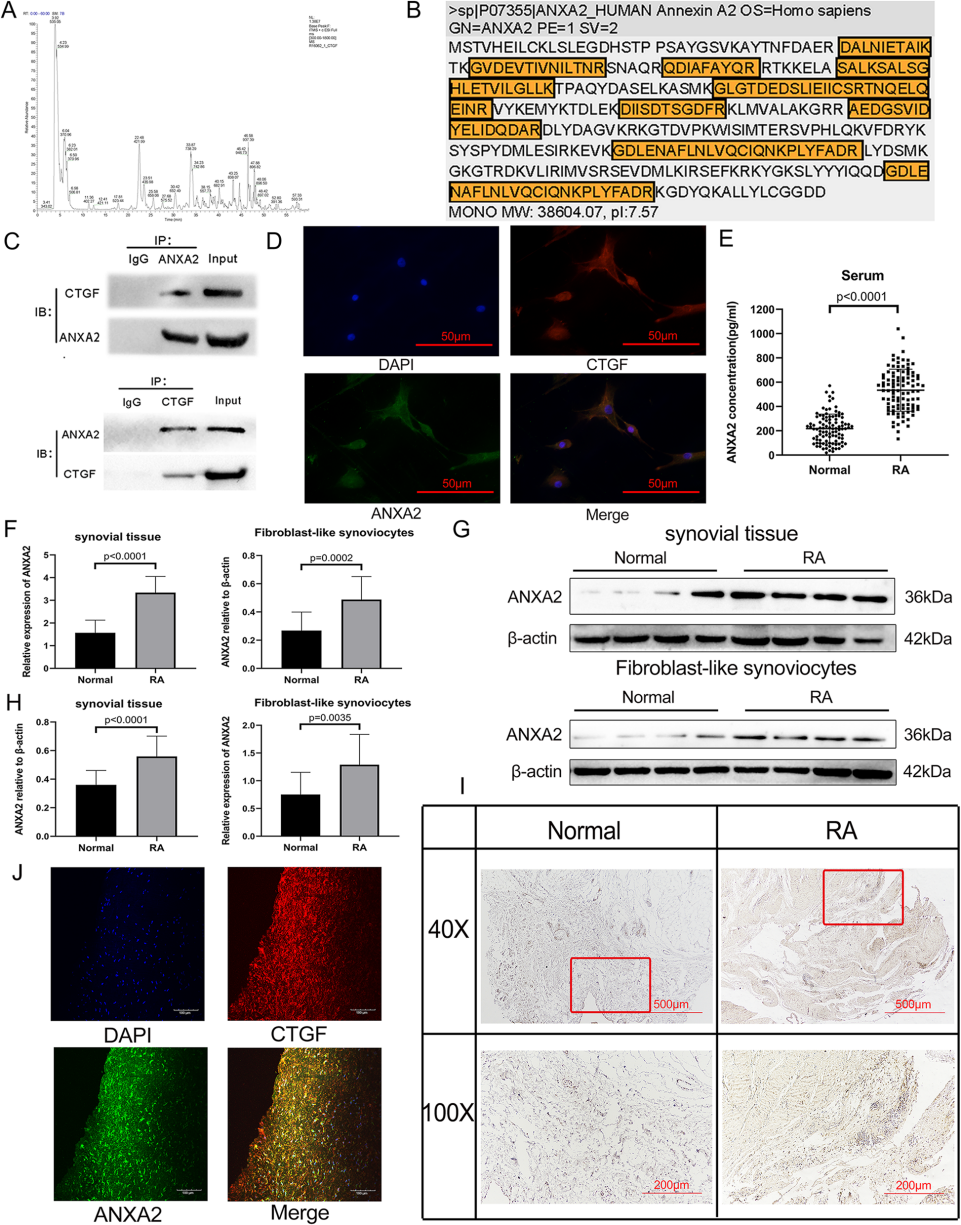
ANXA2 is the specific protein that interacts with CTGF. **A** Base peak chromatography from LC-MS/MS analysis of the proteins separated by SDS-PAGE gel electrophoresis after Co-IP using anti-CTGF antibody with FLSs lysates. IgG was used as a control. **B** The amino acid coverage of the identified ANXA2 sequence is shown with the appraisal sequence in yellow. **C** Co-IP using anti-ANXA2 and anti-CTGF antibodies with FLS lysates. **D** Immunofluorescence of CTGF and ANXA2 was performed in FLSs with a magnification of × 400. **E** The concentration of the serum ANXA2 from RA patients (*n* = 100) and HCs (*n* = 100) was determined by ELISA. **F** The expression of ANXA2 mRNA in synovial tissues and FLSs from RA patients (*n* = 16) and HCs (*n* = 16) were detected by qPCR. **G**, **H** The expression of ANXA2 in synovial tissues and FLSs from RA patients (*n* = 16) and HCs (*n* = 16) were measured by WB. **I** The expression of the ANXA2 of synovial tissue sections from RA patients and HCs were determined by IHC with a magnification of × 40 and × 200. **J** Immunofluorescence of CTGF and ANXA2 performed in synovial tissues of RA patients with a magnification of × 200

In order to screen potential CTGF interacting proteins, Co-IP were performed using anti-CTGF mAb in RA FLS lysates. Then, the protein was eluted from the beads, separated by SDS-PAGE electrophoresis, and subsequently detected by LC-MS/MS analysis (Fig. 2A). Of note, the proteins identified from LC-MS/MS were listed in Supplementary Table S3 among which human ANXA2 was evaluated with high amino acid coverage (Fig. 2B). Next, the interaction of CTGF with ANXA2 was further confirmed by Co-IP using anti-CTGF mAb and anti-ANXA2 mAb (Fig. 2C). Co-localization of CTGF and ANXA2 in FLS was observed by immunofluorescence (IF) (Fig. 2D). In summary, these results identified ANXA2 as a binding partner of CTGF.

### The expression of ANXA2 increased in RA patients

Compared with HCs, the concentrations of ANXA2 were higher in RA serum determined by ELISA (Fig. 2E). Consistent with these findings, the overexpression of ANXA2 was further validated by qPCR (Fig. 2F) and WB (Fig. 2G, H) both in the synovial tissues and FLS of RA patients compared with HCs. In addition, elevated expression of ANXA2 was also observed in synovial tissues of RA patients by IHC staining, which mainly expressed around the blood vessels (Fig. 2I). Moreover, co-localization of CTGF and ANXA2 in synovial tissues of RA patients was observed by IF (Fig. 2J).

### The TSP1 domain of CTGF is essential in the interaction of CTGF and ANXA2

**Fig. 3:**
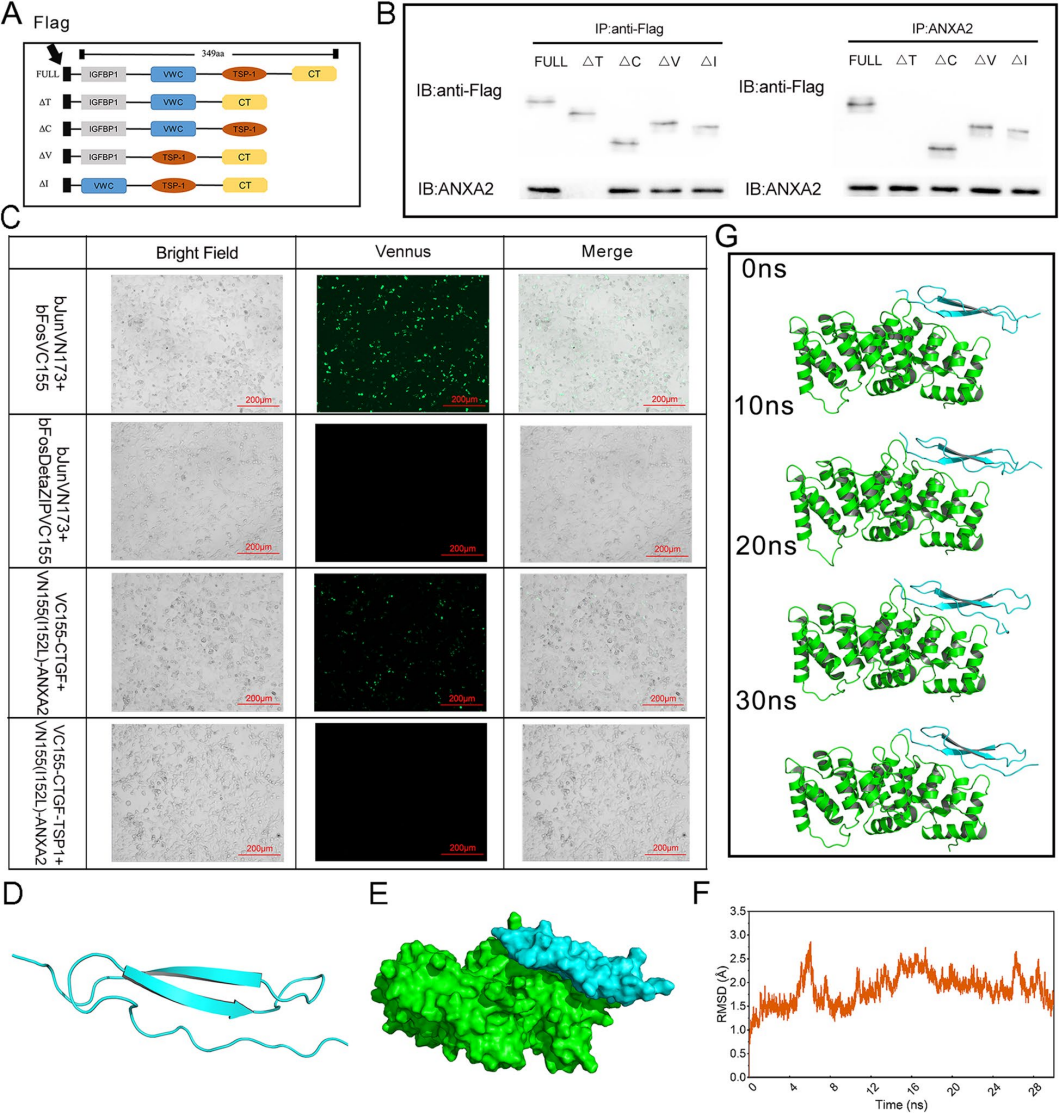
The TSP1 domain of CTGF is the direct region that interact with ANXA2. **A** Schematic representations of full-length CTGF and its deletion mutants. **B** Co-IP using anti-ANXA2 and anti-FLAG antibodies with lysates of various deletion mutants transfected into HEK293T cells. **C** Fluorescence expression of pBiFC-VN155 (I152L)-ANXA2 co-transfected with pBiFC-VC155-CTGF or pBiFC-VC155-CTGF-∆TSP1 in HEK293T cells at a magnification of × 200. **D** Structure of TSP1 domain of CTGF by homology modeling. **E** Predicted binding mode of ANXA2/CTGF-TSP1, green and blue represent the ANXA2 and CTGF-TSP1 domain regions, respectively. **F** Root mean square deviation (RMSD) of the backbone atoms (C, CA, and N) of the ANXA2/CTGF-TSP1 complex as a function of time. **G** Conformations of the complex during molecular dynamics simulation

To further clarify which domain of CTGF was involved in ANXA2/CTGF interaction, we constructed several CTGF mutants (Fig. 3A). Next, Co-IP assays were performed using HEK293T cells transfected with these Flag-tagged mutants (Fig. 3B). The result showed that TSP1 domain of CTGF played an essential role in the interaction between CTGF and ANXA2. To further verify the function of the interaction in vivo, we subcloned CTGF-full length or TSP1-deleted mutants and ANXA2 into bimolecular fluorescent vectors pBiFC-VC155 and pBiFC-VN (I152L) vector, respectively, and BiFC was performed by co-transfecting them into HEK293T cells. As expected, VC155-CTGF and VN (I152L)-ANXA2 co-transfection group observed fluorescence, and no significant signal was detected in the VC155-CTGF-∆TSP1 group (Fig. 3C). These findings suggested that CTGF directly interacts with ANXA2, and the TSP1 domain of CTGF protein is necessary for the interaction.

### Homology modeling and computational analysis of ANXA2/CTGF-TSP1 combination

To explore the combining conformation of CTGF-TSP1 and ANXA2 at the molecular level, a multilevel computational analysis was performed. The structure of the TSP1 domain of CTGF protein is shown (Fig. 3D), and then CTGF and ANXA2 are successively docked and their conformations are appropriately selected according to the ZDOCK score (948.942) (Fig. 3E). To further assess the stability of the ANXA2/CTGF-TSP1 complex structure in the biomimetic environment, we conducted a 30-ns molecular dynamics (MD) simulation and tracked the RMSD variation of the complex (Fig. 3F). After 10 ns, ANXA2/CTGF-TSP1 tended to be stable in the bionic circumstance with an RMSD value of 1.99 ± 0.24 Å. Next, during a 30-ns simulation, four snapshots of MD trajectory further characterized the conformational fluctuations of the ANXA2/CTGF-TSP1 complex (Fig. 3G). Additionally, as expected, no obvious conformational changes occurred during the simulation, which suggested a strong interaction between the CTGF-TSP1 domain and ANXA2 with stable combination.

### ANXA2/CTGF-TSP1 complex induced FLS proliferation, migration, and angiogenesis

**Fig. 4:**
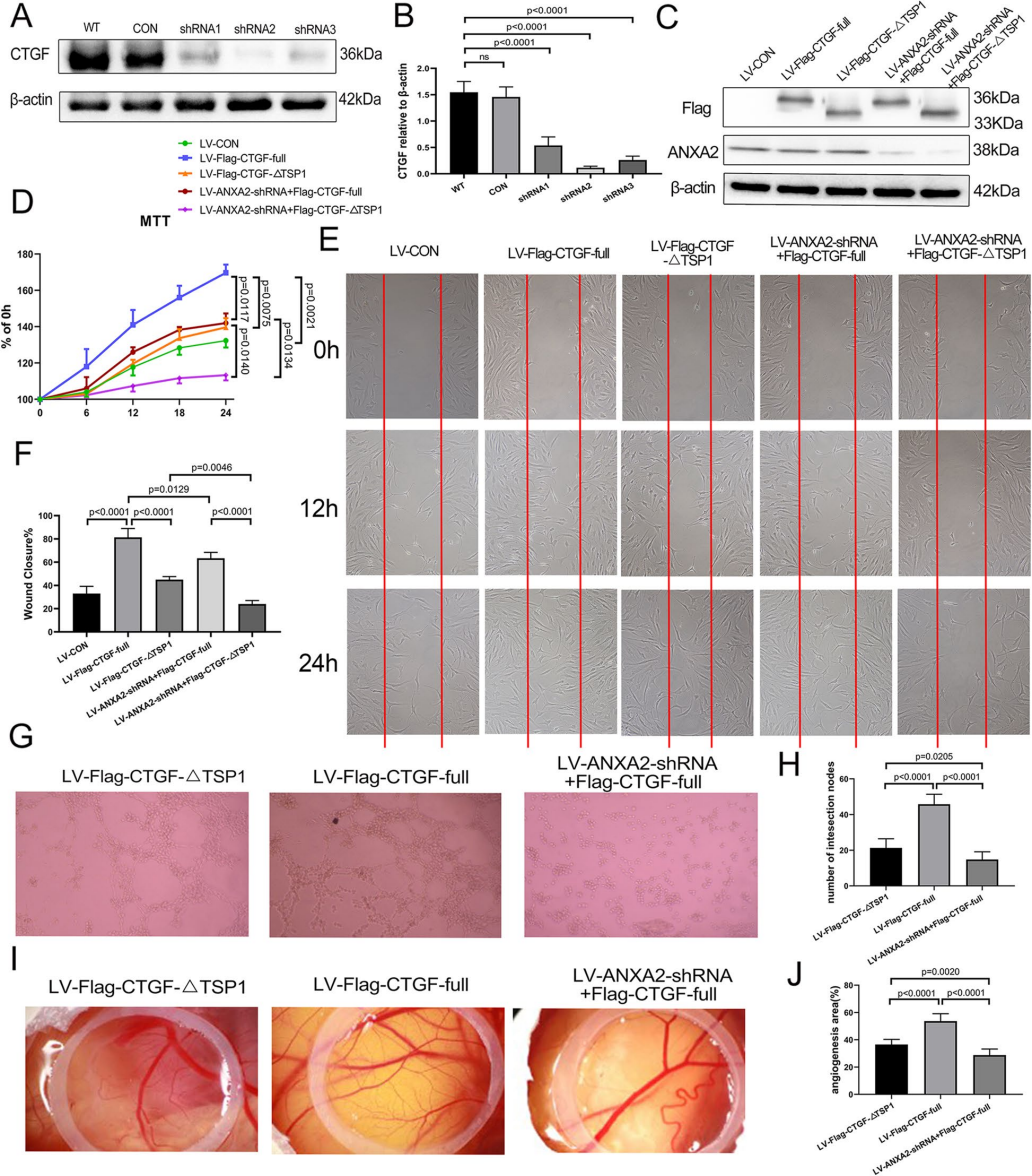
The ANXA2/CTGF-TSP1 complex is required on cell proliferation, migration, and angiogenesis in vivo and in vitro. **A**, **B** The expression of CTGF in wild-type FLS and CTGF-KD FLS were measured by WB. The experiments were repeatedly performed using three independent samples. **C** The expression of Flag-CTGF/CTGF-∆TSP1 and ANXA2 in CTGF-KD FLS transfected with lentivirus (LV-CON, LV-Flag-CTGF-∆TSP1, LV-Flag-CTGF-full length, LV-sh-ANXA2+Flag-CTGF-full length, LV-sh-ANXA2+Flag-LV-CTGF-∆TSP1) were measured by WB. **D** MTT assay. CTGF-KD FLS were transfected with lentivirus (groups mentioned above in Fig. 4C, *n* = 3) and then incubated for 6 h, 12 h, 18 h, and 24 h. The absorbance value of each well at the 490-nm wavelength was measured. **E** Scratch migration assay. CTGF-KD FLS were seeded on a six-well plate and transfected with lentivirus (groups mentioned above in **C**, *n* = 3). After 24 h, a scratch was made along the diameter of the well using a pipette tip. An image was captured every 12 h by a camera under an inverted microscope (original magnification × 40). **F** The percentage of wound closure at 24 h was quantified as (area of original wound − area of measured wound)/area of original wound × 100%. **G** Tube formation assay. HUVECs were transfected with lentivirus (LV-Flag-CTGF-full length, LV-Flag-CTGF-∆TSP1, and LV-sh-ANXA2+Flag-CTGF-full length) and then incubated for 6 h, and then tube formation was observed and photographed (*n* = 10). **H** The number of intersections among the branches of assembled HUVEC networks was calculated in the whole field. **I** CAM assay. CAMs were treated with lentivirus (groups mentioned above in **G**) on day 7, and all CAMs were photographed 3 days later (*n* = 10). **J** The percentage of angiogenetic area = vascular area/CAM area × 100%. ImageJ was used to assess the vascular and CAM areas

It was generally reckoned that synovial hyperplasia and angiogenesis played an important role RA process. Our previous study has also verified the destructive role of CTGF in FLS proliferation, migration, and angiogenesis in RA [14, 15]. Therefore, we went on to explore the effect of the ANXA2/CTGF-TSP1 complex on FLS proliferation and migration. Three different CTGF shRNA lentiviral vectors were constructed and transfected into RA FLSs, and the shRNA2 group showed the most significant reduction by WB (Fig. 4A, B). Then, we constructed Flag-CTGF-full length and Flag-CTGF-∆TSP1 and rescued them in the CTGF-KD FLS. Besides, no significant difference was observed on ANXA2 expression both in Flag-CTGF-full length groups and control group, which indicated that CTGF cannot influence ANXA2 expression (Fig. 4C). Therefore, following the results above, we hypothesized that it should be the complex of ANXA2 and CTGF that induce RA process, and we performed cell proliferation and migration experiments. According to the results, the rescue of CTGF-full length promoted FLS proliferation and migration, but the rescue of CTGF-∆TSP1 showed no significant change compared to the control. Moreover, the knockdown of ANXA2 caused by co-transfecting LV-sh-ANXA2 suppressed FLS proliferation and migration compared to the rescue of CTGF or CTGF-∆TSP1 alone (Fig. 4D–F). Then, the angiogenesis ability of the ANXA2/CTGF complex was evaluated by tube formation experiment in vitro and CAM assays in vivo (Fig. 4G–J). As expected, an extreme loss of angiogenesis function was found in the ∆TSP1 group compared with the full-length group, and the knockdown of ANXA2 also showed a similar loss. According to these findings, we indicated that the ANXA2/CTGF-TSP1 complex played a role in FLS proliferation, migration, and angiogenesis.

### ANXA2/CTGF-TSP1 complex aggravated inflammatory invasion in SCID mouse model

**Fig. 5:**
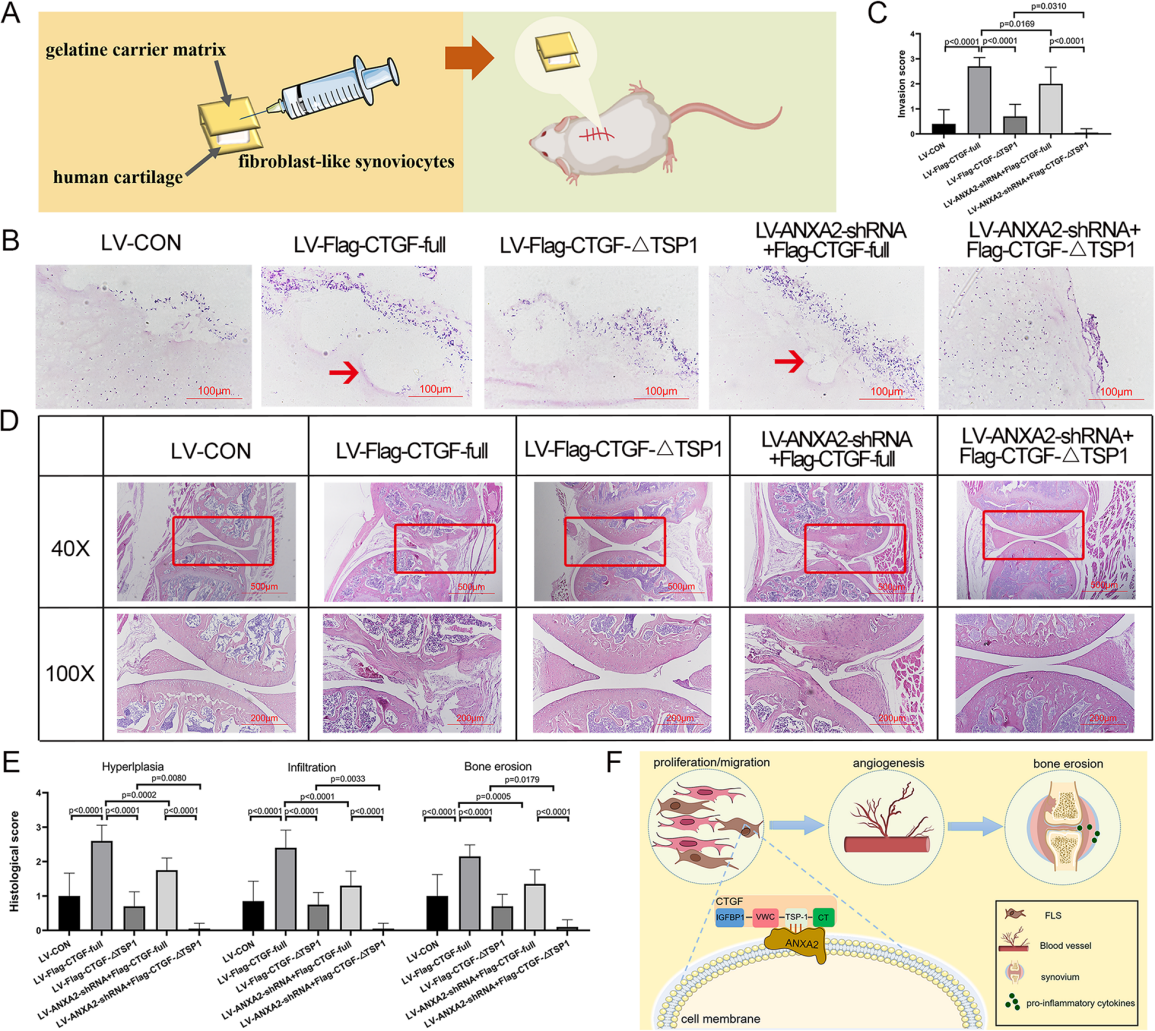
The ANXA2/CTGF-TSP1 complex is responsible for the invasion and migration of RA FLS in the SCID model. Cartilage graft injected with the FLS (treated as methods above) was transplanted into SCID mice (*n* = 10 mice per group). **A** The schematic protocol for the FLS-cartilage-SCID model (arrow depicts the site of transplants.) **B** H&E staining of the cartilage removed from SCID mice with a magnification of × 200. The arrows indicate RA FLS invaded into the cartilage. **C** Measurement of invasion of RA FLS into human cartilage implants transferred under the skin of SCID mice. The level of invasiveness was scored as follows: 0 = no or minimal invasion, 1 = visible invasion (two-cell depth), 2 = invasion (five-cell depth), and 3 = deep invasion (more than ten-cell depth). The sections were examined by two experienced scientists in a blinded manner. **D** H&E staining of the knee joints of the SCID mice (original magnification, up × 40 and down × 100). **E** Histological scores for synovial infiltration, synovial hyperplasia, and cartilage and bone damage were assessed and graded on a scale of 0 (normal) to 3 (severe). **F** Interaction between the ANXA2 and TSP1 domain of CTGF forces FLS migration and pannus formation in the RA process

To further verify the effects of the ANXA2/CTGF-TSP1 complex in the RA process, we constructed a SCID mouse co-implantation model to assess the severity of invasion and inflammation (Fig. 5A). The results indicated that the rescue of Flag-CTGF-full length showed a significant induction of cartilage inflammatory invasion compared to CTGF-KD RA FLS, but the rescue of CTGF-∆TSP1 showed no obvious induction of cartilage inflammatory invasion. Additionally, ANXA2-KD exhibited obviously milder invasion compared to the rescue of Flag-CTGF-full length or Flag-CTGF-∆TSP1 alone (Fig. 5B, C). Moreover, the rescue of Flag-CTGF-full length deteriorated synovial hyperplasia, inflammatory cell infiltration, and cartilage erosion and increased the histological score of the knee joint compared to the control, but the rescue of Flag-CTGF-∆TSP1 showed no significant change. Additionally, the destruction of the knee joint was maximally attenuated with ANXA2-KD compared to the rescue of Flag-CTGF-full length or Flag-CTGF-∆TSP1 alone (Fig. 5D, E). In conclusion, the results above indicated that the ANXA2/CTGF-TSP1 complex forced FLS proliferation and migration to enhance angiogenesis and bone erosion (Fig. 5F).

## Discussion

Rheumatoid arthritis (RA) is a chronic autoimmune disease characterized by persistent synovitis and pannus formation, which result in cartilage destruction and bone erosion [1]. So far, anti-pannus formation treatment has been clinically discussed, however, target at it, a safe and effective therapeutic method is far from well-developed.

CTGF is a well-studied protein in arthritis; many studies confirmed its function on pannus formation. In our previous proteomic study, CTGF was screened as a hub-gene increased in RA patients [14]. Herein, we showed an increased expression of CTGF in RA patients even at an early stage, and no significant difference between the active and inactive groups confirm that CTGF can be used as an early RA diagnostic indicator, but not a RA activity staging indicator [15]. Functionally, Ding et al. and our previous study altogether indicated CTGF promoted FLS proliferation and migration and suppression of CTGF ameliorated neovascularization, suggesting CTGF induces pannus formation in RA [13, 14]. However, the detailed mechanism is still poorly discussed.

In recent years, the protein interaction network of CTGF raised wild concerns. Angiogenesis-related factors, such as VEGF and integrin family proteins, have been confirmed to interact with CTGF [24–26]. Therefore, we hypothesized that CTGF may induce pannus formation through protein binding and investigated CTGF interacted proteins in FLS using LC-MS/MS-based interactome analysis; eventually, ANXA2 was selected.

ANXA2 is a key protein implicated in cell proliferation, migration, and invasion [27–29]. Specifically, in RA patients, ANXA2 expression were higher, and in the CIA mouse model, the overexpression of AXNA2 promoted neovascularization, indicating that ANXA2 may induce RA pannus formation [20]. Thus, it is obvious that ANXA2 and CTGF have a coincide effect on RA progress, but no study has reported their combination before. In this study, we confirmed a solid interaction between CTGF and ANXA2 by Co-IP and further focused on the exact domain of CTGF that combines ANXA2.

Although the three-dimensional (3D) structure of CTGF is still unclear, the 3D structure and biological function of CTGF domains were predicted and explored [30]. Molecular biological studies suggest four specific domains of CTGF, including IGFBP, VWC, TSP1, and CT, can interact with a variety of molecules, including cytokines, growth factors, receptors, and matrix proteins [30–34]. Importantly, a controversial effect of the TSP-1 domain of CTGF on angiogenesis was also raised. Inoki et al. reported that the TSP-1 domain of CTGF could bind VEGF and inhibits its function, which leads to the decreased angiogenic activity [26]. Conversely, another study reported that CTGF-targeting mAb inhibits angiogenesis induced by each module of CTGF including TSP-1, suggesting that the TSP-1 domain of CTGF promoted angiogenesis [35]. In this study, we confirmed a new protein ANXA2 that could be combined with the TSP-1 domain of CTGF, in addition to the three known proteins (Integrin α6β1, LRP, and VEGF), which can promote angiogenesis. Of note, we predicted the structure of ANXA2, CTGF-TSP1, and their complex by homology modeling, and the high stability of the complex was observed using dynamics simulation. Furthermore, we investigated the function of this complex. CTGF was the first knockdown in FLS and then rescued with CTGF-full length or CTGF-∆TSP1 lentivirus, co-transfected with ANXA2-shRNA, and performed MTT, cell scratch, and tube formation experiments. The result verified the interaction of the CTGF-TSP1 domain and ANXA2 could induce FLS proliferation, migration, and angiogenesis. To further confirm, we constructed an in vivo FLS-cartilage-SCID mouse model, a well-established model allowing quantification of FLS invasion and assessment of human cartilage destruction [36, 37]. We found decreased pannus formation, characterized by moderate synovial hyperplasia and less inflammatory infiltration, in the CTGF-TSP1 domain deleted and ANXA2 deficient group. According to these results, we demonstrate the interaction of the CTGF-TSP1 domain, and ANXA2 contributes to the proliferation and migration of RA FLS and angiogenesis, which finally leads to pannus formation.

In summary, our study raises the importance of the CTGF interaction network in RA pannus formation, states a new CTGF-combined protein ANXA2, and determines the exact binding site TSP1 domain as well as the function of CTGF-ANXA2 complex on FLS proliferation, migration, and angiogenesis. Indeed, the downstream mechanism of the CTGF-ANXA2 complex that induces angiogenesis needs further exploration. Our data shed light on the cross-talk in the FLS population, and this might help to better understand the cellular interactions during pannus formation and provide new insights into the pathogenesis of rheumatoid arthritis.

## Conclusions

In summary, our results indicated that the TSP1 domain of CTGF was necessary for the interaction with ANXA2. Their cooperation enhanced both proliferation and migration of RA FLS and angiogenesis, as well as pannus formation in the FLS-cartilage-SCID model.

## Supplementary Information

**Additional file 1: Figure S1.** Analysis of the ELISA results revealed no linear correlation between CTGF and ANXA2 with *r*=0.08627, *p*>0.05.

**Additional file 2.** Supplementary methods.

**Additional file 3: Table S1.** Demographic and clinical characteristics of the patients with RA and healthy controls. Values are expressed median (minimum, maximum) unless state otherwise. Abbreviations: ACPA, anticitrullinated protein antibodies; CRP, C reactive protein; DAS-28, disease activity score 28 joints; ESR, erythrocyte sedimentation rate; RF, rheumatoid factor; NA, not applicable.

**Additional file 4: Table S2.** Sequences of qRT-PCR primers, cDNA primers and shRNA-targeting genes.

**Additional file 5: Table S3.** LC-MS/MS identification of proteins isolated from FLS lysates of RA patients by immunoprecipitation using Anti-CTGF antibody.

## Data Availability

The datasets used and/or analyzed during the current study are available from the corresponding author on reasonable request.

## Abbreviations

ANXA2: Annexin A2
CAM: Chick chorioallantoic membrane
CTGF: Connective tissue growth factor
CO-IP: Co-immunoprecipitation
ELISA: Enzyme-linked immunosorbent assay
FLS: Fibroblast-like synoviocytes
HE: Hematoxylin and eosin
HUVEC: Human umbilical vein endothelial cells
IHC: Immunohistochemical staining
IF: Immunofluorescence
LC-MS/MS: Liquid chromatography-tandem mass spectrometry
qPCR: Real-time quantitative PCR
RA: Rheumatoid arthritis
SCID: Severe combined immunodeficiency
△TSP1: TSP1-deleted domain
