# Supplementary figures and images for: The protein-protein interaction between connective tissue growth factor and annexin A2 is relevant to pannus formation in rheumatoid arthritis
Source: Arthritis Res Ther. 2021 Oct 26;23:266. doi: 10.1186/s13075-021-02656-y (PMC8547044; doi:10.1186/s13075-021-02656-y)

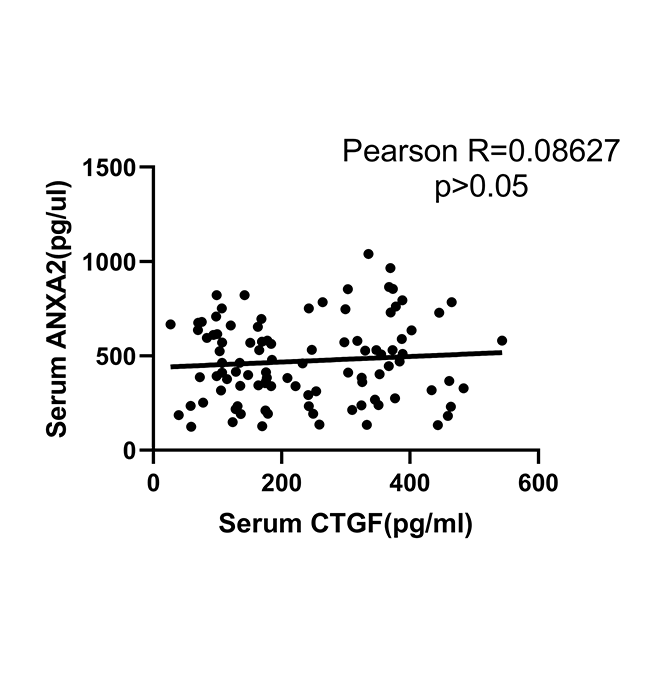

Supplement: Supplementary file 1 — Additional file 1: Figure S1. Analysis of the ELISA results revealed no linear correlation between CTGF and ANXA2 with r=0.08627, p>0.05. [file 13075_2021_2656_MOESM1_ESM.tif]
