# Supplementary material for: The protein-protein interaction between connective tissue growth factor and annexin A2 is relevant to pannus formation in rheumatoid arthritis
Source: Arthritis Res Ther. 2021 Oct 26;23:266. doi: 10.1186/s13075-021-02656-y (PMC8547044; doi:10.1186/s13075-021-02656-y)
Supplement: Supplementary file 2 — Additional file 2. Supplementary methods. [file 13075_2021_2656_MOESM2_ESM.docx]

**Supplementary method**

**LC-MS/MS Analysis**

Gel pieces were cut from SDS-PAGE and destained with 30% ACN/100 mM NH_4_HCO_3_.After being reduced and alkylated, gel pieces were digested overnight in 12.5 ng/μl trypsin in 25 mM NH_4_HCO_3_ followed by being extracted three times with 60% ACN/0.1% TFA. The peptides were separated again on preparative gels and then visualized by silver staining. Protein spots of interest were cut, destained in 30 mM potassium ferricyanide/100mM sodium thiosulfate (1:1 v/v), washed and lyophilized before being digested overnight and extracted. The extracted peptides samples were then dissolved and incubated in UA buffer with DTT, iodoacetamide and trypsin at 37℃ for 16-18 h, after which 200 μg of the mixture was incorporated into 30μl SDT buffer (4% SDS, 100 mM DTT, 150 mM Tris-HCl pH 8.0) and washed using UA buffer (8 M Urea, 150 mM Tris-HCl pH 8.0) by repeated ultrafiltration (Microcon units, 10 kD). The protein suspensions were finally digested, filtrated, desalted and reconstituted in 40 µl of 0.1% (v/v) formic acid.

After estimating the peptide content, the fraction was injected for nanoLC-MS/MS analysis which was performed on a Q Exactive mass spectrometer (Thermo Scientific) coupled to Easy nLC (Thermo Fisher Scientific). MS/MS spectra were searched using MASCOT engine (Matrix Science, London, UK; version 2.2) against a nonredundant International Protein Index arabidopsis sequence database v3.85 (released at September 2011; 39679 sequences) from the European Bioinformatics Institute (http://www.ebi.ac.uk/).

Gel pieces cut from the SDS-PAGE were analysed for the containing proteins by ultra-performance liquid chromatography tandem mass spectrometry (UP LC-MS/MS) equipped with an Agilent Eclipse Plus C18 column(2.1 mm × 100 mm × 1.7 µm) and Sciex 6500 Q-TRAP mass spectrometer (Sciex, USA). AnalystTM 1.6.2 software (Applied Biosystems, California, USA) was applied to acquire the data and peptides were analyzed on a Q-Exactive Plus mass spectrometer (Thermo Fisher Scientific, San Jose, CA) equipped with an Easy-nLC 1000 (Thermo Fisher Scientific).

**Protein domain mapping**

CTGF cDNAs were subcloned into pT7CFE1-NFtag vectors using primers containing FLAG epitope tag sequences to generate CTGF-FLAG constructs. HEK293T cells were transfected by these plasmids (15 μg) mixed with polyethylenimine (PEI, 15 μg/ml). After 48-72 hours, transfected cells were lysed, and protein complexes were immunorecipitated using anti-FLAG or anti-ANXA2 affinity gel (Sigma). Complexes were then subjected to immunoblotting with the anti-FLAG Ab or anti-ANXA2 Ab.

**Quantitative polymerase chain reaction (QPCR) analysis**

Total RNA was extracted from synovial tissues of RA patients and normal subjects using Trizol reagent (Invitrogen) and reverse transcripted into cDNA using RT reagent Kit with gDNA Eraser (Takara, Japan) kit according to the manufacturer’s protocol. QPCR was performed with a total of 39 cycles consisting of 15 seconds at 95°C and 30 seconds at 60°C in CFX96 Touch Real-Time PCR Detection System (BioRad, USA) with SYBR®Premix Ex Taq^TM^ II (Takara, Japan). All reactions were conducted in triplicate and 2 ^–△△C t^ method was applied to calculate the relative expressions of target genes compared with that of β-actin. The primers of CTGF, ANXA2 and β-actin genes are listed in the supplementary Table 2.

**Enzyme-linked Immuneosorbent Assay (ELISA) of cytokines in serum**

Concentrations of cytokines (Biolegend, USA) including CTGF and ANXA2 in the serum of RA patients were determined using standard two-site sandwich ELISA kits according to the manufacturer’s protocols. A standard curve was established using standard concentrations and the results were analyzed in the Bio-Rad iMarkTM microplate reader.

**Immunohistochemistry**

Sections of synovial tissues from RA patients and normal subjects were deparaffinized and rehydrated, followed by the epitope recovery using sodium citrate buffer (pH=6.0) at 95℃ for 20 minutes. The slides were then treated with H₂O₂ against endogenous peroxidase and blocked with 5% BSA. Anti-CTGF antibody (ab6992, Abcam) or Anti-ANXA2 (ab41803, Abcam) antibody was incubated with the slides overnight at 4℃ followed by the corresponding secondary antibodies conjugated with HRP at 37℃ for an hour. DAB substrate was used for signal visualization and high-resolution images were captured using a Nikon photomicroscope.

**Immunofluorescence**

FLS from RA patients were cultured in normal slides until 70% ~ 80% confluence and then fixed with 4% paraformaldehyde (PFA). Sections of synovial tissues from RA patients were first performed to epitope recovery as mentioned in immunohistochenistry. Next, the slides were permeabilized in 0.25% Triton X-100, blocked with 5% BSA, incubated with primary antibodies of Anti-CTGF body (ab6992, Abcam) and Anti-ANXA2 antibody (ab41803, Abcam) overnight at 4℃, followed by secondary antibodies of Donkey anti-rabbit IgG DyLightTM594 conjugated and Donkey anti-mouse IgG(H+L) Alexa Fluor488 conjugated respectively at 37℃ for an hour. All the antibodies were diluted according to the manufacturer’s instructions. DAPI (Invitrogen) was used to stain the nuclei. The staining of FLS was visualized on a Nikon photomicroscope and the staining of synovial tissues was viewed by a confocal microscope (Zeiss LSM800, Germany).

**Western Blotting Analysis**

Synovial tissues of human were lysed with RIPA followed by the determination of the protein concentrations using Bradford Protein Assay according to the manufacturer’s instructions. The expressions of CTGF (ab6992, Abcam), ANXA2 (ab41803, Abcam), Flag (80010-1-RR, proteintech) were analyzed by western blotting. β-actin (sc-47778, santacruze) served as an internal control and the expression levels were analyzed using Image J.

**Homology modeling of TSP1 and construction of the ANXA2/TSP1 complex**

Homology modeling of the TSP1 domain of CTGF was performed using Modeller 9.25 program(1). The sequence of TSP1 domain was download from the UniProt (accession number: P29279). The crystal structure of TSP1-CCN3 (PDB ID: 6RK1) (2) was used as a model template. Before the construction of the homology model, sequence alignment of TSP1/TSP1-CCN3 was done. After modeling, ten conformations were obtained, and the conformations with the lowest DOPE score were selected for further study.

To obtain a reasonable ANXA2/TSP-1 complex, the protein-protein docking method was adopted to search for the binding conformation. The docking work was carried using ZDOCK 3.0.2 program (3). The acceptor ANXA2 (PDB ID: 2HYV) (4) was downloaded from the PDB database, and the docking ligand was the TSP1 structure obtained by the above homology modeling. When running the ZDOCK program, the output conformation is set to 2000, and the shape complementation, electrostatic potential, and energy between the acceptor protein and ligand protein were evaluated by ZDOCK scores. After docking, all out conformation of the output was clustered by “cluster.pl” script in MMTSB Tool Set (5). Preliminary, three clusters are determined by combining the ZDOCK score. In the end, the exact binding conformation was obtained according to Rosetta program optimate and molecular dynamics (MD) simulation.

**Dynamic simulation of ANXA2/TSP-1 complexes**

Three complex models were constructed above for MD simulation. Before the simulation, each system was prepared using the LEaP module of AMBER16 package (6). The AMBER force field ff14SB (7) was applied for describing accepter and ligand proteins. And then, each system was immersed into a rectangular periodic box of pre-equilibrated TIP3P water with at least 10 Å distance around the complexes. Finally, appropriate numbers of sodium counter ions were added to maintain the electroneutrality of the simulation system.

All MD simulations were performed using the pmemd module in AMBER16 package. For each simulation, a sophisticated protocol (minimization, heating, equilibration and production) was followed. Initially, water molecules were minimized through 2500 steps of steepest descent followed by 2500 steps of conjugate gradient while proteins were kept at the position except for the hydrogens. Then, the same minimization protocol was applied to optimize the side chains. Finally, the whole system was relaxed for 5000 steps without any restraints. After energy minimization, each system was gradually heated at constant volume from 0 K to 300 K over a coupling time of 100 ps with position restraints. To accommodate solvent density, the whole system was equilibrated over 100 ps at a constant pressure of 1 bar. Subsequently, another 100 ps pre-equilibration was performed for pressure relaxation with a weak restraint on the protein backbone. After that, 30 ns MD simulation was conducted for each system to produce trajectories. During MD simulations, periodic boundary conditions were employed and the direct space interaction was calculated by using the particle mesh Ewald (PME) method with a long-range electrostatic interaction (8). All bonds involving hydrogen atoms were constrained with the SHAKE algorithm (9) allowing an integration time step of 2 fs. MD simulations revealed that the complex model of cluster 1 was most stable, which was visually analyzed in results section.

**Reference**

1. Webb B, Sali A. Comparative Protein Structure Modeling Using MODELLER. Curr Protoc Protein Sci. 2016;**86**:2 9 1-2 9 37.

2. Xu ER, Lafita A, Bateman A, Hyvonen M. The thrombospondin module 1 domain of the matricellular protein CCN3 shows an atypical disulfide pattern and incomplete CWR layers. Acta Crystallogr D Struct Biol. 2020;**76**:124-34.

3. Pierce BG, Hourai Y, Weng Z. Accelerating protein docking in ZDOCK using an advanced 3D convolution library. PLoS One. 2011;**6**:e24657.

4. Shao C, Zhang F, Kemp MM, Linhardt RJ, Waisman DM, Head JF, et al. Crystallographic analysis of calcium-dependent heparin binding to annexin A2. J Biol Chem. 2006;**281**:31689-95.

5. Feig M, Karanicolas J, Brooks CL, 3rd. MMTSB Tool Set: enhanced sampling and multiscale modeling methods for applications in structural biology. J Mol Graph Model. 2004;**22**:377-95.

6. Bertini I, Case DA, Ferella L, Giachetti A, Rosato A. A Grid-enabled web portal for NMR structure refinement with AMBER. Bioinformatics. 2011;**27**:2384-90.

7. Maier JA, Martinez C, Kasavajhala K, Wickstrom L, Hauser KE, Simmerling C. ff14SB: Improving the Accuracy of Protein Side Chain and Backbone Parameters from ff99SB. J Chem Theory Comput. 2015;**11**:3696-713.

8. Sagui C, Darden TA. Molecular dynamics simulations of biomolecules: long-range electrostatic effects. Annu Rev Biophys Biomol Struct. 1999;**28**:155-79.

9. de Jong H. Modeling and simulation of genetic regulatory systems: a literature review. J Comput Biol. 2002;**9**:67-103.
