# Supplementary material for: The protein-protein interaction between connective tissue growth factor and annexin A2 is relevant to pannus formation in rheumatoid arthritis
Source: Arthritis Res Ther. 2021 Oct 26;23:266. doi: 10.1186/s13075-021-02656-y (PMC8547044; doi:10.1186/s13075-021-02656-y)
Supplement: Supplementary file 3 — Additional file 3: Table S1. Demographic and clinical characteristics of the patients with RA and healthy controls. Values are expressed median (minimum, maximum) unless state otherwise. Abbreviations: ACPA, anticitrullinated protein antibodies; CRP, C reactive protein; DAS-28, disease activity score 28 joints; ESR, erythrocyte sedimentation rate; RF, rheumatoid factor; NA, not applicable. [file 13075_2021_2656_MOESM3_ESM.docx]

|  | **Normal** | **Inactive RA (DAS28<3.2)** | **Active RA**  **(DAS28>3.2)** |
| --- | --- | --- | --- |
| **Number, n** | 50 | 45 | 47 |
| **Age(years)** | 52[22, 85] | 55[39, 80] | 57[23, 79] |
| **Male/Female, n** | 21/29 | 5/40 | 10/37 |
| **CRP(mg/L)** | NA | 2.45[0.2, 35.2] | 40[4.58, 130] |
| **DAS28**  **score** | NA | 2.74[2.04, 2.91] | 4.52[3.59, 8.56] |
| **ESR(mm/h)** | NA | 35.7[10, 118] | 54[7, 100] |
| **RF positive, n(%)** | NA | 29(64) | 35(74) |
| **ACPA positive, n(%)** | NA | 35(77) | 41(88) |
| **Duration of disease(years)** | NA | 7[0, 30] | 10[0, 44] |

**Table S1. Demographic and clinical characteristics of the patients with RA and healthy controls**

Values are expressed median (minimum, maximum) unless state otherwise

Abbreviations: ACPA, anticitrullinated protein antibodies; CRP, C reactive protein; DAS-28, disease activity score 28 joints; ESR, erythrocyte sedimentation rate; RF, rheumatoid factor; NA, not applicable.
