# Supplementary material for: The protein-protein interaction between connective tissue growth factor and annexin A2 is relevant to pannus formation in rheumatoid arthritis
Source: Arthritis Res Ther. 2021 Oct 26;23:266. doi: 10.1186/s13075-021-02656-y (PMC8547044; doi:10.1186/s13075-021-02656-y)
Supplement: Supplementary file 4 — Additional file 4: Table S2. Sequences of qRT-PCR primers, cDNA primers and shRNA-targeting genes. [file 13075_2021_2656_MOESM4_ESM.docx]

**Table S2.** Sequences of qRT-PCR primers, cDNA primers and shRNA-targeting genes.

| **qRT-PCR** | **Forward (5’→3’)** | **Reverse (5’→3’)** |
| --- | --- | --- |
| *CTGF* | GCGGCTTACCGACTGGA | ACAGGCGGCTCTGCTTC |
| *ANXA2* | TCGGACACATCTGGTGACTTCC | CCTCTTCACTCCAGCGTCATAG |
| *β-ACTIN* | ATGGTGGGTATGGGTCAGAAG | TGGCTGGGGTGTTGAAGGTC |

| **cDNA** | **Forward (5’→3’)** | **Reverse (5’→3’)** |
| --- | --- | --- |
| CTGF-IGFBP1-VWC | AAGCTGGCCACCACCCATATGATGACCGCCGCCAGTAT | TGAACCGCCACCACCGTTGGCTCTAATCATAGTTGGGT |
| CTGF-IGFBP1-VWC-TSP1 | AAGCTGGCCACCACCCATATGATGACCGCCGCCAGTAT | CTTAATTAAGATATCGAATTCTTCGCAAGGCCTGACCAT |
| CTGF-TSP1-CT | GGTGGTGGCGGTTCACCCAAGGACCAAAC | CTTAATTAAGATATCGAATTCTCATGCCATGTCTCCGTAC |
| CTGF-VWC-TSP1-CT | AAGCTGGCCACCACCCATATGGCCAACCGCAAGAT | CTTAATTAAGATATCGAATTCTCATGCCATGTCTCCGTAC |
| CTGF-CT | GGTGGTGGCGGTTCA AACATTAAGAAGGGCAA | CTTAATTAAGATATCGAATTCTCATGCCATGTCTCCGTAC |
| CTGF-IGFBP1 | AAGCTGGCCACCACCCATATGATGACCGCCGCCAGTAT | TGAACCGCCACCACCGGAGCCGAAGTCACAG |
| VC155-CTGF-ΔTSP1 | GGTGGTGGCGGTTCA AACATTAAGAAGGGCAA | TGAACCGCCACCACCGTTGGCTCTAATCATAGTTGGGT |
| VC155-CTGF | AGATTACGCTCTTATGGCCATGGAGGCCATGACCGCCG | TTTGCACGCCGGACGGGTACC TGCCATGTCTCC |
| VN152L-ANXA2 | ACCGAGATCTCTCGAGGTACCATGTCTACTGTTCACGAAATCC | GCTCCCGCCACCTCC GGTACC GTCATCTCC |

| **shRNA** | **Target gene sequence (5’→3’)** |
| --- | --- |
| ANXA2 shRNA1 | ACTTTGATGCTGAGCGGGA |
| ANXA2 shRNA2 | TGTGAAAGCCTATACTAAC |
| ANXA2 shRNA3 | GGAGTGAAGAGGAAAGGAACT |

| **shRNA** | **Target gene sequence (5’→3’)** |
| --- | --- |
| CTGF shRNA1 | CTATGATTAGAGCCAACTG |
| CTGF shRNA2 | GCTGACCTGGAAGAGAACA |
| CTGF shRNA3 | GCTAAATTCTGTGGAGTATGT |
