# Supplementary material for: The protein-protein interaction between connective tissue growth factor and annexin A2 is relevant to pannus formation in rheumatoid arthritis
Source: Arthritis Res Ther. 2021 Oct 26;23:266. doi: 10.1186/s13075-021-02656-y (PMC8547044; doi:10.1186/s13075-021-02656-y)
Supplement: Supplementary file 5 — Additional file 5: Table S3. LC-MS/MS identification of proteins isolated from FLS lysates of RA patients by immunoprecipitation using Anti-CTGF antibody. [file 13075_2021_2656_MOESM5_ESM.docx]

**Table S3.** LC-MS/MS identification of proteins isolated from FLS lysates of RA patients by immunoprecipitation using Anti-CTGF antibody.

|  | **Reference** | **PepCount** | **UniquePepCount** | **CoverPercent** | **MW** | **PI** | **IdentifiedName** |  |  |  |  |  |  |  |  |
| --- | --- | --- | --- | --- | --- | --- | --- | --- | --- | --- | --- | --- | --- | --- | --- |
|  | **File, Scan(s)** | **Sequence** | **MH+** | **Diff(MH+)** | **Charge** | **Rank** | **XC** | **DeltaCn** | **Sp** | **RSp** | **Ions** | **PI** | **GroupCount** | **ProteinCount** | |
| $1-1 |  | 44 | 22 | 46.96% | 69366.9 | 5.9 | sp\|P02768\|ALBU_HUMAN Serum albumin OS=Homo sapiens GN=ALB PE=1 SV=2 |  |  |  |  |  |  |  |  |
| $1-2 |  | 44 | 22 | 47.04% | 69267.8 | 5.9 | tr\|A8K9P0\|A8K9P0_HUMAN cDNA FLJ78413, highly similar to Homo sapiens albumin, mRNA OS=Homo sapiens PE=2 SV=1 |  |  |  |  |  |  |  |  |
|  | R16062_1_CTGF,6712 | K.AAFTECCQAADK.A | 1372.4502 | 0.3522 | 2 | 1 | 2.2302 | 0.31 | 857.6 | 1 | 15\|22 | 4.37 | 1 | 2 |  |
|  | R16062_1_CTGF,7119 | K.AEFAEVSK.L | 880.9651 | 0.9051 | 2 | 1 | 2.3288 | 0.2313 | 570.3 | 1 | 12\|14 | 4.53 | 1 | 2 |  |
|  | R16062_1_CTGF,20631 | K.ALVLIAFAQYLQQCPFEDHVK.L | 2491.8625 | 1.8115 | 3 | 1 | 4.0949 | 0.5029 | 1066.4 | 1 | 30\|80 | 5.32 | 1 | 2 |  |
|  | R16062_1_CTGF,20656 | K.ALVLIAFAQYLQQCPFEDHVK.L | 2491.8625 | 2.9555 | 3 | 1 | 4.7639 | 0.6077 | 1238.6 | 1 | 34\|80 | 5.32 | 1 | 2 |  |
|  | R16062_1_CTGF,14260 | K.AVM*DDFAAFVEK.C | 1359.5296 | -1.6974 | 2 | 1 | 3.1497 | 0.3656 | 915 | 1 | 16\|22 | 4.03 | 1 | 2 |  |
|  | R16062_1_CTGF,14313 | K.AVM*DDFAAFVEK.C | 1359.5296 | 0.1726 | 2 | 1 | 2.5017 | 0.3457 | 706.2 | 1 | 17\|22 | 4.03 | 1 | 2 |  |
|  | R16062_1_CTGF,14331 | K.AVM*DDFAAFVEK.C | 1359.5296 | -0.5934 | 2 | 1 | 2.3731 | 0.3825 | 848.4 | 1 | 17\|22 | 4.03 | 1 | 2 |  |
|  | R16062_1_CTGF,14358 | K.AVM*DDFAAFVEK.C | 1359.5296 | 1.0996 | 2 | 1 | 2.2867 | 0.3166 | 846.6 | 1 | 17\|22 | 4.03 | 1 | 2 |  |
|  | R16062_1_CTGF,6824 | K.CCTESLVNR.R | 1139.229 | -0.602 | 2 | 1 | 2.2551 | 0.4735 | 1007 | 1 | 12\|16 | 5.99 | 1 | 2 |  |
|  | R16062_1_CTGF,6877 | K.CCTESLVNR.R | 1139.229 | 1.163 | 2 | 1 | 2.3676 | 0.4258 | 954.1 | 1 | 14\|16 | 5.99 | 1 | 2 |  |
|  | R16062_1_CTGF,6242 | K.DDNPNLPR.L | 940.9806 | 0.7426 | 2 | 1 | 2.2339 | 0.2219 | 874 | 1 | 11\|14 | 4.21 | 1 | 2 |  |
|  | R16062_1_CTGF,19484 | K.DVFLGM*FLYEYAR.R | 1640.8834 | 0.8524 | 2 | 1 | 3.0139 | 0.4881 | 997.6 | 1 | 17\|24 | 4.37 | 1 | 2 |  |
|  | R16062_1_CTGF,19496 | K.DVFLGM*FLYEYAR.R | 1640.8834 | -0.6686 | 2 | 1 | 3.423 | 0.4331 | 1324.6 | 1 | 18\|24 | 4.37 | 1 | 2 |  |
|  | R16062_1_CTGF,19505 | K.DVFLGM*FLYEYAR.R | 1640.8834 | 0.5964 | 2 | 1 | 2.6589 | 0.4979 | 996.8 | 1 | 17\|24 | 4.37 | 1 | 2 |  |
|  | R16062_1_CTGF,19516 | K.DVFLGM*FLYEYAR.R | 1640.8834 | 0.7984 | 2 | 1 | 2.5715 | 0.3833 | 986.3 | 1 | 17\|24 | 4.37 | 1 | 2 |  |
|  | R16062_1_CTGF,19785 | K.DVFLGM*FLYEYAR.R | 1640.8834 | 1.8794 | 2 | 1 | 2.6796 | 0.4265 | 845.7 | 1 | 15\|24 | 4.37 | 1 | 2 |  |
|  | R16062_1_CTGF,11191 | K.FQNALLVR.Y | 961.1429 | 0.5669 | 2 | 1 | 2.4422 | 0.2544 | 1058.4 | 1 | 12\|14 | 9.75 | 1 | 2 |  |
|  | R16062_1_CTGF,10335 | K.KQTALVELVK.H | 1129.3751 | 2.2211 | 2 | 1 | 2.4736 | 0.2965 | 1316.6 | 1 | 15\|18 | 8.59 | 1 | 2 |  |
|  | R16062_1_CTGF,11134 | K.KVPQVSTPTLVEVSR.N | 1640.9062 | 0.5442 | 2 | 1 | 2.4474 | 0.5398 | 315.9 | 1 | 15\|28 | 8.75 | 1 | 2 |  |
|  | R16062_1_CTGF,11157 | K.KVPQVSTPTLVEVSR.N | 1640.9062 | 0.6052 | 2 | 1 | 2.3243 | 0.4234 | 336.6 | 1 | 16\|28 | 8.75 | 1 | 2 |  |
|  | R16062_1_CTGF,13412 | K.LVAASQAALGL.- | 1014.2009 | 0.6839 | 2 | 1 | 2.7882 | 0.5005 | 996.1 | 1 | 16\|20 | 5.53 | 1 | 2 |  |
|  | R16062_1_CTGF,13422 | K.LVAASQAALGL.- | 1014.2009 | 0.2749 | 2 | 1 | 3.0405 | 0.3819 | 1232 | 1 | 16\|20 | 5.53 | 1 | 2 |  |
|  | R16062_1_CTGF,11182 | K.LVNEVTEFAK.T | 1150.3065 | 0.8625 | 1 | 1 | 2.0039 | 0.3011 | 690.1 | 1 | 11\|18 | 4.53 | 1 | 2 |  |
|  | R16062_1_CTGF,12316 | K.PLVEEPQNLIK.Q | 1280.4953 | 0.0293 | 2 | 1 | 2.2083 | 0.3007 | 880.9 | 1 | 13\|20 | 4.53 | 1 | 2 |  |
|  | R16062_1_CTGF,14567 | K.QNCELFEQLGEYK.F | 1658.784 | -0.011 | 2 | 1 | 2.4323 | 0.3734 | 1545.4 | 1 | 19\|24 | 4.25 | 1 | 2 |  |
|  | R16062_1_CTGF,14577 | K.QNCELFEQLGEYK.F | 1658.784 | 0.449 | 2 | 1 | 2.7525 | 0.378 | 1150.6 | 1 | 17\|24 | 4.25 | 1 | 2 |  |
|  | R16062_1_CTGF,17211 | K.SHCIAEVENDEM*PADLPSLAADFVESK.D | 2992.2119 | 2.8169 | 3 | 1 | 4.1521 | 0.521 | 1125.5 | 1 | 36\|104 | 3.95 | 1 | 2 |  |
|  | R16062_1_CTGF,17327 | K.SHCIAEVENDEM*PADLPSLAADFVESK.D | 2992.2119 | -2.1631 | 3 | 1 | 4.2316 | 0.3745 | 844.7 | 1 | 33\|104 | 3.95 | 1 | 2 |  |
|  | R16062_1_CTGF,16095 | K.VFDEFKPLVEEPQNLIK.Q | 2046.3515 | 0.8305 | 2 | 1 | 2.8547 | 0.4511 | 555.1 | 1 | 17\|32 | 4.41 | 1 | 2 |  |
|  | R16062_1_CTGF,16106 | K.VFDEFKPLVEEPQNLIK.Q | 2046.3515 | 1.3165 | 2 | 1 | 3.1597 | 0.4164 | 681.8 | 1 | 19\|32 | 4.41 | 1 | 2 |  |
|  | R16062_1_CTGF,16197 | K.VFDEFKPLVEEPQNLIK.Q | 2046.3515 | -2.1115 | 2 | 1 | 3.3842 | 0.4492 | 562 | 1 | 16\|32 | 4.41 | 1 | 2 |  |
|  | R16062_1_CTGF,8296 | K.VHTECCHGDLLECADDR.A | 2088.1594 | -0.1966 | 3 | 1 | 4.1199 | 0.5698 | 1059 | 1 | 32\|64 | 4.42 | 1 | 2 |  |
|  | R16062_1_CTGF,8319 | K.VHTECCHGDLLECADDR.A | 2088.1594 | 1.3534 | 3 | 1 | 3.9577 | 0.4998 | 1258.5 | 1 | 31\|64 | 4.42 | 1 | 2 |  |
|  | R16062_1_CTGF,6609 | K.YICENQDSISSK.L | 1444.5197 | 0.1597 | 2 | 1 | 2.2788 | 0.4401 | 611.4 | 1 | 14\|22 | 4.37 | 1 | 2 |  |
|  | R16062_1_CTGF,6957 | K.YICENQDSISSK.L | 1444.5197 | 1.8347 | 2 | 1 | 2.6659 | 0.4261 | 1162.5 | 1 | 18\|22 | 4.37 | 1 | 2 |  |
|  | R16062_1_CTGF,6979 | K.YICENQDSISSK.L | 1444.5197 | 1.8547 | 2 | 1 | 2.8368 | 0.4045 | 1259.3 | 1 | 19\|22 | 4.37 | 1 | 2 |  |
|  | R16062_1_CTGF,7032 | K.YICENQDSISSK.L | 1444.5197 | -1.5123 | 2 | 1 | 3.3577 | 0.4001 | 1090.8 | 1 | 17\|22 | 4.37 | 1 | 2 |  |
|  | R16062_1_CTGF,15144 | R.HPDYSVVLLLR.L | 1312.5422 | 1.1952 | 2 | 1 | 2.5133 | 0.2711 | 984.1 | 1 | 14\|20 | 6.74 | 1 | 2 |  |
|  | R16062_1_CTGF,20221 | R.M*PCAEDYLSVVLNQLCVLHEK.T | 2535.8857 | -2.6683 | 3 | 1 | 5.1351 | 0.4736 | 1705.4 | 1 | 33\|80 | 4.65 | 1 | 2 |  |
|  | R16062_1_CTGF,20242 | R.M*PCAEDYLSVVLNQLCVLHEK.T | 2535.8857 | 1.0147 | 3 | 1 | 3.7864 | 0.3995 | 936.6 | 1 | 26\|80 | 4.65 | 1 | 2 |  |
|  | R16062_1_CTGF,20515 | R.MPCAEDYLSVVLNQLCVLHEK.T | 2519.8863 | 1.1873 | 3 | 1 | 3.9377 | 0.4521 | 1246.3 | 1 | 28\|80 | 4.65 | 1 | 2 |  |
|  | R16062_1_CTGF,20542 | R.MPCAEDYLSVVLNQLCVLHEK.T | 2519.8863 | 1.1923 | 3 | 1 | 4.2464 | 0.4069 | 1660.3 | 1 | 32\|80 | 4.65 | 1 | 2 |  |
|  | R16062_1_CTGF,18552 | R.RHPYFYAPELLFFAK.R | 1900.2131 | 0.7511 | 3 | 1 | 3.8122 | 0.4187 | 1094.5 | 1 | 26\|56 | 8.5 | 1 | 2 |  |
|  | R16062_1_CTGF,13293 | R.RPCFSALEVDETYVPK.E | 1912.1259 | 0.9789 | 2 | 1 | 2.2234 | 0.406 | 807.5 | 1 | 17\|30 | 4.68 | 1 | 2 |  |
| $2-1 |  | 39 | 20 | 9.16% | 343670 | 6.3 | sp\|P12111\|CO6A3_HUMAN Collagen alpha-3(VI) chain OS=Homo sapiens GN=COL6A3 PE=1 SV=5 |  |  |  |  |  |  |  |  |
| $2-2 |  | 39 | 20 | 11.33% | 278119 | 8.4 | tr\|E7ENL6\|E7ENL6_HUMAN Collagen alpha-3(VI) chain OS=Homo sapiens GN=COL6A3 PE=1 SV=2 |  |  |  |  |  |  |  |  |
| $2-3 |  | 39 | 20 | 9.16% | 343670 | 6.3 | tr\|D9ZGF2\|D9ZGF2_HUMAN Collagen, type VI, alpha 3 OS=Homo sapiens GN=COL6A3 PE=4 SV=1 |  |  |  |  |  |  |  |  |
| $2-4 |  | 39 | 20 | 11.32% | 278212 | 8.4 | tr\|B7ZW00\|B7ZW00_HUMAN COL6A3 protein OS=Homo sapiens GN=COL6A3 PE=2 SV=1 |  |  |  |  |  |  |  |  |
|  | R16062_1_CTGF,14932 | K.ALNLGYALDYAQR.Y | 1468.639 | -0.959 | 2 | 1 | 2.9211 | 0.3707 | 779.1 | 1 | 14\|24 | 5.88 | 1 | 4 |  |
|  | R16062_1_CTGF,14944 | K.ALNLGYALDYAQR.Y | 1468.639 | 0.803 | 2 | 1 | 2.7589 | 0.5117 | 1025.1 | 1 | 15\|24 | 5.88 | 1 | 4 |  |
|  | R16062_1_CTGF,14956 | K.ALNLGYALDYAQR.Y | 1468.639 | 0.41 | 2 | 1 | 3.1235 | 0.5698 | 1599 | 1 | 17\|24 | 5.88 | 1 | 4 |  |
|  | R16062_1_CTGF,5703 | K.DEVQNAVQR.L | 1059.1155 | 1.2475 | 2 | 1 | 2.3302 | 0.2552 | 1188 | 1 | 14\|16 | 4.37 | 1 | 4 |  |
|  | R16062_1_CTGF,21710 | K.GADQAELEEIAFDSSLVFIPAEFR.A | 2655.8968 | 0.5508 | 2 | 1 | 3.6562 | 0.5248 | 820.3 | 1 | 21\|46 | 3.77 | 1 | 4 |  |
|  | R16062_1_CTGF,9630 | K.IIDELNVKPEGTR.I | 1484.6799 | 0.5559 | 2 | 1 | 2.5888 | 0.2852 | 690.1 | 1 | 15\|24 | 4.68 | 1 | 4 |  |
|  | R16062_1_CTGF,9640 | K.IIDELNVKPEGTR.I | 1484.6799 | 0.3459 | 2 | 1 | 2.3239 | 0.2159 | 327.8 | 8 | 10\|24 | 4.68 | 1 | 4 |  |
|  | R16062_1_CTGF,13068 | K.LLVLITGGK.S | 914.1676 | 0.6086 | 2 | 1 | 2.4591 | 0.359 | 452 | 3 | 11\|16 | 8.75 | 1 | 4 |  |
|  | R16062_1_CTGF,13076 | K.LLVLITGGK.S | 914.1676 | -0.8794 | 2 | 1 | 2.5384 | 0.3139 | 436.9 | 1 | 12\|16 | 8.75 | 1 | 4 |  |
|  | R16062_1_CTGF,13082 | K.LLVLITGGK.S | 914.1676 | 0.1936 | 2 | 1 | 2.2937 | 0.2751 | 588.4 | 1 | 12\|16 | 8.75 | 1 | 4 |  |
|  | R16062_1_CTGF,13093 | K.LLVLITGGK.S | 914.1676 | 0.5676 | 2 | 1 | 2.2517 | 0.3566 | 526.6 | 1 | 12\|16 | 8.75 | 1 | 4 |  |
|  | R16062_1_CTGF,22157 | K.NADPAELEQIVLSPAFILAAESLPK.I | 2638.0092 | 1.1642 | 2 | 1 | 2.7308 | 0.4399 | 434.1 | 1 | 18\|48 | 4 | 1 | 4 |  |
|  | R16062_1_CTGF,10602 | K.SLDEISQPAQELK.R | 1458.5961 | 0.5181 | 2 | 1 | 2.9548 | 0.2403 | 809.6 | 1 | 17\|24 | 4.14 | 1 | 4 |  |
|  | R16062_1_CTGF,16863 - 16865 | R.AAPLQGM*LPGLLAPLR.T | 1635.0114 | 0.9864 | 2 | 1 | 2.5149 | 0.406 | 276.7 | 1 | 16\|30 | 9.8 | 1 | 4 |  |
|  | R16062_1_CTGF,16932 | R.AAPLQGM*LPGLLAPLR.T | 1635.0114 | 0.4984 | 3 | 1 | 3.8169 | 0.4834 | 1208.6 | 1 | 26\|60 | 9.8 | 1 | 4 |  |
|  | R16062_1_CTGF,16968 | R.AAPLQGM*LPGLLAPLR.T | 1635.0114 | 2.8054 | 2 | 1 | 2.2802 | 0.4332 | 312.7 | 1 | 15\|30 | 9.8 | 1 | 4 |  |
|  | R16062_1_CTGF,17156 - 17157 | R.DVVFLIDGSQSAGPEFQYVR.T | 2228.4459 | 1.0499 | 2 | 1 | 4.0696 | 0.6114 | 1259.3 | 1 | 22\|38 | 4.03 | 1 | 4 |  |
|  | R16062_1_CTGF,17189 | R.DVVFLIDGSQSAGPEFQYVR.T | 2228.4459 | 0.3179 | 2 | 1 | 3.1915 | 0.5147 | 906.4 | 1 | 19\|38 | 4.03 | 1 | 4 |  |
|  | R16062_1_CTGF,9056 | R.IAVAQYSDDVK.V | 1209.3308 | 0.6418 | 2 | 1 | 2.3626 | 0.5204 | 917.4 | 1 | 14\|20 | 4.21 | 1 | 4 |  |
|  | R16062_1_CTGF,9067 | R.IAVAQYSDDVK.V | 1209.3308 | 0.1308 | 2 | 1 | 2.5511 | 0.4081 | 1994.7 | 1 | 18\|20 | 4.21 | 1 | 4 |  |
|  | R16062_1_CTGF,9117 | R.IAVAQYSDDVK.V | 1209.3308 | 2.0528 | 2 | 1 | 2.4714 | 0.4038 | 1278.7 | 1 | 17\|20 | 4.21 | 1 | 4 |  |
|  | R16062_1_CTGF,9121 | R.IAVAQYSDDVK.V | 1209.3308 | 1.2068 | 2 | 1 | 2.4857 | 0.2687 | 1306.6 | 1 | 16\|20 | 4.21 | 1 | 4 |  |
|  | R16062_1_CTGF,19777 | R.IEEGVPQFLVLISSGK.S | 1716.9994 | -0.4676 | 3 | 1 | 3.8563 | 0.4953 | 1431.1 | 1 | 27\|60 | 4.53 | 1 | 4 |  |
|  | R16062_1_CTGF,19970 | R.ITEGVPQLLIVLTADR.S | 1739.0498 | -0.5362 | 2 | 1 | 3.2758 | 0.4524 | 292.2 | 1 | 15\|30 | 4.37 | 1 | 4 |  |
|  | R16062_1_CTGF,19980 | R.ITEGVPQLLIVLTADR.S | 1739.0498 | -0.6452 | 2 | 1 | 3.0543 | 0.4959 | 225.2 | 1 | 14\|30 | 4.37 | 1 | 4 |  |
|  | R16062_1_CTGF,12605 | R.LQPVLQPLPSPGVGGK.R | 1587.8881 | 1.0791 | 2 | 1 | 2.4903 | 0.4159 | 429.1 | 1 | 17\|30 | 8.75 | 1 | 4 |  |
|  | R16062_1_CTGF,15766 | R.LVDYLDVGFDTTR.V | 1514.6613 | 0.4913 | 2 | 1 | 3.1491 | 0.4611 | 1070.6 | 1 | 16\|24 | 3.93 | 1 | 4 |  |
|  | R16062_1_CTGF,15809 | R.LVDYLDVGFDTTR.V | 1514.6613 | -0.3497 | 2 | 1 | 2.5764 | 0.2266 | 967.4 | 1 | 16\|24 | 3.93 | 1 | 4 |  |
|  | R16062_1_CTGF,15822 | R.LVDYLDVGFDTTR.V | 1514.6613 | 0.5053 | 2 | 1 | 2.3081 | 0.2377 | 1545.7 | 1 | 17\|24 | 3.93 | 1 | 4 |  |
|  | R16062_1_CTGF,10731 | R.NNLFTSSAGYR.A | 1230.3118 | -0.7412 | 2 | 1 | 2.223 | 0.2646 | 499.5 | 3 | 11\|20 | 8.75 | 1 | 4 |  |
|  | R16062_1_CTGF,10741 | R.NNLFTSSAGYR.A | 1230.3118 | 0.9238 | 2 | 1 | 2.3389 | 0.2708 | 770.5 | 1 | 12\|20 | 8.75 | 1 | 4 |  |
|  | R16062_1_CTGF,14363 | R.QINVGNALEYVSR.N | 1463.6208 | 0.6018 | 2 | 1 | 2.2015 | 0.2728 | 515.8 | 2 | 13\|24 | 6 | 1 | 4 |  |
|  | R16062_1_CTGF,14376 | R.QINVGNALEYVSR.N | 1463.6208 | 0.5608 | 2 | 1 | 2.3592 | 0.3664 | 490.8 | 1 | 13\|24 | 6 | 1 | 4 |  |
|  | R16062_1_CTGF,11488 | R.QLGTVQQVISER.V | 1358.5261 | -0.2559 | 2 | 1 | 2.5448 | 0.3233 | 704.2 | 2 | 13\|22 | 6 | 1 | 4 |  |
|  | R16062_1_CTGF,11602 | R.QLGTVQQVISER.V | 1358.5261 | 1.7751 | 2 | 1 | 2.3307 | 0.2585 | 857.5 | 1 | 13\|22 | 6 | 1 | 4 |  |
|  | R16062_1_CTGF,19457 | R.QLTLLGGPTPNTGAALEFVLR.N | 2169.5094 | 0.9644 | 2 | 1 | 3.1474 | 0.5236 | 626.8 | 1 | 16\|40 | 6 | 1 | 4 |  |
|  | R16062_1_CTGF,7372 | R.VAVVQYSDR.T | 1037.1514 | 1.1024 | 2 | 1 | 2.514 | 0.3864 | 810.4 | 1 | 13\|16 | 5.81 | 1 | 4 |  |
|  | R16062_1_CTGF,8663 | R.VVESLDVGQDR.V | 1217.3113 | 1.2343 | 2 | 1 | 2.6315 | 0.4852 | 1195.8 | 1 | 16\|20 | 4.03 | 1 | 4 |  |
|  | R16062_1_CTGF,8674 | R.VVESLDVGQDR.V | 1217.3113 | 0.3873 | 2 | 1 | 2.7241 | 0.1637 | 875.3 | 1 | 14\|20 | 4.03 | 1 | 4 |  |
| $3-1 |  | 33 | 13 | 44.54% | 38604.1 | 7.6 | sp\|P07355\|ANXA2_HUMAN Annexin A2 OS=Homo sapiens GN=ANXA2 PE=1 SV=2 |  |  |  |  |  |  |  |  |
| $3-2 |  | 33 | 13 | 44.54% | 38604.1 | 7.6 | tr\|A0A024R5Z7\|A0A024R5Z7_HUMAN Annexin OS=Homo sapiens GN=ANXA2 PE=3 SV=1 |  |  |  |  |  |  |  |  |
| $3-3 |  | 33 | 13 | 44.54% | 38576 | 7.6 | tr\|V9HW65\|V9HW65_HUMAN Annexin OS=Homo sapiens GN=HEL-S-270 PE=2 SV=1 |  |  |  |  |  |  |  |  |
|  | R16062_1_CTGF,11939 | K.DIISDTSGDFR.K | 1226.275 | -0.798 | 2 | 1 | 2.745 | 0.3833 | 806.5 | 1 | 13\|20 | 3.93 | 1 | 3 |  |
|  | R16062_1_CTGF,10567 | K.DIISDTSGDFRK.L | 1354.448 | 0.458 | 2 | 1 | 2.657 | 0.3846 | 946.8 | 1 | 14\|22 | 4.43 | 1 | 3 |  |
|  | R16062_1_CTGF,21276 | K.GDLENAFLNLVQCIQNKPLYFADR.L | 2840.1763 | 1.3103 | 3 | 1 | 4.0045 | 0.4164 | 433.2 | 1 | 24\|92 | 4.56 | 1 | 3 |  |
|  | R16062_1_CTGF,17836 | K.GLGTDEDSLIEIICSR.T | 1778.9331 | -1.7609 | 2 | 1 | 2.8455 | 0.3208 | 575 | 1 | 15\|30 | 3.92 | 1 | 3 |  |
|  | R16062_1_CTGF,17943 | K.GLGTDEDSLIEIICSR.T | 1778.9331 | 1.1261 | 2 | 1 | 3.1765 | 0.4751 | 1288.4 | 1 | 17\|30 | 3.92 | 1 | 3 |  |
|  | R16062_1_CTGF,17954 | K.GLGTDEDSLIEIICSR.T | 1778.9331 | 0.5671 | 2 | 1 | 2.7418 | 0.4419 | 1399.6 | 1 | 19\|30 | 3.92 | 1 | 3 |  |
|  | R16062_1_CTGF,17817 | K.GVDEVTIVNILTNR.S | 1543.7474 | 0.5054 | 2 | 1 | 3.1575 | 0.4212 | 698.8 | 2 | 14\|26 | 4.37 | 1 | 3 |  |
|  | R16062_1_CTGF,17866 | K.GVDEVTIVNILTNR.S | 1543.7474 | 0.4614 | 3 | 1 | 4.3006 | 0.4971 | 1377.3 | 1 | 26\|52 | 4.37 | 1 | 3 |  |
|  | R16062_1_CTGF,17888 | K.GVDEVTIVNILTNR.S | 1543.7474 | 0.1034 | 3 | 1 | 4.4787 | 0.4918 | 1834.1 | 1 | 30\|52 | 4.37 | 1 | 3 |  |
|  | R16062_1_CTGF,17925 | K.GVDEVTIVNILTNR.S | 1543.7474 | 1.7304 | 2 | 1 | 3.2994 | 0.4666 | 1053.9 | 1 | 18\|26 | 4.37 | 1 | 3 |  |
|  | R16062_1_CTGF,17991 | K.GVDEVTIVNILTNR.S | 1543.7474 | 1.8024 | 2 | 1 | 2.7142 | 0.4139 | 1104 | 1 | 17\|26 | 4.37 | 1 | 3 |  |
|  | R16062_1_CTGF,20154 | K.SALSGHLETVILGLLK.T | 1651.9721 | -0.4599 | 2 | 1 | 3.1199 | 0.4376 | 1925.4 | 1 | 22\|30 | 6.47 | 1 | 3 |  |
|  | R16062_1_CTGF,20172 | K.SALSGHLETVILGLLK.T | 1651.9721 | -1.4309 | 2 | 1 | 3.3366 | 0.471 | 961 | 1 | 18\|30 | 6.47 | 1 | 3 |  |
|  | R16062_1_CTGF,20178 | K.SALSGHLETVILGLLK.T | 1651.9721 | 0.4791 | 2 | 1 | 3.5 | 0.4777 | 1065.6 | 1 | 17\|30 | 6.47 | 1 | 3 |  |
|  | R16062_1_CTGF,20187 | K.SALSGHLETVILGLLK.T | 1651.9721 | 0.2391 | 2 | 1 | 3.7946 | 0.5534 | 2084.7 | 1 | 20\|30 | 6.47 | 1 | 3 |  |
|  | R16062_1_CTGF,20611 | K.SALSGHLETVILGLLK.T | 1651.9721 | 0.6901 | 2 | 1 | 3.1885 | 0.4247 | 926.7 | 1 | 16\|30 | 6.47 | 1 | 3 |  |
|  | R16062_1_CTGF,12857 | K.SLYYYIQQDTK.G | 1422.5639 | -1.4381 | 2 | 1 | 2.5631 | 0.3952 | 1209.4 | 1 | 15\|20 | 5.55 | 1 | 3 |  |
|  | R16062_1_CTGF,12870 | K.SLYYYIQQDTK.G | 1422.5639 | -1.8201 | 2 | 1 | 2.779 | 0.3789 | 834.5 | 1 | 13\|20 | 5.55 | 1 | 3 |  |
|  | R16062_1_CTGF,12891 | K.SLYYYIQQDTK.G | 1422.5639 | -0.3781 | 2 | 1 | 2.7818 | 0.521 | 1358.4 | 1 | 15\|20 | 5.55 | 1 | 3 |  |
|  | R16062_1_CTGF,12894 | K.SLYYYIQQDTK.G | 1422.5639 | -0.5101 | 2 | 1 | 2.8257 | 0.4721 | 1353.5 | 1 | 16\|20 | 5.55 | 1 | 3 |  |
|  | R16062_1_CTGF,7923 | K.TPAQYDASELK.A | 1223.3143 | 2.1903 | 2 | 1 | 2.4508 | 0.4565 | 802.1 | 1 | 16\|20 | 4.37 | 1 | 3 |  |
|  | R16062_1_CTGF,13864 | R.AEDGSVIDYELIDQDAR.D | 1909.9855 | 0.5425 | 2 | 1 | 3.3511 | 0.5712 | 771.6 | 1 | 16\|32 | 3.66 | 1 | 3 |  |
|  | R16062_1_CTGF,13876 | R.AEDGSVIDYELIDQDAR.D | 1909.9855 | -0.5665 | 2 | 1 | 2.3994 | 0.3681 | 441.6 | 1 | 15\|32 | 3.66 | 1 | 3 |  |
|  | R16062_1_CTGF,13885 | R.AEDGSVIDYELIDQDAR.D | 1909.9855 | 0.6365 | 2 | 1 | 3.2051 | 0.4289 | 816.4 | 1 | 16\|32 | 3.66 | 1 | 3 |  |
|  | R16062_1_CTGF,13896 | R.AEDGSVIDYELIDQDAR.D | 1909.9855 | 0.7285 | 2 | 1 | 3.0526 | 0.46 | 994.2 | 1 | 19\|32 | 3.66 | 1 | 3 |  |
|  | R16062_1_CTGF,12212 | R.DALNIETAIK.T | 1088.2367 | -0.1143 | 2 | 1 | 2.6785 | 0.2927 | 1169.5 | 1 | 15\|18 | 4.37 | 1 | 3 |  |
|  | R16062_1_CTGF,12223 | R.DALNIETAIK.T | 1088.2367 | 0.2547 | 2 | 1 | 2.7894 | 0.3095 | 1061.8 | 1 | 14\|18 | 4.37 | 1 | 3 |  |
|  | R16062_1_CTGF,10486 | R.QDIAFAYQR.R | 1112.22 | 0.15 | 2 | 1 | 2.3234 | 0.3387 | 652 | 1 | 12\|16 | 5.84 | 1 | 3 |  |
|  | R16062_1_CTGF,13092 | R.RAEDGSVIDYELIDQDAR.D | 2066.1718 | 0.1168 | 3 | 1 | 3.8537 | 0.3502 | 1406.8 | 1 | 28\|68 | 3.96 | 1 | 3 |  |
|  | R16062_1_CTGF,7506 | R.TNQELQEINR.V | 1245.3247 | -0.0253 | 2 | 1 | 2.3417 | 0.2791 | 849.7 | 1 | 13\|18 | 4.53 | 1 | 3 |  |
|  | R16062_1_CTGF,7515 | R.TNQELQEINR.V | 1245.3247 | 0.0417 | 2 | 1 | 2.5617 | 0.2716 | 1195 | 1 | 15\|18 | 4.53 | 1 | 3 |  |
|  | R16062_1_CTGF,7538 | R.TNQELQEINR.V | 1245.3247 | 1.1037 | 2 | 1 | 3.289 | 0.3454 | 1256 | 1 | 14\|18 | 4.53 | 1 | 3 |  |
|  | R16062_1_CTGF,7645 | R.TNQELQEINR.V | 1245.3247 | 1.8557 | 2 | 1 | 2.6993 | 0.3036 | 1335.6 | 1 | 15\|18 | 4.53 | 1 | 3 |  |
| $4-1 |  | 20 | 13 | 24.07% | 77079.9 | 6.7 | tr\|Q53H26\|Q53H26_HUMAN Transferrin variant (Fragment) OS=Homo sapiens PE=2 SV=1 |  |  |  |  |  |  |  |  |
|  | R16062_1_CTGF,12829 | K.CSTSSLLEACTFR.R | 1532.6651 | 0.3711 | 2 | 1 | 2.4199 | 0.4228 | 740.2 | 1 | 14\|24 | 5.99 | 2 | 2 |  |
|  | R16062_1_CTGF,9270 | K.DGAGDVAFVK.H | 979.0688 | 1.0618 | 2 | 1 | 2.5037 | 0.22 | 1686 | 1 | 16\|18 | 4.21 | 2 | 2 |  |
|  | R16062_1_CTGF,9294 | K.DGAGDVAFVK.H | 979.0688 | 0.2138 | 2 | 1 | 2.5948 | 0.1317 | 1081.9 | 1 | 15\|18 | 4.21 | 2 | 2 |  |
|  | R16062_1_CTGF,7396 | K.DSAHGFLK.V | 874.9638 | 0.5768 | 2 | 1 | 2.3767 | 0.335 | 895.4 | 2 | 12\|14 | 6.74 | 2 | 2 |  |
|  | R16062_1_CTGF,20972 | K.EDLIWELLNQAQEHFGK.D | 2071.278 | 0.924 | 2 | 1 | 3.3545 | 0.4635 | 1565.5 | 1 | 20\|32 | 4.4 | 2 | 2 |  |
|  | R16062_1_CTGF,12124 | K.EFQLFSSPHGK.D | 1277.41 | 0.316 | 2 | 1 | 2.2311 | 0.3316 | 705.8 | 1 | 13\|20 | 6.85 | 2 | 2 |  |
|  | R16062_1_CTGF,10796 | K.HSTIFENLANK.A | 1274.4077 | -0.0783 | 2 | 1 | 2.2266 | 0.2986 | 811.1 | 2 | 13\|20 | 6.75 | 2 | 2 |  |
|  | R16062_1_CTGF,10827 | K.HSTIFENLANK.A | 1274.4077 | 0.5427 | 2 | 1 | 2.6875 | 0.3806 | 553.9 | 1 | 13\|20 | 6.75 | 2 | 2 |  |
|  | R16062_1_CTGF,9189 | K.IECVSAETTEDCIAK.I | 1726.8484 | -0.2326 | 2 | 1 | 2.4604 | 0.48 | 552.6 | 1 | 14\|28 | 4 | 2 | 2 |  |
|  | R16062_1_CTGF,9198 | K.IECVSAETTEDCIAK.I | 1726.8484 | 0.4554 | 2 | 1 | 2.6615 | 0.4161 | 843.7 | 1 | 17\|28 | 4 | 2 | 2 |  |
|  | R16062_1_CTGF,14307 | K.SDNCEDTPEAGYFAVAVVK.K | 2073.1962 | 0.7702 | 2 | 1 | 2.3021 | 0.3521 | 531.3 | 1 | 16\|36 | 3.92 | 2 | 2 |  |
|  | R16062_1_CTGF,14316 | K.SDNCEDTPEAGYFAVAVVK.K | 2073.1962 | 0.8532 | 2 | 1 | 2.4285 | 0.4684 | 258.6 | 1 | 16\|36 | 3.92 | 2 | 2 |  |
|  | R16062_1_CTGF,10111 | K.SVIPSDGPSVACVK.K | 1416.5958 | 0.9808 | 2 | 1 | 2.2711 | 0.4774 | 263.1 | 1 | 16\|26 | 5.55 | 2 | 2 |  |
|  | R16062_1_CTGF,13747 | R.DQYELLCLDNTR.K | 1540.6506 | 0.9546 | 2 | 1 | 2.4969 | 0.3701 | 1085.2 | 1 | 16\|22 | 4.03 | 2 | 2 |  |
|  | R16062_1_CTGF,13755 | R.DQYELLCLDNTR.K | 1540.6506 | 0.6456 | 2 | 1 | 3.3421 | 0.3648 | 1659 | 1 | 17\|22 | 4.03 | 2 | 2 |  |
|  | R16062_1_CTGF,12685 | R.FDEFFSEGCAPGSK.K | 1578.6542 | 0.6792 | 2 | 1 | 3.3023 | 0.5207 | 707.8 | 1 | 15\|26 | 4.14 | 2 | 2 |  |
|  | R16062_1_CTGF,19857 | R.SAGWNIPIGLLYCDLPEPR.K | 2172.4621 | 0.8271 | 2 | 1 | 3.2456 | 0.4379 | 1255 | 1 | 23\|36 | 4.37 | 1 | 1 |  |
|  | R16062_1_CTGF,19868 | R.SAGWNIPIGLLYCDLPEPR.K | 2172.4621 | 1.0421 | 2 | 1 | 2.6836 | 0.4098 | 801.6 | 1 | 21\|36 | 4.37 | 1 | 1 |  |
|  | R16062_1_CTGF,19978 | R.SAGWNIPIGLLYCDLPEPR.K | 2172.4621 | -2.0349 | 2 | 1 | 2.6939 | 0.3632 | 1189.2 | 1 | 19\|36 | 4.37 | 1 | 1 |  |
|  | R16062_1_CTGF,19663 | R.SM*GGKEDLIWELLNQAQEHFGK.D | 2547.8282 | -0.7488 | 3 | 1 | 4.2052 | 0.3899 | 1366.1 | 1 | 28\|84 | 4.83 | 2 | 2 |  |
| $5-1 |  | 18 | 13 | 23.75% | 74832.2 | 6.8 | tr\|B4E1B2\|B4E1B2_HUMAN cDNA FLJ53691, highly similar to Serotransferrin OS=Homo sapiens PE=2 SV=1 |  |  |  |  |  |  |  |  |
|  | R16062_1_CTGF,12829 | K.CSTSSLLEACTFR.R | 1532.6651 | 0.3711 | 2 | 1 | 2.4199 | 0.4228 | 740.2 | 1 | 14\|24 | 5.99 | 2 | 2 |  |
|  | R16062_1_CTGF,9270 | K.DGAGDVAFVK.H | 979.0688 | 1.0618 | 2 | 1 | 2.5037 | 0.22 | 1686 | 1 | 16\|18 | 4.21 | 2 | 2 |  |
|  | R16062_1_CTGF,9294 | K.DGAGDVAFVK.H | 979.0688 | 0.2138 | 2 | 1 | 2.5948 | 0.1317 | 1081.9 | 1 | 15\|18 | 4.21 | 2 | 2 |  |
|  | R16062_1_CTGF,7396 | K.DSAHGFLK.V | 874.9638 | 0.5768 | 2 | 1 | 2.3767 | 0.335 | 895.4 | 2 | 12\|14 | 6.74 | 2 | 2 |  |
|  | R16062_1_CTGF,20972 | K.EDLIWELLNQAQEHFGK.D | 2071.278 | 0.924 | 2 | 1 | 3.3545 | 0.4635 | 1565.5 | 1 | 20\|32 | 4.4 | 2 | 2 |  |
|  | R16062_1_CTGF,12124 | K.EFQLFSSPHGK.D | 1277.41 | 0.316 | 2 | 1 | 2.2311 | 0.3316 | 705.8 | 1 | 13\|20 | 6.85 | 2 | 2 |  |
|  | R16062_1_CTGF,10796 | K.HSTIFENLANK.A | 1274.4077 | -0.0783 | 2 | 1 | 2.2266 | 0.2986 | 811.1 | 2 | 13\|20 | 6.75 | 2 | 2 |  |
|  | R16062_1_CTGF,10827 | K.HSTIFENLANK.A | 1274.4077 | 0.5427 | 2 | 1 | 2.6875 | 0.3806 | 553.9 | 1 | 13\|20 | 6.75 | 2 | 2 |  |
|  | R16062_1_CTGF,9189 | K.IECVSAETTEDCIAK.I | 1726.8484 | -0.2326 | 2 | 1 | 2.4604 | 0.48 | 552.6 | 1 | 14\|28 | 4 | 2 | 2 |  |
|  | R16062_1_CTGF,9198 | K.IECVSAETTEDCIAK.I | 1726.8484 | 0.4554 | 2 | 1 | 2.6615 | 0.4161 | 843.7 | 1 | 17\|28 | 4 | 2 | 2 |  |
|  | R16062_1_CTGF,14307 | K.SDNCEDTPEAGYFAVAVVK.K | 2073.1962 | 0.7702 | 2 | 1 | 2.3021 | 0.3521 | 531.3 | 1 | 16\|36 | 3.92 | 2 | 2 |  |
|  | R16062_1_CTGF,14316 | K.SDNCEDTPEAGYFAVAVVK.K | 2073.1962 | 0.8532 | 2 | 1 | 2.4285 | 0.4684 | 258.6 | 1 | 16\|36 | 3.92 | 2 | 2 |  |
|  | R16062_1_CTGF,8292 | K.SVEEYANCHLAR.A | 1449.5444 | 0.5804 | 2 | 1 | 2.2304 | 0.3728 | 587.9 | 1 | 13\|22 | 5.38 | 1 | 1 |  |
|  | R16062_1_CTGF,10111 | K.SVIPSDGPSVACVK.K | 1416.5958 | 0.9808 | 2 | 1 | 2.2711 | 0.4774 | 263.1 | 1 | 16\|26 | 5.55 | 2 | 2 |  |
|  | R16062_1_CTGF,13747 | R.DQYELLCLDNTR.K | 1540.6506 | 0.9546 | 2 | 1 | 2.4969 | 0.3701 | 1085.2 | 1 | 16\|22 | 4.03 | 2 | 2 |  |
|  | R16062_1_CTGF,13755 | R.DQYELLCLDNTR.K | 1540.6506 | 0.6456 | 2 | 1 | 3.3421 | 0.3648 | 1659 | 1 | 17\|22 | 4.03 | 2 | 2 |  |
|  | R16062_1_CTGF,12685 | R.FDEFFSEGCAPGSK.K | 1578.6542 | 0.6792 | 2 | 1 | 3.3023 | 0.5207 | 707.8 | 1 | 15\|26 | 4.14 | 2 | 2 |  |
|  | R16062_1_CTGF,19663 | R.SM*GGKEDLIWELLNQAQEHFGK.D | 2547.8282 | -0.7488 | 3 | 1 | 4.2052 | 0.3899 | 1366.1 | 1 | 28\|84 | 4.83 | 2 | 2 |  |
| $6-1 |  | 18 | 8 | 35.31% | 35936.8 | 4.9 | sp\|P08758\|ANXA5_HUMAN Annexin A5 OS=Homo sapiens GN=ANXA5 PE=1 SV=2 |  |  |  |  |  |  |  |  |
| $6-2 |  | 18 | 8 | 35.31% | 35936.8 | 4.9 | tr\|V9HWE0\|V9HWE0_HUMAN Annexin OS=Homo sapiens GN=HEL-S-7 PE=2 SV=1 |  |  |  |  |  |  |  |  |
|  | R16062_1_CTGF,19685 | K.GLGTDEESILTLLTSR.S | 1705.8887 | -0.5553 | 2 | 1 | 3.3072 | 0.5538 | 937.3 | 1 | 16\|30 | 4.14 | 1 | 2 |  |
|  | R16062_1_CTGF,19703 | K.GLGTDEESILTLLTSR.S | 1705.8887 | 0.4527 | 2 | 1 | 3.179 | 0.4977 | 1079.8 | 1 | 16\|30 | 4.14 | 1 | 2 |  |
|  | R16062_1_CTGF,19715 | K.GLGTDEESILTLLTSR.S | 1705.8887 | 0.5537 | 2 | 1 | 3.3043 | 0.3552 | 1137.2 | 1 | 18\|30 | 4.14 | 1 | 2 |  |
|  | R16062_1_CTGF,19739 | K.GLGTDEESILTLLTSR.S | 1705.8887 | -2.6883 | 2 | 1 | 3.1083 | 0.2506 | 1166.2 | 1 | 17\|30 | 4.14 | 1 | 2 |  |
|  | R16062_1_CTGF,19837 | K.GLGTDEESILTLLTSR.S | 1705.8887 | 1.8037 | 2 | 1 | 2.9348 | 0.3797 | 961.2 | 1 | 18\|30 | 4.14 | 1 | 2 |  |
|  | R16062_1_CTGF,15085 | K.NFATSLYSM*IK.G | 1291.4984 | 0.3624 | 2 | 1 | 2.3297 | 0.2615 | 618.7 | 1 | 13\|20 | 8.59 | 1 | 2 |  |
|  | R16062_1_CTGF,10848 | K.VLTEIIASR.T | 1002.1901 | -0.7539 | 2 | 1 | 2.8043 | 0.3984 | 1474.6 | 1 | 15\|16 | 5.97 | 1 | 2 |  |
|  | R16062_1_CTGF,10859 | K.VLTEIIASR.T | 1002.1901 | -0.0049 | 2 | 1 | 2.7024 | 0.3963 | 1555.5 | 1 | 15\|16 | 5.97 | 1 | 2 |  |
|  | R16062_1_CTGF,10969 | K.VLTEIIASR.T | 1002.1901 | 1.4041 | 2 | 1 | 2.8274 | 0.4444 | 878.8 | 1 | 14\|16 | 5.97 | 1 | 2 |  |
|  | R16062_1_CTGF,19052 | R.DLLDDLKSELTGK.F | 1447.6132 | 1.0502 | 2 | 1 | 2.9312 | 0.4224 | 935.4 | 1 | 15\|24 | 4.23 | 1 | 2 |  |
|  | R16062_1_CTGF,18997 | R.DPDAGIDEAQVEQDAQALFQAGELK.W | 2659.8007 | -0.8623 | 2 | 1 | 3.203 | 0.3948 | 1194.6 | 1 | 22\|48 | 3.62 | 1 | 2 |  |
|  | R16062_1_CTGF,20319 | R.ETSGNLEQLLLAVVK.S | 1614.8655 | 0.4545 | 2 | 1 | 3.0026 | 0.4211 | 654.3 | 1 | 15\|28 | 4.53 | 1 | 2 |  |
|  | R16062_1_CTGF,20337 | R.ETSGNLEQLLLAVVK.S | 1614.8655 | -0.6865 | 2 | 1 | 4.0669 | 0.471 | 829.5 | 1 | 15\|28 | 4.53 | 1 | 2 |  |
|  | R16062_1_CTGF,20341 | R.ETSGNLEQLLLAVVK.S | 1614.8655 | 0.4585 | 2 | 1 | 3.3611 | 0.4698 | 1123.8 | 1 | 18\|28 | 4.53 | 1 | 2 |  |
|  | R16062_1_CTGF,20353 | R.ETSGNLEQLLLAVVK.S | 1614.8655 | 1.5095 | 2 | 1 | 2.9217 | 0.4465 | 809 | 1 | 15\|28 | 4.53 | 1 | 2 |  |
|  | R16062_1_CTGF,15710 | R.SEIDLFNIR.K | 1107.2417 | 0.2117 | 2 | 1 | 2.6969 | 0.2216 | 977.5 | 3 | 13\|16 | 4.37 | 1 | 2 |  |
|  | R16062_1_CTGF,15721 | R.SEIDLFNIR.K | 1107.2417 | 0.8797 | 2 | 1 | 2.4225 | 0.2145 | 1149.9 | 4 | 14\|16 | 4.37 | 1 | 2 |  |
|  | R16062_1_CTGF,20555 | R.SIPAYLAETLYYAMK.G | 1735.0367 | -0.1953 | 2 | 1 | 2.2265 | 0.3752 | 179.5 | 3 | 11\|28 | 5.72 | 1 | 2 |  |
| $7-1 |  | 17 | 8 | 19.59% | 57936.9 | 8 | sp\|P14618\|KPYM_HUMAN Pyruvate kinase PKM OS=Homo sapiens GN=PKM PE=1 SV=4 |  |  |  |  |  |  |  |  |
| $7-2 |  | 17 | 8 | 19.59% | 57936.9 | 8 | tr\|V9HWB8\|V9HWB8_HUMAN Pyruvate kinase OS=Homo sapiens GN=HEL-S-30 PE=1 SV=1 |  |  |  |  |  |  |  |  |
|  | R16062_1_CTGF,13772 | K.GADFLVTEVENGGSLGSK.K | 1780.9141 | -1.2399 | 2 | 1 | 2.8393 | 0.3546 | 702.3 | 1 | 16\|34 | 4.14 | 1 | 2 |  |
|  | R16062_1_CTGF,13811 | K.GADFLVTEVENGGSLGSK.K | 1780.9141 | 0.4181 | 2 | 1 | 3.3777 | 0.346 | 1076.6 | 1 | 19\|34 | 4.14 | 1 | 2 |  |
|  | R16062_1_CTGF,13831 | K.GADFLVTEVENGGSLGSK.K | 1780.9141 | -0.2199 | 2 | 1 | 3.7212 | 0.5339 | 1826.5 | 1 | 22\|34 | 4.14 | 1 | 2 |  |
|  | R16062_1_CTGF,14930 | K.GADFLVTEVENGGSLGSK.K | 1780.9141 | 0.6461 | 2 | 1 | 2.9268 | 0.2783 | 1127.7 | 1 | 17\|34 | 4.14 | 1 | 2 |  |
|  | R16062_1_CTGF,14945 | K.GADFLVTEVENGGSLGSK.K | 1780.9141 | 1.2571 | 2 | 1 | 2.717 | 0.2841 | 881.1 | 1 | 16\|34 | 4.14 | 1 | 2 |  |
|  | R16062_1_CTGF,15351 | K.GADFLVTEVENGGSLGSK.K | 1780.9141 | -0.5859 | 2 | 1 | 2.5236 | 0.2802 | 563.8 | 1 | 15\|34 | 4.14 | 1 | 2 |  |
|  | R16062_1_CTGF,13683 | K.GVNLPGAAVDLPAVSEK.D | 1637.8591 | 0.9251 | 2 | 1 | 2.8664 | 0.4728 | 730.7 | 1 | 16\|32 | 4.37 | 1 | 2 |  |
|  | R16062_1_CTGF,13690 | K.GVNLPGAAVDLPAVSEK.D | 1637.8591 | 0.4631 | 2 | 1 | 2.4179 | 0.3644 | 780.9 | 1 | 17\|32 | 4.37 | 1 | 2 |  |
|  | R16062_1_CTGF,15756 | K.IYVDDGLISLQVK.Q | 1463.7006 | 0.6766 | 2 | 1 | 2.6665 | 0.2918 | 763.4 | 1 | 15\|24 | 4.21 | 1 | 2 |  |
|  | R16062_1_CTGF,15791 | K.IYVDDGLISLQVK.Q | 1463.7006 | -0.7024 | 2 | 1 | 2.7639 | 0.2972 | 756.9 | 1 | 15\|24 | 4.21 | 1 | 2 |  |
|  | R16062_1_CTGF,15827 | K.IYVDDGLISLQVK.Q | 1463.7006 | 0.9546 | 2 | 1 | 2.2794 | 0.3693 | 276.2 | 3 | 11\|24 | 4.21 | 1 | 2 |  |
|  | R16062_1_CTGF,12731 | K.KGVNLPGAAVDLPAVSEK.D | 1766.0321 | -0.2339 | 2 | 1 | 2.2585 | 0.4541 | 455.9 | 1 | 13\|34 | 6.07 | 1 | 2 |  |
|  | R16062_1_CTGF,11992 | R.GDLGIEIPAEK.V | 1142.2843 | -0.7447 | 2 | 1 | 2.2519 | 0.3907 | 1035.5 | 1 | 15\|20 | 4.14 | 1 | 2 |  |
|  | R16062_1_CTGF,16388 | R.LAPITSDPTEATAVGAVEASFK.C | 2176.4092 | 1.5482 | 2 | 1 | 4.0117 | 0.4869 | 1260.9 | 1 | 28\|42 | 4.14 | 1 | 2 |  |
|  | R16062_1_CTGF,16475 | R.LAPITSDPTEATAVGAVEASFK.C | 2176.4092 | 1.7762 | 2 | 1 | 2.4288 | 0.3315 | 424.5 | 1 | 17\|42 | 4.14 | 1 | 2 |  |
|  | R16062_1_CTGF,10659 | R.NTGIICTIGPASR.S | 1360.5355 | 2.2165 | 2 | 1 | 2.2466 | 0.4829 | 892.7 | 1 | 15\|24 | 8.25 | 1 | 2 |  |
|  | R16062_1_CTGF,8198 | R.PVAVALDTK.G | 914.0814 | 0.5604 | 2 | 1 | 2.3866 | 0.3372 | 962.1 | 1 | 13\|16 | 6.26 | 1 | 2 |  |
| $8-1 |  | 13 | 8 | 15.36% | 74139.7 | 6.6 | sp\|P02545\|LMNA_HUMAN Prelamin-A/C OS=Homo sapiens GN=LMNA PE=1 SV=1 |  |  |  |  |  |  |  |  |
|  | R16062_1_CTGF,14065 | K.DLEALLNSK.E | 1003.1318 | 0.1818 | 2 | 1 | 2.7228 | 0.3587 | 957.1 | 2 | 12\|16 | 4.37 | 1 | 1 |  |
|  | R16062_1_CTGF,13106 | R.IDSLSAQLSQLQK.Q | 1431.617 | 0.467 | 2 | 1 | 2.3741 | 0.3169 | 832.6 | 1 | 15\|24 | 5.84 | 1 | 1 |  |
|  | R16062_1_CTGF,6682 | R.ITESEEVVSR.E | 1149.2337 | -0.5763 | 2 | 1 | 2.7344 | 0.4243 | 983.2 | 1 | 13\|18 | 4.25 | 1 | 1 |  |
|  | R16062_1_CTGF,6692 | R.ITESEEVVSR.E | 1149.2337 | 0.2137 | 2 | 1 | 2.6696 | 0.3953 | 756.5 | 1 | 13\|18 | 4.25 | 1 | 1 |  |
|  | R16062_1_CTGF,15833 | R.M*QQQLDEYQELLDIK.L | 1911.1231 | 0.8171 | 2 | 1 | 3.5161 | 0.4774 | 1739.7 | 1 | 21\|28 | 3.92 | 1 | 1 |  |
|  | R16062_1_CTGF,15843 | R.M*QQQLDEYQELLDIK.L | 1911.1231 | -0.4119 | 2 | 1 | 4.7341 | 0.5381 | 1336.3 | 1 | 20\|28 | 3.92 | 1 | 1 |  |
|  | R16062_1_CTGF,15898 | R.M*QQQLDEYQELLDIK.L | 1911.1231 | 0.7801 | 2 | 1 | 3.6612 | 0.5137 | 2369.7 | 1 | 22\|28 | 3.92 | 1 | 1 |  |
|  | R16062_1_CTGF,9859 | R.NSNLVGAAHEELQQSR.I | 1753.8552 | 0.5002 | 2 | 1 | 3.0057 | 0.579 | 875.5 | 1 | 16\|30 | 5.4 | 1 | 1 |  |
|  | R16062_1_CTGF,9869 | R.NSNLVGAAHEELQQSR.I | 1753.8552 | 0.6102 | 2 | 1 | 3.244 | 0.5319 | 1067.6 | 1 | 18\|30 | 5.4 | 1 | 1 |  |
|  | R16062_1_CTGF,6544 | R.SGAQASSTPLSPTR.I | 1360.4557 | 1.0197 | 2 | 1 | 2.3013 | 0.4278 | 690.9 | 1 | 15\|26 | 9.47 | 1 | 1 |  |
|  | R16062_1_CTGF,9191 | R.TALINSTGEEVAM*R.K | 1508.6801 | 0.4431 | 2 | 1 | 2.5852 | 0.4691 | 1190 | 1 | 17\|26 | 4.53 | 1 | 1 |  |
|  | R16062_1_CTGF,6954 | R.VAVEEVDEEGK.F | 1204.2662 | 0.3262 | 2 | 1 | 2.4868 | 0.4971 | 1136.5 | 1 | 16\|20 | 3.91 | 1 | 1 |  |
|  | R16062_1_CTGF,6965 | R.VAVEEVDEEGK.F | 1204.2662 | 0.4052 | 2 | 1 | 2.5466 | 0.4938 | 1209.3 | 1 | 17\|20 | 3.91 | 1 | 1 |  |
| $9-1 |  | 23 | 7 | 21.87% | 41736.8 | 5.3 | sp\|P60709\|ACTB_HUMAN Actin, cytoplasmic 1 OS=Homo sapiens GN=ACTB PE=1 SV=1 |  |  |  |  |  |  |  |  |
| $9-2 |  | 23 | 7 | 21.87% | 41792.9 | 5.3 | sp\|P63261\|ACTG_HUMAN Actin, cytoplasmic 2 OS=Homo sapiens GN=ACTG1 PE=1 SV=1 |  |  |  |  |  |  |  |  |
| $9-3 |  | 23 | 7 | 21.87% | 41736.8 | 5.3 | tr\|Q1KLZ0\|Q1KLZ0_HUMAN HCG15971, isoform CRA_a OS=Homo sapiens GN=PS1TP5BP1 PE=2 SV=1 |  |  |  |  |  |  |  |  |
| $9-4 |  | 23 | 7 | 22.59% | 40503.4 | 5.8 | tr\|Q8WVW5\|Q8WVW5_HUMAN Putative uncharacterized protein (Fragment) OS=Homo sapiens PE=2 SV=1 |  |  |  |  |  |  |  |  |
| $9-5 |  | 23 | 7 | 21.87% | 41720.7 | 5.3 | tr\|Q53G76\|Q53G76_HUMAN Beta actin variant (Fragment) OS=Homo sapiens PE=2 SV=1 |  |  |  |  |  |  |  |  |
| $9-6 |  | 23 | 7 | 23.36% | 39226 | 5.4 | tr\|B4E335\|B4E335_HUMAN cDNA FLJ52842, highly similar to Actin, cytoplasmic 1 OS=Homo sapiens PE=2 SV=1 |  |  |  |  |  |  |  |  |
| $9-7 |  | 23 | 7 | 21.87% | 41764.8 | 5.4 | tr\|Q53G99\|Q53G99_HUMAN Beta actin variant (Fragment) OS=Homo sapiens PE=2 SV=1 |  |  |  |  |  |  |  |  |
| $9-8 |  | 23 | 7 | 21.87% | 41722.7 | 5.3 | tr\|Q53GK6\|Q53GK6_HUMAN Beta actin variant (Fragment) OS=Homo sapiens PE=2 SV=1 |  |  |  |  |  |  |  |  |
| $9-9 |  | 23 | 7 | 24.62% | 37348.9 | 5.5 | tr\|B4DVQ0\|B4DVQ0_HUMAN cDNA FLJ58286, highly similar to Actin, cytoplasmic 2 OS=Homo sapiens PE=2 SV=1 |  |  |  |  |  |  |  |  |
| $9-10 | | 23 | 7 | 23.03% | 39799.5 | 5.2 | tr\|B4E3A4\|B4E3A4_HUMAN cDNA FLJ57283, highly similar to Actin, cytoplasmic 2 OS=Homo sapiens PE=2 SV=1 |  |  |  |  |  |  |  |  |
|  | R16062_1_CTGF,6607 | K.AGFAGDDAPR.A | 977.013 | 1.549 | 2 | 1 | 2.2568 | 0.2115 | 1142.9 | 1 | 15\|18 | 4.21 | 2 | 19 |  |
|  | R16062_1_CTGF,6944 | K.AGFAGDDAPR.A | 977.013 | -0.146 | 2 | 1 | 2.201 | 0.3391 | 858.1 | 1 | 13\|18 | 4.21 | 2 | 19 |  |
|  | R16062_1_CTGF,14071 | K.DLYANTVLSGGTTM*YPGIADR.M | 2232.4564 | 0.9814 | 2 | 1 | 2.8455 | 0.463 | 919.6 | 1 | 18\|40 | 4.21 | 1 | 10 |  |
|  | R16062_1_CTGF,14083 | K.DLYANTVLSGGTTM*YPGIADR.M | 2232.4564 | 0.1924 | 2 | 1 | 2.6605 | 0.4503 | 1234.1 | 1 | 20\|40 | 4.21 | 1 | 10 |  |
|  | R16062_1_CTGF,14107 | K.DLYANTVLSGGTTM*YPGIADR.M | 2232.4564 | 1.5634 | 2 | 1 | 3.1445 | 0.5224 | 1134.7 | 1 | 21\|40 | 4.21 | 1 | 10 |  |
|  | R16062_1_CTGF,14171 | K.DLYANTVLSGGTTM*YPGIADR.M | 2232.4564 | 1.6204 | 2 | 1 | 3.0789 | 0.5385 | 1073.2 | 1 | 21\|40 | 4.21 | 1 | 10 |  |
|  | R16062_1_CTGF,5612 | K.DSYVGDEAQSK.R | 1199.2065 | 0.2925 | 2 | 1 | 2.5233 | 0.4763 | 1016.9 | 1 | 17\|20 | 4.03 | 2 | 19 |  |
|  | R16062_1_CTGF,5623 - 5624 | K.DSYVGDEAQSK.R | 1199.2065 | -0.0155 | 2 | 1 | 2.7277 | 0.3658 | 792.8 | 1 | 18\|20 | 4.03 | 2 | 19 |  |
|  | R16062_1_CTGF,5634 | K.DSYVGDEAQSK.R | 1199.2065 | 0.8525 | 2 | 1 | 2.541 | 0.5368 | 1034.9 | 1 | 17\|20 | 4.03 | 2 | 19 |  |
|  | R16062_1_CTGF,7749 | K.QEYDESGPSIVHR.K | 1517.5822 | 1.1862 | 2 | 1 | 2.3847 | 0.5904 | 538 | 1 | 12\|24 | 4.65 | 1 | 10 |  |
|  | R16062_1_CTGF,13401 | K.SYELPDGQVITIGNER.F | 1791.9401 | 0.5051 | 2 | 1 | 2.6408 | 0.4404 | 752.6 | 1 | 20\|30 | 4.14 | 2 | 19 |  |
|  | R16062_1_CTGF,13444 | K.SYELPDGQVITIGNER.F | 1791.9401 | 0.5331 | 2 | 1 | 2.4361 | 0.3534 | 789 | 1 | 19\|30 | 4.14 | 2 | 19 |  |
|  | R16062_1_CTGF,14481 | K.SYELPDGQVITIGNER.F | 1791.9401 | 0.0741 | 2 | 1 | 2.5171 | 0.4775 | 1160.3 | 1 | 18\|30 | 4.14 | 2 | 19 |  |
|  | R16062_1_CTGF,14518 | K.SYELPDGQVITIGNER.F | 1791.9401 | 1.4201 | 2 | 1 | 2.9243 | 0.5515 | 897.4 | 1 | 18\|30 | 4.14 | 2 | 19 |  |
|  | R16062_1_CTGF,14541 | K.SYELPDGQVITIGNER.F | 1791.9401 | 1.3251 | 2 | 1 | 2.5249 | 0.5202 | 891.8 | 1 | 18\|30 | 4.14 | 2 | 19 |  |
|  | R16062_1_CTGF,14941 | K.SYELPDGQVITIGNER.F | 1791.9401 | 0.0721 | 2 | 1 | 3.7924 | 0.5219 | 1372.9 | 1 | 21\|30 | 4.14 | 2 | 19 |  |
|  | R16062_1_CTGF,14953 | K.SYELPDGQVITIGNER.F | 1791.9401 | -0.5989 | 2 | 1 | 3.4993 | 0.5054 | 1279 | 1 | 22\|30 | 4.14 | 2 | 19 |  |
|  | R16062_1_CTGF,15011 | K.SYELPDGQVITIGNER.F | 1791.9401 | 1.0781 | 2 | 1 | 2.8991 | 0.3201 | 768.3 | 1 | 18\|30 | 4.14 | 2 | 19 |  |
|  | R16062_1_CTGF,8951 | R.GYSFTTTAER.E | 1133.1928 | -1.5132 | 2 | 1 | 2.8464 | 0.5588 | 1233.3 | 1 | 15\|18 | 6 | 1 | 10 |  |
|  | R16062_1_CTGF,8989 | R.GYSFTTTAER.E | 1133.1928 | 0.2688 | 2 | 1 | 2.4345 | 0.3357 | 859 | 1 | 15\|18 | 6 | 1 | 10 |  |
|  | R16062_1_CTGF,9000 | R.GYSFTTTAER.E | 1133.1928 | 0.3288 | 2 | 1 | 2.4725 | 0.1953 | 792.5 | 1 | 15\|18 | 6 | 1 | 10 |  |
|  | R16062_1_CTGF,9034 | R.GYSFTTTAER.E | 1133.1928 | 1.4888 | 2 | 1 | 2.4456 | 0.3561 | 896.9 | 1 | 15\|18 | 6 | 1 | 10 |  |
|  | R16062_1_CTGF,12678 | R.KDLYANTVLSGGTTM*YPGIADR.M | 2360.6293 | 2.4543 | 3 | 1 | 3.9712 | 0.5963 | 1199 | 1 | 31\|84 | 5.96 | 1 | 10 |  |
| $10-1 | | 16 | 7 | 23.56% | 43810.1 | 9.5 | sp\|P51888\|PRELP_HUMAN Prolargin OS=Homo sapiens GN=PRELP PE=1 SV=1 |  |  |  |  |  |  |  |  |
| $10-2 | | 16 | 7 | 23.56% | 43783.1 | 9.5 | tr\|Q6FHG6\|Q6FHG6_HUMAN PRELP protein (Fragment) OS=Homo sapiens GN=PRELP PE=2 SV=1 |  |  |  |  |  |  |  |  |
|  | R16062_1_CTGF,12136 | K.IETIPNGYFK.S | 1182.3501 | -1.7829 | 2 | 1 | 2.346 | 0.1595 | 942.3 | 1 | 14\|18 | 6 | 1 | 2 |  |
|  | R16062_1_CTGF,12146 | K.IETIPNGYFK.S | 1182.3501 | 0.0021 | 2 | 1 | 2.2298 | 0.2899 | 598.5 | 1 | 14\|18 | 6 | 1 | 2 |  |
|  | R16062_1_CTGF,12159 | K.IETIPNGYFK.S | 1182.3501 | 0.0811 | 2 | 1 | 2.4866 | 0.3016 | 535.9 | 1 | 12\|18 | 6 | 1 | 2 |  |
|  | R16062_1_CTGF,15946 | K.LENLLLLDLQHNR.L | 1591.8368 | 1.3148 | 2 | 1 | 2.6669 | 0.4511 | 751.3 | 1 | 13\|24 | 5.32 | 1 | 2 |  |
|  | R16062_1_CTGF,15954 | K.LENLLLLDLQHNR.L | 1591.8368 | 1.2158 | 2 | 1 | 2.6466 | 0.3913 | 883.7 | 1 | 15\|24 | 5.32 | 1 | 2 |  |
|  | R16062_1_CTGF,17015 | K.LPGLVFLYM*EK.N | 1326.629 | -0.669 | 2 | 1 | 2.6732 | 0.4455 | 615.6 | 1 | 13\|20 | 6 | 1 | 2 |  |
|  | R16062_1_CTGF,18904 | K.LPGLVFLYMEK.N | 1310.6296 | 0.5506 | 2 | 1 | 2.9084 | 0.4651 | 1209 | 1 | 17\|20 | 6 | 1 | 2 |  |
|  | R16062_1_CTGF,11604 | K.NQLEEVPSALPR.N | 1353.5063 | -0.6077 | 2 | 1 | 2.8083 | 0.293 | 750.5 | 1 | 13\|22 | 4.53 | 1 | 2 |  |
|  | R16062_1_CTGF,14346 | R.ECYCPPDFPSALYCDSR.N | 2138.2568 | 0.8548 | 2 | 1 | 2.4559 | 0.4508 | 471 | 1 | 17\|32 | 4.03 | 1 | 2 |  |
|  | R16062_1_CTGF,14368 | R.ECYCPPDFPSALYCDSR.N | 2138.2568 | 0.8458 | 2 | 1 | 2.2461 | 0.4833 | 520.2 | 1 | 18\|32 | 4.03 | 1 | 2 |  |
|  | R16062_1_CTGF,10139 | R.LEHLYLNNNSIEK.I | 1587.7587 | -0.1483 | 2 | 1 | 2.638 | 0.3672 | 994.1 | 1 | 15\|24 | 5.4 | 1 | 2 |  |
|  | R16062_1_CTGF,10160 | R.LEHLYLNNNSIEK.I | 1587.7587 | 1.0837 | 2 | 1 | 2.7077 | 0.3682 | 754.9 | 1 | 16\|24 | 5.4 | 1 | 2 |  |
|  | R16062_1_CTGF,11384 | R.VPTAIHQLYLDSNK.I | 1599.8126 | 0.5746 | 2 | 1 | 2.5645 | 0.4045 | 952.9 | 1 | 16\|26 | 6.71 | 1 | 2 |  |
|  | R16062_1_CTGF,11391 | R.VPTAIHQLYLDSNK.I | 1599.8126 | -0.7054 | 2 | 1 | 2.4207 | 0.4757 | 640 | 1 | 13\|26 | 6.71 | 1 | 2 |  |
|  | R16062_1_CTGF,11412 | R.VPTAIHQLYLDSNK.I | 1599.8126 | 0.3186 | 2 | 1 | 2.7478 | 0.5513 | 1136.5 | 1 | 18\|26 | 6.71 | 1 | 2 |  |
|  | R16062_1_CTGF,11420 | R.VPTAIHQLYLDSNK.I | 1599.8126 | 0.5906 | 2 | 1 | 4.1431 | 0.6082 | 1303.4 | 1 | 19\|26 | 6.71 | 1 | 2 |  |
| $11-1 | | 15 | 7 | 59.86% | 15998.4 | 6.7 | sp\|P68871\|HBB_HUMAN Hemoglobin subunit beta OS=Homo sapiens GN=HBB PE=1 SV=2 |  |  |  |  |  |  |  |  |
| $11-2 | | 15 | 7 | 59.86% | 15998.4 | 6.7 | tr\|D9YZU5\|D9YZU5_HUMAN Hemoglobin, beta OS=Homo sapiens GN=HBB PE=3 SV=1 |  |  |  |  |  |  |  |  |
|  | R16062_1_CTGF,9579 | K.LHVDPENFR.L | 1127.2347 | -1.7913 | 2 | 1 | 2.3337 | 0.2283 | 566.3 | 1 | 11\|16 | 5.32 | 4 | 6 |  |
|  | R16062_1_CTGF,12466 | K.SAVTALWGK.V | 933.0864 | 1.0334 | 2 | 1 | 2.5672 | 0.4622 | 944.5 | 1 | 13\|16 | 8.47 | 3 | 4 |  |
|  | R16062_1_CTGF,14848 | K.VLGAFSDGLAHLDNLK.G | 1670.8908 | -1.0252 | 2 | 1 | 2.5143 | 0.1645 | 646 | 1 | 17\|30 | 5.21 | 3 | 5 |  |
|  | R16062_1_CTGF,15391 | K.VLGAFSDGLAHLDNLK.G | 1670.8908 | -2.9452 | 2 | 1 | 2.625 | 0.1512 | 871.8 | 1 | 16\|30 | 5.21 | 3 | 5 |  |
|  | R16062_1_CTGF,10320 | K.VNVDEVGGEALGR.L | 1315.415 | -2.893 | 2 | 1 | 3.1366 | 0.2348 | 1612.6 | 1 | 18\|24 | 4.14 | 2 | 3 |  |
|  | R16062_1_CTGF,10663 | K.VNVDEVGGEALGR.L | 1315.415 | -1.054 | 2 | 1 | 2.8368 | 0.1924 | 1003.9 | 1 | 16\|24 | 4.14 | 2 | 3 |  |
|  | R16062_1_CTGF,10666 | K.VNVDEVGGEALGR.L | 1315.415 | 0.907 | 2 | 1 | 3.1979 | 0.168 | 947.4 | 2 | 17\|24 | 4.14 | 2 | 3 |  |
|  | R16062_1_CTGF,10684 | K.VNVDEVGGEALGR.L | 1315.415 | 0.34 | 2 | 1 | 3.0868 | 0.2112 | 1151.3 | 2 | 17\|24 | 4.14 | 2 | 3 |  |
|  | R16062_1_CTGF,8571 | K.VVAGVANALAHK.Y | 1150.3561 | 0.4061 | 2 | 1 | 2.571 | 0.4834 | 838.3 | 1 | 17\|22 | 8.73 | 2 | 4 |  |
|  | R16062_1_CTGF,8582 | K.VVAGVANALAHK.Y | 1150.3561 | 0.2791 | 2 | 1 | 2.9539 | 0.5515 | 834.6 | 1 | 17\|22 | 8.73 | 2 | 4 |  |
|  | R16062_1_CTGF,14767 | R.FFESFGDLSTPDAVM*GNPK.V | 2076.2717 | 0.0897 | 2 | 1 | 2.499 | 0.1744 | 609.8 | 1 | 16\|36 | 4.03 | 3 | 4 |  |
|  | R16062_1_CTGF,15229 | R.FFESFGDLSTPDAVM*GNPK.V | 2076.2717 | 0.5327 | 2 | 1 | 2.2769 | 0.2597 | 260.2 | 1 | 11\|36 | 4.03 | 3 | 4 |  |
|  | R16062_1_CTGF,15300 | R.FFESFGDLSTPDAVM*GNPK.V | 2076.2717 | -0.3443 | 2 | 1 | 2.4545 | 0.1603 | 347.6 | 1 | 14\|36 | 4.03 | 3 | 4 |  |
|  | R16062_1_CTGF,15677 | R.LLVVYPWTQR.F | 1275.5235 | 1.0095 | 2 | 1 | 2.2141 | 0.3369 | 1095.1 | 1 | 15\|18 | 8.75 | 4 | 6 |  |
|  | R16062_1_CTGF,15820 | R.LLVVYPWTQR.F | 1275.5235 | -2.2995 | 2 | 1 | 2.5806 | 0.2315 | 710.6 | 1 | 12\|18 | 8.75 | 4 | 6 |  |
| $12-1 | | 14 | 7 | 16.95% | 53651.9 | 5.1 | sp\|P08670\|VIME_HUMAN Vimentin OS=Homo sapiens GN=VIM PE=1 SV=4 |  |  |  |  |  |  |  |  |
| $12-2 | | 14 | 7 | 16.95% | 53651.9 | 5.1 | tr\|V9HWE1\|V9HWE1_HUMAN Epididymis luminal protein 113 OS=Homo sapiens GN=HEL113 PE=2 SV=1 |  |  |  |  |  |  |  |  |
|  | R16062_1_CTGF,8759 | K.FADLSEAANR.N | 1094.1599 | 0.9619 | 2 | 1 | 2.3366 | 0.3929 | 895.7 | 1 | 14\|18 | 4.37 | 1 | 2 |  |
|  | R16062_1_CTGF,8960 | K.FADLSEAANR.N | 1094.1599 | 1.8659 | 2 | 1 | 2.2309 | 0.3942 | 630.5 | 1 | 13\|18 | 4.37 | 1 | 2 |  |
|  | R16062_1_CTGF,16107 | K.ILLAELEQLK.G | 1170.424 | 0.321 | 2 | 1 | 3.076 | 0.2055 | 1460.7 | 1 | 16\|18 | 4.53 | 1 | 2 |  |
|  | R16062_1_CTGF,16118 | K.ILLAELEQLK.G | 1170.424 | 0.722 | 2 | 1 | 2.5521 | 0.2688 | 1289.6 | 1 | 16\|18 | 4.53 | 1 | 2 |  |
|  | R16062_1_CTGF,9365 | K.VELQELNDR.F | 1116.207 | -1.592 | 2 | 1 | 2.6238 | 0.1962 | 1138 | 1 | 14\|16 | 4.14 | 1 | 2 |  |
|  | R16062_1_CTGF,9541 | K.VELQELNDR.F | 1116.207 | 0.124 | 2 | 1 | 2.3123 | 0.2345 | 735.4 | 1 | 12\|16 | 4.14 | 1 | 2 |  |
|  | R16062_1_CTGF,9509 | R.DNLAEDIM*R.L | 1093.1936 | -0.4824 | 2 | 1 | 2.2705 | 0.255 | 761.1 | 2 | 12\|16 | 4.03 | 1 | 2 |  |
|  | R16062_1_CTGF,12120 | R.ETNLDSLPLVDTHSK.R | 1669.8149 | 0.9879 | 2 | 1 | 2.4058 | 0.3126 | 299.6 | 1 | 13\|28 | 4.54 | 1 | 2 |  |
|  | R16062_1_CTGF,14974 | R.KVESLQEEIAFLK.K | 1534.7788 | 0.6618 | 3 | 1 | 3.9666 | 0.3422 | 1998.7 | 1 | 26\|48 | 4.79 | 1 | 2 |  |
|  | R16062_1_CTGF,14993 | R.KVESLQEEIAFLK.K | 1534.7788 | 2.1508 | 2 | 1 | 2.7365 | 0.261 | 1084.7 | 1 | 16\|24 | 4.79 | 1 | 2 |  |
|  | R16062_1_CTGF,14996 | R.KVESLQEEIAFLK.K | 1534.7788 | 0.4748 | 3 | 1 | 4.1333 | 0.3105 | 1862.3 | 1 | 29\|48 | 4.79 | 1 | 2 |  |
|  | R16062_1_CTGF,15047 | R.KVESLQEEIAFLK.K | 1534.7788 | 2.7698 | 2 | 1 | 2.7329 | 0.3697 | 1751.7 | 1 | 19\|24 | 4.79 | 1 | 2 |  |
|  | R16062_1_CTGF,12494 | R.QVQSLTCEVDALK.G | 1491.6627 | -0.5073 | 2 | 1 | 2.3069 | 0.3353 | 821.3 | 1 | 15\|24 | 4.37 | 1 | 2 |  |
|  | R16062_1_CTGF,12499 | R.QVQSLTCEVDALK.G | 1491.6627 | -0.6133 | 2 | 1 | 2.8606 | 0.4104 | 654.5 | 1 | 14\|24 | 4.37 | 1 | 2 |  |
| $13-1 | | 13 | 7 | 24.59% | 47650.9 | 5.3 | sp\|P01011\|AACT_HUMAN Alpha-1-antichymotrypsin OS=Homo sapiens GN=SERPINA3 PE=1 SV=2 |  |  |  |  |  |  |  |  |
| $13-2 | | 13 | 7 | 24.59% | 47650.9 | 5.3 | tr\|A0A024R6P0\|A0A024R6P0_HUMAN Serpin peptidase inhibitor, clade A (Alpha-1 antiproteinase, antitrypsin), member 3, isoform CRA_c OS=Homo sapiens GN=SERPINA3 PE=3 SV=1 |  |  |  |  |  |  |  |  |
| $13-3 | | 13 | 7 | 23.21% | 50628.3 | 5.4 | tr\|B3KS79\|B3KS79_HUMAN cDNA FLJ35730 fis, clone TESTI2003131, highly similar to ALPHA-1-ANTICHYMOTRYPSIN OS=Homo sapiens PE=2 SV=1 |  |  |  |  |  |  |  |  |
|  | R16062_1_CTGF,8662 | K.ADLSGITGAR.N | 961.055 | 0.306 | 2 | 1 | 2.5663 | 0.341 | 680 | 3 | 12\|18 | 5.88 | 1 | 3 |  |
|  | R16062_1_CTGF,14919 | K.AVLDVFEEGTEASAATAVK.I | 1909.0838 | 0.7718 | 2 | 1 | 2.4458 | 0.3319 | 337.6 | 1 | 15\|36 | 4 | 1 | 3 |  |
|  | R16062_1_CTGF,14954 | K.AVLDVFEEGTEASAATAVK.I | 1909.0838 | 1.1378 | 2 | 1 | 3.7101 | 0.6225 | 781.1 | 1 | 21\|36 | 4 | 1 | 3 |  |
|  | R16062_1_CTGF,17416 | K.ITLLSALVETR.T | 1216.4527 | 0.2497 | 2 | 1 | 3.3059 | 0.5086 | 1461.9 | 1 | 16\|20 | 6 | 1 | 3 |  |
|  | R16062_1_CTGF,17443 | K.ITLLSALVETR.T | 1216.4527 | -0.7893 | 2 | 1 | 3.4463 | 0.4532 | 1063.2 | 1 | 16\|20 | 6 | 1 | 3 |  |
|  | R16062_1_CTGF,17448 | K.ITLLSALVETR.T | 1216.4527 | 0.3477 | 2 | 1 | 3.1709 | 0.4963 | 952.7 | 1 | 15\|20 | 6 | 1 | 3 |  |
|  | R16062_1_CTGF,17470 | K.ITLLSALVETR.T | 1216.4527 | 0.9957 | 2 | 1 | 3.4778 | 0.4668 | 1261.3 | 1 | 16\|20 | 6 | 1 | 3 |  |
|  | R16062_1_CTGF,13726 | K.M*EEVEAM*LLPETLK.R | 1665.9513 | 0.2453 | 2 | 1 | 2.3404 | 0.3924 | 354.1 | 1 | 13\|26 | 4.09 | 1 | 3 |  |
|  | R16062_1_CTGF,12093 | R.DEELSCTVVELK.Y | 1422.554 | -0.784 | 2 | 1 | 2.8546 | 0.2712 | 804.5 | 1 | 13\|22 | 4 | 1 | 3 |  |
|  | R16062_1_CTGF,12104 | R.DEELSCTVVELK.Y | 1422.554 | 0.091 | 2 | 1 | 3.0155 | 0.2445 | 1368.7 | 1 | 17\|22 | 4 | 1 | 3 |  |
|  | R16062_1_CTGF,21531 | R.DYNLNDILLQLGIEEAFTSK.A | 2297.5465 | 0.7015 | 2 | 1 | 2.2577 | 0.3316 | 629.8 | 1 | 17\|38 | 3.92 | 1 | 3 |  |
|  | R16062_1_CTGF,13642 | R.LYGSEAFATDFQDSAAAK.K | 1892.9998 | 0.8458 | 2 | 1 | 2.8015 | 0.4707 | 618.5 | 1 | 17\|34 | 4.03 | 1 | 3 |  |
|  | R16062_1_CTGF,13660 | R.LYGSEAFATDFQDSAAAK.K | 1892.9998 | 0.8308 | 2 | 1 | 2.7459 | 0.4554 | 776.9 | 1 | 17\|34 | 4.03 | 1 | 3 |  |
| $14-1 | | 11 | 7 | 14.12% | 75873.4 | 5.4 | sp\|P08133\|ANXA6_HUMAN Annexin A6 OS=Homo sapiens GN=ANXA6 PE=1 SV=3 |  |  |  |  |  |  |  |  |
|  | R16062_1_CTGF,14765 | K.ALIEILATR.T | 1000.2174 | 0.8534 | 2 | 1 | 2.889 | 0.405 | 1445.1 | 1 | 15\|16 | 6.05 | 1 | 1 |  |
|  | R16062_1_CTGF,14790 | K.ALIEILATR.T | 1000.2174 | 0.2864 | 2 | 1 | 2.8742 | 0.4008 | 1259.2 | 1 | 14\|16 | 6.05 | 1 | 1 |  |
|  | R16062_1_CTGF,15024 | K.CLIEILASR.T | 1075.2777 | 0.1887 | 2 | 1 | 2.3348 | 0.3059 | 543.3 | 1 | 13\|16 | 6 | 1 | 1 |  |
|  | R16062_1_CTGF,15857 | K.EAILDIITSR.S | 1131.3047 | 0.4027 | 2 | 1 | 2.4774 | 0.4184 | 955.9 | 1 | 14\|18 | 4.37 | 1 | 1 |  |
|  | R16062_1_CTGF,15868 | K.EAILDIITSR.S | 1131.3047 | 0.2297 | 2 | 1 | 2.7035 | 0.4581 | 873.6 | 1 | 14\|18 | 4.37 | 1 | 1 |  |
|  | R16062_1_CTGF,18402 | K.GLGTDEDTIIDIITHR.S | 1769.9344 | 0.9504 | 2 | 1 | 3.1649 | 0.4729 | 774.3 | 1 | 15\|30 | 4.22 | 1 | 1 |  |
|  | R16062_1_CTGF,18073 | K.WGTDEAQFIYILGNR.S | 1783.9645 | -0.5425 | 2 | 1 | 2.9031 | 0.3249 | 952.6 | 1 | 16\|28 | 4.37 | 1 | 1 |  |
|  | R16062_1_CTGF,14716 | R.DLEADIIGDTSGHFQK.M | 1746.8562 | 1.5442 | 2 | 1 | 2.2284 | 0.2931 | 577.7 | 1 | 15\|30 | 4.22 | 1 | 1 |  |
|  | R16062_1_CTGF,14721 | R.DLEADIIGDTSGHFQK.M | 1746.8562 | 0.3562 | 2 | 1 | 2.7708 | 0.4525 | 925.9 | 1 | 17\|30 | 4.22 | 1 | 1 |  |
|  | R16062_1_CTGF,16783 | R.EDAQVAAEILEIADTPSGDK.T | 2073.2011 | 1.5101 | 2 | 1 | 3.2496 | 0.5515 | 1072.4 | 1 | 20\|38 | 3.71 | 1 | 1 |  |
|  | R16062_1_CTGF,16811 | R.EDAQVAAEILEIADTPSGDK.T | 2073.2011 | 0.9451 | 2 | 1 | 3.1101 | 0.4337 | 1364.9 | 1 | 20\|38 | 3.71 | 1 | 1 |  |
| $15-1 | | 13 | 6 | 75.25% | 11022.5 | 6 | tr\|Q4TZM4\|Q4TZM4_HUMAN Hemoglobin beta chain (Fragment) OS=Homo sapiens GN=HBB PE=3 SV=1 |  |  |  |  |  |  |  |  |
|  | R16062_1_CTGF,9579 | K.LHVDPENFR.L | 1127.2347 | -1.7913 | 2 | 1 | 2.3337 | 0.2283 | 566.3 | 1 | 11\|16 | 5.32 | 4 | 6 |  |
|  | R16062_1_CTGF,12466 | K.SAVTALWGK.V | 933.0864 | 1.0334 | 2 | 1 | 2.5672 | 0.4622 | 944.5 | 1 | 13\|16 | 8.47 | 3 | 4 |  |
|  | R16062_1_CTGF,15446 | K.VLGAFSNGLAHLDNLK.G | 1669.9061 | 1.2201 | 2 | 1 | 3.4847 | 0.1448 | 1395.2 | 1 | 20\|30 | 6.71 | 1 | 1 |  |
|  | R16062_1_CTGF,15471 | K.VLGAFSNGLAHLDNLK.G | 1669.9061 | 2.0551 | 2 | 1 | 3.8218 | 0.4945 | 1717.5 | 1 | 21\|30 | 6.71 | 1 | 1 |  |
|  | R16062_1_CTGF,10320 | K.VNVDEVGGEALGR.L | 1315.415 | -2.893 | 2 | 1 | 3.1366 | 0.2348 | 1612.6 | 1 | 18\|24 | 4.14 | 2 | 3 |  |
|  | R16062_1_CTGF,10663 | K.VNVDEVGGEALGR.L | 1315.415 | -1.054 | 2 | 1 | 2.8368 | 0.1924 | 1003.9 | 1 | 16\|24 | 4.14 | 2 | 3 |  |
|  | R16062_1_CTGF,10666 | K.VNVDEVGGEALGR.L | 1315.415 | 0.907 | 2 | 1 | 3.1979 | 0.168 | 947.4 | 2 | 17\|24 | 4.14 | 2 | 3 |  |
|  | R16062_1_CTGF,10684 | K.VNVDEVGGEALGR.L | 1315.415 | 0.34 | 2 | 1 | 3.0868 | 0.2112 | 1151.3 | 2 | 17\|24 | 4.14 | 2 | 3 |  |
|  | R16062_1_CTGF,14767 | R.FFESFGDLSTPDAVM*GNPK.V | 2076.2717 | 0.0897 | 2 | 1 | 2.499 | 0.1744 | 609.8 | 1 | 16\|36 | 4.03 | 3 | 4 |  |
|  | R16062_1_CTGF,15229 | R.FFESFGDLSTPDAVM*GNPK.V | 2076.2717 | 0.5327 | 2 | 1 | 2.2769 | 0.2597 | 260.2 | 1 | 11\|36 | 4.03 | 3 | 4 |  |
|  | R16062_1_CTGF,15300 | R.FFESFGDLSTPDAVM*GNPK.V | 2076.2717 | -0.3443 | 2 | 1 | 2.4545 | 0.1603 | 347.6 | 1 | 14\|36 | 4.03 | 3 | 4 |  |
|  | R16062_1_CTGF,15677 | R.LLVVYPWTQR.F | 1275.5235 | 1.0095 | 2 | 1 | 2.2141 | 0.3369 | 1095.1 | 1 | 15\|18 | 8.75 | 4 | 6 |  |
|  | R16062_1_CTGF,15820 | R.LLVVYPWTQR.F | 1275.5235 | -2.2995 | 2 | 1 | 2.5806 | 0.2315 | 710.6 | 1 | 12\|18 | 8.75 | 4 | 6 |  |
| $16-1 | | 12 | 6 | 33.64% | 36105.8 | 8.5 | sp\|P01857\|IGHG1_HUMAN Ig gamma-1 chain C region OS=Homo sapiens GN=IGHG1 PE=1 SV=1 |  |  |  |  |  |  |  |  |
| $16-2 | | 12 | 6 | 23.37% | 52426.1 | 8.5 | tr\|A0A087WYC5\|A0A087WYC5_HUMAN Ig gamma-1 chain C region OS=Homo sapiens GN=IGHG1 PE=1 SV=1 |  |  |  |  |  |  |  |  |
| $16-3 | | 12 | 6 | 23.47% | 51912.6 | 8.3 | tr\|A0A087X079\|A0A087X079_HUMAN Ig gamma-1 chain C region OS=Homo sapiens GN=IGHG1 PE=1 SV=1 |  |  |  |  |  |  |  |  |
| $16-4 | | 12 | 6 | 23.82% | 51153.8 | 7.5 | tr\|A0A087WV47\|A0A087WV47_HUMAN Ig gamma-1 chain C region OS=Homo sapiens GN=IGHG1 PE=1 SV=1 |  |  |  |  |  |  |  |  |
| $16-5 | | 12 | 6 | 27.82% | 43911.8 | 6.6 | tr\|A0A0A0MS08\|A0A0A0MS08_HUMAN Ig gamma-1 chain C region (Fragment) OS=Homo sapiens GN=IGHG1 PE=1 SV=1 |  |  |  |  |  |  |  |  |
| $16-6 | | 12 | 6 | 24.03% | 50488.1 | 8.7 | tr\|A0A087X1C7\|A0A087X1C7_HUMAN Ig gamma-1 chain C region OS=Homo sapiens GN=IGHG1 PE=1 SV=1 |  |  |  |  |  |  |  |  |
| $16-7 | | 12 | 6 | 23.52% | 51596.3 | 8.4 | tr\|A8K008\|A8K008_HUMAN cDNA FLJ78387 OS=Homo sapiens PE=1 SV=1 |  |  |  |  |  |  |  |  |
| $16-8 | | 12 | 6 | 23.87% | 51082.9 | 8.9 | tr\|Q6GMX6\|Q6GMX6_HUMAN IGH@ protein OS=Homo sapiens GN=IGH@ PE=1 SV=1 |  |  |  |  |  |  |  |  |
| $16-9 | | 12 | 6 | 23.37% | 52362.4 | 8.6 | tr\|Q5EFE5\|Q5EFE5_HUMAN Anti-RhD monoclonal T125 gamma1 heavy chain OS=Homo sapiens PE=2 SV=1 |  |  |  |  |  |  |  |  |
| $16-10 | | 12 | 6 | 23.52% | 51724.5 | 8.1 | tr\|Q6N089\|Q6N089_HUMAN Putative uncharacterized protein DKFZp686P15220 OS=Homo sapiens GN=DKFZp686P15220 PE=1 SV=1 |  |  |  |  |  |  |  |  |
| $16-11 | | 12 | 6 | 23.82% | 50870.5 | 7.9 | tr\|S6B291\|S6B291_HUMAN IgG H chain OS=Homo sapiens PE=1 SV=1 |  |  |  |  |  |  |  |  |
| $16-12 | | 12 | 6 | 23.62% | 51715.5 | 7.9 | tr\|V9HW68\|V9HW68_HUMAN Epididymis luminal protein 214 OS=Homo sapiens GN=HEL-214 PE=2 SV=1 |  |  |  |  |  |  |  |  |
| $16-13 | | 12 | 6 | 31.90% | 38162.1 | 8.3 | tr\|Q6PYX1\|Q6PYX1_HUMAN Hepatitis B virus receptor binding protein (Fragment) OS=Homo sapiens PE=1 SV=1 |  |  |  |  |  |  |  |  |
| $16-14 | | 12 | 6 | 23.03% | 52852 | 8.8 | tr\|Q7Z351\|Q7Z351_HUMAN Putative uncharacterized protein DKFZp686N02209 OS=Homo sapiens GN=DKFZp686N02209 PE=2 SV=1 |  |  |  |  |  |  |  |  |
| $16-15 | | 12 | 6 | 23.12% | 52612.6 | 8.5 | tr\|Q6N094\|Q6N094_HUMAN Putative uncharacterized protein DKFZp686O01196 OS=Homo sapiens GN=DKFZp686O01196 PE=2 SV=1 |  |  |  |  |  |  |  |  |
| $16-16 | | 12 | 6 | 23.92% | 50822.6 | 8.5 | tr\|A0A087X010\|A0A087X010_HUMAN Ig gamma-1 chain C region OS=Homo sapiens GN=IGHG1 PE=1 SV=1 |  |  |  |  |  |  |  |  |
| $16-17 | | 12 | 6 | 23.37% | 52109.9 | 7.5 | tr\|A0A087WYE1\|A0A087WYE1_HUMAN Ig gamma-1 chain C region OS=Homo sapiens GN=IGHG1 PE=1 SV=1 |  |  |  |  |  |  |  |  |
| $16-18 | | 12 | 6 | 23.37% | 52042.9 | 8.3 | tr\|Q6MZQ6\|Q6MZQ6_HUMAN Putative uncharacterized protein DKFZp686G11190 OS=Homo sapiens GN=DKFZp686G11190 PE=2 SV=1 |  |  |  |  |  |  |  |  |
| $16-19 | | 12 | 6 | 23.08% | 52758.8 | 8.8 | tr\|Q6N097\|Q6N097_HUMAN Putative uncharacterized protein DKFZp686H20196 OS=Homo sapiens GN=DKFZp686H20196 PE=2 SV=1 |  |  |  |  |  |  |  |  |
| $16-20 | | 12 | 6 | 23.92% | 50915.6 | 8.1 | tr\|A0A0G2JNK4\|A0A0G2JNK4_HUMAN Ig gamma-1 chain C region OS=Homo sapiens GN=IGHG1 PE=1 SV=1 |  |  |  |  |  |  |  |  |
| $16-21 | | 12 | 6 | 23.82% | 50926.6 | 8.3 | tr\|Q6N096\|Q6N096_HUMAN Putative uncharacterized protein DKFZp686I15196 OS=Homo sapiens GN=DKFZp686I15196 PE=2 SV=1 |  |  |  |  |  |  |  |  |
| $16-22 | | 12 | 6 | 23.47% | 52121 | 7.5 | tr\|Q6MZV7\|Q6MZV7_HUMAN Putative uncharacterized protein DKFZp686C11235 OS=Homo sapiens GN=DKFZp686C11235 PE=2 SV=1 |  |  |  |  |  |  |  |  |
|  | R16062_1_CTGF,13134 | K.FNWYVDGVEVHNAK.T | 1678.8284 | 0.7944 | 2 | 1 | 2.4699 | 0.3558 | 823.4 | 1 | 14\|26 | 5.32 | 1 | 22 |  |
|  | R16062_1_CTGF,15825 | K.GFYPSDIAVEWESNGQPENNYK.T | 2545.6579 | -0.5081 | 2 | 1 | 3.3031 | 0.4501 | 953.6 | 1 | 18\|42 | 4 | 2 | 24 |  |
|  | R16062_1_CTGF,15834 | K.GFYPSDIAVEWESNGQPENNYK.T | 2545.6579 | 0.2199 | 2 | 1 | 3.174 | 0.4404 | 1392.9 | 1 | 22\|42 | 4 | 2 | 24 |  |
|  | R16062_1_CTGF,11202 | K.STSGGTAALGCLVK.D | 1322.484 | 1.915 | 2 | 1 | 2.5835 | 0.398 | 756.3 | 1 | 15\|26 | 7.94 | 2 | 29 |  |
|  | R16062_1_CTGF,16618 | K.THTCPPCPAPELLGGPSVFLFPPKPK.D | 2846.3022 | 1.2782 | 3 | 1 | 4.2852 | 0.5532 | 1001.5 | 1 | 29\|100 | 7.76 | 1 | 22 |  |
|  | R16062_1_CTGF,16637 | K.THTCPPCPAPELLGGPSVFLFPPKPK.D | 2846.3022 | 1.7062 | 3 | 1 | 4.105 | 0.4982 | 951.1 | 1 | 30\|100 | 7.76 | 1 | 22 |  |
|  | R16062_1_CTGF,13065 | R.TPEVTCVVVDVSHEDPEVK.F | 2140.3272 | -0.4638 | 2 | 1 | 2.9304 | 0.4179 | 608.6 | 1 | 16\|36 | 4.17 | 1 | 22 |  |
|  | R16062_1_CTGF,13084 | R.TPEVTCVVVDVSHEDPEVK.F | 2140.3272 | 1.0122 | 2 | 1 | 3.3328 | 0.4776 | 1204.2 | 1 | 19\|36 | 4.17 | 1 | 22 |  |
|  | R16062_1_CTGF,13096 | R.TPEVTCVVVDVSHEDPEVK.F | 2140.3272 | -0.1928 | 2 | 1 | 2.9164 | 0.4846 | 723.4 | 1 | 19\|36 | 4.17 | 1 | 22 |  |
|  | R16062_1_CTGF,13155 | R.TPEVTCVVVDVSHEDPEVK.F | 2140.3272 | 0.8702 | 2 | 1 | 2.8213 | 0.5425 | 1108.6 | 1 | 18\|36 | 4.17 | 1 | 22 |  |
|  | R16062_1_CTGF,18198 - 18199 | R.VVSVLTVLHQDWLNGK.E | 1809.1018 | -0.6892 | 2 | 1 | 3.2713 | 0.5131 | 607.2 | 1 | 13\|30 | 6.71 | 3 | 31 |  |
|  | R16062_1_CTGF,18208 | R.VVSVLTVLHQDWLNGK.E | 1809.1018 | 0.3478 | 2 | 1 | 2.4351 | 0.3916 | 1185.7 | 1 | 18\|30 | 6.71 | 3 | 31 |  |
| $17-1 | | 12 | 6 | 24.57% | 38714.2 | 6.6 | sp\|P04083\|ANXA1_HUMAN Annexin A1 OS=Homo sapiens GN=ANXA1 PE=1 SV=2 |  |  |  |  |  |  |  |  |
| $17-2 | | 12 | 6 | 24.57% | 38714.2 | 6.6 | tr\|Q5TZZ9\|Q5TZZ9_HUMAN Annexin OS=Homo sapiens GN=ANXA1 PE=2 SV=1 |  |  |  |  |  |  |  |  |
|  | R16062_1_CTGF,19203 | K.GLGTDEDTLIEILASR.T | 1703.8728 | 1.3018 | 2 | 1 | 2.3358 | 0.3497 | 983.3 | 1 | 16\|30 | 3.92 | 1 | 2 |  |
|  | R16062_1_CTGF,19213 | K.GLGTDEDTLIEILASR.T | 1703.8728 | 0.5398 | 2 | 1 | 2.7695 | 0.3203 | 918.4 | 1 | 15\|30 | 3.92 | 1 | 2 |  |
|  | R16062_1_CTGF,16667 | K.GTDVNVFNTILTTR.S | 1551.7263 | 0.3843 | 2 | 1 | 3.0054 | 0.4393 | 735.3 | 1 | 15\|26 | 5.84 | 1 | 2 |  |
|  | R16062_1_CTGF,18799 | K.GVDEATIIDILTK.R | 1388.5889 | 0.7949 | 2 | 1 | 2.6143 | 0.4543 | 885.6 | 1 | 15\|24 | 4.03 | 1 | 2 |  |
|  | R16062_1_CTGF,18849 | K.GVDEATIIDILTK.R | 1388.5889 | 0.5839 | 2 | 1 | 2.3451 | 0.2216 | 413 | 1 | 11\|24 | 4.03 | 1 | 2 |  |
|  | R16062_1_CTGF,16340 | K.M*YGISLCQAILDETK.G | 1759.0084 | 0.3534 | 2 | 1 | 2.7157 | 0.5001 | 567.2 | 1 | 14\|28 | 4.37 | 1 | 2 |  |
|  | R16062_1_CTGF,10378 | K.TPAQFDADELR.A | 1263.3385 | 0.1905 | 2 | 1 | 2.8954 | 0.3859 | 1112.1 | 1 | 15\|20 | 4.03 | 1 | 2 |  |
|  | R16062_1_CTGF,10452 | K.TPAQFDADELR.A | 1263.3385 | 1.4905 | 2 | 1 | 2.6701 | 0.2389 | 1166.7 | 1 | 15\|20 | 4.03 | 1 | 2 |  |
|  | R16062_1_CTGF,11225 | R.SEDFGVNEDLADSDAR.A | 1740.7205 | 0.3535 | 2 | 1 | 3.2699 | 0.5548 | 1445.3 | 1 | 20\|30 | 3.66 | 1 | 2 |  |
|  | R16062_1_CTGF,11237 | R.SEDFGVNEDLADSDAR.A | 1740.7205 | -1.6575 | 2 | 1 | 2.4066 | 0.3565 | 657.5 | 1 | 14\|30 | 3.66 | 1 | 2 |  |
|  | R16062_1_CTGF,11255 | R.SEDFGVNEDLADSDAR.A | 1740.7205 | 0.5345 | 2 | 1 | 2.7966 | 0.5113 | 847.7 | 1 | 17\|30 | 3.66 | 1 | 2 |  |
|  | R16062_1_CTGF,11272 | R.SEDFGVNEDLADSDAR.A | 1740.7205 | 0.5895 | 2 | 1 | 2.8906 | 0.1912 | 1359 | 1 | 19\|30 | 3.66 | 1 | 2 |  |
| $18-1 | | 12 | 6 | 72.38% | 11446 | 6.2 | tr\|Q6J1Z7\|Q6J1Z7_HUMAN Hemoglobin beta (Fragment) OS=Homo sapiens GN=HBB PE=3 SV=1 |  |  |  |  |  |  |  |  |
|  | R16062_1_CTGF,9579 | K.LHVDPENFR.L | 1127.2347 | -1.7913 | 2 | 1 | 2.3337 | 0.2283 | 566.3 | 1 | 11\|16 | 5.32 | 4 | 6 |  |
|  | R16062_1_CTGF,12466 | K.SAVTALWGK.V | 933.0864 | 1.0334 | 2 | 1 | 2.5672 | 0.4622 | 944.5 | 1 | 13\|16 | 8.47 | 3 | 4 |  |
|  | R16062_1_CTGF,14848 | K.VLGAFSDGLAHLDNLK.G | 1670.8908 | -1.0252 | 2 | 1 | 2.5143 | 0.1645 | 646 | 1 | 17\|30 | 5.21 | 3 | 5 |  |
|  | R16062_1_CTGF,15391 | K.VLGAFSDGLAHLDNLK.G | 1670.8908 | -2.9452 | 2 | 1 | 2.625 | 0.1512 | 871.8 | 1 | 16\|30 | 5.21 | 3 | 5 |  |
|  | R16062_1_CTGF,10117 | K.VNVDAVGGEALGR.L | 1257.3787 | 0.1147 | 2 | 1 | 3.1979 | 0.4818 | 1227.2 | 1 | 16\|24 | 4.37 | 2 | 3 |  |
|  | R16062_1_CTGF,10214 | K.VNVDAVGGEALGR.L | 1257.3787 | 1.1207 | 2 | 1 | 2.7504 | 0.4885 | 1364 | 1 | 19\|24 | 4.37 | 2 | 3 |  |
|  | R16062_1_CTGF,10239 | K.VNVDAVGGEALGR.L | 1257.3787 | 1.2887 | 2 | 1 | 2.5084 | 0.4745 | 1005 | 1 | 16\|24 | 4.37 | 2 | 3 |  |
|  | R16062_1_CTGF,14767 | R.FFESFGDLSTPDAVM*GNPK.V | 2076.2717 | 0.0897 | 2 | 1 | 2.499 | 0.1744 | 609.8 | 1 | 16\|36 | 4.03 | 3 | 4 |  |
|  | R16062_1_CTGF,15229 | R.FFESFGDLSTPDAVM*GNPK.V | 2076.2717 | 0.5327 | 2 | 1 | 2.2769 | 0.2597 | 260.2 | 1 | 11\|36 | 4.03 | 3 | 4 |  |
|  | R16062_1_CTGF,15300 | R.FFESFGDLSTPDAVM*GNPK.V | 2076.2717 | -0.3443 | 2 | 1 | 2.4545 | 0.1603 | 347.6 | 1 | 14\|36 | 4.03 | 3 | 4 |  |
|  | R16062_1_CTGF,15677 | R.LLVVYPWTQR.F | 1275.5235 | 1.0095 | 2 | 1 | 2.2141 | 0.3369 | 1095.1 | 1 | 15\|18 | 8.75 | 4 | 6 |  |
|  | R16062_1_CTGF,15820 | R.LLVVYPWTQR.F | 1275.5235 | -2.2995 | 2 | 1 | 2.5806 | 0.2315 | 710.6 | 1 | 12\|18 | 8.75 | 4 | 6 |  |
| $19-1 | | 11 | 6 | 9.59% | 92469 | 4.8 | sp\|P14625\|ENPL_HUMAN Endoplasmin OS=Homo sapiens GN=HSP90B1 PE=1 SV=1 |  |  |  |  |  |  |  |  |
| $19-2 | | 11 | 6 | 9.59% | 92469 | 4.8 | tr\|V9HWP2\|V9HWP2_HUMAN Epididymis luminal protein 35 OS=Homo sapiens GN=HEL-S-125m PE=2 SV=1 |  |  |  |  |  |  |  |  |
| $19-3 | | 11 | 6 | 9.60% | 92339.9 | 4.8 | tr\|Q5CAQ5\|Q5CAQ5_HUMAN Tumor rejection antigen (Gp96) 1 OS=Homo sapiens GN=TRA1 PE=2 SV=1 |  |  |  |  |  |  |  |  |
|  | R16062_1_CTGF,8116 | K.DISTNYYASQK.K | 1290.3607 | 0.1747 | 2 | 1 | 2.3625 | 0.3287 | 1157.5 | 1 | 15\|20 | 5.83 | 1 | 3 |  |
|  | R16062_1_CTGF,12279 | K.GVVDSDDLPLNVSR.E | 1486.6095 | -2.1455 | 2 | 1 | 2.3793 | 0.2771 | 596.4 | 1 | 15\|26 | 3.93 | 1 | 3 |  |
|  | R16062_1_CTGF,13510 | K.SILFVPTSAPR.G | 1188.4011 | 0.7051 | 2 | 1 | 3.1306 | 0.3608 | 794.7 | 1 | 15\|20 | 9.47 | 1 | 3 |  |
|  | R16062_1_CTGF,13521 | K.SILFVPTSAPR.G | 1188.4011 | 0.9411 | 2 | 1 | 2.7435 | 0.2661 | 1220.9 | 1 | 19\|20 | 9.47 | 1 | 3 |  |
|  | R16062_1_CTGF,10101 - 10102 | R.ELISNASDALDK.I | 1276.3755 | 0.3325 | 2 | 1 | 2.4943 | 0.3724 | 667.3 | 1 | 13\|22 | 4.03 | 2 | 7 |  |
|  | R16062_1_CTGF,10115 | R.ELISNASDALDK.I | 1276.3755 | 0.8545 | 2 | 1 | 2.6074 | 0.4474 | 706.3 | 1 | 14\|22 | 4.03 | 2 | 7 |  |
|  | R16062_1_CTGF,11928 | R.FQSSHHPTDITSLDQYVER.M | 2261.3929 | 0.6059 | 3 | 1 | 3.7609 | 0.4736 | 1494.7 | 1 | 29\|72 | 5.21 | 1 | 3 |  |
|  | R16062_1_CTGF,11938 | R.FQSSHHPTDITSLDQYVER.M | 2261.3929 | 0.6459 | 3 | 1 | 3.7697 | 0.4861 | 1510.4 | 1 | 31\|72 | 5.21 | 1 | 3 |  |
|  | R16062_1_CTGF,11765 | R.LSLNIDPDAK.V | 1086.2208 | 0.0878 | 2 | 1 | 2.5838 | 0.3201 | 1178.9 | 1 | 15\|18 | 4.21 | 1 | 3 |  |
|  | R16062_1_CTGF,11795 | R.LSLNIDPDAK.V | 1086.2208 | 1.1908 | 2 | 1 | 2.2258 | 0.3569 | 1206.1 | 1 | 14\|18 | 4.21 | 1 | 3 |  |
|  | R16062_1_CTGF,11807 | R.LSLNIDPDAK.V | 1086.2208 | 1.1028 | 2 | 1 | 2.3325 | 0.2056 | 1068.3 | 1 | 13\|18 | 4.21 | 1 | 3 |  |
| $20-1 | | 18 | 5 | 21.01% | 38429.1 | 6.2 | sp\|P51884\|LUM_HUMAN Lumican OS=Homo sapiens GN=LUM PE=1 SV=2 |  |  |  |  |  |  |  |  |
|  | R16062_1_CTGF,17080 | K.NIPTVNENLENYYLEVNQLEK.F | 2537.7635 | 1.2095 | 2 | 1 | 3.0977 | 0.4583 | 443.5 | 1 | 16\|40 | 4.09 | 1 | 1 |  |
|  | R16062_1_CTGF,17085 | K.NIPTVNENLENYYLEVNQLEK.F | 2537.7635 | 1.0325 | 2 | 1 | 2.8844 | 0.4869 | 241.9 | 1 | 16\|40 | 4.09 | 1 | 1 |  |
|  | R16062_1_CTGF,17184 | K.NIPTVNENLENYYLEVNQLEK.F | 2537.7635 | 0.7555 | 3 | 1 | 4.4013 | 0.3993 | 1155.1 | 1 | 31\|80 | 4.09 | 1 | 1 |  |
|  | R16062_1_CTGF,17245 | K.NIPTVNENLENYYLEVNQLEK.F | 2537.7635 | 2.0455 | 2 | 1 | 2.8244 | 0.4593 | 351.4 | 1 | 15\|40 | 4.09 | 1 | 1 |  |
|  | R16062_1_CTGF,17217 | K.SLEYLDLSFNQIAR.L | 1669.8597 | -0.6453 | 2 | 1 | 3.373 | 0.4191 | 856.4 | 1 | 16\|26 | 4.37 | 1 | 1 |  |
|  | R16062_1_CTGF,17228 | K.SLEYLDLSFNQIAR.L | 1669.8597 | -0.6403 | 2 | 1 | 3.8309 | 0.3193 | 1030.4 | 1 | 16\|26 | 4.37 | 1 | 1 |  |
|  | R16062_1_CTGF,17316 | K.SLEYLDLSFNQIAR.L | 1669.8597 | 0.7757 | 2 | 1 | 2.5895 | 0.4046 | 617 | 1 | 15\|26 | 4.37 | 1 | 1 |  |
|  | R16062_1_CTGF,17338 | K.SLEYLDLSFNQIAR.L | 1669.8597 | 1.2347 | 2 | 1 | 2.4204 | 0.2309 | 698.2 | 1 | 14\|26 | 4.37 | 1 | 1 |  |
|  | R16062_1_CTGF,14729 | R.FNALQYLR.L | 1025.1855 | 0.1305 | 2 | 1 | 2.6164 | 0.3312 | 868.4 | 1 | 13\|14 | 8.75 | 1 | 1 |  |
|  | R16062_1_CTGF,14733 | R.FNALQYLR.L | 1025.1855 | 0.8875 | 2 | 1 | 2.5256 | 0.3123 | 1391.3 | 1 | 13\|14 | 8.75 | 1 | 1 |  |
|  | R16062_1_CTGF,20397 | R.LPSGLPVSLLTLYLDNNK.I | 1958.2874 | 0.7434 | 2 | 1 | 3.2464 | 0.5295 | 552.7 | 1 | 20\|34 | 5.84 | 1 | 1 |  |
|  | R16062_1_CTGF,20442 | R.LPSGLPVSLLTLYLDNNK.I | 1958.2874 | 1.3634 | 2 | 1 | 3.2664 | 0.4863 | 525.8 | 1 | 19\|34 | 5.84 | 1 | 1 |  |
|  | R16062_1_CTGF,20851 | R.LPSGLPVSLLTLYLDNNK.I | 1958.2874 | -0.4456 | 2 | 1 | 3.0595 | 0.4272 | 628.3 | 1 | 20\|34 | 5.84 | 1 | 1 |  |
|  | R16062_1_CTGF,20864 | R.LPSGLPVSLLTLYLDNNK.I | 1958.2874 | 1.2174 | 2 | 1 | 3.4006 | 0.5236 | 998.5 | 1 | 20\|34 | 5.84 | 1 | 1 |  |
|  | R16062_1_CTGF,20870 - 20871 | R.LPSGLPVSLLTLYLDNNK.I | 1958.2874 | 0.4214 | 2 | 1 | 3.5018 | 0.5676 | 877.9 | 1 | 18\|34 | 5.84 | 1 | 1 |  |
|  | R16062_1_CTGF,5790 | R.NNQIDHIDEK.A | 1226.2783 | -0.8277 | 2 | 1 | 2.4197 | 0.2379 | 1043.1 | 1 | 14\|18 | 4.54 | 1 | 1 |  |
|  | R16062_1_CTGF,5803 | R.NNQIDHIDEK.A | 1226.2783 | 0.8073 | 2 | 1 | 2.2376 | 0.3721 | 706.3 | 1 | 13\|18 | 4.54 | 1 | 1 |  |
|  | R16062_1_CTGF,5812 | R.NNQIDHIDEK.A | 1226.2783 | 0.3503 | 2 | 1 | 2.4391 | 0.2248 | 976.6 | 1 | 14\|18 | 4.54 | 1 | 1 |  |
| $21-1 | | 10 | 5 | 40.82% | 16055.4 | 7.8 | sp\|P02042\|HBD_HUMAN Hemoglobin subunit delta OS=Homo sapiens GN=HBD PE=1 SV=2 |  |  |  |  |  |  |  |  |
| $21-2 | | 10 | 5 | 40.82% | 16055.4 | 7.8 | tr\|A0N071\|A0N071_HUMAN Delta globin OS=Homo sapiens GN=HBD PE=3 SV=1 |  |  |  |  |  |  |  |  |
|  | R16062_1_CTGF,9579 | K.LHVDPENFR.L | 1127.2347 | -1.7913 | 2 | 1 | 2.3337 | 0.2283 | 566.3 | 1 | 11\|16 | 5.32 | 4 | 6 |  |
|  | R16062_1_CTGF,14848 | K.VLGAFSDGLAHLDNLK.G | 1670.8908 | -1.0252 | 2 | 1 | 2.5143 | 0.1645 | 646 | 1 | 17\|30 | 5.21 | 3 | 5 |  |
|  | R16062_1_CTGF,15391 | K.VLGAFSDGLAHLDNLK.G | 1670.8908 | -2.9452 | 2 | 1 | 2.625 | 0.1512 | 871.8 | 1 | 16\|30 | 5.21 | 3 | 5 |  |
|  | R16062_1_CTGF,10117 | K.VNVDAVGGEALGR.L | 1257.3787 | 0.1147 | 2 | 1 | 3.1979 | 0.4818 | 1227.2 | 1 | 16\|24 | 4.37 | 2 | 3 |  |
|  | R16062_1_CTGF,10214 | K.VNVDAVGGEALGR.L | 1257.3787 | 1.1207 | 2 | 1 | 2.7504 | 0.4885 | 1364 | 1 | 19\|24 | 4.37 | 2 | 3 |  |
|  | R16062_1_CTGF,10239 | K.VNVDAVGGEALGR.L | 1257.3787 | 1.2887 | 2 | 1 | 2.5084 | 0.4745 | 1005 | 1 | 16\|24 | 4.37 | 2 | 3 |  |
|  | R16062_1_CTGF,8571 | K.VVAGVANALAHK.Y | 1150.3561 | 0.4061 | 2 | 1 | 2.571 | 0.4834 | 838.3 | 1 | 17\|22 | 8.73 | 2 | 4 |  |
|  | R16062_1_CTGF,8582 | K.VVAGVANALAHK.Y | 1150.3561 | 0.2791 | 2 | 1 | 2.9539 | 0.5515 | 834.6 | 1 | 17\|22 | 8.73 | 2 | 4 |  |
|  | R16062_1_CTGF,15677 | R.LLVVYPWTQR.F | 1275.5235 | 1.0095 | 2 | 1 | 2.2141 | 0.3369 | 1095.1 | 1 | 15\|18 | 8.75 | 4 | 6 |  |
|  | R16062_1_CTGF,15820 | R.LLVVYPWTQR.F | 1275.5235 | -2.2995 | 2 | 1 | 2.5806 | 0.2315 | 710.6 | 1 | 12\|18 | 8.75 | 4 | 6 |  |
| $22-1 | | 9 | 5 | 12.23% | 72332.9 | 5.1 | sp\|P11021\|GRP78_HUMAN 78 kDa glucose-regulated protein OS=Homo sapiens GN=HSPA5 PE=1 SV=2 |  |  |  |  |  |  |  |  |
| $22-2 | | 9 | 5 | 12.23% | 72332.9 | 5.1 | tr\|V9HWB4\|V9HWB4_HUMAN Epididymis secretory sperm binding protein Li 89n OS=Homo sapiens GN=HEL-S-89n PE=2 SV=1 |  |  |  |  |  |  |  |  |
|  | R16062_1_CTGF,10657 | K.LYGSAGPPPTGEEDTAEKDEL.- | 2177.2646 | -0.3064 | 2 | 1 | 2.502 | 0.5127 | 644.9 | 1 | 16\|40 | 3.77 | 1 | 2 |  |
|  | R16062_1_CTGF,10665 | K.LYGSAGPPPTGEEDTAEKDEL.- | 2177.2646 | 0.6876 | 2 | 1 | 2.3555 | 0.4619 | 453.6 | 1 | 16\|40 | 3.77 | 1 | 2 |  |
|  | R16062_1_CTGF,11127 | K.NQLTSNPENTVFDAK.R | 1678.7819 | 0.2099 | 2 | 1 | 2.4898 | 0.2207 | 772.9 | 1 | 16\|28 | 4.37 | 1 | 2 |  |
|  | R16062_1_CTGF,11160 | K.NQLTSNPENTVFDAK.R | 1678.7819 | 1.3329 | 2 | 1 | 2.4261 | 0.1493 | 787.1 | 1 | 16\|28 | 4.37 | 1 | 2 |  |
|  | R16062_1_CTGF,11516 | K.SQIFSTASDNQPTVTIK.V | 1838.0088 | 0.5408 | 2 | 1 | 2.7508 | 0.3975 | 1132.4 | 1 | 20\|32 | 5.55 | 1 | 2 |  |
|  | R16062_1_CTGF,11520 | K.SQIFSTASDNQPTVTIK.V | 1838.0088 | 0.7868 | 2 | 1 | 3.0525 | 0.4291 | 1079.9 | 1 | 18\|32 | 5.55 | 1 | 2 |  |
|  | R16062_1_CTGF,14434 - 14435 | R.IINEPTAAAIAYGLDK.R | 1660.8927 | 0.2447 | 2 | 1 | 3.7359 | 0.5046 | 850.3 | 1 | 19\|30 | 4.37 | 2 | 7 |  |
|  | R16062_1_CTGF,14445 | R.IINEPTAAAIAYGLDK.R | 1660.8927 | 0.5087 | 2 | 1 | 3.3099 | 0.4476 | 1154.2 | 1 | 19\|30 | 4.37 | 2 | 7 |  |
|  | R16062_1_CTGF,12898 | R.NELESYAYSLK.N | 1317.4261 | 0.6331 | 2 | 1 | 2.3365 | 0.2278 | 660.7 | 1 | 12\|20 | 4.53 | 1 | 2 |  |
| $23-1 | | 8 | 5 | 12.86% | 49955.6 | 5.4 | sp\|P19971\|TYPH_HUMAN Thymidine phosphorylase OS=Homo sapiens GN=TYMP PE=1 SV=2 |  |  |  |  |  |  |  |  |
| $23-2 | | 8 | 5 | 12.86% | 49955.6 | 5.4 | tr\|E5KRG5\|E5KRG5_HUMAN Thymidine phosphorylase OS=Homo sapiens GN=hCG_1988078 PE=3 SV=1 |  |  |  |  |  |  |  |  |
| $23-3 | | 8 | 5 | 12.86% | 49923.6 | 5.4 | tr\|B2RBL3\|B2RBL3_HUMAN Thymidine phosphorylase OS=Homo sapiens PE=2 SV=1 |  |  |  |  |  |  |  |  |
| $23-4 | | 8 | 5 | 13.93% | 46087.2 | 5.4 | tr\|C9JGI3\|C9JGI3_HUMAN Thymidine phosphorylase (Fragment) OS=Homo sapiens GN=TYMP PE=1 SV=1 |  |  |  |  |  |  |  |  |
|  | R16062_1_CTGF,5156 | R.ALCSGSPAER.R | 1048.1261 | 0.5131 | 2 | 1 | 2.3373 | 0.3388 | 601.2 | 1 | 12\|18 | 6.04 | 1 | 4 |  |
|  | R16062_1_CTGF,19630 | R.ALPLALVLHELGAGR.S | 1530.8398 | 0.4398 | 2 | 1 | 2.6947 | 0.4737 | 692.6 | 1 | 16\|28 | 6.79 | 1 | 4 |  |
|  | R16062_1_CTGF,19672 | R.ALPLALVLHELGAGR.S | 1530.8398 | 0.3218 | 2 | 1 | 2.576 | 0.5007 | 823.4 | 1 | 19\|28 | 6.79 | 1 | 4 |  |
|  | R16062_1_CTGF,11904 | R.ALQEALVLSDR.A | 1215.3817 | 0.1647 | 2 | 1 | 2.7081 | 0.4328 | 1007 | 1 | 14\|20 | 4.37 | 1 | 4 |  |
|  | R16062_1_CTGF,11915 | R.ALQEALVLSDR.A | 1215.3817 | 0.5067 | 2 | 1 | 2.8875 | 0.4668 | 1194.4 | 1 | 16\|20 | 4.37 | 1 | 4 |  |
|  | R16062_1_CTGF,9396 | R.M*LAAQGVDPGLAR.A | 1315.5247 | 0.3087 | 2 | 1 | 2.666 | 0.3384 | 922.1 | 1 | 15\|24 | 5.59 | 1 | 4 |  |
|  | R16062_1_CTGF,9280 | R.VAAALDDGSALGR.F | 1216.3265 | 0.4215 | 2 | 1 | 2.6041 | 0.3827 | 1226.6 | 1 | 16\|24 | 4.21 | 1 | 4 |  |
|  | R16062_1_CTGF,9291 | R.VAAALDDGSALGR.F | 1216.3265 | 0.4655 | 2 | 1 | 2.3588 | 0.3092 | 1058 | 1 | 17\|24 | 4.21 | 1 | 4 |  |
| $24-1 | | 7 | 5 | 4.06% | 193516 | 5.2 | sp\|Q05707\|COEA1_HUMAN Collagen alpha-1(XIV) chain OS=Homo sapiens GN=COL14A1 PE=1 SV=3 |  |  |  |  |  |  |  |  |
| $24-2 | | 7 | 5 | 6.61% | 122174 | 5.1 | tr\|A8KAL5\|A8KAL5_HUMAN cDNA FLJ77224, highly similar to Homo sapiens collagen, type XIV, alpha 1 (undulin) (COL14A1), mRNA (Fragment) OS=Homo sapiens PE=2 SV=1 |  |  |  |  |  |  |  |  |
|  | R16062_1_CTGF,13482 | K.ASAHAITGPPTELITSEVTAR.S | 2123.3528 | 1.6048 | 3 | 1 | 4.1001 | 0.4679 | 1837.7 | 1 | 34\|80 | 5.4 | 1 | 2 |  |
|  | R16062_1_CTGF,14670 | K.TLFLGVTNLQAK.H | 1305.548 | 2.09 | 2 | 1 | 2.5335 | 0.2036 | 1194.9 | 1 | 15\|22 | 8.41 | 1 | 2 |  |
|  | R16062_1_CTGF,7554 | K.TNQLNLQNTATK.A | 1346.4722 | 0.5622 | 2 | 1 | 2.6635 | 0.4496 | 503.8 | 1 | 12\|22 | 8.41 | 1 | 2 |  |
|  | R16062_1_CTGF,7562 | K.TNQLNLQNTATK.A | 1346.4722 | 0.3512 | 2 | 1 | 2.5638 | 0.4302 | 419.5 | 1 | 13\|22 | 8.41 | 1 | 2 |  |
|  | R16062_1_CTGF,15365 | R.ESGVELFAIGVK.N | 1249.438 | 0.243 | 2 | 1 | 2.2651 | 0.4212 | 951.3 | 1 | 14\|22 | 4.53 | 1 | 2 |  |
|  | R16062_1_CTGF,17944 | R.HFLENLVTAFDVGSEK.T | 1806.9964 | 0.5254 | 2 | 1 | 3.4112 | 0.5091 | 1621.7 | 1 | 20\|30 | 4.65 | 1 | 2 |  |
|  | R16062_1_CTGF,17959 | R.HFLENLVTAFDVGSEK.T | 1806.9964 | 1.4694 | 2 | 1 | 3.4643 | 0.5571 | 2019.7 | 1 | 21\|30 | 4.65 | 1 | 2 |  |
| $25-1 | | 6 | 5 | 20.06% | 39746.7 | 8.8 | sp\|P07585\|PGS2_HUMAN Decorin OS=Homo sapiens GN=DCN PE=1 SV=1 |  |  |  |  |  |  |  |  |
| $25-2 | | 6 | 5 | 20.06% | 39746.7 | 8.8 | tr\|Q6FH10\|Q6FH10_HUMAN DCN protein OS=Homo sapiens GN=DCN PE=2 SV=1 |  |  |  |  |  |  |  |  |
|  | R16062_1_CTGF,19719 | K.ASYSGVSLFSNPVQYWEIQPSTFR.C | 2765.0282 | 1.3082 | 2 | 1 | 2.5831 | 0.5274 | 432 | 1 | 15\|46 | 6.05 | 1 | 2 |  |
|  | R16062_1_CTGF,14126 | K.DLPPDTTLLDLQNNK.I | 1697.8682 | -1.5058 | 2 | 1 | 3.5891 | 0.3884 | 469.8 | 1 | 18\|28 | 3.93 | 1 | 2 |  |
|  | R16062_1_CTGF,15226 | R.DFEPSLGPVCPFR.C | 1521.6922 | 0.4592 | 2 | 1 | 2.287 | 0.3531 | 357.7 | 1 | 14\|24 | 4.37 | 1 | 2 |  |
|  | R16062_1_CTGF,5121 | R.VPGGLAEHK.Y | 908.0369 | -0.9211 | 2 | 1 | 2.2347 | 0.3079 | 387.2 | 1 | 14\|16 | 6.72 | 1 | 2 |  |
|  | R16062_1_CTGF,8826 | R.VVQCSDLGLDK.V | 1234.3752 | 0.4892 | 2 | 1 | 2.5569 | 0.4436 | 693.7 | 1 | 15\|20 | 4.21 | 1 | 2 |  |
|  | R16062_1_CTGF,8837 | R.VVQCSDLGLDK.V | 1234.3752 | 0.5992 | 2 | 1 | 2.3551 | 0.464 | 902.3 | 1 | 16\|20 | 4.21 | 1 | 2 |  |
| $26-1 | | 6 | 5 | 12.67% | 56782.5 | 6 | sp\|P30101\|PDIA3_HUMAN Protein disulfide-isomerase A3 OS=Homo sapiens GN=PDIA3 PE=1 SV=4 |  |  |  |  |  |  |  |  |
| $26-2 | | 6 | 5 | 12.67% | 56782.5 | 6 | tr\|V9HVY3\|V9HVY3_HUMAN Protein disulfide-isomerase OS=Homo sapiens GN=HEL-S-269 PE=2 SV=1 |  |  |  |  |  |  |  |  |
| $26-3 | | 6 | 5 | 13.33% | 54102.6 | 6.8 | tr\|B3KQT9\|B3KQT9_HUMAN Protein disulfide-isomerase OS=Homo sapiens PE=2 SV=1 |  |  |  |  |  |  |  |  |
|  | R16062_1_CTGF,10031 | K.LNFAVASR.K | 878.0108 | 0.4988 | 2 | 1 | 2.2478 | 0.2486 | 557.8 | 2 | 10\|14 | 9.75 | 1 | 3 |  |
|  | R16062_1_CTGF,17239 | K.TFSHELSDFGLESTAGEIPVVAIR.T | 2576.8428 | -0.0482 | 3 | 1 | 3.9419 | 0.4719 | 824.5 | 1 | 31\|92 | 4.4 | 1 | 3 |  |
|  | R16062_1_CTGF,10494 | K.YGVSGYPTLK.I | 1085.2345 | -0.3535 | 2 | 1 | 2.4437 | 0.3897 | 631 | 1 | 13\|18 | 8.5 | 2 | 4 |  |
|  | R16062_1_CTGF,17107 | R.ELSDFISYLQR.E | 1371.5201 | 1.2941 | 2 | 1 | 2.289 | 0.438 | 623.1 | 1 | 13\|20 | 4.37 | 1 | 3 |  |
|  | R16062_1_CTGF,14766 | R.FLQDYFDGNLK.R | 1360.4958 | 1.1148 | 2 | 1 | 2.4788 | 0.1768 | 834.3 | 1 | 15\|20 | 4.21 | 1 | 3 |  |
|  | R16062_1_CTGF,14777 | R.FLQDYFDGNLK.R | 1360.4958 | -0.7992 | 2 | 1 | 2.229 | 0.2522 | 941.6 | 1 | 15\|20 | 4.21 | 1 | 3 |  |
| $27-1 | | 6 | 5 | 12.63% | 61963.2 | 6 | sp\|Q14195\|DPYL3_HUMAN Dihydropyrimidinase-related protein 3 OS=Homo sapiens GN=DPYSL3 PE=1 SV=1 |  |  |  |  |  |  |  |  |
| $27-2 | | 6 | 5 | 10.53% | 73910.3 | 6.1 | tr\|Q6DEN2\|Q6DEN2_HUMAN DPYSL3 protein OS=Homo sapiens GN=DPYSL3 PE=2 SV=1 |  |  |  |  |  |  |  |  |
| $27-3 | | 6 | 5 | 12.63% | 61979.2 | 6 | tr\|Q8IXW6\|Q8IXW6_HUMAN Dihydropyrimidinase-like 3 OS=Homo sapiens GN=DPYSL3 PE=1 SV=1 |  |  |  |  |  |  |  |  |
|  | R16062_1_CTGF,12335 | K.QIGDNLIVPGGVK.T | 1310.5246 | 0.7176 | 2 | 1 | 2.5938 | 0.4426 | 445.2 | 2 | 12\|24 | 5.84 | 1 | 3 |  |
|  | R16062_1_CTGF,9371 | K.SAADLISQAR.K | 1032.1332 | 0.6122 | 2 | 1 | 3.0491 | 0.3414 | 901.7 | 1 | 14\|18 | 5.55 | 1 | 3 |  |
|  | R16062_1_CTGF,11898 | R.AITIASQTNCPLYVTK.V | 1781.0369 | 0.4589 | 2 | 1 | 2.5028 | 0.3501 | 577 | 1 | 13\|30 | 8.24 | 1 | 3 |  |
|  | R16062_1_CTGF,11906 | R.AITIASQTNCPLYVTK.V | 1781.0369 | 0.4429 | 2 | 1 | 2.2657 | 0.4599 | 405.5 | 1 | 12\|30 | 8.24 | 1 | 3 |  |
|  | R16062_1_CTGF,14020 | R.GM*YDGPVFDLTTTPK.G | 1658.8539 | 0.6749 | 2 | 1 | 2.4424 | 0.403 | 613 | 1 | 14\|28 | 4.21 | 1 | 3 |  |
|  | R16062_1_CTGF,16331 | R.ISVGSDSDLVIWDPDAVK.I | 1917.1059 | -0.6881 | 2 | 1 | 2.5868 | 0.3801 | 472.3 | 1 | 13\|34 | 3.77 | 1 | 3 |  |
| $28-1 | | 14 | 4 | 12.47% | 42009 | 5.2 | sp\|P62736\|ACTA_HUMAN Actin, aortic smooth muscle OS=Homo sapiens GN=ACTA2 PE=1 SV=1 |  |  |  |  |  |  |  |  |
| $28-2 | | 14 | 4 | 12.47% | 42019 | 5.2 | sp\|P68032\|ACTC_HUMAN Actin, alpha cardiac muscle 1 OS=Homo sapiens GN=ACTC1 PE=1 SV=1 |  |  |  |  |  |  |  |  |
| $28-3 | | 14 | 4 | 12.50% | 41876.9 | 5.3 | sp\|P63267\|ACTH_HUMAN Actin, gamma-enteric smooth muscle OS=Homo sapiens GN=ACTG2 PE=1 SV=1 |  |  |  |  |  |  |  |  |
| $28-4 | | 14 | 4 | 12.47% | 42051.1 | 5.2 | sp\|P68133\|ACTS_HUMAN Actin, alpha skeletal muscle OS=Homo sapiens GN=ACTA1 PE=1 SV=1 |  |  |  |  |  |  |  |  |
| $28-5 | | 14 | 4 | 12.47% | 42009 | 5.2 | tr\|D2JYH4\|D2JYH4_HUMAN Actin, alpha 2, smooth muscle, aorta OS=Homo sapiens GN=ACTA2 PE=3 SV=1 |  |  |  |  |  |  |  |  |
| $28-6 | | 14 | 4 | 12.47% | 42047 | 5.2 | tr\|A8K3K1\|A8K3K1_HUMAN cDNA FLJ78096, highly similar to Homo sapiens actin, alpha, cardiac muscle (ACTC), mRNA OS=Homo sapiens PE=2 SV=1 |  |  |  |  |  |  |  |  |
| $28-7 | | 14 | 4 | 12.50% | 41816.9 | 5.3 | tr\|B3KW67\|B3KW67_HUMAN cDNA FLJ42347 fis, clone UTERU2003399, highly similar to Actin, gamma-enteric smooth muscle OS=Homo sapiens PE=2 SV=1 |  |  |  |  |  |  |  |  |
| $28-8 | | 14 | 4 | 13.74% | 37824.4 | 5.5 | tr\|B3KUD3\|B3KUD3_HUMAN cDNA FLJ39583 fis, clone SKMUS2004897, highly similar to ACTIN, ALPHA SKELETAL MUSCLE OS=Homo sapiens PE=2 SV=1 |  |  |  |  |  |  |  |  |
| $28-9 | | 14 | 4 | 13.70% | 38579.1 | 5.2 | tr\|B7Z6P1\|B7Z6P1_HUMAN cDNA FLJ53662, highly similar to Actin, alpha skeletal muscle OS=Homo sapiens PE=2 SV=1 |  |  |  |  |  |  |  |  |
|  | R16062_1_CTGF,6607 | K.AGFAGDDAPR.A | 977.013 | 1.549 | 2 | 1 | 2.2568 | 0.2115 | 1142.9 | 1 | 15\|18 | 4.21 | 2 | 19 |  |
|  | R16062_1_CTGF,6944 | K.AGFAGDDAPR.A | 977.013 | -0.146 | 2 | 1 | 2.201 | 0.3391 | 858.1 | 1 | 13\|18 | 4.21 | 2 | 19 |  |
|  | R16062_1_CTGF,5612 | K.DSYVGDEAQSK.R | 1199.2065 | 0.2925 | 2 | 1 | 2.5233 | 0.4763 | 1016.9 | 1 | 17\|20 | 4.03 | 2 | 19 |  |
|  | R16062_1_CTGF,5623 - 5624 | K.DSYVGDEAQSK.R | 1199.2065 | -0.0155 | 2 | 1 | 2.7277 | 0.3658 | 792.8 | 1 | 18\|20 | 4.03 | 2 | 19 |  |
|  | R16062_1_CTGF,5634 | K.DSYVGDEAQSK.R | 1199.2065 | 0.8525 | 2 | 1 | 2.541 | 0.5368 | 1034.9 | 1 | 17\|20 | 4.03 | 2 | 19 |  |
|  | R16062_1_CTGF,13401 | K.SYELPDGQVITIGNER.F | 1791.9401 | 0.5051 | 2 | 1 | 2.6408 | 0.4404 | 752.6 | 1 | 20\|30 | 4.14 | 2 | 19 |  |
|  | R16062_1_CTGF,13444 | K.SYELPDGQVITIGNER.F | 1791.9401 | 0.5331 | 2 | 1 | 2.4361 | 0.3534 | 789 | 1 | 19\|30 | 4.14 | 2 | 19 |  |
|  | R16062_1_CTGF,14481 | K.SYELPDGQVITIGNER.F | 1791.9401 | 0.0741 | 2 | 1 | 2.5171 | 0.4775 | 1160.3 | 1 | 18\|30 | 4.14 | 2 | 19 |  |
|  | R16062_1_CTGF,14518 | K.SYELPDGQVITIGNER.F | 1791.9401 | 1.4201 | 2 | 1 | 2.9243 | 0.5515 | 897.4 | 1 | 18\|30 | 4.14 | 2 | 19 |  |
|  | R16062_1_CTGF,14541 | K.SYELPDGQVITIGNER.F | 1791.9401 | 1.3251 | 2 | 1 | 2.5249 | 0.5202 | 891.8 | 1 | 18\|30 | 4.14 | 2 | 19 |  |
|  | R16062_1_CTGF,14941 | K.SYELPDGQVITIGNER.F | 1791.9401 | 0.0721 | 2 | 1 | 3.7924 | 0.5219 | 1372.9 | 1 | 21\|30 | 4.14 | 2 | 19 |  |
|  | R16062_1_CTGF,14953 | K.SYELPDGQVITIGNER.F | 1791.9401 | -0.5989 | 2 | 1 | 3.4993 | 0.5054 | 1279 | 1 | 22\|30 | 4.14 | 2 | 19 |  |
|  | R16062_1_CTGF,15011 | K.SYELPDGQVITIGNER.F | 1791.9401 | 1.0781 | 2 | 1 | 2.8991 | 0.3201 | 768.3 | 1 | 18\|30 | 4.14 | 2 | 19 |  |
|  | R16062_1_CTGF,10205 | R.GYSFVTTAER.E | 1131.2201 | 0.2401 | 2 | 1 | 2.2847 | 0.378 | 604.5 | 1 | 12\|18 | 6 | 1 | 9 |  |
| $29-1 | | 9 | 4 | 17.91% | 36053.1 | 8.6 | sp\|P04406\|G3P_HUMAN Glyceraldehyde-3-phosphate dehydrogenase OS=Homo sapiens GN=GAPDH PE=1 SV=3 |  |  |  |  |  |  |  |  |
| $29-2 | | 9 | 4 | 23.08% | 27870.7 | 6.5 | tr\|E7EUT5\|E7EUT5_HUMAN Glyceraldehyde-3-phosphate dehydrogenase OS=Homo sapiens GN=GAPDH PE=1 SV=1 |  |  |  |  |  |  |  |  |
| $29-3 | | 9 | 4 | 17.91% | 36053.1 | 8.6 | tr\|V9HVZ4\|V9HVZ4_HUMAN Glyceraldehyde-3-phosphate dehydrogenase OS=Homo sapiens GN=HEL-S-162eP PE=2 SV=1 |  |  |  |  |  |  |  |  |
| $29-4 | | 9 | 4 | 17.91% | 36049.1 | 8.6 | tr\|Q2TSD0\|Q2TSD0_HUMAN Glyceraldehyde-3-phosphate dehydrogenase OS=Homo sapiens PE=2 SV=1 |  |  |  |  |  |  |  |  |
|  | R16062_1_CTGF,10943 | K.IISNASCTTNCLAPLAK.V | 1835.0796 | -0.2644 | 2 | 1 | 2.2072 | 0.18 | 753.5 | 1 | 17\|32 | 8.06 | 1 | 4 |  |
|  | R16062_1_CTGF,10949 | K.IISNASCTTNCLAPLAK.V | 1835.0796 | 0.3846 | 2 | 1 | 2.3189 | 0.4724 | 1224.2 | 1 | 19\|32 | 8.06 | 1 | 4 |  |
|  | R16062_1_CTGF,10982 | K.IISNASCTTNCLAPLAK.V | 1835.0796 | 1.4586 | 2 | 1 | 2.5436 | 0.4181 | 1258.3 | 1 | 19\|32 | 8.06 | 1 | 4 |  |
|  | R16062_1_CTGF,15399 | K.LISWYDNEFGYSNR.V | 1764.8749 | 0.0769 | 2 | 1 | 2.456 | 0.4118 | 410.9 | 1 | 12\|26 | 4.37 | 1 | 4 |  |
|  | R16062_1_CTGF,15405 | K.LISWYDNEFGYSNR.V | 1764.8749 | 0.9659 | 2 | 1 | 2.7749 | 0.497 | 698.1 | 1 | 13\|26 | 4.37 | 1 | 4 |  |
|  | R16062_1_CTGF,11926 | R.GALQNIIPASTGAAK.A | 1412.6169 | 0.5079 | 2 | 1 | 2.2719 | 0.3724 | 337.3 | 1 | 13\|28 | 8.75 | 1 | 4 |  |
|  | R16062_1_CTGF,11937 | R.GALQNIIPASTGAAK.A | 1412.6169 | 0.3889 | 2 | 1 | 2.6732 | 0.3616 | 485.3 | 1 | 14\|28 | 8.75 | 1 | 4 |  |
|  | R16062_1_CTGF,12247 | R.VPTANVSVVDLTCR.L | 1531.73 | 0.44 | 2 | 1 | 2.7064 | 0.5121 | 1010.9 | 1 | 17\|26 | 5.8 | 1 | 4 |  |
|  | R16062_1_CTGF,12331 | R.VPTANVSVVDLTCR.L | 1531.73 | 2.37 | 2 | 1 | 2.537 | 0.4928 | 623.3 | 1 | 16\|26 | 5.8 | 1 | 4 |  |
| $30-1 | | 8 | 4 | 64.15% | 11608.8 | 5.6 | sp\|P01834\|IGKC_HUMAN Ig kappa chain C region OS=Homo sapiens GN=IGKC PE=1 SV=1 |  |  |  |  |  |  |  |  |
| $30-2 | | 8 | 4 | 29.44% | 25136.1 | 5.9 | tr\|A0A087X130\|A0A087X130_HUMAN Ig kappa chain C region OS=Homo sapiens GN=IGKC PE=1 SV=1 |  |  |  |  |  |  |  |  |
| $30-3 | | 8 | 4 | 29.06% | 25631 | 8.8 | tr\|A0A087WYL9\|A0A087WYL9_HUMAN Ig kappa chain C region OS=Homo sapiens GN=IGKC PE=1 SV=1 |  |  |  |  |  |  |  |  |
| $30-4 | | 8 | 4 | 29.06% | 25697.8 | 8.7 | tr\|Q5EFE6\|Q5EFE6_HUMAN Anti-RhD monoclonal T125 kappa light chain OS=Homo sapiens PE=2 SV=1 |  |  |  |  |  |  |  |  |
| $30-5 | | 8 | 4 | 28.94% | 25645.9 | 6.2 | tr\|V9HW34\|V9HW34_HUMAN Epididymis luminal protein 213 OS=Homo sapiens GN=HEL-213 PE=2 SV=1 |  |  |  |  |  |  |  |  |
| $30-6 | | 8 | 4 | 28.81% | 25807 | 8.2 | tr\|Q6GMX0\|Q6GMX0_HUMAN Uncharacterized protein OS=Homo sapiens PE=1 SV=1 |  |  |  |  |  |  |  |  |
| $30-7 | | 8 | 4 | 28.81% | 25623.9 | 8.2 | tr\|A0A087WTX5\|A0A087WTX5_HUMAN Ig kappa chain C region OS=Homo sapiens GN=IGKC PE=1 SV=1 |  |  |  |  |  |  |  |  |
| $30-8 | | 8 | 4 | 28.81% | 25701.9 | 7.5 | tr\|Q7Z3Y4\|Q7Z3Y4_HUMAN Uncharacterized protein OS=Homo sapiens PE=1 SV=1 |  |  |  |  |  |  |  |  |
| $30-9 | | 8 | 4 | 28.81% | 25834 | 6.1 | tr\|Q6PIL8\|Q6PIL8_HUMAN IGK@ protein OS=Homo sapiens GN=IGK@ PE=1 SV=1 |  |  |  |  |  |  |  |  |
| $30-10 | | 8 | 4 | 29.18% | 25600.7 | 5.8 | tr\|A0A087WZW8\|A0A087WZW8_HUMAN Protein IGKV3-11 OS=Homo sapiens GN=IGKV3-11 PE=4 SV=1 |  |  |  |  |  |  |  |  |
| $30-11 | | 8 | 4 | 29.06% | 25570.9 | 8.8 | tr\|A0A087WWV8\|A0A087WWV8_HUMAN Protein IGKV1-8 OS=Homo sapiens GN=IGKV1-8 PE=4 SV=1 |  |  |  |  |  |  |  |  |
| $30-12 | | 8 | 4 | 51.52% | 14459.3 | 7.7 | tr\|A0A087X1V9\|A0A087X1V9_HUMAN Beta-2-microglobulin OS=Homo sapiens GN=IGKC PE=1 SV=2 |  |  |  |  |  |  |  |  |
| $30-13 | | 8 | 4 | 28.94% | 25520.7 | 6.1 | tr\|Q6PJF2\|Q6PJF2_HUMAN IGK@ protein OS=Homo sapiens GN=IGK@ PE=1 SV=1 |  |  |  |  |  |  |  |  |
| $30-14 | | 8 | 4 | 28.45% | 26234.5 | 8.2 | tr\|Q8TCD0\|Q8TCD0_HUMAN Uncharacterized protein OS=Homo sapiens PE=1 SV=1 |  |  |  |  |  |  |  |  |
| $30-15 | | 8 | 4 | 31.05% | 24029.9 | 8.3 | tr\|Q0KKI6\|Q0KKI6_HUMAN Immunoblobulin light chain (Fragment) OS=Homo sapiens PE=1 SV=1 |  |  |  |  |  |  |  |  |
| $30-16 | | 8 | 4 | 28.81% | 25772.9 | 5.9 | tr\|Q6P5S8\|Q6P5S8_HUMAN IGK@ protein OS=Homo sapiens GN=IGK@ PE=1 SV=1 |  |  |  |  |  |  |  |  |
|  | R16062_1_CTGF,16810 | -.TVAAPSVFIFPPSDEQLK.S | 1947.22 | -0.418 | 2 | 1 | 2.4384 | 0.3707 | 241.4 | 1 | 12\|34 | 4.37 | 1 | 16 |  |
|  | R16062_1_CTGF,16824 | -.TVAAPSVFIFPPSDEQLK.S | 1947.22 | 0.694 | 2 | 1 | 2.2778 | 0.3881 | 433.2 | 1 | 16\|34 | 4.37 | 1 | 16 |  |
|  | R16062_1_CTGF,13148 | K.DSTYSLSSTLTLSK.A | 1503.6336 | 0.0536 | 2 | 1 | 2.3166 | 0.351 | 426.8 | 1 | 13\|26 | 5.84 | 1 | 16 |  |
|  | R16062_1_CTGF,13210 | K.DSTYSLSSTLTLSK.A | 1503.6336 | 1.3176 | 2 | 1 | 2.7001 | 0.3457 | 635.7 | 1 | 14\|26 | 5.84 | 1 | 16 |  |
|  | R16062_1_CTGF,18920 | K.SGTASVVCLLNNFYPR.E | 1799.0141 | 2.0901 | 2 | 1 | 2.4929 | 0.4014 | 863.6 | 1 | 16\|30 | 7.94 | 1 | 16 |  |
|  | R16062_1_CTGF,18989 | K.SGTASVVCLLNNFYPR.E | 1799.0141 | 0.5071 | 2 | 1 | 2.345 | 0.3552 | 548.4 | 1 | 13\|30 | 7.94 | 1 | 16 |  |
|  | R16062_1_CTGF,7000 | K.VDNALQSGNSQESVTEQDSK.D | 2137.1621 | 0.8421 | 2 | 1 | 3.5063 | 0.4954 | 1738.5 | 1 | 23\|38 | 3.92 | 1 | 16 |  |
|  | R16062_1_CTGF,7012 | K.VDNALQSGNSQESVTEQDSK.D | 2137.1621 | 0.5211 | 2 | 1 | 3.2769 | 0.5666 | 1226.9 | 1 | 21\|38 | 3.92 | 1 | 16 |  |
| $31-1 | | 8 | 4 | 10.69% | 74680.9 | 7.6 | sp\|Q15582\|BGH3_HUMAN Transforming growth factor-beta-induced protein ig-h3 OS=Homo sapiens GN=TGFBI PE=1 SV=1 |  |  |  |  |  |  |  |  |
| $31-2 | | 8 | 4 | 10.69% | 74650.8 | 7.6 | tr\|Q53GU8\|Q53GU8_HUMAN Transforming growth factor, beta-induced, 68kDa variant (Fragment) OS=Homo sapiens PE=2 SV=1 |  |  |  |  |  |  |  |  |
|  | R16062_1_CTGF,21128 | K.GCPAALPLSNLYETLGVVGSTTTQLYTDR.T | 3099.4316 | -0.2604 | 3 | 1 | 3.8213 | 0.5382 | 918.7 | 1 | 32\|112 | 4.37 | 1 | 2 |  |
|  | R16062_1_CTGF,21144 | K.GCPAALPLSNLYETLGVVGSTTTQLYTDR.T | 3099.4316 | 1.6796 | 3 | 1 | 3.7838 | 0.4177 | 973.5 | 1 | 29\|112 | 4.37 | 1 | 2 |  |
|  | R16062_1_CTGF,17024 | R.FSM*LVAAIQSAGLTETLNR.E | 2039.3424 | -1.3306 | 2 | 1 | 2.394 | 0.3118 | 714.5 | 1 | 15\|36 | 6 | 1 | 2 |  |
|  | R16062_1_CTGF,17044 | R.FSM*LVAAIQSAGLTETLNR.E | 2039.3424 | 1.6064 | 2 | 1 | 2.5744 | 0.4523 | 785.1 | 1 | 17\|36 | 6 | 1 | 2 |  |
|  | R16062_1_CTGF,17058 | R.FSM*LVAAIQSAGLTETLNR.E | 2039.3424 | 0.5494 | 2 | 1 | 3.1857 | 0.398 | 1863.4 | 1 | 21\|36 | 6 | 1 | 2 |  |
|  | R16062_1_CTGF,17055 | R.GDELADSALEIFK.Q | 1408.5355 | 0.3215 | 2 | 1 | 2.6357 | 0.3759 | 1348.9 | 1 | 17\|24 | 3.92 | 1 | 2 |  |
|  | R16062_1_CTGF,17143 | R.GDELADSALEIFK.Q | 1408.5355 | 1.3865 | 2 | 1 | 3.0088 | 0.2949 | 1304.5 | 1 | 16\|24 | 3.92 | 1 | 2 |  |
|  | R16062_1_CTGF,18766 - 18769 | R.LTLLAPLNSVFK.D | 1316.6139 | -0.2971 | 2 | 1 | 2.5919 | 0.1917 | 456.3 | 1 | 14\|22 | 8.75 | 1 | 2 |  |
| $32-1 | | 8 | 4 | 12.47% | 52494.7 | 5.9 | sp\|P10909\|CLUS_HUMAN Clusterin OS=Homo sapiens GN=CLU PE=1 SV=1 |  |  |  |  |  |  |  |  |
|  | R16062_1_CTGF,15571 | K.LFDSDPITVTVPVEVSR.K | 1875.1122 | 0.5452 | 2 | 1 | 2.7417 | 0.4548 | 699.3 | 1 | 17\|32 | 4.03 | 1 | 1 |  |
|  | R16062_1_CTGF,15579 - 15580 | K.LFDSDPITVTVPVEVSR.K | 1875.1122 | 0.7662 | 2 | 1 | 3.1737 | 0.4545 | 1069.7 | 1 | 20\|32 | 4.03 | 1 | 1 |  |
|  | R16062_1_CTGF,15624 | K.LFDSDPITVTVPVEVSR.K | 1875.1122 | 1.0452 | 2 | 1 | 2.6036 | 0.4691 | 1058 | 1 | 19\|32 | 4.03 | 1 | 1 |  |
|  | R16062_1_CTGF,17919 | R.ASSIIDELFQDR.F | 1394.5121 | 0.5241 | 2 | 1 | 2.9236 | 0.3169 | 955.4 | 1 | 15\|22 | 4.03 | 1 | 1 |  |
|  | R16062_1_CTGF,17922 | R.ASSIIDELFQDR.F | 1394.5121 | 0.3331 | 2 | 1 | 2.3748 | 0.2301 | 935.7 | 1 | 15\|22 | 4.03 | 1 | 1 |  |
|  | R16062_1_CTGF,8946 | R.EILSVDCSTNNPSQAK.L | 1763.8785 | 0.5215 | 2 | 1 | 3.0205 | 0.4486 | 798.7 | 1 | 17\|30 | 4.37 | 1 | 1 |  |
|  | R16062_1_CTGF,8957 | R.EILSVDCSTNNPSQAK.L | 1763.8785 | 0.6365 | 2 | 1 | 2.5384 | 0.4252 | 1131.4 | 1 | 18\|30 | 4.37 | 1 | 1 |  |
|  | R16062_1_CTGF,9782 | R.ELDESLQVAER.L | 1289.3743 | -0.8527 | 2 | 1 | 2.3858 | 0.2681 | 384 | 2 | 11\|20 | 4 | 1 | 1 |  |
| $33-1 | | 7 | 4 | 7.18% | 70052.2 | 5.5 | sp\|P0DMV9\|HS71B_HUMAN Heat shock 70 kDa protein 1B OS=Homo sapiens GN=HSPA1B PE=1 SV=1 |  |  |  |  |  |  |  |  |
| $33-2 | | 7 | 4 | 7.18% | 70052.2 | 5.5 | sp\|P0DMV8\|HS71A_HUMAN Heat shock 70 kDa protein 1A OS=Homo sapiens GN=HSPA1A PE=1 SV=1 |  |  |  |  |  |  |  |  |
| $33-3 | | 7 | 4 | 7.18% | 70052.2 | 5.5 | tr\|A8K5I0\|A8K5I0_HUMAN Epididymis secretory protein Li 103 OS=Homo sapiens GN=HSPA1B PE=2 SV=1 |  |  |  |  |  |  |  |  |
| $33-4 | | 7 | 4 | 7.17% | 70109.2 | 5.5 | tr\|A0A0G2JIW1\|A0A0G2JIW1_HUMAN Heat shock 70 kDa protein 1B OS=Homo sapiens GN=HSPA1B PE=1 SV=1 |  |  |  |  |  |  |  |  |
| $33-5 | | 7 | 4 | 7.46% | 67419.3 | 5.4 | tr\|B4DNT8\|B4DNT8_HUMAN cDNA FLJ54370, highly similar to Heat shock 70 kDa protein 1 OS=Homo sapiens PE=2 SV=1 |  |  |  |  |  |  |  |  |
| $33-6 | | 7 | 4 | 6.49% | 77495.7 | 6 | tr\|Q59EJ3\|Q59EJ3_HUMAN Heat shock 70kDa protein 1A variant (Fragment) OS=Homo sapiens PE=2 SV=1 |  |  |  |  |  |  |  |  |
|  | R16062_1_CTGF,14918 | K.DAGVIAGLNVLR.I | 1198.3975 | 0.1645 | 2 | 1 | 3.0132 | 0.3954 | 1723.8 | 1 | 19\|22 | 5.84 | 2 | 11 |  |
|  | R16062_1_CTGF,14933 | K.DAGVIAGLNVLR.I | 1198.3975 | 0.1015 | 2 | 1 | 2.5173 | 0.3155 | 1146.4 | 1 | 16\|22 | 5.84 | 2 | 11 |  |
|  | R16062_1_CTGF,14546 | K.LLQDFFNGR.D | 1110.2473 | 0.1213 | 2 | 1 | 2.3748 | 0.4172 | 954.5 | 1 | 13\|16 | 5.84 | 1 | 6 |  |
|  | R16062_1_CTGF,11810 | K.NQVALNPQNTVFDAK.R | 1659.8249 | 0.3119 | 2 | 1 | 2.5024 | 0.4511 | 667.2 | 1 | 15\|28 | 5.84 | 1 | 6 |  |
|  | R16062_1_CTGF,11819 | K.NQVALNPQNTVFDAK.R | 1659.8249 | 0.5679 | 2 | 1 | 2.783 | 0.437 | 968 | 1 | 18\|28 | 5.84 | 1 | 6 |  |
|  | R16062_1_CTGF,14864 | R.FEELCSDLFR.S | 1316.4348 | 1.2978 | 2 | 1 | 2.5565 | 0.2079 | 1174.2 | 1 | 15\|18 | 4.14 | 1 | 6 |  |
|  | R16062_1_CTGF,14880 | R.FEELCSDLFR.S | 1316.4348 | 0.8618 | 2 | 1 | 2.5565 | 0.287 | 1802.9 | 1 | 16\|18 | 4.14 | 1 | 6 |  |
| $34-1 | | 7 | 4 | 12.44% | 46736.5 | 5.4 | sp\|P01009\|A1AT_HUMAN Alpha-1-antitrypsin OS=Homo sapiens GN=SERPINA1 PE=1 SV=3 |  |  |  |  |  |  |  |  |
| $34-2 | | 7 | 4 | 12.44% | 46736.5 | 5.4 | tr\|E9KL23\|E9KL23_HUMAN Epididymis secretory sperm binding protein Li 44a OS=Homo sapiens PE=2 SV=1 |  |  |  |  |  |  |  |  |
|  | R16062_1_CTGF,10603 | K.DTEEEDFHVDQVTTVK.V | 1892.9549 | 0.7389 | 2 | 1 | 2.7007 | 0.413 | 852.1 | 1 | 15\|30 | 4.01 | 1 | 2 |  |
|  | R16062_1_CTGF,13137 | K.SVLGQLGITK.V | 1016.2168 | 0.4418 | 2 | 1 | 2.5125 | 0.4733 | 722.3 | 1 | 15\|18 | 8.47 | 1 | 2 |  |
|  | R16062_1_CTGF,13147 | K.SVLGQLGITK.V | 1016.2168 | 0.4048 | 2 | 1 | 2.3014 | 0.4266 | 548.6 | 1 | 12\|18 | 8.47 | 1 | 2 |  |
|  | R16062_1_CTGF,12555 | K.VFSNGADLSGVTEEAPLK.L | 1835.0049 | 0.6599 | 2 | 1 | 3.3343 | 0.4225 | 950.9 | 1 | 19\|34 | 4.14 | 1 | 2 |  |
|  | R16062_1_CTGF,13000 | K.VFSNGADLSGVTEEAPLK.L | 1835.0049 | -0.0361 | 2 | 1 | 3.6023 | 0.4546 | 1126.8 | 1 | 20\|34 | 4.14 | 1 | 2 |  |
|  | R16062_1_CTGF,13022 | K.VFSNGADLSGVTEEAPLK.L | 1835.0049 | -0.4281 | 2 | 1 | 3.3818 | 0.407 | 1874.4 | 1 | 23\|34 | 4.14 | 1 | 2 |  |
|  | R16062_1_CTGF,7944 | R.SASLHLPK.L | 853.0012 | -0.1688 | 2 | 1 | 2.2203 | 0.1497 | 1342.2 | 1 | 13\|14 | 8.49 | 1 | 2 |  |
| $35-1 | | 7 | 4 | 10.49% | 62293.6 | 6 | sp\|Q16555\|DPYL2_HUMAN Dihydropyrimidinase-related protein 2 OS=Homo sapiens GN=DPYSL2 PE=1 SV=1 |  |  |  |  |  |  |  |  |
| $35-2 | | 7 | 4 | 9.55% | 68183.9 | 5.9 | tr\|Q59GB4\|Q59GB4_HUMAN Dihydropyrimidinase-like 2 variant (Fragment) OS=Homo sapiens PE=2 SV=1 |  |  |  |  |  |  |  |  |
| $35-3 | | 7 | 4 | 10.49% | 62270.5 | 5.9 | tr\|Q53ET2\|Q53ET2_HUMAN Dihydropyrimidinase-like 2 variant (Fragment) OS=Homo sapiens PE=2 SV=1 |  |  |  |  |  |  |  |  |
|  | R16062_1_CTGF,12436 | K.QIGENLIVPGGVK.T | 1324.5513 | 0.6083 | 2 | 1 | 2.2128 | 0.3212 | 279.1 | 8 | 10\|24 | 6 | 1 | 3 |  |
|  | R16062_1_CTGF,12514 | R.AITIANQTNCPLYITK.V | 1822.0891 | 0.5961 | 2 | 1 | 2.4866 | 0.4493 | 853.3 | 1 | 15\|30 | 8.24 | 1 | 3 |  |
|  | R16062_1_CTGF,20400 | R.FQLTDCQIYEVLSVIR.D | 1985.2632 | 0.5592 | 2 | 1 | 2.3932 | 0.3976 | 1038.4 | 1 | 18\|30 | 4.37 | 1 | 3 |  |
|  | R16062_1_CTGF,20407 | R.FQLTDCQIYEVLSVIR.D | 1985.2632 | 0.8332 | 2 | 1 | 2.621 | 0.485 | 1098.1 | 1 | 16\|30 | 4.37 | 1 | 3 |  |
|  | R16062_1_CTGF,11857 | R.GLYDGPVCEVSVTPK.T | 1621.8068 | 0.8578 | 2 | 1 | 2.3629 | 0.1761 | 801.6 | 2 | 15\|28 | 4.37 | 1 | 3 |  |
|  | R16062_1_CTGF,11863 | R.GLYDGPVCEVSVTPK.T | 1621.8068 | -0.2602 | 2 | 1 | 2.2545 | 0.2681 | 549.2 | 1 | 14\|28 | 4.37 | 1 | 3 |  |
|  | R16062_1_CTGF,11922 | R.GLYDGPVCEVSVTPK.T | 1621.8068 | 1.5418 | 2 | 1 | 2.53 | 0.2693 | 850.3 | 1 | 15\|28 | 4.37 | 1 | 3 |  |
| $36-1 | | 7 | 4 | 10.13% | 66022.7 | 5.6 | sp\|Q07065\|CKAP4_HUMAN Cytoskeleton-associated protein 4 OS=Homo sapiens GN=CKAP4 PE=1 SV=2 |  |  |  |  |  |  |  |  |
| $36-2 | | 7 | 4 | 10.13% | 66022.7 | 5.6 | tr\|A0A024RBH2\|A0A024RBH2_HUMAN Cytoskeleton-associated protein 4, isoform CRA_c OS=Homo sapiens GN=CKAP4 PE=4 SV=1 |  |  |  |  |  |  |  |  |
| $36-3 | | 7 | 4 | 10.17% | 67818.5 | 9.3 | tr\|Q6NWZ1\|Q6NWZ1_HUMAN CKAP4 protein (Fragment) OS=Homo sapiens GN=CKAP4 PE=2 SV=1 |  |  |  |  |  |  |  |  |
| $36-4 | | 7 | 4 | 10.89% | 62060.7 | 5.5 | tr\|Q8TB01\|Q8TB01_HUMAN Similar to cytoskeleton-associated protein 4 (Fragment) OS=Homo sapiens PE=2 SV=1 |  |  |  |  |  |  |  |  |
| $36-5 | | 7 | 4 | 11.71% | 58151 | 5.2 | tr\|B3KVX6\|B3KVX6_HUMAN cDNA FLJ41699 fis, clone HCHON2004776, highly similar to Homo sapiens cytoskeleton-associated protein 4 (CKAP4), mRNA OS=Homo sapiens PE=2 SV=1 |  |  |  |  |  |  |  |  |
|  | R16062_1_CTGF,19802 | K.VQSLQATFGTFESILR.S | 1798.0326 | -0.4124 | 2 | 1 | 2.4624 | 0.4078 | 419 | 1 | 12\|30 | 5.97 | 1 | 5 |  |
|  | R16062_1_CTGF,19804 | K.VQSLQATFGTFESILR.S | 1798.0326 | 1.4466 | 2 | 1 | 2.3564 | 0.4356 | 471.4 | 1 | 12\|30 | 5.97 | 1 | 5 |  |
|  | R16062_1_CTGF,19859 | K.VQSLQATFGTFESILR.S | 1798.0326 | 0.8666 | 2 | 1 | 2.4444 | 0.3926 | 613 | 1 | 13\|30 | 5.97 | 1 | 5 |  |
|  | R16062_1_CTGF,13031 | R.DFTSLENTVEER.L | 1440.4945 | 0.5915 | 2 | 1 | 2.551 | 0.3429 | 689.5 | 1 | 13\|22 | 4 | 1 | 5 |  |
|  | R16062_1_CTGF,11393 | R.LEGLGSSEADQDGLASTVR.S | 1905.9984 | 0.5834 | 2 | 1 | 2.4074 | 0.4061 | 593.5 | 1 | 15\|36 | 3.92 | 1 | 5 |  |
|  | R16062_1_CTGF,11401 | R.LEGLGSSEADQDGLASTVR.S | 1905.9984 | 0.9084 | 2 | 1 | 2.2643 | 0.4306 | 790.5 | 1 | 18\|36 | 3.92 | 1 | 5 |  |
|  | R16062_1_CTGF,11657 | R.SVGELPSTVESLQK.V | 1474.6387 | -0.3113 | 2 | 1 | 2.962 | 0.3921 | 691.9 | 1 | 15\|26 | 4.53 | 1 | 5 |  |
| $37-1 | | 6 | 4 | 7.75% | 78182.1 | 8.5 | sp\|P02788\|TRFL_HUMAN Lactotransferrin OS=Homo sapiens GN=LTF PE=1 SV=6 |  |  |  |  |  |  |  |  |
| $37-2 | | 6 | 4 | 7.90% | 76626 | 8.4 | tr\|E7EQB2\|E7EQB2_HUMAN Lactotransferrin (Fragment) OS=Homo sapiens GN=LTF PE=1 SV=1 |  |  |  |  |  |  |  |  |
| $37-3 | | 6 | 4 | 7.74% | 78357.2 | 8.5 | tr\|B3VMW0\|B3VMW0_HUMAN Lactoferrin OS=Homo sapiens PE=2 SV=1 |  |  |  |  |  |  |  |  |
| $37-4 | | 6 | 4 | 7.74% | 78322.2 | 8.6 | tr\|Q5EK51\|Q5EK51_HUMAN Lactoferrin OS=Homo sapiens PE=2 SV=1 |  |  |  |  |  |  |  |  |
| $37-5 | | 6 | 4 | 8.26% | 73170 | 8.1 | tr\|B7ZAL5\|B7ZAL5_HUMAN cDNA, FLJ79229, highly similar to Lactotransferrin (EC 3.4.21.-) OS=Homo sapiens PE=2 SV=1 |  |  |  |  |  |  |  |  |
| $37-6 | | 6 | 4 | 7.77% | 77969.8 | 8.5 | tr\|E7ER44\|E7ER44_HUMAN Lactotransferrin OS=Homo sapiens GN=LTF PE=1 SV=1 |  |  |  |  |  |  |  |  |
| $37-7 | | 6 | 4 | 7.74% | 78382.3 | 8.6 | tr\|W8QEY1\|W8QEY1_HUMAN Lactoferrin OS=Homo sapiens PE=2 SV=1 |  |  |  |  |  |  |  |  |
| $37-8 | | 6 | 4 | 7.75% | 78182.1 | 8.5 | tr\|V9HWI4\|V9HWI4_HUMAN Epididymis luminal protein 110 OS=Homo sapiens GN=HEL110 PE=2 SV=1 |  |  |  |  |  |  |  |  |
| $37-9 | | 6 | 4 | 18.33% | 33025.3 | 7.8 | tr\|B4E1V0\|B4E1V0_HUMAN cDNA FLJ54839, highly similar to Lactotransferrin (EC 3.4.21.-) OS=Homo sapiens PE=2 SV=1 |  |  |  |  |  |  |  |  |
| $37-10 | | 6 | 4 | 7.89% | 76753.2 | 8.4 | tr\|B3KSL2\|B3KSL2_HUMAN cDNA FLJ36533 fis, clone TRACH2004428, highly similar to Lactotransferrin (EC 3.4.21.-) (Fragment) OS=Homo sapiens PE=2 SV=1 |  |  |  |  |  |  |  |  |
| $37-11 | | 6 | 4 | 7.76% | 77980.7 | 8.5 | tr\|Q2TUW9\|Q2TUW9_HUMAN Lactoferrin OS=Homo sapiens PE=2 SV=1 |  |  |  |  |  |  |  |  |
|  | R16062_1_CTGF,16695 | K.LADFALLCLDGK.R | 1336.5523 | 1.0763 | 2 | 1 | 2.325 | 0.2815 | 514.4 | 1 | 12\|22 | 4.21 | 1 | 11 |  |
|  | R16062_1_CTGF,16719 | K.LADFALLCLDGK.R | 1336.5523 | 0.8443 | 2 | 1 | 2.8123 | 0.4684 | 1278.5 | 1 | 17\|22 | 4.21 | 1 | 11 |  |
|  | R16062_1_CTGF,13539 | K.NLLFNDNTECLAR.L | 1580.718 | 0.265 | 2 | 1 | 2.3296 | 0.3611 | 714.1 | 1 | 14\|24 | 4.37 | 1 | 11 |  |
|  | R16062_1_CTGF,14463 | K.YLGPQYVAGITNLK.K | 1537.7844 | 0.6284 | 2 | 1 | 2.858 | 0.4022 | 690.3 | 1 | 15\|26 | 8.5 | 1 | 11 |  |
|  | R16062_1_CTGF,14536 | K.YLGPQYVAGITNLK.K | 1537.7844 | 1.7524 | 2 | 1 | 2.5017 | 0.3213 | 372.6 | 1 | 11\|26 | 8.5 | 1 | 11 |  |
|  | R16062_1_CTGF,11151 | R.SNLCALCIGDEQGENK.C | 1808.9127 | 0.6287 | 2 | 1 | 2.6056 | 0.3879 | 866.2 | 1 | 16\|30 | 4.14 | 1 | 11 |  |
| $38-1 | | 6 | 4 | 34.95% | 11367.3 | 11 | sp\|P62805\|H4_HUMAN Histone H4 OS=Homo sapiens GN=HIST1H4A PE=1 SV=2 |  |  |  |  |  |  |  |  |
| $38-2 | | 6 | 4 | 34.95% | 11367.3 | 11 | tr\|B2R4R0\|B2R4R0_HUMAN Histone H4 OS=Homo sapiens GN=HIST1H4L PE=2 SV=1 |  |  |  |  |  |  |  |  |
| $38-3 | | 6 | 4 | 34.95% | 11314.2 | 11 | tr\|Q0VAS5\|Q0VAS5_HUMAN Histone H4 OS=Homo sapiens GN=HIST1H4H PE=2 SV=1 |  |  |  |  |  |  |  |  |
|  | R16062_1_CTGF,13584 | K.VFLENVIR.D | 990.1811 | 0.9301 | 2 | 1 | 2.4608 | 0.2928 | 605.1 | 1 | 12\|14 | 5.97 | 1 | 3 |  |
|  | R16062_1_CTGF,13588 | K.VFLENVIR.D | 990.1811 | 0.4151 | 2 | 1 | 2.2471 | 0.3204 | 511 | 11 | 11\|14 | 5.97 | 1 | 3 |  |
|  | R16062_1_CTGF,5426 | R.DAVTYTEHAK.R | 1135.2087 | 1.0337 | 2 | 1 | 2.3148 | 0.3524 | 701.1 | 1 | 13\|18 | 5.32 | 1 | 3 |  |
|  | R16062_1_CTGF,6635 | R.DNIQGITK.P | 888.9888 | -0.1162 | 2 | 1 | 2.4529 | 0.2283 | 343.4 | 1 | 12\|14 | 5.84 | 1 | 3 |  |
|  | R16062_1_CTGF,11002 | R.ISGLIYEETR.G | 1181.3206 | -1.1724 | 2 | 1 | 2.5885 | 0.3824 | 882.9 | 1 | 14\|18 | 4.53 | 1 | 3 |  |
|  | R16062_1_CTGF,11288 | R.ISGLIYEETR.G | 1181.3206 | 0.9376 | 2 | 1 | 2.3077 | 0.1229 | 1100.8 | 2 | 15\|18 | 4.53 | 1 | 3 |  |
| $39-1 | | 6 | 4 | 8.49% | 93313.7 | 7.3 | sp\|Q15063\|POSTN_HUMAN Periostin OS=Homo sapiens GN=POSTN PE=1 SV=2 |  |  |  |  |  |  |  |  |
| $39-2 | | 6 | 4 | 8.49% | 93313.7 | 7.3 | tr\|A0A024RDS2\|A0A024RDS2_HUMAN Periostin, osteoblast specific factor, isoform CRA_c OS=Homo sapiens GN=POSTN PE=4 SV=1 |  |  |  |  |  |  |  |  |
| $39-3 | | 6 | 4 | 9.11% | 87020.4 | 7.9 | tr\|A0A024RDT5\|A0A024RDT5_HUMAN Periostin, osteoblast specific factor, isoform CRA_a OS=Homo sapiens GN=POSTN PE=4 SV=1 |  |  |  |  |  |  |  |  |
| $39-4 | | 6 | 4 | 8.79% | 90143.2 | 8.1 | tr\|B1ALD9\|B1ALD9_HUMAN Periostin OS=Homo sapiens GN=POSTN PE=1 SV=1 |  |  |  |  |  |  |  |  |
| $39-5 | | 6 | 4 | 9.09% | 87271.8 | 8.1 | tr\|C0IMJ3\|C0IMJ3_HUMAN Periostin isoform thy6 OS=Homo sapiens PE=2 SV=1 |  |  |  |  |  |  |  |  |
|  | R16062_1_CTGF,17230 | K.VGLNELYNGQILETIGGK.Q | 1919.1682 | 0.8562 | 2 | 1 | 2.4539 | 0.5022 | 634.8 | 1 | 14\|34 | 4.53 | 1 | 5 |  |
|  | R16062_1_CTGF,18099 | R.AAAITSDILEALGR.D | 1401.5909 | -1.7291 | 2 | 1 | 2.7449 | 0.4095 | 629 | 1 | 15\|26 | 4.37 | 1 | 5 |  |
|  | R16062_1_CTGF,18120 | R.AAAITSDILEALGR.D | 1401.5909 | -0.8851 | 2 | 1 | 3.8428 | 0.512 | 1136 | 1 | 17\|26 | 4.37 | 1 | 5 |  |
|  | R16062_1_CTGF,18122 | R.AAAITSDILEALGR.D | 1401.5909 | 0.6919 | 2 | 1 | 3.5158 | 0.4951 | 1961.4 | 1 | 20\|26 | 4.37 | 1 | 5 |  |
|  | R16062_1_CTGF,16202 | R.DQGPNVCALQQILGTK.K | 1742.9488 | 1.2148 | 2 | 1 | 2.3085 | 0.1587 | 180.9 | 34 | 10\|30 | 5.83 | 1 | 5 |  |
|  | R16062_1_CTGF,21069 | R.VLTQIGTSIQDFIEAEDDLSSFR.A | 2585.8049 | 0.1489 | 3 | 1 | 4.4937 | 0.5194 | 1307.8 | 1 | 29\|88 | 3.77 | 1 | 5 |  |
| $40-1 | | 4 | 4 | 8.78% | 67568.5 | 4.5 | sp\|P27824\|CALX_HUMAN Calnexin OS=Homo sapiens GN=CANX PE=1 SV=2 |  |  |  |  |  |  |  |  |
|  | R16062_1_CTGF,14947 | K.APVPTGEVYFADSFDR.G | 1771.9073 | 0.4263 | 2 | 1 | 2.8264 | 0.4754 | 638.7 | 1 | 18\|30 | 4.03 | 1 | 1 |  |
|  | R16062_1_CTGF,19310 | K.IPNPDFFEDLEPFR.M | 1736.9046 | -0.5764 | 2 | 1 | 2.8643 | 0.412 | 770.2 | 1 | 15\|26 | 3.92 | 1 | 1 |  |
|  | R16062_1_CTGF,11590 | K.TPELNLDQFHDK.T | 1457.5699 | 0.7579 | 2 | 1 | 2.3899 | 0.2677 | 692.9 | 1 | 13\|22 | 4.53 | 1 | 1 |  |
|  | R16062_1_CTGF,14836 | R.GTLSGWILSK.A | 1062.244 | 0.253 | 2 | 1 | 2.4757 | 0.3938 | 734.4 | 1 | 13\|18 | 8.75 | 1 | 1 |  |
| $41-1 | | 8 | 3 | 3.92% | 103058 | 5.3 | sp\|P12814\|ACTN1_HUMAN Alpha-actinin-1 OS=Homo sapiens GN=ACTN1 PE=1 SV=2 |  |  |  |  |  |  |  |  |
| $41-2 | | 8 | 3 | 3.84% | 104854 | 5.3 | sp\|O43707\|ACTN4_HUMAN Alpha-actinin-4 OS=Homo sapiens GN=ACTN4 PE=1 SV=2 |  |  |  |  |  |  |  |  |
| $41-3 | | 8 | 3 | 3.92% | 103058 | 5.3 | tr\|A0A024R694\|A0A024R694_HUMAN Actinin, alpha 1, isoform CRA_a OS=Homo sapiens GN=ACTN1 PE=4 SV=1 |  |  |  |  |  |  |  |  |
| $41-4 | | 8 | 3 | 6.55% | 61575.4 | 5.1 | tr\|B4DFY0\|B4DFY0_HUMAN cDNA FLJ53313, highly similar to Alpha-actinin-1 OS=Homo sapiens PE=2 SV=1 |  |  |  |  |  |  |  |  |
| $41-5 | | 8 | 3 | 4.26% | 94783.8 | 5.5 | tr\|B7Z565\|B7Z565_HUMAN cDNA FLJ54739, highly similar to Alpha-actinin-1 OS=Homo sapiens PE=2 SV=1 |  |  |  |  |  |  |  |  |
| $41-6 | | 8 | 3 | 4.26% | 94825.9 | 5.5 | tr\|H9KV75\|H9KV75_HUMAN Alpha-actinin-1 OS=Homo sapiens GN=ACTN1 PE=1 SV=1 |  |  |  |  |  |  |  |  |
| $41-7 | | 8 | 3 | 5.52% | 73619.8 | 5.2 | tr\|Q96BG6\|Q96BG6_HUMAN ACTN4 protein (Fragment) OS=Homo sapiens GN=ACTN4 PE=2 SV=2 |  |  |  |  |  |  |  |  |
|  | R16062_1_CTGF,17606 | K.GYEEWLLNEIR.R | 1422.5671 | -0.8419 | 2 | 1 | 2.3594 | 0.3406 | 668.5 | 1 | 13\|20 | 4.25 | 1 | 7 |  |
|  | R16062_1_CTGF,17653 | K.GYEEWLLNEIR.R | 1422.5671 | 0.2211 | 2 | 1 | 2.5454 | 0.3849 | 710.6 | 1 | 12\|20 | 4.25 | 1 | 7 |  |
|  | R16062_1_CTGF,17670 | K.GYEEWLLNEIR.R | 1422.5671 | 0.6101 | 2 | 1 | 2.3802 | 0.3114 | 639.7 | 1 | 12\|20 | 4.25 | 1 | 7 |  |
|  | R16062_1_CTGF,12339 | R.TINEVENQILTR.D | 1430.5891 | -0.5789 | 2 | 1 | 2.4995 | 0.2689 | 780.7 | 1 | 14\|22 | 4.53 | 1 | 7 |  |
|  | R16062_1_CTGF,12382 | R.TINEVENQILTR.D | 1430.5891 | -0.4539 | 2 | 1 | 2.8489 | 0.3543 | 869.2 | 1 | 13\|22 | 4.53 | 1 | 7 |  |
|  | R16062_1_CTGF,19534 | R.VGWEQLLTTIAR.T | 1387.6091 | 0.0421 | 2 | 1 | 2.7613 | 0.4408 | 743.8 | 1 | 13\|22 | 5.97 | 1 | 7 |  |
|  | R16062_1_CTGF,19608 | R.VGWEQLLTTIAR.T | 1387.6091 | 0.9351 | 2 | 1 | 2.6819 | 0.2483 | 649.1 | 1 | 12\|22 | 5.97 | 1 | 7 |  |
|  | R16062_1_CTGF,19641 | R.VGWEQLLTTIAR.T | 1387.6091 | 0.4691 | 2 | 1 | 2.4161 | 0.3842 | 826.7 | 1 | 14\|22 | 5.97 | 1 | 7 |  |
| $42-1 | | 7 | 3 | 9.35% | 44614.5 | 8.3 | sp\|P00558\|PGK1_HUMAN Phosphoglycerate kinase 1 OS=Homo sapiens GN=PGK1 PE=1 SV=3 |  |  |  |  |  |  |  |  |
| $42-2 | | 7 | 3 | 9.35% | 44614.5 | 8.3 | tr\|V9HWF4\|V9HWF4_HUMAN Phosphoglycerate kinase OS=Homo sapiens GN=HEL-S-68p PE=2 SV=1 |  |  |  |  |  |  |  |  |
|  | R16062_1_CTGF,16792 | K.ACANPAAGSVILLENLR.F | 1770.0174 | -0.5336 | 2 | 1 | 2.744 | 0.3714 | 796.1 | 1 | 18\|32 | 6.04 | 1 | 2 |  |
|  | R16062_1_CTGF,16822 | K.ACANPAAGSVILLENLR.F | 1770.0174 | 0.6894 | 2 | 1 | 2.9349 | 0.4412 | 1006.5 | 1 | 17\|32 | 6.04 | 1 | 2 |  |
|  | R16062_1_CTGF,16837 | K.ACANPAAGSVILLENLR.F | 1770.0174 | 1.2454 | 2 | 1 | 2.7095 | 0.4825 | 757.6 | 1 | 16\|32 | 6.04 | 1 | 2 |  |
|  | R16062_1_CTGF,16383 | K.VLPGVDALSNI.- | 1098.2747 | 0.4707 | 2 | 1 | 2.6841 | 0.4752 | 1420.3 | 1 | 15\|20 | 3.8 | 1 | 2 |  |
|  | R16062_1_CTGF,16410 | K.VLPGVDALSNI.- | 1098.2747 | 0.1587 | 2 | 1 | 2.637 | 0.468 | 1151.8 | 1 | 14\|20 | 3.8 | 1 | 2 |  |
|  | R16062_1_CTGF,13567 - 13568 | K.YSLEPVAVELK.S | 1248.45 | -0.04 | 2 | 1 | 2.4836 | 0.3849 | 741.3 | 1 | 15\|20 | 4.53 | 1 | 2 |  |
|  | R16062_1_CTGF,13590 | K.YSLEPVAVELK.S | 1248.45 | 0.511 | 2 | 1 | 2.2639 | 0.4207 | 704.4 | 1 | 15\|20 | 4.53 | 1 | 2 |  |
| $43-1 | | 7 | 3 | 14.43% | 33922.3 | 5.5 | sp\|P20774\|MIME_HUMAN Mimecan OS=Homo sapiens GN=OGN PE=1 SV=1 |  |  |  |  |  |  |  |  |
| $43-2 | | 7 | 3 | 14.43% | 33922.3 | 5.5 | tr\|A8K0R3\|A8K0R3_HUMAN Osteoglycin (Osteoinductive factor, mimecan), isoform CRA_a OS=Homo sapiens GN=OGN PE=2 SV=1 |  |  |  |  |  |  |  |  |
| $43-3 | | 7 | 3 | 14.43% | 33922.3 | 5.3 | tr\|Q7Z532\|Q7Z532_HUMAN Osteoglycin OG OS=Homo sapiens PE=2 SV=1 |  |  |  |  |  |  |  |  |
| $43-4 | | 7 | 3 | 16.04% | 30456.5 | 8.5 | tr\|Q5TBF5\|Q5TBF5_HUMAN Mimecan (Fragment) OS=Homo sapiens GN=OGN PE=1 SV=1 |  |  |  |  |  |  |  |  |
| $43-5 | | 7 | 3 | 12.08% | 40553.1 | 8.1 | tr\|B4DI63\|B4DI63_HUMAN cDNA FLJ59205, highly similar to Mimecan OS=Homo sapiens PE=2 SV=1 |  |  |  |  |  |  |  |  |
|  | R16062_1_CTGF,13396 | K.DFADIPNLR.R | 1061.173 | 0.496 | 2 | 1 | 2.3582 | 0.3959 | 854.6 | 1 | 13\|16 | 4.21 | 1 | 5 |  |
|  | R16062_1_CTGF,13440 | K.DFADIPNLR.R | 1061.173 | -1.001 | 2 | 1 | 2.4467 | 0.161 | 525.2 | 3 | 11\|16 | 4.21 | 1 | 5 |  |
|  | R16062_1_CTGF,13445 | K.DFADIPNLR.R | 1061.173 | 0.422 | 2 | 1 | 2.3968 | 0.3537 | 760.9 | 1 | 12\|16 | 4.21 | 1 | 5 |  |
|  | R16062_1_CTGF,13456 | K.DFADIPNLR.R | 1061.173 | 0.162 | 2 | 1 | 2.3908 | 0.4004 | 586.5 | 2 | 11\|16 | 4.21 | 1 | 5 |  |
|  | R16062_1_CTGF,21442 | K.LSLLEELSLAENQLLK.L | 1814.1134 | 0.8824 | 2 | 1 | 3.1165 | 0.3528 | 1053.6 | 1 | 18\|30 | 4.25 | 1 | 5 |  |
|  | R16062_1_CTGF,20363 - 20364 | R.LDFTGNLIEDIEDGTFSK.L | 2015.1632 | 0.6642 | 2 | 1 | 2.5112 | 0.3837 | 530.5 | 1 | 15\|34 | 3.77 | 1 | 5 |  |
|  | R16062_1_CTGF,20398 | R.LDFTGNLIEDIEDGTFSK.L | 2015.1632 | 0.5142 | 2 | 1 | 2.5984 | 0.41 | 575.5 | 1 | 15\|34 | 3.77 | 1 | 5 |  |
| $44-1 | | 6 | 3 | 39.44% | 15257.5 | 8.7 | sp\|P69905\|HBA_HUMAN Hemoglobin subunit alpha OS=Homo sapiens GN=HBA1 PE=1 SV=2 |  |  |  |  |  |  |  |  |
| $44-2 | | 6 | 3 | 39.44% | 15257.5 | 8.7 | tr\|D1MGQ2\|D1MGQ2_HUMAN Alpha-2 globin chain OS=Homo sapiens GN=HBA2 PE=3 SV=1 |  |  |  |  |  |  |  |  |
| $44-3 | | 6 | 3 | 43.75% | 13582.5 | 8 | tr\|V9H1D9\|V9H1D9_HUMAN Alpha globin OS=Homo sapiens PE=3 SV=1 |  |  |  |  |  |  |  |  |
| $44-4 | | 6 | 3 | 39.44% | 15257.5 | 8.7 | tr\|A0A0K2BMD8\|A0A0K2BMD8_HUMAN Mutant hemoglobin alpha 2 globin chain OS=Homo sapiens GN=HBA2 PE=3 SV=1 |  |  |  |  |  |  |  |  |
|  | R16062_1_CTGF,13612 | K.FLASVSTVLTSK.Y | 1253.4698 | 0.6538 | 2 | 1 | 2.4864 | 0.4555 | 927.7 | 1 | 15\|22 | 8.75 | 1 | 4 |  |
|  | R16062_1_CTGF,13631 | K.FLASVSTVLTSK.Y | 1253.4698 | 0.2578 | 2 | 1 | 2.6497 | 0.4588 | 1034.1 | 1 | 15\|22 | 8.75 | 1 | 4 |  |
|  | R16062_1_CTGF,15455 | K.VADALTNAVAHVDDM*PNALSALSDLHAHK.L | 3014.3201 | -1.6099 | 3 | 1 | 4.3599 | 0.4915 | 871.7 | 1 | 27\|112 | 5.1 | 1 | 4 |  |
|  | R16062_1_CTGF,8311 | K.VGAHAGEYGAEALER.M | 1530.6241 | 0.4451 | 3 | 1 | 4.2603 | 0.2793 | 1815.7 | 2 | 27\|56 | 4.75 | 1 | 4 |  |
|  | R16062_1_CTGF,8409 | K.VGAHAGEYGAEALER.M | 1530.6241 | 2.1101 | 3 | 1 | 3.7634 | 0.293 | 3219.4 | 1 | 34\|56 | 4.75 | 1 | 4 |  |
|  | R16062_1_CTGF,8442 | K.VGAHAGEYGAEALER.M | 1530.6241 | 2.1051 | 3 | 1 | 3.8773 | 0.2314 | 2161.4 | 1 | 30\|56 | 4.75 | 1 | 4 |  |
| $45-1 | | 6 | 3 | 17.41% | 27324.8 | 9.4 | sp\|P23946\|CMA1_HUMAN Chymase OS=Homo sapiens GN=CMA1 PE=1 SV=1 |  |  |  |  |  |  |  |  |
|  | R16062_1_CTGF,14997 | K.GDSGGPLLCAGVAQGIVSYGR.S | 2035.2407 | 0.6407 | 2 | 1 | 3.1525 | 0.597 | 1223.9 | 1 | 20\|40 | 5.83 | 1 | 1 |  |
|  | R16062_1_CTGF,15026 | K.GDSGGPLLCAGVAQGIVSYGR.S | 2035.2407 | 0.6427 | 2 | 1 | 3.0546 | 0.5815 | 962.2 | 1 | 19\|40 | 5.83 | 1 | 1 |  |
|  | R16062_1_CTGF,9905 | R.NFVLTAAHCAGR.S | 1317.4724 | -1.1566 | 2 | 1 | 2.2869 | 0.3146 | 577.6 | 1 | 12\|22 | 8.26 | 1 | 1 |  |
|  | R16062_1_CTGF,9909 | R.NFVLTAAHCAGR.S | 1317.4724 | 1.1394 | 2 | 1 | 2.2925 | 0.3294 | 974.5 | 1 | 14\|22 | 8.26 | 1 | 1 |  |
|  | R16062_1_CTGF,9916 - 9917 | R.NFVLTAAHCAGR.S | 1317.4724 | 1.3184 | 2 | 1 | 2.4829 | 0.3226 | 578 | 1 | 12\|22 | 8.26 | 1 | 1 |  |
|  | R16062_1_CTGF,16979 | R.PWINQILQAN.- | 1197.368 | 0.007 | 2 | 1 | 2.5526 | 0.4151 | 503.6 | 1 | 13\|18 | 5.96 | 1 | 1 |  |
| $46-1 | | 6 | 3 | 15.90% | 35940.5 | 7.2 | sp\|P01861\|IGHG4_HUMAN Ig gamma-4 chain C region OS=Homo sapiens GN=IGHG4 PE=1 SV=1 |  |  |  |  |  |  |  |  |
| $46-2 | | 6 | 3 | 15.90% | 35940.5 | 7.2 | tr\|A0A0G2JPD4\|A0A0G2JPD4_HUMAN Ig gamma-4 chain C region (Fragment) OS=Homo sapiens GN=IGHG4 PE=1 SV=2 |  |  |  |  |  |  |  |  |
|  | R16062_1_CTGF,15825 | K.GFYPSDIAVEWESNGQPENNYK.T | 2545.6579 | -0.5081 | 2 | 1 | 3.3031 | 0.4501 | 953.6 | 1 | 18\|42 | 4 | 2 | 24 |  |
|  | R16062_1_CTGF,15834 | K.GFYPSDIAVEWESNGQPENNYK.T | 2545.6579 | 0.2199 | 2 | 1 | 3.174 | 0.4404 | 1392.9 | 1 | 22\|42 | 4 | 2 | 24 |  |
|  | R16062_1_CTGF,11499 | R.STSESTAALGCLVK.D | 1424.5731 | 0.9321 | 2 | 1 | 3.0647 | 0.4986 | 1026 | 1 | 18\|26 | 5.72 | 1 | 2 |  |
|  | R16062_1_CTGF,11508 | R.STSESTAALGCLVK.D | 1424.5731 | 0.9841 | 2 | 1 | 2.8232 | 0.3919 | 992.6 | 1 | 18\|26 | 5.72 | 1 | 2 |  |
|  | R16062_1_CTGF,18198 - 18199 | R.VVSVLTVLHQDWLNGK.E | 1809.1018 | -0.6892 | 2 | 1 | 3.2713 | 0.5131 | 607.2 | 1 | 13\|30 | 6.71 | 3 | 31 |  |
|  | R16062_1_CTGF,18208 | R.VVSVLTVLHQDWLNGK.E | 1809.1018 | 0.3478 | 2 | 1 | 2.4351 | 0.3916 | 1185.7 | 1 | 18\|30 | 6.71 | 3 | 31 |  |
| $47-1 | | 6 | 3 | 13.26% | 41287 | 8.2 | sp\|P01860\|IGHG3_HUMAN Ig gamma-3 chain C region OS=Homo sapiens GN=IGHG3 PE=1 SV=2 |  |  |  |  |  |  |  |  |
| $47-2 | | 6 | 3 | 9.67% | 56889.5 | 8.6 | tr\|A0A087WXL8\|A0A087WXL8_HUMAN Ig gamma-3 chain C region OS=Homo sapiens GN=IGHG3 PE=1 SV=1 |  |  |  |  |  |  |  |  |
| $47-3 | | 6 | 3 | 13.26% | 41215.9 | 8.2 | tr\|A0A075B6N8\|A0A075B6N8_HUMAN Ig gamma-3 chain C region (Fragment) OS=Homo sapiens GN=IGHG3 PE=1 SV=1 |  |  |  |  |  |  |  |  |
| $47-4 | | 6 | 3 | 9.60% | 57156 | 8.5 | tr\|A0A087WVW2\|A0A087WVW2_HUMAN Ig gamma-3 chain C region OS=Homo sapiens GN=IGHG3 PE=1 SV=1 |  |  |  |  |  |  |  |  |
| $47-5 | | 6 | 3 | 9.63% | 56813.2 | 6.4 | tr\|Q5EBM2\|Q5EBM2_HUMAN Uncharacterized protein OS=Homo sapiens PE=1 SV=1 |  |  |  |  |  |  |  |  |
| $47-6 | | 6 | 3 | 9.65% | 57019.5 | 8.4 | tr\|Q6N030\|Q6N030_HUMAN Putative uncharacterized protein DKFZp686I15212 OS=Homo sapiens GN=DKFZp686I15212 PE=1 SV=1 |  |  |  |  |  |  |  |  |
| $47-7 | | 6 | 3 | 9.82% | 56111 | 7.8 | tr\|Q8NF17\|Q8NF17_HUMAN FLJ00385 protein (Fragment) OS=Homo sapiens GN=FLJ00385 PE=1 SV=1 |  |  |  |  |  |  |  |  |
|  | R16062_1_CTGF,11202 | K.STSGGTAALGCLVK.D | 1322.484 | 1.915 | 2 | 1 | 2.5835 | 0.398 | 756.3 | 1 | 15\|26 | 7.94 | 2 | 29 |  |
|  | R16062_1_CTGF,17380 | R.CPAPELLGGPSVFLFPPKPK.D | 2152.5571 | -0.4389 | 3 | 1 | 3.7566 | 0.5482 | 810.2 | 1 | 28\|76 | 8.2 | 1 | 7 |  |
|  | R16062_1_CTGF,17433 | R.CPAPELLGGPSVFLFPPKPK.D | 2152.5571 | 1.1571 | 3 | 1 | 4.7605 | 0.6117 | 1417.8 | 1 | 33\|76 | 8.2 | 1 | 7 |  |
|  | R16062_1_CTGF,17485 | R.CPAPELLGGPSVFLFPPKPK.D | 2152.5571 | 1.1401 | 3 | 1 | 3.8919 | 0.4896 | 745 | 1 | 25\|76 | 8.2 | 1 | 7 |  |
|  | R16062_1_CTGF,18198 - 18199 | R.VVSVLTVLHQDWLNGK.E | 1809.1018 | -0.6892 | 2 | 1 | 3.2713 | 0.5131 | 607.2 | 1 | 13\|30 | 6.71 | 3 | 31 |  |
|  | R16062_1_CTGF,18208 | R.VVSVLTVLHQDWLNGK.E | 1809.1018 | 0.3478 | 2 | 1 | 2.4351 | 0.3916 | 1185.7 | 1 | 18\|30 | 6.71 | 3 | 31 |  |
| $48-1 | | 5 | 3 | 9.94% | 36688.6 | 8.4 | sp\|P00338\|LDHA_HUMAN L-lactate dehydrogenase A chain OS=Homo sapiens GN=LDHA PE=1 SV=2 |  |  |  |  |  |  |  |  |
| $48-2 | | 5 | 3 | 9.94% | 36688.6 | 8.4 | tr\|V9HWB9\|V9HWB9_HUMAN L-lactate dehydrogenase OS=Homo sapiens GN=HEL-S-133P PE=2 SV=1 |  |  |  |  |  |  |  |  |
| $48-3 | | 5 | 3 | 21.15% | 17259.2 | 8.9 | tr\|F5GXY2\|F5GXY2_HUMAN L-lactate dehydrogenase A chain (Fragment) OS=Homo sapiens GN=LDHA PE=1 SV=6 |  |  |  |  |  |  |  |  |
| $48-4 | | 5 | 3 | 28.95% | 12482.5 | 5.8 | tr\|F5GXH2\|F5GXH2_HUMAN L-lactate dehydrogenase A chain (Fragment) OS=Homo sapiens GN=LDHA PE=1 SV=1 |  |  |  |  |  |  |  |  |
| $48-5 | | 5 | 3 | 22.92% | 15845.5 | 6.6 | tr\|F5GYU2\|F5GYU2_HUMAN L-lactate dehydrogenase A chain (Fragment) OS=Homo sapiens GN=LDHA PE=1 SV=1 |  |  |  |  |  |  |  |  |
|  | R16062_1_CTGF,19446 | K.DLADELALVDVIEDK.L | 1658.8288 | 0.5678 | 2 | 1 | 3.2264 | 0.5286 | 1174.7 | 1 | 17\|28 | 3.66 | 1 | 5 |  |
|  | R16062_1_CTGF,16117 | K.DQLIYNLLK.E | 1120.3235 | 0.3955 | 2 | 1 | 2.3352 | 0.1494 | 748 | 1 | 13\|16 | 5.84 | 1 | 5 |  |
|  | R16062_1_CTGF,16129 | K.DQLIYNLLK.E | 1120.3235 | -1.0585 | 2 | 1 | 2.3647 | 0.1641 | 931.7 | 1 | 13\|16 | 5.84 | 1 | 5 |  |
|  | R16062_1_CTGF,16140 | K.DQLIYNLLK.E | 1120.3235 | 0.7065 | 2 | 1 | 2.4075 | 0.2231 | 718.4 | 4 | 11\|16 | 5.84 | 1 | 5 |  |
|  | R16062_1_CTGF,10249 | K.LVIITAGAR.Q | 914.1277 | 0.0937 | 2 | 1 | 2.36 | 0.3752 | 904.2 | 1 | 12\|16 | 9.75 | 1 | 5 |  |
| $49-1 | | 5 | 3 | 3.56% | 151077 | 9.5 | sp\|Q92954\|PRG4_HUMAN Proteoglycan 4 OS=Homo sapiens GN=PRG4 PE=1 SV=2 |  |  |  |  |  |  |  |  |
| $49-2 | | 5 | 3 | 3.56% | 151077 | 9.5 | tr\|A0A024R930\|A0A024R930_HUMAN Proteoglycan 4, isoform CRA_a OS=Homo sapiens GN=PRG4 PE=4 SV=1 |  |  |  |  |  |  |  |  |
| $49-3 | | 5 | 3 | 10.12% | 55494.3 | 9.9 | tr\|B3KQ20\|B3KQ20_HUMAN cDNA FLJ32635 fis, clone SYNOV2000178, highly similar to Proteoglycan-4 OS=Homo sapiens PE=2 SV=1 |  |  |  |  |  |  |  |  |
|  | R16062_1_CTGF,19128 | K.GFGGLTGQIVAALSTAK.Y | 1591.8336 | 1.1686 | 2 | 1 | 2.8965 | 0.5755 | 1714.6 | 1 | 20\|32 | 8.75 | 1 | 3 |  |
|  | R16062_1_CTGF,19183 | K.GFGGLTGQIVAALSTAK.Y | 1591.8336 | 1.5986 | 2 | 1 | 3.367 | 0.6178 | 1265.9 | 1 | 19\|32 | 8.75 | 1 | 3 |  |
|  | R16062_1_CTGF,16861 | R.GLPNVVTSAISLPNIR.K | 1651.9322 | -0.9508 | 2 | 1 | 2.8949 | 0.512 | 344.3 | 1 | 14\|30 | 9.75 | 1 | 3 |  |
|  | R16062_1_CTGF,16893 | R.GLPNVVTSAISLPNIR.K | 1651.9322 | 0.8702 | 2 | 1 | 2.376 | 0.3518 | 569.1 | 1 | 15\|30 | 9.75 | 1 | 3 |  |
|  | R16062_1_CTGF,19519 | R.ITEVWGIPSPIDTVFTR.C | 1932.2085 | -0.0215 | 2 | 1 | 2.3862 | 0.4637 | 229.7 | 1 | 13\|32 | 4.37 | 1 | 3 |  |
| $50-1 | | 5 | 3 | 0.92% | 531793 | 5.7 | sp\|Q15149\|PLEC_HUMAN Plectin OS=Homo sapiens GN=PLEC PE=1 SV=3 |  |  |  |  |  |  |  |  |
| $50-2 | | 5 | 3 | 6.12% | 80780.6 | 6.3 | tr\|E9PMV1\|E9PMV1_HUMAN Plectin (Fragment) OS=Homo sapiens GN=PLEC PE=1 SV=1 |  |  |  |  |  |  |  |  |
|  | R16062_1_CTGF,15256 | K.GIYQSLEGAVQAGQLK.V | 1662.8687 | -0.1473 | 2 | 1 | 2.5131 | 0.2993 | 558.7 | 2 | 14\|30 | 6 | 1 | 2 |  |
|  | R16062_1_CTGF,16038 | K.LQNVQIALDYLR.H | 1446.6765 | 1.3375 | 2 | 1 | 2.5408 | 0.4462 | 756.1 | 1 | 13\|22 | 5.84 | 1 | 2 |  |
|  | R16062_1_CTGF,16082 | K.LQNVQIALDYLR.H | 1446.6765 | 1.5605 | 2 | 1 | 2.3211 | 0.2482 | 917.9 | 1 | 14\|22 | 5.84 | 1 | 2 |  |
|  | R16062_1_CTGF,12010 | R.LLDPEDVDVPQPDEK.S | 1709.8326 | 1.3936 | 2 | 1 | 2.2361 | 0.2683 | 667.9 | 1 | 17\|28 | 3.66 | 1 | 2 |  |
|  | R16062_1_CTGF,12037 | R.LLDPEDVDVPQPDEK.S | 1709.8326 | 0.3236 | 2 | 1 | 2.2787 | 0.272 | 630.2 | 2 | 14\|28 | 3.66 | 1 | 2 |  |
| $51-1 | | 5 | 3 | 5.39% | 83264.4 | 5 | sp\|P08238\|HS90B_HUMAN Heat shock protein HSP 90-beta OS=Homo sapiens GN=HSP90AB1 PE=1 SV=4 |  |  |  |  |  |  |  |  |
| $51-2 | | 5 | 3 | 5.39% | 83264.4 | 5 | tr\|A0A024RD80\|A0A024RD80_HUMAN Heat shock protein 90kDa alpha (Cytosolic), class B member 1, isoform CRA_a OS=Homo sapiens GN=HSP90AB1 PE=3 SV=1 |  |  |  |  |  |  |  |  |
| $51-3 | | 5 | 3 | 5.46% | 82170.1 | 5 | tr\|B4DGL0\|B4DGL0_HUMAN cDNA FLJ53619, highly similar to Heat shock protein HSP 90-beta OS=Homo sapiens PE=2 SV=1 |  |  |  |  |  |  |  |  |
| $51-4 | | 5 | 3 | 5.69% | 79195 | 5 | tr\|B4DMA2\|B4DMA2_HUMAN cDNA FLJ54023, highly similar to Heat shock protein HSP 90-beta OS=Homo sapiens PE=2 SV=1 |  |  |  |  |  |  |  |  |
|  | R16062_1_CTGF,10101 - 10102 | R.ELISNASDALDK.I | 1276.3755 | 0.3325 | 2 | 1 | 2.4943 | 0.3724 | 667.3 | 1 | 13\|22 | 4.03 | 2 | 7 |  |
|  | R16062_1_CTGF,10115 | R.ELISNASDALDK.I | 1276.3755 | 0.8545 | 2 | 1 | 2.6074 | 0.4474 | 706.3 | 1 | 14\|22 | 4.03 | 2 | 7 |  |
|  | R16062_1_CTGF,13228 | R.GVVDSEDIPLNLSR.E ! R.GVVDSEDLPLNISR.E | 1514.6629 | 1.6369 | 2 | 1 | 2.768 | 0.5211 | 513 | 1 | 14\|26 | 4.03 | 1 | 4 |  |
|  | R16062_1_CTGF,13248 | R.GVVDSEDIPLNLSR.E ! R.GVVDSEDLPLNISR.E | 1514.6629 | 0.7379 | 2 | 1 | 2.3463 | 0.3642 | 370.3 | 3 | 13\|26 | 4.03 | 1 | 4 |  |
|  | R16062_1_CTGF,12171 | R.TLTIVDTGIGM*TK.A ! R.TLTLVDTGIGM*TK.A | 1366.6067 | 0.8227 | 2 | 1 | 2.3178 | 0.4278 | 505 | 1 | 13\|24 | 5.5 | 1 | 4 |  |
| $52-1 | | 4 | 3 | 8.66% | 53154.2 | 5.9 | sp\|P01019\|ANGT_HUMAN Angiotensinogen OS=Homo sapiens GN=AGT PE=1 SV=1 |  |  |  |  |  |  |  |  |
| $52-2 | | 4 | 3 | 8.66% | 53154.2 | 5.9 | tr\|B0ZBE2\|B0ZBE2_HUMAN Angiotensinogen (Serpin peptidase inhibitor, clade A, member 8) OS=Homo sapiens GN=AGT PE=3 SV=1 |  |  |  |  |  |  |  |  |
| $52-3 | | 4 | 3 | 8.66% | 53124.1 | 5.9 | tr\|Q53YY1\|Q53YY1_HUMAN Angiotensinogen (Serine (Or cysteine) proteinase inhibitor, clade A (Alpha-1 antiproteinase, antitrypsin), member 8) OS=Homo sapiens GN=AGT PE=2 SV=1 |  |  |  |  |  |  |  |  |
| $52-4 | | 4 | 3 | 8.66% | 53084.1 | 5.8 | tr\|Q53GY3\|Q53GY3_HUMAN Angiotensinogen variant (Fragment) OS=Homo sapiens PE=2 SV=1 |  |  |  |  |  |  |  |  |
| $52-5 | | 4 | 3 | 8.55% | 53777.9 | 6 | tr\|Q59EP2\|Q59EP2_HUMAN Angiotensinogen variant (Fragment) OS=Homo sapiens PE=2 SV=1 |  |  |  |  |  |  |  |  |
| $52-6 | | 4 | 3 | 9.01% | 51062.7 | 5.8 | tr\|B4E1B3\|B4E1B3_HUMAN cDNA FLJ53950, highly similar to Angiotensinogen OS=Homo sapiens PE=2 SV=1 |  |  |  |  |  |  |  |  |
| $52-7 | | 4 | 3 | 8.66% | 53114.1 | 5.9 | tr\|Q86U78\|Q86U78_HUMAN Angiotensinogen (Serine (Or cysteine) proteinase inhibitor, clade A (Alpha-1 antiproteinase, antitrypsin), member 8) OS=Homo sapiens PE=2 SV=1 |  |  |  |  |  |  |  |  |
|  | R16062_1_CTGF,13176 | K.ALQDQLVLVAAK.L | 1269.5156 | 0.5156 | 2 | 1 | 2.3338 | 0.3654 | 962.5 | 1 | 16\|22 | 5.88 | 1 | 7 |  |
|  | R16062_1_CTGF,13193 | K.ALQDQLVLVAAK.L | 1269.5156 | 0.4676 | 2 | 1 | 2.6291 | 0.4018 | 773.9 | 1 | 13\|22 | 5.88 | 1 | 7 |  |
|  | R16062_1_CTGF,19607 | K.VLSALQAVQGLLVAQGR.A | 1724.0415 | 0.6755 | 3 | 1 | 4.3085 | 0.5602 | 1994.3 | 1 | 33\|64 | 9.72 | 1 | 7 |  |
|  | R16062_1_CTGF,14549 | R.SLDFTELDVAAEK.I | 1438.5616 | -0.0304 | 2 | 1 | 2.2306 | 0.3175 | 514.9 | 2 | 13\|24 | 3.92 | 1 | 7 |  |
| $53-1 | | 4 | 3 | 6.81% | 61054.4 | 5.7 | sp\|P10809\|CH60_HUMAN 60 kDa heat shock protein, mitochondrial OS=Homo sapiens GN=HSPD1 PE=1 SV=2 |  |  |  |  |  |  |  |  |
| $53-2 | | 4 | 3 | 6.81% | 61054.4 | 5.7 | tr\|A0A024R3X4\|A0A024R3X4_HUMAN Heat shock 60kDa protein 1 (Chaperonin), isoform CRA_a OS=Homo sapiens GN=HSPD1 PE=3 SV=1 |  |  |  |  |  |  |  |  |
| $53-3 | | 4 | 3 | 7.09% | 58570.7 | 5.8 | tr\|B7Z4F6\|B7Z4F6_HUMAN cDNA FLJ54912, highly similar to 60 kDa heat shock protein, mitochondrial OS=Homo sapiens PE=2 SV=1 |  |  |  |  |  |  |  |  |
| $53-4 | | 4 | 3 | 7.54% | 55083.4 | 5.5 | tr\|B7Z5E7\|B7Z5E7_HUMAN cDNA FLJ51046, highly similar to 60 kDa heat shock protein, mitochondrial OS=Homo sapiens PE=2 SV=1 |  |  |  |  |  |  |  |  |
| $53-5 | | 4 | 3 | 6.85% | 60680 | 5.8 | tr\|B3GQS7\|B3GQS7_HUMAN Mitochondrial heat shock 60kD protein 1 variant 1 OS=Homo sapiens GN=HSPD1 PE=2 SV=1 |  |  |  |  |  |  |  |  |
| $53-6 | | 4 | 3 | 6.91% | 60047.3 | 5.6 | tr\|B7Z597\|B7Z597_HUMAN cDNA FLJ54373, highly similar to 60 kDa heat shock protein, mitochondrial OS=Homo sapiens PE=2 SV=1 |  |  |  |  |  |  |  |  |
|  | R16062_1_CTGF,14736 | K.TLNDELEIIEGM*K.F | 1521.7156 | 0.5136 | 2 | 1 | 2.5625 | 0.4462 | 940.6 | 1 | 14\|24 | 4 | 1 | 6 |  |
|  | R16062_1_CTGF,15147 | R.AAVEEGIVLGGGCALLR.C | 1685.9404 | 0.6964 | 2 | 1 | 2.589 | 0.236 | 740.7 | 1 | 16\|32 | 4.53 | 1 | 6 |  |
|  | R16062_1_CTGF,6372 | R.VTDALNATR.A | 961.055 | 0.004 | 2 | 1 | 2.4399 | 0.3717 | 1083.1 | 1 | 13\|16 | 5.81 | 1 | 6 |  |
|  | R16062_1_CTGF,6440 | R.VTDALNATR.A | 961.055 | 0.129 | 2 | 1 | 2.3171 | 0.3953 | 939.5 | 1 | 12\|16 | 5.81 | 1 | 6 |  |
| $54-1 | | 3 | 3 | 2.35% | 189252 | 6.1 | sp\|P46940\|IQGA1_HUMAN Ras GTPase-activating-like protein IQGAP1 OS=Homo sapiens GN=IQGAP1 PE=1 SV=1 |  |  |  |  |  |  |  |  |
| $54-2 | | 3 | 3 | 3.59% | 124793 | 8.8 | tr\|H0YLE8\|H0YLE8_HUMAN Ras GTPase-activating-like protein IQGAP1 OS=Homo sapiens GN=IQGAP1 PE=1 SV=1 |  |  |  |  |  |  |  |  |
| $54-3 | | 3 | 3 | 2.35% | 189280 | 6.1 | tr\|A4QPB0\|A4QPB0_HUMAN IQ motif containing GTPase activating protein 1 OS=Homo sapiens GN=IQGAP1 PE=1 SV=1 |  |  |  |  |  |  |  |  |
| $54-4 | | 3 | 3 | 3.59% | 124624 | 8.5 | tr\|B4E2M0\|B4E2M0_HUMAN cDNA FLJ55443, highly similar to Ras GTPase-activating-like protein IQGAP1 OS=Homo sapiens PE=2 SV=1 |  |  |  |  |  |  |  |  |
| $54-5 | | 3 | 3 | 2.35% | 189252 | 6.1 | tr\|A0A024RC65\|A0A024RC65_HUMAN HCG1991735, isoform CRA_a OS=Homo sapiens GN=hCG_1991735 PE=4 SV=1 |  |  |  |  |  |  |  |  |
|  | R16062_1_CTGF,14446 | K.IIGNLLYYR.Y | 1125.345 | -0.855 | 2 | 1 | 2.3627 | 0.4281 | 612.5 | 1 | 11\|16 | 8.59 | 1 | 5 |  |
|  | R16062_1_CTGF,13802 | K.VDQIQEIVTGNPTVIK.M | 1755.006 | 0.384 | 2 | 1 | 2.8828 | 0.418 | 992.9 | 1 | 18\|30 | 4.37 | 1 | 5 |  |
|  | R16062_1_CTGF,12623 | R.FFQTACDVPELQDK.F | 1698.8481 | 0.0641 | 2 | 1 | 2.6363 | 0.5391 | 857.6 | 1 | 16\|26 | 4.03 | 1 | 5 |  |
| $55-1 | | 3 | 3 | 7.00% | 55154.1 | 6.1 | sp\|P05155\|IC1_HUMAN Plasma protease C1 inhibitor OS=Homo sapiens GN=SERPING1 PE=1 SV=2 |  |  |  |  |  |  |  |  |
| $55-2 | | 3 | 3 | 6.45% | 59492 | 6.3 | tr\|E9PGN7\|E9PGN7_HUMAN Plasma protease C1 inhibitor OS=Homo sapiens GN=SERPING1 PE=1 SV=1 |  |  |  |  |  |  |  |  |
| $55-3 | | 3 | 3 | 7.00% | 55154.1 | 6.1 | tr\|E9KL26\|E9KL26_HUMAN Epididymis tissue protein Li 173 OS=Homo sapiens PE=2 SV=1 |  |  |  |  |  |  |  |  |
| $55-4 | | 3 | 3 | 10.51% | 37288 | 7.9 | tr\|Q5UGI6\|Q5UGI6_HUMAN Serine/cysteine proteinase inhibitor clade G member 1 splice variant 2 (Fragment) OS=Homo sapiens GN=SERPING1 PE=2 SV=1 |  |  |  |  |  |  |  |  |
|  | R16062_1_CTGF,14913 | K.TNLESILSYPK.D | 1265.4375 | 0.1955 | 2 | 1 | 2.2277 | 0.3128 | 432 | 1 | 11\|20 | 5.66 | 1 | 4 |  |
|  | R16062_1_CTGF,12374 | R.LEDM*EQALSPSVFK.A | 1610.8108 | 0.5938 | 2 | 1 | 2.5352 | 0.42 | 1187.6 | 1 | 17\|26 | 4.14 | 1 | 4 |  |
|  | R16062_1_CTGF,10057 | R.LLDSLPSDTR.L | 1117.2349 | 0.2259 | 2 | 1 | 2.2998 | 0.3655 | 457.7 | 1 | 13\|18 | 4.21 | 1 | 4 |  |
| $56-1 | | 3 | 3 | 6.22% | 70288.4 | 5.3 | sp\|P13796\|PLSL_HUMAN Plastin-2 OS=Homo sapiens GN=LCP1 PE=1 SV=6 |  |  |  |  |  |  |  |  |
| $56-2 | | 3 | 3 | 6.22% | 70289.3 | 5.2 | tr\|V9HWJ7\|V9HWJ7_HUMAN Epididymis secretory protein Li 37 OS=Homo sapiens GN=HEL-S-37 PE=2 SV=1 |  |  |  |  |  |  |  |  |
| $56-3 | | 3 | 3 | 6.22% | 70259.3 | 5.2 | tr\|Q53FI1\|Q53FI1_HUMAN L-plastin variant (Fragment) OS=Homo sapiens PE=2 SV=1 |  |  |  |  |  |  |  |  |
| $56-4 | | 3 | 3 | 6.22% | 70288.4 | 5.3 | tr\|A0A024RDT4\|A0A024RDT4_HUMAN Lymphocyte cytosolic protein 1 (L-plastin), isoform CRA_a OS=Homo sapiens GN=LCP1 PE=4 SV=1 |  |  |  |  |  |  |  |  |
|  | R16062_1_CTGF,19983 | K.VNDDIIVNWVNETLR.E | 1800.9934 | -1.0126 | 2 | 1 | 3.4646 | 0.4252 | 771.5 | 1 | 14\|28 | 4.03 | 1 | 4 |  |
|  | R16062_1_CTGF,10344 | R.QFVTATDVVR.G | 1136.283 | -0.255 | 2 | 1 | 2.2275 | 0.1438 | 867 | 2 | 12\|18 | 5.84 | 1 | 4 |  |
|  | R16062_1_CTGF,16960 | R.YTLNILEEIGGGQK.V | 1535.7237 | 0.0377 | 2 | 1 | 2.4008 | 0.2048 | 371.8 | 6 | 11\|26 | 4.53 | 1 | 4 |  |
| $57-1 | | 7 | 2 | 7.98% | 43178.9 | 5.7 | sp\|Q06828\|FMOD_HUMAN Fibromodulin OS=Homo sapiens GN=FMOD PE=1 SV=2 |  |  |  |  |  |  |  |  |
| $57-2 | | 7 | 2 | 10.45% | 32117 | 9.3 | tr\|B3KS64\|B3KS64_HUMAN cDNA FLJ35580 fis, clone SPLEN2006389, highly similar to FIBROMODULIN OS=Homo sapiens PE=2 SV=1 |  |  |  |  |  |  |  |  |
| $57-3 | | 7 | 2 | 8.29% | 41495 | 5.6 | tr\|B4E1J3\|B4E1J3_HUMAN cDNA FLJ53615, highly similar to Fibromodulin OS=Homo sapiens PE=2 SV=1 |  |  |  |  |  |  |  |  |
| $57-4 | | 7 | 2 | 7.98% | 43178.9 | 5.7 | tr\|A0A024R971\|A0A024R971_HUMAN Fibromodulin, isoform CRA_a OS=Homo sapiens GN=FMOD PE=4 SV=1 |  |  |  |  |  |  |  |  |
|  | R16062_1_CTGF,14548 | K.IPPVNTNLENLYLQGNR.I | 1956.1917 | 0.7457 | 2 | 1 | 2.3336 | 0.4589 | 235.2 | 1 | 15\|32 | 6 | 1 | 4 |  |
|  | R16062_1_CTGF,14571 | K.IPPVNTNLENLYLQGNR.I | 1956.1917 | -0.3603 | 2 | 1 | 2.9711 | 0.4334 | 330.2 | 1 | 16\|32 | 6 | 1 | 4 |  |
|  | R16062_1_CTGF,14582 | K.IPPVNTNLENLYLQGNR.I | 1956.1917 | 0.8397 | 2 | 1 | 2.4814 | 0.4512 | 337.3 | 1 | 15\|32 | 6 | 1 | 4 |  |
|  | R16062_1_CTGF,14590 | K.IPPVNTNLENLYLQGNR.I | 1956.1917 | 0.8527 | 2 | 1 | 2.3409 | 0.4019 | 368.9 | 1 | 17\|32 | 6 | 1 | 4 |  |
|  | R16062_1_CTGF,16814 | R.SLILLDLSYNHLR.K | 1557.8189 | 0.2929 | 2 | 1 | 2.655 | 0.227 | 1265.9 | 1 | 16\|24 | 6.46 | 1 | 4 |  |
|  | R16062_1_CTGF,16821 | R.SLILLDLSYNHLR.K | 1557.8189 | -0.5571 | 2 | 1 | 2.3989 | 0.2888 | 755.5 | 1 | 13\|24 | 6.46 | 1 | 4 |  |
|  | R16062_1_CTGF,16848 | R.SLILLDLSYNHLR.K | 1557.8189 | 0.4729 | 2 | 1 | 2.5002 | 0.1663 | 794 | 1 | 14\|24 | 6.46 | 1 | 4 |  |
| $58-1 | | 6 | 2 | 23.58% | 11293.6 | 6.9 | sp\|P0CG05\|LAC2_HUMAN Ig lambda-2 chain C regions OS=Homo sapiens GN=IGLC2 PE=1 SV=1 |  |  |  |  |  |  |  |  |
| $58-2 | | 6 | 2 | 23.58% | 11237.5 | 6.9 | sp\|P0CG06\|LAC3_HUMAN Ig lambda-3 chain C regions OS=Homo sapiens GN=IGLC3 PE=1 SV=1 |  |  |  |  |  |  |  |  |
| $58-3 | | 6 | 2 | 10.59% | 25020.9 | 8.1 | tr\|Q6PIQ7\|Q6PIQ7_HUMAN IGL@ protein OS=Homo sapiens GN=IGL@ PE=1 SV=1 |  |  |  |  |  |  |  |  |
| $58-4 | | 6 | 2 | 17.99% | 14678.4 | 6.3 | tr\|Q6PJR7\|Q6PJR7_HUMAN Beta-2-microglobulin OS=Homo sapiens GN=IGL@ PE=1 SV=1 |  |  |  |  |  |  |  |  |
| $58-5 | | 6 | 2 | 10.68% | 24653.6 | 5.9 | tr\|Q6GMW3\|Q6GMW3_HUMAN IGL@ protein OS=Homo sapiens GN=IGL@ PE=1 SV=1 |  |  |  |  |  |  |  |  |
| $58-6 | | 6 | 2 | 10.64% | 24823.7 | 6.8 | tr\|S6BGD6\|S6BGD6_HUMAN IgG L chain OS=Homo sapiens PE=1 SV=1 |  |  |  |  |  |  |  |  |
| $58-7 | | 6 | 2 | 10.78% | 24741.7 | 5.4 | tr\|C6KXN3\|C6KXN3_HUMAN Lambda light chain of human immunoglobulin surface antigen-related protein (Fragment) OS=Homo sapiens GN=IgLC-rG PE=1 SV=1 |  |  |  |  |  |  |  |  |
| $58-8 | | 6 | 2 | 11.74% | 22742.8 | 8.2 | tr\|S6BGF9\|S6BGF9_HUMAN IgG L chain OS=Homo sapiens PE=2 SV=1 |  |  |  |  |  |  |  |  |
| $58-9 | | 6 | 2 | 11.57% | 23121.8 | 7.6 | tr\|S6BAR0\|S6BAR0_HUMAN IgG L chain OS=Homo sapiens PE=2 SV=1 |  |  |  |  |  |  |  |  |
| $58-10 | | 6 | 2 | 10.64% | 24799.6 | 6.9 | tr\|Q567P1\|Q567P1_HUMAN IGL@ protein OS=Homo sapiens GN=IGL@ PE=2 SV=1 |  |  |  |  |  |  |  |  |
| $58-11 | | 6 | 2 | 11.52% | 23117 | 8.8 | tr\|S6B2B0\|S6B2B0_HUMAN IgG L chain OS=Homo sapiens PE=2 SV=1 |  |  |  |  |  |  |  |  |
| $58-12 | | 6 | 2 | 10.64% | 24654.5 | 7.5 | tr\|A2NUT2\|A2NUT2_HUMAN Lambda-chain (AA -20 to 215) OS=Homo sapiens PE=1 SV=1 |  |  |  |  |  |  |  |  |
| $58-13 | | 6 | 2 | 11.57% | 22919.7 | 8.6 | tr\|S6BAN6\|S6BAN6_HUMAN IgG L chain OS=Homo sapiens PE=2 SV=1 |  |  |  |  |  |  |  |  |
| $58-14 | | 6 | 2 | 10.64% | 24887.8 | 6.3 | tr\|Q6IN99\|Q6IN99_HUMAN IGL@ protein OS=Homo sapiens GN=IGL@ PE=2 SV=1 |  |  |  |  |  |  |  |  |
| $58-15 | | 6 | 2 | 11.52% | 22860.7 | 8.3 | tr\|S6C4S2\|S6C4S2_HUMAN IgG L chain OS=Homo sapiens PE=2 SV=1 |  |  |  |  |  |  |  |  |
| $58-16 | | 6 | 2 | 10.68% | 24792.6 | 5.9 | tr\|Q8N355\|Q8N355_HUMAN IGL@ protein OS=Homo sapiens GN=IGL@ PE=1 SV=1 |  |  |  |  |  |  |  |  |
| $58-17 | | 6 | 2 | 10.59% | 25024 | 7.6 | tr\|Q8NEJ1\|Q8NEJ1_HUMAN Uncharacterized protein OS=Homo sapiens PE=2 SV=1 |  |  |  |  |  |  |  |  |
| $58-18 | | 6 | 2 | 10.59% | 24700.5 | 6.4 | tr\|Q6GMX3\|Q6GMX3_HUMAN IGL@ protein OS=Homo sapiens GN=IGL@ PE=2 SV=1 |  |  |  |  |  |  |  |  |
| $58-19 | | 6 | 2 | 11.63% | 22905.9 | 8.7 | tr\|S6AWE6\|S6AWE6_HUMAN IgG L chain OS=Homo sapiens PE=2 SV=1 |  |  |  |  |  |  |  |  |
| $58-20 | | 6 | 2 | 10.59% | 24823.6 | 6.3 | tr\|Q6IPQ0\|Q6IPQ0_HUMAN IGL@ protein OS=Homo sapiens GN=IGL@ PE=2 SV=1 |  |  |  |  |  |  |  |  |
| $58-21 | | 6 | 2 | 11.63% | 22871.8 | 8.7 | tr\|S6B286\|S6B286_HUMAN IgG L chain OS=Homo sapiens PE=2 SV=1 |  |  |  |  |  |  |  |  |
| $58-22 | | 6 | 2 | 10.64% | 24746.7 | 5.7 | tr\|A0A5E4\|A0A5E4_HUMAN Uncharacterized protein OS=Homo sapiens PE=2 SV=1 |  |  |  |  |  |  |  |  |
| $58-23 | | 6 | 2 | 10.73% | 24960.8 | 5.2 | tr\|Q8N5F4\|Q8N5F4_HUMAN IGL@ protein OS=Homo sapiens GN=IGL@ PE=1 SV=1 |  |  |  |  |  |  |  |  |
| $58-24 | | 6 | 2 | 10.68% | 25014.9 | 5.9 | tr\|Q7Z2U7\|Q7Z2U7_HUMAN Uncharacterized protein OS=Homo sapiens PE=2 SV=1 |  |  |  |  |  |  |  |  |
| $58-25 | | 6 | 2 | 10.64% | 24632.4 | 6.9 | tr\|Q6PJG0\|Q6PJG0_HUMAN Uncharacterized protein OS=Homo sapiens PE=1 SV=1 |  |  |  |  |  |  |  |  |
| $58-26 | | 6 | 2 | 23.58% | 11236.5 | 6.9 | tr\|A0A075B6K9\|A0A075B6K9_HUMAN Ig lambda-2 chain C regions (Fragment) OS=Homo sapiens GN=IGLC2 PE=4 SV=1 |  |  |  |  |  |  |  |  |
| $58-27 | | 6 | 2 | 10.68% | 24909.8 | 6.2 | tr\|Q6GMV8\|Q6GMV8_HUMAN Uncharacterized protein OS=Homo sapiens PE=2 SV=1 |  |  |  |  |  |  |  |  |
| $58-28 | | 6 | 2 | 10.59% | 24809.7 | 6.4 | tr\|Q6GMX4\|Q6GMX4_HUMAN IGL@ protein OS=Homo sapiens GN=IGL@ PE=1 SV=1 |  |  |  |  |  |  |  |  |
| $58-29 | | 6 | 2 | 23.58% | 11208.5 | 6.9 | tr\|A0A075B6L0\|A0A075B6L0_HUMAN Ig lambda-3 chain C regions (Fragment) OS=Homo sapiens GN=IGLC3 PE=1 SV=2 |  |  |  |  |  |  |  |  |
| $58-30 | | 6 | 2 | 11.52% | 22851.6 | 8.2 | tr\|S6BAP8\|S6BAP8_HUMAN IgG L chain OS=Homo sapiens PE=2 SV=1 |  |  |  |  |  |  |  |  |
| $58-31 | | 6 | 2 | 23.58% | 11265.5 | 6.9 | tr\|Q8TCJ5\|Q8TCJ5_HUMAN Putative uncharacterized protein DKFZp667J0810 (Fragment) OS=Homo sapiens GN=DKFZp667J0810 PE=2 SV=1 |  |  |  |  |  |  |  |  |
|  | R16062_1_CTGF,4584 | K.AGVETTTPSK.Q | 991.0778 | -0.0242 | 2 | 1 | 2.9199 | 0.2802 | 883.3 | 2 | 13\|18 | 6.05 | 1 | 31 |  |
|  | R16062_1_CTGF,4591 | K.AGVETTTPSK.Q | 991.0778 | -0.0172 | 2 | 1 | 2.4962 | 0.3223 | 1026.4 | 1 | 15\|18 | 6.05 | 1 | 31 |  |
|  | R16062_1_CTGF,4667 | K.AGVETTTPSK.Q | 991.0778 | 1.0958 | 2 | 1 | 2.4592 | 0.4128 | 706.4 | 1 | 13\|18 | 6.05 | 1 | 31 |  |
|  | R16062_1_CTGF,15360 | K.YAASSYLSLTPEQWK.S | 1744.925 | -0.349 | 2 | 1 | 3.0426 | 0.4703 | 1147 | 1 | 18\|28 | 6 | 1 | 31 |  |
|  | R16062_1_CTGF,15381 | K.YAASSYLSLTPEQWK.S | 1744.925 | 0.405 | 2 | 1 | 2.7109 | 0.4981 | 847.6 | 1 | 15\|28 | 6 | 1 | 31 |  |
|  | R16062_1_CTGF,15404 | K.YAASSYLSLTPEQWK.S | 1744.925 | 1.558 | 2 | 1 | 2.5128 | 0.4842 | 845.4 | 1 | 16\|28 | 6 | 1 | 31 |  |
| $59-1 | | 5 | 2 | 7.37% | 43417.2 | 6.6 | sp\|Q9BXN1\|ASPN_HUMAN Asporin OS=Homo sapiens GN=ASPN PE=1 SV=2 |  |  |  |  |  |  |  |  |
| $59-2 | | 5 | 2 | 7.29% | 43891.6 | 6 | tr\|Q6P528\|Q6P528_HUMAN ASPN protein OS=Homo sapiens GN=ASPN PE=2 SV=1 |  |  |  |  |  |  |  |  |
|  | R16062_1_CTGF,17472 | K.GLTSLYGLILNNNK.L | 1520.7554 | -1.0136 | 2 | 1 | 2.8836 | 0.3276 | 686.8 | 1 | 14\|26 | 8.59 | 1 | 2 |  |
|  | R16062_1_CTGF,17540 | K.GLTSLYGLILNNNK.L | 1520.7554 | 0.9264 | 2 | 1 | 3.0102 | 0.3942 | 1233.8 | 1 | 17\|26 | 8.59 | 1 | 2 |  |
|  | R16062_1_CTGF,17564 | K.GLTSLYGLILNNNK.L | 1520.7554 | 1.3714 | 2 | 1 | 2.8178 | 0.3466 | 725.5 | 1 | 13\|26 | 8.59 | 1 | 2 |  |
|  | R16062_1_CTGF,17401 | K.SLYSAISLFNNPVK.Y | 1553.7838 | 1.3218 | 2 | 1 | 2.4794 | 0.3939 | 865.1 | 1 | 16\|26 | 8.31 | 1 | 2 |  |
|  | R16062_1_CTGF,17439 | K.SLYSAISLFNNPVK.Y | 1553.7838 | 0.0858 | 2 | 1 | 2.82 | 0.5213 | 731.6 | 1 | 15\|26 | 8.31 | 1 | 2 |  |
| $60-1 | | 5 | 2 | 6.49% | 51676.4 | 6.6 | sp\|P02790\|HEMO_HUMAN Hemopexin OS=Homo sapiens GN=HPX PE=1 SV=2 |  |  |  |  |  |  |  |  |
|  | R16062_1_CTGF,18287 | K.LYLVQGTQVYVFLTK.G | 1773.1078 | 0.4448 | 2 | 1 | 2.7427 | 0.4189 | 723.9 | 1 | 16\|28 | 8.5 | 1 | 1 |  |
|  | R16062_1_CTGF,14429 | R.GECQAEGVLFFQGDR.E | 1713.8229 | -0.5891 | 2 | 1 | 3.0085 | 0.5034 | 603.3 | 1 | 14\|28 | 4.14 | 1 | 1 |  |
|  | R16062_1_CTGF,14439 | R.GECQAEGVLFFQGDR.E | 1713.8229 | -0.7121 | 2 | 1 | 2.5018 | 0.3596 | 1053 | 1 | 16\|28 | 4.14 | 1 | 1 |  |
|  | R16062_1_CTGF,14458 | R.GECQAEGVLFFQGDR.E | 1713.8229 | 1.1649 | 2 | 1 | 2.3759 | 0.2884 | 1770.4 | 1 | 19\|28 | 4.14 | 1 | 1 |  |
|  | R16062_1_CTGF,14476 | R.GECQAEGVLFFQGDR.E | 1713.8229 | 1.2819 | 2 | 1 | 2.3276 | 0.4099 | 1683.1 | 1 | 19\|28 | 4.14 | 1 | 1 |  |
| $61-1 | | 4 | 2 | 4.37% | 70375 | 5.8 | sp\|P34931\|HS71L_HUMAN Heat shock 70 kDa protein 1-like OS=Homo sapiens GN=HSPA1L PE=1 SV=2 |  |  |  |  |  |  |  |  |
| $61-2 | | 4 | 2 | 4.37% | 70404.1 | 6 | tr\|Q53FA3\|Q53FA3_HUMAN Heat shock 70 kDa protein 1-like (Fragment) OS=Homo sapiens GN=HSPA1L PE=1 SV=1 |  |  |  |  |  |  |  |  |
| $61-3 | | 4 | 2 | 4.37% | 70375.9 | 5.6 | tr\|B2RCQ9\|B2RCQ9_HUMAN cDNA, FLJ96225, highly similar to Homo sapiens heat shock 70kDa protein 1-like (HSPA1L), mRNA OS=Homo sapiens PE=2 SV=1 |  |  |  |  |  |  |  |  |
| $61-4 | | 4 | 2 | 3.97% | 77561.3 | 8.2 | tr\|B4DI54\|B4DI54_HUMAN cDNA FLJ56386, highly similar to Heat shock 70 kDa protein 1L OS=Homo sapiens PE=2 SV=1 |  |  |  |  |  |  |  |  |
| $61-5 | | 4 | 2 | 5.26% | 58332.4 | 6.1 | tr\|B4DXY3\|B4DXY3_HUMAN cDNA FLJ56517, highly similar to Heat shock 70 kDa protein 1L OS=Homo sapiens PE=2 SV=1 |  |  |  |  |  |  |  |  |
|  | R16062_1_CTGF,14918 | K.DAGVIAGLNVLR.I | 1198.3975 | 0.1645 | 2 | 1 | 3.0132 | 0.3954 | 1723.8 | 1 | 19\|22 | 5.84 | 2 | 11 |  |
|  | R16062_1_CTGF,14933 | K.DAGVIAGLNVLR.I | 1198.3975 | 0.1015 | 2 | 1 | 2.5173 | 0.3155 | 1146.4 | 1 | 16\|22 | 5.84 | 2 | 11 |  |
|  | R16062_1_CTGF,14434 - 14435 | R.IINEPTAAAIAYGLDK.R | 1660.8927 | 0.2447 | 2 | 1 | 3.7359 | 0.5046 | 850.3 | 1 | 19\|30 | 4.37 | 2 | 7 |  |
|  | R16062_1_CTGF,14445 | R.IINEPTAAAIAYGLDK.R | 1660.8927 | 0.5087 | 2 | 1 | 3.3099 | 0.4476 | 1154.2 | 1 | 19\|30 | 4.37 | 2 | 7 |  |
| $62-1 | | 4 | 2 | 6.11% | 55928.1 | 8.5 | sp\|P02675\|FIBB_HUMAN Fibrinogen beta chain OS=Homo sapiens GN=FGB PE=1 SV=2 |  |  |  |  |  |  |  |  |
| $62-2 | | 4 | 2 | 8.72% | 39736.4 | 7 | tr\|D3DP13\|D3DP13_HUMAN Fibrinogen beta chain, isoform CRA_e OS=Homo sapiens GN=FGB PE=4 SV=1 |  |  |  |  |  |  |  |  |
| $62-3 | | 4 | 2 | 6.11% | 55928.1 | 8.5 | tr\|V9HVY1\|V9HVY1_HUMAN Epididymis secretory sperm binding protein Li 78p OS=Homo sapiens GN=HEL-S-78p PE=2 SV=1 |  |  |  |  |  |  |  |  |
| $62-4 | | 4 | 2 | 6.33% | 54253.1 | 8.3 | tr\|B4E1D3\|B4E1D3_HUMAN cDNA FLJ53952, highly similar to Fibrinogen beta chain OS=Homo sapiens PE=2 SV=1 |  |  |  |  |  |  |  |  |
|  | R16062_1_CTGF,12455 | K.DNENVVNEYSSELEK.H | 1769.8018 | 0.5468 | 2 | 1 | 2.6625 | 0.4708 | 507.1 | 1 | 14\|28 | 3.91 | 1 | 4 |  |
|  | R16062_1_CTGF,10298 | R.TPCTVSCNIPVVSGK.E | 1619.829 | 0.513 | 2 | 1 | 2.4922 | 0.4812 | 850.9 | 1 | 16\|28 | 7.74 | 1 | 4 |  |
|  | R16062_1_CTGF,10311 | R.TPCTVSCNIPVVSGK.E | 1619.829 | -0.556 | 2 | 1 | 2.7919 | 0.4314 | 649.7 | 1 | 15\|28 | 7.74 | 1 | 4 |  |
|  | R16062_1_CTGF,10366 | R.TPCTVSCNIPVVSGK.E | 1619.829 | 0.725 | 2 | 1 | 2.7624 | 0.4493 | 1010 | 1 | 17\|28 | 7.74 | 1 | 4 |  |
| $63-1 | | 4 | 2 | 4.79% | 61161.5 | 6 | sp\|Q9NZN4\|EHD2_HUMAN EH domain-containing protein 2 OS=Homo sapiens GN=EHD2 PE=1 SV=2 |  |  |  |  |  |  |  |  |
| $63-2 | | 4 | 2 | 4.88% | 60040.2 | 6.1 | tr\|B4DLA1\|B4DLA1_HUMAN cDNA FLJ53453, highly similar to EH-domain-containing protein 2 OS=Homo sapiens PE=2 SV=1 |  |  |  |  |  |  |  |  |
| $63-3 | | 4 | 2 | 6.30% | 46904.6 | 6.7 | tr\|Q8NCJ3\|Q8NCJ3_HUMAN cDNA FLJ90220 fis, clone MAMMA1003126, moderately similar to Human Hpast (HPAST) mRNA OS=Homo sapiens PE=2 SV=1 |  |  |  |  |  |  |  |  |
| $63-4 | | 4 | 2 | 4.79% | 61161.5 | 6 | tr\|A0A024R0S6\|A0A024R0S6_HUMAN EH-domain containing 2, isoform CRA_a OS=Homo sapiens GN=EHD2 PE=4 SV=1 |  |  |  |  |  |  |  |  |
|  | R16062_1_CTGF,18313 | K.LEISDEFSEAIGALR.G | 1650.8116 | -0.9564 | 2 | 1 | 2.4819 | 0.2204 | 314.4 | 1 | 11\|28 | 4 | 1 | 4 |  |
|  | R16062_1_CTGF,17054 | R.LFELEEQDLFR.D | 1439.5945 | 0.1825 | 2 | 1 | 2.4066 | 0.3406 | 1181 | 1 | 15\|20 | 4 | 1 | 4 |  |
|  | R16062_1_CTGF,17075 | R.LFELEEQDLFR.D | 1439.5945 | -0.6005 | 2 | 1 | 3.2622 | 0.4261 | 1246 | 1 | 15\|20 | 4 | 1 | 4 |  |
|  | R16062_1_CTGF,17086 | R.LFELEEQDLFR.D | 1439.5945 | 0.6065 | 2 | 1 | 3.1456 | 0.3729 | 1446 | 1 | 18\|20 | 4 | 1 | 4 |  |
| $64-1 | | 4 | 2 | 4.12% | 73680.4 | 5.9 | sp\|P38646\|GRP75_HUMAN Stress-70 protein, mitochondrial OS=Homo sapiens GN=HSPA9 PE=1 SV=2 |  |  |  |  |  |  |  |  |
| $64-2 | | 4 | 2 | 4.11% | 73853.6 | 6 | tr\|Q8N1C8\|Q8N1C8_HUMAN HSPA9 protein (Fragment) OS=Homo sapiens GN=HSPA9 PE=2 SV=1 |  |  |  |  |  |  |  |  |
| $64-3 | | 4 | 2 | 6.41% | 47362 | 6.2 | tr\|B7Z1V7\|B7Z1V7_HUMAN cDNA FLJ51811, highly similar to Stress-70 protein, mitochondrial OS=Homo sapiens PE=2 SV=1 |  |  |  |  |  |  |  |  |
| $64-4 | | 4 | 2 | 4.12% | 73779.6 | 6 | tr\|V9HW84\|V9HW84_HUMAN Epididymis secretory sperm binding protein Li 124m OS=Homo sapiens GN=HEL-S-124m PE=2 SV=1 |  |  |  |  |  |  |  |  |
| $64-5 | | 4 | 2 | 4.21% | 72400.9 | 5.7 | tr\|B7Z4V2\|B7Z4V2_HUMAN cDNA FLJ51907, highly similar to Stress-70 protein, mitochondrial OS=Homo sapiens PE=2 SV=1 |  |  |  |  |  |  |  |  |
|  | R16062_1_CTGF,17101 | R.AQFEGIVTDLIR.R | 1362.5564 | -1.1346 | 2 | 1 | 2.2714 | 0.2533 | 427.1 | 1 | 13\|22 | 4.37 | 1 | 5 |  |
|  | R16062_1_CTGF,17132 | R.AQFEGIVTDLIR.R | 1362.5564 | -0.8496 | 2 | 1 | 3.1634 | 0.2991 | 1054.9 | 1 | 16\|22 | 4.37 | 1 | 5 |  |
|  | R16062_1_CTGF,14199 | R.VINEPTAAALAYGLDK.S | 1646.866 | 0.248 | 2 | 1 | 2.6241 | 0.4915 | 445.1 | 1 | 17\|30 | 4.37 | 1 | 5 |  |
|  | R16062_1_CTGF,14212 | R.VINEPTAAALAYGLDK.S | 1646.866 | 0.929 | 2 | 1 | 2.4317 | 0.225 | 421.7 | 1 | 14\|30 | 4.37 | 1 | 5 |  |
| $65-1 | | 3 | 2 | 19.51% | 13519.3 | 6.8 | tr\|H7BZJ3\|H7BZJ3_HUMAN Protein disulfide-isomerase A3 (Fragment) OS=Homo sapiens GN=PDIA3 PE=1 SV=1 |  |  |  |  |  |  |  |  |
|  | R16062_1_CTGF,13594 | -.SDVLELTDDNFESR.I | 1640.6873 | 0.2533 | 2 | 1 | 3.1022 | 0.3034 | 1208 | 1 | 17\|26 | 3.77 | 1 | 1 |  |
|  | R16062_1_CTGF,13599 | -.SDVLELTDDNFESR.I | 1640.6873 | 0.5143 | 2 | 1 | 3.3438 | 0.3048 | 1616.2 | 1 | 18\|26 | 3.77 | 1 | 1 |  |
|  | R16062_1_CTGF,10494 | K.YGVSGYPTLK.I | 1085.2345 | -0.3535 | 2 | 1 | 2.4437 | 0.3897 | 631 | 1 | 13\|18 | 8.5 | 2 | 4 |  |
| $66-1 | | 3 | 2 | 4.28% | 68569.5 | 6 | sp\|P04843\|RPN1_HUMAN Dolichyl-diphosphooligosaccharide--protein glycosyltransferase subunit 1 OS=Homo sapiens GN=RPN1 PE=1 SV=1 |  |  |  |  |  |  |  |  |
| $66-2 | | 3 | 2 | 5.98% | 49921.2 | 6.1 | tr\|B7Z4L4\|B7Z4L4_HUMAN Dolichyl-diphosphooligosaccharide--protein glycosyltransferase subunit 1 OS=Homo sapiens GN=RPN1 PE=1 SV=1 |  |  |  |  |  |  |  |  |
| $66-3 | | 3 | 2 | 4.48% | 65838.4 | 5.8 | tr\|B4DL99\|B4DL99_HUMAN Dolichyl-diphosphooligosaccharide--protein glycosyltransferase subunit 1 OS=Homo sapiens PE=2 SV=1 |  |  |  |  |  |  |  |  |
| $66-4 | | 3 | 2 | 6.88% | 43368.9 | 6 | tr\|B4DNJ5\|B4DNJ5_HUMAN Dolichyl-diphosphooligosaccharide--protein glycosyltransferase subunit 1 OS=Homo sapiens PE=2 SV=1 |  |  |  |  |  |  |  |  |
| $66-5 | | 3 | 2 | 4.58% | 64581.9 | 6.1 | tr\|Q96HX3\|Q96HX3_HUMAN Dolichyl-diphosphooligosaccharide--protein glycosyltransferase subunit 1 (Fragment) OS=Homo sapiens PE=2 SV=1 |  |  |  |  |  |  |  |  |
| $66-6 | | 3 | 2 | 4.28% | 68579.6 | 6 | tr\|Q53EP4\|Q53EP4_HUMAN Dolichyl-diphosphooligosaccharide--protein glycosyltransferase subunit 1 (Fragment) OS=Homo sapiens PE=2 SV=1 |  |  |  |  |  |  |  |  |
| $66-7 | | 3 | 2 | 4.28% | 68606.6 | 6 | tr\|Q6IBR0\|Q6IBR0_HUMAN Dolichyl-diphosphooligosaccharide--protein glycosyltransferase subunit 1 OS=Homo sapiens GN=RPN1 PE=2 SV=1 |  |  |  |  |  |  |  |  |
|  | R16062_1_CTGF,14231 | K.ALTSEIALLQSR.L | 1302.5025 | 0.8235 | 2 | 1 | 2.8158 | 0.2696 | 1504.2 | 1 | 16\|22 | 6.05 | 1 | 7 |  |
|  | R16062_1_CTGF,14236 | K.ALTSEIALLQSR.L | 1302.5025 | 0.4455 | 2 | 1 | 3.2114 | 0.3291 | 1929.6 | 1 | 18\|22 | 6.05 | 1 | 7 |  |
|  | R16062_1_CTGF,16768 | K.VACITEQVLTLVNK.R | 1588.8646 | 0.2936 | 2 | 1 | 2.3605 | 0.4007 | 952.2 | 1 | 16\|26 | 5.97 | 1 | 7 |  |
| $67-1 | | 3 | 2 | 7.04% | 53139.9 | 6.8 | sp\|P52209\|6PGD_HUMAN 6-phosphogluconate dehydrogenase, decarboxylating OS=Homo sapiens GN=PGD PE=1 SV=3 |  |  |  |  |  |  |  |  |
|  | R16062_1_CTGF,17899 | K.LVPLLDTGDIIIDGGNSEYR.D | 2161.3978 | 1.8558 | 2 | 1 | 2.87 | 0.4733 | 435.1 | 1 | 17\|38 | 3.84 | 1 | 1 |  |
|  | R16062_1_CTGF,20388 | R.NPELQNLLLDDFFK.S | 1706.9199 | 0.3059 | 2 | 1 | 2.2869 | 0.29 | 1049.7 | 1 | 15\|26 | 4.03 | 1 | 1 |  |
|  | R16062_1_CTGF,20396 | R.NPELQNLLLDDFFK.S | 1706.9199 | 0.3799 | 2 | 1 | 3.9108 | 0.1418 | 1333.9 | 1 | 19\|26 | 4.03 | 1 | 1 |  |
| $68-1 | | 3 | 2 | 12.65% | 27745.2 | 4.7 | sp\|P63104\|1433Z_HUMAN 14-3-3 protein zeta/delta OS=Homo sapiens GN=YWHAZ PE=1 SV=1 |  |  |  |  |  |  |  |  |
| $68-2 | | 3 | 2 | 18.45% | 19072.4 | 4.5 | tr\|B0AZS6\|B0AZS6_HUMAN 14-3-3 protein zeta/delta OS=Homo sapiens GN=YWHAZ PE=1 SV=1 |  |  |  |  |  |  |  |  |
| $68-3 | | 3 | 2 | 12.65% | 27745.2 | 4.7 | tr\|D0PNI1\|D0PNI1_HUMAN Epididymis luminal protein 4 OS=Homo sapiens GN=YWHAZ PE=2 SV=1 |  |  |  |  |  |  |  |  |
| $68-4 | | 3 | 2 | 12.60% | 28036.7 | 4.9 | tr\|E7EX29\|E7EX29_HUMAN 14-3-3 protein zeta/delta (Fragment) OS=Homo sapiens GN=YWHAZ PE=1 SV=1 |  |  |  |  |  |  |  |  |
|  | R16062_1_CTGF,20090 | K.TAFDEAIAELDTLSEESYK.D | 2133.2517 | -0.2273 | 2 | 1 | 2.9519 | 0.5964 | 417.5 | 1 | 17\|36 | 3.77 | 1 | 4 |  |
|  | R16062_1_CTGF,19613 | R.DICNDVLSLLEK.F | 1419.5964 | 0.3394 | 2 | 1 | 2.3672 | 0.2669 | 733.6 | 1 | 15\|22 | 4.03 | 1 | 4 |  |
|  | R16062_1_CTGF,19633 | R.DICNDVLSLLEK.F | 1419.5964 | 0.5434 | 2 | 1 | 2.9644 | 0.1981 | 1459.8 | 1 | 17\|22 | 4.03 | 1 | 4 |  |
| $69-1 | | 3 | 2 | 3.84% | 85697.6 | 5.9 | sp\|P06396\|GELS_HUMAN Gelsolin OS=Homo sapiens GN=GSN PE=1 SV=1 |  |  |  |  |  |  |  |  |
| $69-2 | | 3 | 2 | 6.19% | 52342.8 | 5.3 | tr\|B3KS49\|B3KS49_HUMAN cDNA FLJ35478 fis, clone SMINT2007796, highly similar to Gelsolin OS=Homo sapiens PE=2 SV=1 |  |  |  |  |  |  |  |  |
| $69-3 | | 3 | 2 | 4.01% | 82525.6 | 5.4 | tr\|A0A0A0MS51\|A0A0A0MS51_HUMAN Gelsolin OS=Homo sapiens GN=GSN PE=1 SV=1 |  |  |  |  |  |  |  |  |
| $69-4 | | 3 | 2 | 3.91% | 84745.3 | 5.6 | tr\|A0A0A0MT01\|A0A0A0MT01_HUMAN Gelsolin OS=Homo sapiens GN=GSN PE=1 SV=1 |  |  |  |  |  |  |  |  |
| $69-5 | | 3 | 2 | 14.49% | 22948.8 | 4.6 | tr\|Q5T0H8\|Q5T0H8_HUMAN Gelsolin OS=Homo sapiens GN=GSN PE=1 SV=1 |  |  |  |  |  |  |  |  |
| $69-6 | | 3 | 2 | 3.91% | 84766.4 | 5.6 | tr\|B7Z6N2\|B7Z6N2_HUMAN cDNA FLJ56154, highly similar to Gelsolin OS=Homo sapiens PE=2 SV=1 |  |  |  |  |  |  |  |  |
| $69-7 | | 3 | 2 | 4.35% | 75752 | 5.6 | tr\|B7Z4U6\|B7Z4U6_HUMAN cDNA FLJ55803, highly similar to Gelsolin OS=Homo sapiens PE=2 SV=1 |  |  |  |  |  |  |  |  |
| $69-8 | | 3 | 2 | 3.97% | 83103.5 | 5.6 | tr\|B7Z9A0\|B7Z9A0_HUMAN cDNA FLJ56212, highly similar to Gelsolin OS=Homo sapiens PE=2 SV=1 |  |  |  |  |  |  |  |  |
| $69-9 | | 3 | 2 | 4.20% | 78831.6 | 5.8 | tr\|B7Z992\|B7Z992_HUMAN cDNA FLJ53698, highly similar to Gelsolin OS=Homo sapiens PE=2 SV=1 |  |  |  |  |  |  |  |  |
| $69-10 | | 3 | 2 | 6.19% | 52372.8 | 5.2 | tr\|Q5T0H9\|Q5T0H9_HUMAN Gelsolin OS=Homo sapiens GN=GSN PE=1 SV=1 |  |  |  |  |  |  |  |  |
| $69-11 | | 3 | 2 | 4.26% | 77789.4 | 5.5 | tr\|B7Z2X4\|B7Z2X4_HUMAN cDNA FLJ53327, highly similar to Gelsolin OS=Homo sapiens PE=2 SV=1 |  |  |  |  |  |  |  |  |
|  | R16062_1_CTGF,8474 | K.TGAQELLR.V | 888.004 | 0.213 | 2 | 1 | 2.6628 | 0.226 | 507.9 | 1 | 13\|14 | 5.66 | 1 | 11 |  |
|  | R16062_1_CTGF,16659 | R.AQPVQVAEGSEPDGFWEALGGK.A | 2273.4436 | -0.8154 | 2 | 1 | 2.6138 | 0.2672 | 316.5 | 1 | 13\|42 | 4 | 1 | 11 |  |
|  | R16062_1_CTGF,16665 | R.AQPVQVAEGSEPDGFWEALGGK.A | 2273.4436 | 1.1206 | 2 | 1 | 3.6466 | 0.515 | 761.6 | 1 | 19\|42 | 4 | 1 | 11 |  |
| $70-1 | | 3 | 2 | 21.54% | 14135.5 | 11 | sp\|P04908\|H2A1B_HUMAN Histone H2A type 1-B/E OS=Homo sapiens GN=HIST1H2AB PE=1 SV=2 |  |  |  |  |  |  |  |  |
| $70-2 | | 3 | 2 | 21.54% | 14105.4 | 11 | sp\|Q93077\|H2A1C_HUMAN Histone H2A type 1-C OS=Homo sapiens GN=HIST1H2AC PE=1 SV=3 |  |  |  |  |  |  |  |  |
| $70-3 | | 3 | 2 | 21.54% | 14107.4 | 11 | sp\|P20671\|H2A1D_HUMAN Histone H2A type 1-D OS=Homo sapiens GN=HIST1H2AD PE=1 SV=2 |  |  |  |  |  |  |  |  |
| $70-4 | | 3 | 2 | 21.88% | 13906.2 | 11 | sp\|Q96KK5\|H2A1H_HUMAN Histone H2A type 1-H OS=Homo sapiens GN=HIST1H2AH PE=1 SV=3 |  |  |  |  |  |  |  |  |
| $70-5 | | 3 | 2 | 21.88% | 13936.2 | 11 | sp\|Q99878\|H2A1J_HUMAN Histone H2A type 1-J OS=Homo sapiens GN=HIST1H2AJ PE=1 SV=3 |  |  |  |  |  |  |  |  |
| $70-6 | | 3 | 2 | 21.54% | 14091.4 | 11 | sp\|P0C0S8\|H2A1_HUMAN Histone H2A type 1 OS=Homo sapiens GN=HIST1H2AG PE=1 SV=2 |  |  |  |  |  |  |  |  |
| $70-7 | | 3 | 2 | 21.54% | 14095.5 | 11 | sp\|Q6FI13\|H2A2A_HUMAN Histone H2A type 2-A OS=Homo sapiens GN=HIST2H2AA3 PE=1 SV=3 |  |  |  |  |  |  |  |  |
| $70-8 | | 3 | 2 | 21.71% | 13988.3 | 11 | sp\|Q16777\|H2A2C_HUMAN Histone H2A type 2-C OS=Homo sapiens GN=HIST2H2AC PE=1 SV=4 |  |  |  |  |  |  |  |  |
| $70-9 | | 3 | 2 | 21.54% | 14121.4 | 11 | sp\|Q7L7L0\|H2A3_HUMAN Histone H2A type 3 OS=Homo sapiens GN=HIST3H2A PE=1 SV=3 |  |  |  |  |  |  |  |  |
| $70-10 | | 3 | 2 | 21.71% | 14019.4 | 11 | sp\|Q9BTM1\|H2AJ_HUMAN Histone H2A.J OS=Homo sapiens GN=H2AFJ PE=1 SV=1 |  |  |  |  |  |  |  |  |
| $70-11 | | 3 | 2 | 21.54% | 14135.5 | 11 | tr\|Q08AJ9\|Q08AJ9_HUMAN Histone H2A OS=Homo sapiens GN=HIST1H2AB PE=2 SV=1 |  |  |  |  |  |  |  |  |
| $70-12 | | 3 | 2 | 21.88% | 13906.2 | 11 | tr\|A3KPC7\|A3KPC7_HUMAN Histone H2A OS=Homo sapiens GN=HIST1H2AH PE=2 SV=1 |  |  |  |  |  |  |  |  |
| $70-13 | | 3 | 2 | 21.54% | 14091.4 | 11 | tr\|A4FTV9\|A4FTV9_HUMAN Histone H2A OS=Homo sapiens GN=HIST1H2AK PE=2 SV=1 |  |  |  |  |  |  |  |  |
| $70-14 | | 3 | 2 | 21.54% | 14110.5 | 11 | tr\|B2R5B3\|B2R5B3_HUMAN Histone H2A OS=Homo sapiens PE=2 SV=1 |  |  |  |  |  |  |  |  |
| $70-15 | | 3 | 2 | 21.54% | 14105.4 | 11 | tr\|A0A024R017\|A0A024R017_HUMAN Histone H2A OS=Homo sapiens GN=HIST1H2AC PE=3 SV=1 |  |  |  |  |  |  |  |  |
| $70-16 | | 3 | 2 | 21.71% | 14019.4 | 11 | tr\|A0A024RAS2\|A0A024RAS2_HUMAN Histone H2A OS=Homo sapiens GN=H2AFJ PE=3 SV=1 |  |  |  |  |  |  |  |  |
|  | R16062_1_CTGF,11759 | R.AGLQFPVGR.I | 945.1004 | 1.1474 | 2 | 1 | 2.2401 | 0.206 | 507.5 | 1 | 11\|16 | 9.8 | 2 | 19 |  |
|  | R16062_1_CTGF,18472 | R.VTIAQGGVLPNIQAVLLPK.K | 1932.3395 | 1.3195 | 2 | 1 | 2.9111 | 0.4205 | 503.8 | 1 | 17\|36 | 8.72 | 1 | 16 |  |
|  | R16062_1_CTGF,18486 | R.VTIAQGGVLPNIQAVLLPK.K | 1932.3395 | 0.8935 | 3 | 1 | 4.0088 | 0.4763 | 2122.7 | 1 | 34\|72 | 8.72 | 1 | 16 |  |
| $71-1 | | 3 | 2 | 2.42% | 129314 | 9.1 | sp\|P08123\|CO1A2_HUMAN Collagen alpha-2(I) chain OS=Homo sapiens GN=COL1A2 PE=1 SV=7 |  |  |  |  |  |  |  |  |
| $71-2 | | 3 | 2 | 2.42% | 129151 | 9.1 | tr\|A0A087WTA8\|A0A087WTA8_HUMAN Collagen alpha-2(I) chain OS=Homo sapiens GN=COL1A2 PE=1 SV=1 |  |  |  |  |  |  |  |  |
|  | R16062_1_CTGF,5397 | R.GEAGAAGPAGPAGPR.G | 1236.3195 | 0.6285 | 2 | 1 | 2.7141 | 0.3694 | 691.6 | 1 | 18\|28 | 6 | 1 | 2 |  |
|  | R16062_1_CTGF,5405 | R.GEAGAAGPAGPAGPR.G | 1236.3195 | 0.5255 | 2 | 1 | 2.5646 | 0.3873 | 991.8 | 1 | 17\|28 | 6 | 1 | 2 |  |
|  | R16062_1_CTGF,8348 | R.GETGPSGPVGPAGAVGPR.G | 1563.6972 | 1.1302 | 2 | 1 | 2.8877 | 0.4856 | 864.1 | 1 | 19\|34 | 6 | 1 | 2 |  |
| $72-1 | | 3 | 2 | 3.76% | 103358 | 6.5 | sp\|Q14624\|ITIH4_HUMAN Inter-alpha-trypsin inhibitor heavy chain H4 OS=Homo sapiens GN=ITIH4 PE=1 SV=4 |  |  |  |  |  |  |  |  |
| $72-2 | | 3 | 2 | 3.74% | 103881 | 6.4 | tr\|B7ZKJ8\|B7ZKJ8_HUMAN ITIH4 protein OS=Homo sapiens GN=ITIH4 PE=1 SV=1 |  |  |  |  |  |  |  |  |
| $72-3 | | 3 | 2 | 3.94% | 98347.6 | 6.2 | tr\|B7Z544\|B7Z544_HUMAN cDNA FLJ51742, highly similar to Inter-alpha-trypsin inhibitor heavy chain H4 OS=Homo sapiens PE=2 SV=1 |  |  |  |  |  |  |  |  |
| $72-4 | | 3 | 2 | 5.49% | 70852.5 | 5.9 | tr\|Q68DH2\|Q68DH2_HUMAN Putative uncharacterized protein DKFZp686G21125 (Fragment) OS=Homo sapiens GN=DKFZp686G21125 PE=2 SV=1 |  |  |  |  |  |  |  |  |
| $72-5 | | 3 | 2 | 5.40% | 71906.7 | 6 | tr\|B7Z551\|B7Z551_HUMAN cDNA FLJ60767, highly similar to Inter-alpha-trypsin inhibitor heavy chain H4 OS=Homo sapiens PE=2 SV=1 |  |  |  |  |  |  |  |  |
| $72-6 | | 3 | 2 | 6.25% | 62161.4 | 5.4 | tr\|B7Z8Q7\|B7Z8Q7_HUMAN cDNA FLJ53871, highly similar to Inter-alpha-trypsin inhibitor heavy chain H4 OS=Homo sapiens PE=2 SV=1 |  |  |  |  |  |  |  |  |
| $72-7 | | 3 | 2 | 3.76% | 103362 | 6.5 | tr\|B2RMS9\|B2RMS9_HUMAN Inter-alpha (Globulin) inhibitor H4 (Plasma Kallikrein-sensitive glycoprotein) OS=Homo sapiens GN=ITIH4 PE=2 SV=1 |  |  |  |  |  |  |  |  |
| $72-8 | | 3 | 2 | 4.87% | 79953.5 | 5.3 | tr\|H7C0L5\|H7C0L5_HUMAN Inter-alpha-trypsin inhibitor heavy chain H4 (Fragment) OS=Homo sapiens GN=ITIH4 PE=1 SV=1 |  |  |  |  |  |  |  |  |
| $72-9 | | 3 | 2 | 5.01% | 76962.3 | 5.7 | tr\|Q59FS1\|Q59FS1_HUMAN Inter-alpha (Globulin) inhibitor H4 (Plasma Kallikrein-sensitive glycoprotein) variant (Fragment) OS=Homo sapiens PE=4 SV=1 |  |  |  |  |  |  |  |  |
|  | R16062_1_CTGF,16797 | R.LGVYELLLK.V | 1048.3004 | 0.3084 | 2 | 1 | 2.713 | 0.4444 | 818 | 1 | 14\|16 | 6 | 1 | 9 |  |
|  | R16062_1_CTGF,21874 | R.LWAYLTIQQLLEQTVSASDADQQALR.N | 2963.2912 | 0.1832 | 3 | 1 | 3.8445 | 0.46 | 1055.5 | 1 | 31\|100 | 4.03 | 1 | 9 |  |
|  | R16062_1_CTGF,21993 | R.LWAYLTIQQLLEQTVSASDADQQALR.N | 2963.2912 | 1.0292 | 3 | 1 | 3.7967 | 0.4293 | 513.6 | 1 | 26\|100 | 4.03 | 1 | 9 |  |
| $73-1 | | 3 | 2 | 4.60% | 69284 | 5.4 | sp\|P04844\|RPN2_HUMAN Dolichyl-diphosphooligosaccharide--protein glycosyltransferase subunit 2 OS=Homo sapiens GN=RPN2 PE=1 SV=3 |  |  |  |  |  |  |  |  |
| $73-2 | | 3 | 2 | 4.60% | 69334.2 | 5.5 | tr\|B2RE46\|B2RE46_HUMAN cDNA, FLJ96923, highly similar to Homo sapiens ribophorin II (RPN2), mRNA OS=Homo sapiens PE=2 SV=1 |  |  |  |  |  |  |  |  |
| $73-3 | | 3 | 2 | 17.47% | 18170.6 | 5.3 | tr\|Q5JYR4\|Q5JYR4_HUMAN Dolichyl-diphosphooligosaccharide--protein glycosyltransferase subunit 2 (Fragment) OS=Homo sapiens GN=RPN2 PE=1 SV=6 |  |  |  |  |  |  |  |  |
| $73-4 | | 3 | 2 | 8.43% | 37109.2 | 5.4 | tr\|Q5JYR7\|Q5JYR7_HUMAN Dolichyl-diphosphooligosaccharide--protein glycosyltransferase subunit 2 (Fragment) OS=Homo sapiens GN=RPN2 PE=1 SV=6 |  |  |  |  |  |  |  |  |
|  | R16062_1_CTGF,14309 | R.LQVTNVLSQPLTQATVK.L | 1841.1421 | 0.5411 | 2 | 1 | 2.6739 | 0.423 | 475.1 | 1 | 13\|32 | 8.75 | 1 | 4 |  |
|  | R16062_1_CTGF,14329 - 14330 | R.LQVTNVLSQPLTQATVK.L | 1841.1421 | 0.4401 | 2 | 1 | 3.0755 | 0.371 | 813 | 1 | 16\|32 | 8.75 | 1 | 4 |  |
|  | R16062_1_CTGF,20411 | R.SIVEEIEDLVAR.L | 1373.5343 | -1.5727 | 2 | 1 | 2.2164 | 0.172 | 1054.8 | 1 | 14\|22 | 4 | 1 | 4 |  |
| $74-1 | | 3 | 2 | 3.65% | 70811 | 5.4 | sp\|P13797\|PLST_HUMAN Plastin-3 OS=Homo sapiens GN=PLS3 PE=1 SV=4 |  |  |  |  |  |  |  |  |
| $74-2 | | 3 | 2 | 5.62% | 46003.8 | 6.2 | tr\|Q96HI1\|Q96HI1_HUMAN Similar to plastin 3 (T isoform) (Fragment) OS=Homo sapiens PE=2 SV=1 |  |  |  |  |  |  |  |  |
| $74-3 | | 3 | 2 | 3.65% | 70766.9 | 5.5 | tr\|Q53GY0\|Q53GY0_HUMAN Plastin 3 variant (Fragment) OS=Homo sapiens PE=2 SV=1 |  |  |  |  |  |  |  |  |
| $74-4 | | 3 | 2 | 3.81% | 67599.3 | 5.5 | tr\|B4DPW9\|B4DPW9_HUMAN cDNA FLJ51929, highly similar to Plastin-3 OS=Homo sapiens PE=2 SV=1 |  |  |  |  |  |  |  |  |
| $74-5 | | 3 | 2 | 3.73% | 69334.4 | 5.7 | tr\|A0A0A0MSQ0\|A0A0A0MSQ0_HUMAN Plastin-3 OS=Homo sapiens GN=PLS3 PE=1 SV=1 |  |  |  |  |  |  |  |  |
|  | R16062_1_CTGF,14233 | R.NEALAALLR.D | 971.1361 | 0.2001 | 2 | 1 | 2.5307 | 0.2839 | 1173.2 | 2 | 14\|16 | 6 | 1 | 5 |  |
|  | R16062_1_CTGF,14256 | R.NEALAALLR.D | 971.1361 | 0.5631 | 2 | 1 | 2.3975 | 0.3143 | 1034.7 | 1 | 14\|16 | 6 | 1 | 5 |  |
|  | R16062_1_CTGF,15979 | R.YTLNVLEDLGDGQK.A | 1565.7067 | 0.2857 | 2 | 1 | 2.8487 | 0.4398 | 484.6 | 1 | 16\|26 | 4.03 | 1 | 5 |  |
| $75-1 | | 2 | 2 | 7.89% | 34364.5 | 5.4 | sp\|Q8NHW5\|RLA0L_HUMAN 60S acidic ribosomal protein P0-like OS=Homo sapiens GN=RPLP0P6 PE=5 SV=1 |  |  |  |  |  |  |  |  |
| $75-2 | | 2 | 2 | 7.89% | 34273.5 | 5.7 | sp\|P05388\|RLA0_HUMAN 60S acidic ribosomal protein P0 OS=Homo sapiens GN=RPLP0 PE=1 SV=1 |  |  |  |  |  |  |  |  |
| $75-3 | | 2 | 2 | 7.89% | 34273.5 | 5.7 | tr\|A0A024RBS2\|A0A024RBS2_HUMAN 60S acidic ribosomal protein P0 OS=Homo sapiens GN=RPLP0 PE=3 SV=1 |  |  |  |  |  |  |  |  |
| $75-4 | | 2 | 2 | 7.89% | 34301.5 | 5.7 | tr\|Q53HW2\|Q53HW2_HUMAN 60S acidic ribosomal protein P0 (Fragment) OS=Homo sapiens PE=2 SV=1 |  |  |  |  |  |  |  |  |
| $75-5 | | 2 | 2 | 7.89% | 34239.5 | 5.7 | tr\|A8K4Z4\|A8K4Z4_HUMAN 60S acidic ribosomal protein P0 OS=Homo sapiens PE=2 SV=1 |  |  |  |  |  |  |  |  |
| $75-6 | | 2 | 2 | 10.12% | 27187.6 | 9.1 | tr\|F8VU65\|F8VU65_HUMAN 60S acidic ribosomal protein P0 (Fragment) OS=Homo sapiens GN=RPLP0 PE=1 SV=1 |  |  |  |  |  |  |  |  |
| $75-7 | | 2 | 2 | 16.34% | 16680.5 | 9.4 | tr\|F8VPE8\|F8VPE8_HUMAN 60S acidic ribosomal protein P0 (Fragment) OS=Homo sapiens GN=RPLP0 PE=1 SV=1 |  |  |  |  |  |  |  |  |
| $75-8 | | 2 | 2 | 9.40% | 28538.6 | 4.7 | tr\|B4E3D5\|B4E3D5_HUMAN cDNA FLJ51469, highly similar to 60S acidic ribosomal protein P0 OS=Homo sapiens PE=2 SV=1 |  |  |  |  |  |  |  |  |
| $75-9 | | 2 | 2 | 10.25% | 26901.3 | 9.1 | tr\|F8VW21\|F8VW21_HUMAN 60S acidic ribosomal protein P0 (Fragment) OS=Homo sapiens GN=RPLP0 PE=1 SV=1 |  |  |  |  |  |  |  |  |
|  | R16062_1_CTGF,17476 | K.TSFFQALGITTK.I | 1314.5117 | -0.4773 | 2 | 1 | 2.6345 | 0.5026 | 806.6 | 1 | 14\|22 | 8.41 | 1 | 9 |  |
|  | R16062_1_CTGF,17090 | R.GTIEILSDVQLIK.T | 1429.6842 | 1.4752 | 2 | 1 | 2.3357 | 0.2197 | 274.6 | 6 | 11\|24 | 4.37 | 1 | 9 |  |
| $76-1 | | 2 | 2 | 14.84% | 13508.6 | 11 | sp\|Q71UI9\|H2AV_HUMAN Histone H2A.V OS=Homo sapiens GN=H2AFV PE=1 SV=3 |  |  |  |  |  |  |  |  |
| $76-2 | | 2 | 2 | 14.84% | 13552.7 | 11 | sp\|P0C0S5\|H2AZ_HUMAN Histone H2A.Z OS=Homo sapiens GN=H2AFZ PE=1 SV=2 |  |  |  |  |  |  |  |  |
| $76-3 | | 2 | 2 | 15.57% | 13163.1 | 10 | tr\|C9J0D1\|C9J0D1_HUMAN Histone H2A OS=Homo sapiens GN=H2AFV PE=3 SV=1 |  |  |  |  |  |  |  |  |
|  | R16062_1_CTGF,11759 | R.AGLQFPVGR.I | 945.1004 | 1.1474 | 2 | 1 | 2.2401 | 0.206 | 507.5 | 1 | 11\|16 | 9.8 | 2 | 19 |  |
|  | R16062_1_CTGF,12598 | R.GDEELDSLIK.A | 1119.2044 | 0.1084 | 2 | 1 | 2.3349 | 0.3307 | 449 | 2 | 10\|18 | 3.92 | 1 | 3 |  |
| $77-1 | | 2 | 2 | 5.37% | 70754.4 | 7.7 | sp\|Q13492\|PICAL_HUMAN Phosphatidylinositol-binding clathrin assembly protein OS=Homo sapiens GN=PICALM PE=1 SV=2 |  |  |  |  |  |  |  |  |
| $77-2 | | 2 | 2 | 5.37% | 70754.4 | 7.7 | tr\|A0A024R5P1\|A0A024R5P1_HUMAN Phosphatidylinositol binding clathrin assembly protein, isoform CRA_i OS=Homo sapiens GN=PICALM PE=4 SV=1 |  |  |  |  |  |  |  |  |
| $77-3 | | 2 | 2 | 5.74% | 66393.6 | 8.8 | tr\|B5BU72\|B5BU72_HUMAN Phosphatidylinositol-binding clathrin assembly protein isoform 2 OS=Homo sapiens GN=PICALM PE=2 SV=1 |  |  |  |  |  |  |  |  |
| $77-4 | | 2 | 2 | 5.37% | 70755.3 | 7.2 | tr\|A8K5U9\|A8K5U9_HUMAN cDNA FLJ75056, highly similar to Homo sapiens phosphatidylinositol binding clathrin assembly protein (PICALM), mRNA OS=Homo sapiens PE=2 SV=1 |  |  |  |  |  |  |  |  |
| $77-5 | | 2 | 2 | 5.74% | 66392.6 | 8.9 | tr\|A0A024R5L7\|A0A024R5L7_HUMAN Phosphatidylinositol binding clathrin assembly protein, isoform CRA_c OS=Homo sapiens GN=PICALM PE=4 SV=1 |  |  |  |  |  |  |  |  |
|  | R16062_1_CTGF,18465 | R.ATTLSNAVSSLASTGLSLTK.V | 1923.1551 | 0.7011 | 2 | 1 | 2.8447 | 0.4984 | 747.7 | 1 | 17\|38 | 8.8 | 1 | 5 |  |
|  | R16062_1_CTGF,18864 | R.LFAAYNEGIINLLEK.Y | 1708.979 | -0.448 | 2 | 1 | 2.6587 | 0.4192 | 316.8 | 10 | 10\|28 | 4.53 | 1 | 5 |  |
| $78-1 | | 2 | 2 | 6.28% | 54305.9 | 5.6 | sp\|P04004\|VTNC_HUMAN Vitronectin OS=Homo sapiens GN=VTN PE=1 SV=1 |  |  |  |  |  |  |  |  |
| $78-2 | | 2 | 2 | 6.28% | 54305.9 | 5.6 | tr\|D9ZGG2\|D9ZGG2_HUMAN Vitronectin OS=Homo sapiens GN=VTN PE=4 SV=1 |  |  |  |  |  |  |  |  |
|  | R16062_1_CTGF,18275 | R.DVWGIEGPIDAAFTR.I | 1647.8126 | 1.3196 | 2 | 1 | 2.4087 | 0.321 | 824.7 | 1 | 19\|28 | 4.03 | 1 | 2 |  |
|  | R16062_1_CTGF,16694 | R.SIAQYWLGCPAPGHL.- | 1670.886 | 1.208 | 2 | 1 | 2.6032 | 0.4165 | 377.6 | 1 | 11\|28 | 6.45 | 1 | 2 |  |
| $79-1 | | 4 | 1 | 11.90% | 13950.2 | 10 | sp\|P33778\|H2B1B_HUMAN Histone H2B type 1-B OS=Homo sapiens GN=HIST1H2BB PE=1 SV=2 |  |  |  |  |  |  |  |  |
| $79-2 | | 4 | 1 | 11.90% | 13906.1 | 10 | sp\|P62807\|H2B1C_HUMAN Histone H2B type 1-C/E/F/G/I OS=Homo sapiens GN=HIST1H2BC PE=1 SV=4 |  |  |  |  |  |  |  |  |
| $79-3 | | 4 | 1 | 11.90% | 13936.1 | 10 | sp\|P58876\|H2B1D_HUMAN Histone H2B type 1-D OS=Homo sapiens GN=HIST1H2BD PE=1 SV=2 |  |  |  |  |  |  |  |  |
| $79-4 | | 4 | 1 | 11.90% | 13892.1 | 10 | sp\|Q93079\|H2B1H_HUMAN Histone H2B type 1-H OS=Homo sapiens GN=HIST1H2BH PE=1 SV=3 |  |  |  |  |  |  |  |  |
| $79-5 | | 4 | 1 | 11.90% | 13904.1 | 10 | sp\|P06899\|H2B1J_HUMAN Histone H2B type 1-J OS=Homo sapiens GN=HIST1H2BJ PE=1 SV=3 |  |  |  |  |  |  |  |  |
| $79-6 | | 4 | 1 | 11.90% | 13890.1 | 10 | sp\|O60814\|H2B1K_HUMAN Histone H2B type 1-K OS=Homo sapiens GN=HIST1H2BK PE=1 SV=3 |  |  |  |  |  |  |  |  |
| $79-7 | | 4 | 1 | 11.90% | 13952.2 | 10 | sp\|Q99880\|H2B1L_HUMAN Histone H2B type 1-L OS=Homo sapiens GN=HIST1H2BL PE=1 SV=3 |  |  |  |  |  |  |  |  |
| $79-8 | | 4 | 1 | 11.90% | 13989.2 | 10 | sp\|Q99879\|H2B1M_HUMAN Histone H2B type 1-M OS=Homo sapiens GN=HIST1H2BM PE=1 SV=3 |  |  |  |  |  |  |  |  |
| $79-9 | | 4 | 1 | 11.90% | 13922.1 | 10 | sp\|Q99877\|H2B1N_HUMAN Histone H2B type 1-N OS=Homo sapiens GN=HIST1H2BN PE=1 SV=3 |  |  |  |  |  |  |  |  |
| $79-10 | | 4 | 1 | 11.90% | 13906.1 | 10 | sp\|P23527\|H2B1O_HUMAN Histone H2B type 1-O OS=Homo sapiens GN=HIST1H2BO PE=1 SV=3 |  |  |  |  |  |  |  |  |
| $79-11 | | 4 | 1 | 11.90% | 13920.1 | 10 | sp\|Q16778\|H2B2E_HUMAN Histone H2B type 2-E OS=Homo sapiens GN=HIST2H2BE PE=1 SV=3 |  |  |  |  |  |  |  |  |
| $79-12 | | 4 | 1 | 11.90% | 13920.1 | 10 | sp\|Q5QNW6\|H2B2F_HUMAN Histone H2B type 2-F OS=Homo sapiens GN=HIST2H2BF PE=1 SV=3 |  |  |  |  |  |  |  |  |
| $79-13 | | 4 | 1 | 11.90% | 13908.1 | 10 | sp\|Q8N257\|H2B3B_HUMAN Histone H2B type 3-B OS=Homo sapiens GN=HIST3H2BB PE=1 SV=3 |  |  |  |  |  |  |  |  |
| $79-14 | | 4 | 1 | 11.90% | 13944.2 | 10 | sp\|P57053\|H2BFS_HUMAN Histone H2B type F-S OS=Homo sapiens GN=H2BFS PE=1 SV=2 |  |  |  |  |  |  |  |  |
| $79-15 | | 4 | 1 | 11.90% | 13906.1 | 10 | tr\|B2R4S9\|B2R4S9_HUMAN Histone H2B OS=Homo sapiens GN=HIST1H2BE PE=2 SV=1 |  |  |  |  |  |  |  |  |
| $79-16 | | 4 | 1 | 11.90% | 13922.1 | 10 | tr\|A0A024RCJ9\|A0A024RCJ9_HUMAN Histone H2B OS=Homo sapiens PE=3 SV=1 |  |  |  |  |  |  |  |  |
| $79-17 | | 4 | 1 | 9.04% | 18803.9 | 11 | tr\|U3KQK0\|U3KQK0_HUMAN Histone H2B OS=Homo sapiens GN=HIST1H2BN PE=1 SV=1 |  |  |  |  |  |  |  |  |
| $79-18 | | 4 | 1 | 11.90% | 13936.1 | 10 | tr\|A0A024QZZ7\|A0A024QZZ7_HUMAN Histone H2B OS=Homo sapiens GN=HIST1H2BD PE=3 SV=1 |  |  |  |  |  |  |  |  |
| $79-19 | | 4 | 1 | 11.90% | 13904.1 | 10 | tr\|A0A024RCJ2\|A0A024RCJ2_HUMAN Histone H2B OS=Homo sapiens GN=HIST1H2BJ PE=3 SV=1 |  |  |  |  |  |  |  |  |
| $79-20 | | 4 | 1 | 10.87% | 15156.5 | 10 | tr\|I6L9F7\|I6L9F7_HUMAN Histone H2B (Fragment) OS=Homo sapiens GN=HIST1H2BM PE=2 SV=1 |  |  |  |  |  |  |  |  |
| $79-21 | | 4 | 1 | 11.90% | 13996.2 | 10 | tr\|A8K9J7\|A8K9J7_HUMAN Histone H2B OS=Homo sapiens PE=2 SV=1 |  |  |  |  |  |  |  |  |
| $79-22 | | 4 | 1 | 11.90% | 13890.1 | 10 | tr\|A0A024RCL8\|A0A024RCL8_HUMAN Histone H2B OS=Homo sapiens GN=HIST1H2BK PE=3 SV=1 |  |  |  |  |  |  |  |  |
| $79-23 | | 4 | 1 | 9.04% | 18040.8 | 10 | tr\|B4DR52\|B4DR52_HUMAN Histone H2B OS=Homo sapiens PE=2 SV=1 |  |  |  |  |  |  |  |  |
| $79-24 | | 4 | 1 | 11.90% | 13834 | 10 | tr\|Q0D2M2\|Q0D2M2_HUMAN HIST1H2BC protein OS=Homo sapiens GN=HIST1H2BC PE=2 SV=1 |  |  |  |  |  |  |  |  |
|  | R16062_1_CTGF,16625 | K.AM*GIM*NSFVNDIFER.I | 1777.0155 | 1.0715 | 2 | 1 | 2.6873 | 0.4551 | 717.1 | 1 | 16\|28 | 4.37 | 1 | 24 |  |
|  | R16062_1_CTGF,16638 | K.AM*GIM*NSFVNDIFER.I | 1777.0155 | -1.9725 | 2 | 1 | 2.5188 | 0.2846 | 591.2 | 1 | 15\|28 | 4.37 | 1 | 24 |  |
|  | R16062_1_CTGF,16672 | K.AM*GIM*NSFVNDIFER.I | 1777.0155 | 2.3125 | 2 | 1 | 2.3831 | 0.3461 | 874.9 | 1 | 18\|28 | 4.37 | 1 | 24 |  |
|  | R16062_1_CTGF,19144 | K.AM*GIMNSFVNDIFER.I | 1761.0161 | 0.4461 | 2 | 1 | 2.3315 | 0.3414 | 392.8 | 1 | 11\|28 | 4.37 | 1 | 24 |  |
| $80-1 | | 4 | 1 | 12.05% | 18502.5 | 8.2 | sp\|P23528\|COF1_HUMAN Cofilin-1 OS=Homo sapiens GN=CFL1 PE=1 SV=3 |  |  |  |  |  |  |  |  |
| $80-2 | | 4 | 1 | 13.42% | 16811.6 | 8.5 | tr\|G3V1A4\|G3V1A4_HUMAN Cofilin 1 (Non-muscle), isoform CRA_a OS=Homo sapiens GN=CFL1 PE=1 SV=1 |  |  |  |  |  |  |  |  |
| $80-3 | | 4 | 1 | 12.05% | 18502.5 | 8.2 | tr\|V9HWI5\|V9HWI5_HUMAN Epididymis secretory protein Li 15 OS=Homo sapiens GN=HEL-S-15 PE=2 SV=1 |  |  |  |  |  |  |  |  |
| $80-4 | | 4 | 1 | 25.32% | 9090.51 | 8.5 | tr\|E9PLJ3\|E9PLJ3_HUMAN Cofilin-1 (Fragment) OS=Homo sapiens GN=CFL1 PE=1 SV=1 |  |  |  |  |  |  |  |  |
| $80-5 | | 4 | 1 | 12.58% | 17777.6 | 8.2 | tr\|E9PP50\|E9PP50_HUMAN Cofilin-1 (Fragment) OS=Homo sapiens GN=CFL1 PE=1 SV=6 |  |  |  |  |  |  |  |  |
| $80-6 | | 4 | 1 | 9.80% | 22728.1 | 8.5 | tr\|E9PK25\|E9PK25_HUMAN Cofilin-1 OS=Homo sapiens GN=CFL1 PE=1 SV=1 |  |  |  |  |  |  |  |  |
| $80-7 | | 4 | 1 | 17.86% | 12459.5 | 6.3 | tr\|B4E112\|B4E112_HUMAN cDNA FLJ51435, moderately similar to Cofilin-1 OS=Homo sapiens PE=2 SV=1 |  |  |  |  |  |  |  |  |
| $80-8 | | 4 | 1 | 22.22% | 10079.6 | 5.5 | tr\|E9PS23\|E9PS23_HUMAN Cofilin-1 (Fragment) OS=Homo sapiens GN=CFL1 PE=1 SV=6 |  |  |  |  |  |  |  |  |
| $80-9 | | 4 | 1 | 16.39% | 13923.2 | 8.9 | tr\|E9PQB7\|E9PQB7_HUMAN Cofilin-1 (Fragment) OS=Homo sapiens GN=CFL1 PE=1 SV=1 |  |  |  |  |  |  |  |  |
|  | R16062_1_CTGF,16101 | K.EILVGDVGQTVDDPYATFVK.M | 2167.4008 | 1.0828 | 2 | 1 | 2.6299 | 0.4699 | 458.5 | 1 | 14\|38 | 3.84 | 1 | 9 |  |
|  | R16062_1_CTGF,16133 | K.EILVGDVGQTVDDPYATFVK.M | 2167.4008 | -0.1002 | 2 | 1 | 3.8771 | 0.5783 | 1301.3 | 1 | 21\|38 | 3.84 | 1 | 9 |  |
|  | R16062_1_CTGF,16183 | K.EILVGDVGQTVDDPYATFVK.M | 2167.4008 | 0.9968 | 2 | 1 | 3.7735 | 0.551 | 1735.1 | 1 | 24\|38 | 3.84 | 1 | 9 |  |
|  | R16062_1_CTGF,16194 | K.EILVGDVGQTVDDPYATFVK.M | 2167.4008 | 1.6648 | 2 | 1 | 3.1281 | 0.5657 | 925.4 | 1 | 19\|38 | 3.84 | 1 | 9 |  |
| $81-1 | | 4 | 1 | 3.05% | 63147 | 8.4 | sp\|P06744\|G6PI_HUMAN Glucose-6-phosphate isomerase OS=Homo sapiens GN=GPI PE=1 SV=4 |  |  |  |  |  |  |  |  |
| $81-2 | | 4 | 1 | 6.34% | 30370.5 | 9 | tr\|A0A0J9YXP8\|A0A0J9YXP8_HUMAN Glucose-6-phosphate isomerase (Fragment) OS=Homo sapiens GN=GPI PE=1 SV=1 |  |  |  |  |  |  |  |  |
| $81-3 | | 4 | 1 | 6.01% | 32048.4 | 9.6 | tr\|A0A0J9YYH3\|A0A0J9YYH3_HUMAN Glucose-6-phosphate isomerase (Fragment) OS=Homo sapiens GN=GPI PE=1 SV=1 |  |  |  |  |  |  |  |  |
| $81-4 | | 4 | 1 | 3.21% | 60185.7 | 8.2 | tr\|B4DE36\|B4DE36_HUMAN Glucose-6-phosphate isomerase OS=Homo sapiens PE=2 SV=1 |  |  |  |  |  |  |  |  |
| $81-5 | | 4 | 1 | 2.97% | 64825 | 9.1 | tr\|A0A0A0MTS2\|A0A0A0MTS2_HUMAN Glucose-6-phosphate isomerase (Fragment) OS=Homo sapiens GN=GPI PE=1 SV=1 |  |  |  |  |  |  |  |  |
| $81-6 | | 4 | 1 | 6.09% | 31548 | 9.6 | tr\|A0A0J9YX90\|A0A0J9YX90_HUMAN Glucose-6-phosphate isomerase (Fragment) OS=Homo sapiens GN=GPI PE=1 SV=1 |  |  |  |  |  |  |  |  |
| $81-7 | | 4 | 1 | 3.59% | 53401.8 | 8.8 | tr\|K7EQ48\|K7EQ48_HUMAN Glucose-6-phosphate isomerase OS=Homo sapiens GN=GPI PE=1 SV=2 |  |  |  |  |  |  |  |  |
| $81-8 | | 4 | 1 | 3.20% | 59990.4 | 8.8 | tr\|B4DVJ0\|B4DVJ0_HUMAN Glucose-6-phosphate isomerase OS=Homo sapiens PE=2 SV=1 |  |  |  |  |  |  |  |  |
|  | R16062_1_CTGF,17770 | K.TLAQLNPESSLFIIASK.T | 1833.1184 | 1.1204 | 2 | 1 | 2.257 | 0.296 | 417.1 | 1 | 14\|32 | 5.66 | 1 | 8 |  |
|  | R16062_1_CTGF,17779 | K.TLAQLNPESSLFIIASK.T | 1833.1184 | -0.2986 | 2 | 1 | 2.314 | 0.4165 | 413.8 | 1 | 15\|32 | 5.66 | 1 | 8 |  |
|  | R16062_1_CTGF,17798 | K.TLAQLNPESSLFIIASK.T | 1833.1184 | 0.7844 | 2 | 1 | 3.0726 | 0.5939 | 689 | 1 | 18\|32 | 5.66 | 1 | 8 |  |
|  | R16062_1_CTGF,17806 | K.TLAQLNPESSLFIIASK.T | 1833.1184 | 1.5054 | 2 | 1 | 2.3755 | 0.3965 | 407.1 | 1 | 14\|32 | 5.66 | 1 | 8 |  |
| $82-1 | | 3 | 1 | 2.20% | 67930.8 | 6 | sp\|P32455\|GBP1_HUMAN Guanylate-binding protein 1 OS=Homo sapiens GN=GBP1 PE=1 SV=2 |  |  |  |  |  |  |  |  |
| $82-2 | | 3 | 1 | 2.18% | 68114.1 | 6.1 | sp\|Q9H0R5\|GBP3_HUMAN Guanylate-binding protein 3 OS=Homo sapiens GN=GBP3 PE=1 SV=3 |  |  |  |  |  |  |  |  |
| $82-3 | | 3 | 1 | 3.64% | 41270.4 | 6.3 | tr\|B4DNS2\|B4DNS2_HUMAN cDNA FLJ51602, highly similar to Interferon-induced guanylate-binding protein 1 OS=Homo sapiens PE=2 SV=1 |  |  |  |  |  |  |  |  |
| $82-4 | | 3 | 1 | 2.34% | 63878 | 6 | tr\|B4E1L5\|B4E1L5_HUMAN cDNA FLJ51601, highly similar to Interferon-induced guanylate-binding protein 1 OS=Homo sapiens PE=2 SV=1 |  |  |  |  |  |  |  |  |
| $82-5 | | 3 | 1 | 2.20% | 67916.8 | 6 | tr\|Q5D1D5\|Q5D1D5_HUMAN Guanylate binding protein 1 OS=Homo sapiens PE=2 SV=1 |  |  |  |  |  |  |  |  |
| $82-6 | | 3 | 1 | 5.96% | 25419.1 | 5.4 | tr\|H3BNS1\|H3BNS1_HUMAN Guanylate-binding protein 3 (Fragment) OS=Homo sapiens GN=GBP3 PE=1 SV=1 |  |  |  |  |  |  |  |  |
|  | R16062_1_CTGF,17527 | K.GIQAEEILQTYLK.S | 1506.7255 | -0.8515 | 2 | 1 | 2.2716 | 0.3946 | 375.1 | 3 | 10\|24 | 4.53 | 1 | 6 |  |
|  | R16062_1_CTGF,17558 | K.GIQAEEILQTYLK.S | 1506.7255 | -0.8935 | 2 | 1 | 3.0926 | 0.4375 | 918 | 1 | 15\|24 | 4.53 | 1 | 6 |  |
|  | R16062_1_CTGF,17585 | K.GIQAEEILQTYLK.S | 1506.7255 | 0.9335 | 2 | 1 | 2.9484 | 0.4712 | 895.1 | 1 | 15\|24 | 4.53 | 1 | 6 |  |
| $83-1 | | 3 | 1 | 2.38% | 50140.7 | 9.1 | sp\|P68104\|EF1A1_HUMAN Elongation factor 1-alpha 1 OS=Homo sapiens GN=EEF1A1 PE=1 SV=1 |  |  |  |  |  |  |  |  |
| $83-2 | | 3 | 1 | 2.38% | 50470 | 9.1 | sp\|Q05639\|EF1A2_HUMAN Elongation factor 1-alpha 2 OS=Homo sapiens GN=EEF1A2 PE=1 SV=1 |  |  |  |  |  |  |  |  |
| $83-3 | | 3 | 1 | 2.38% | 50184.9 | 9.2 | sp\|Q5VTE0\|EF1A3_HUMAN Putative elongation factor 1-alpha-like 3 OS=Homo sapiens GN=EEF1A1P5 PE=5 SV=1 |  |  |  |  |  |  |  |  |
| $83-4 | | 3 | 1 | 2.76% | 42824.2 | 8.5 | tr\|Q16577\|Q16577_HUMAN Elongation factor 1-alpha 1 OS=Homo sapiens GN=PTI-1 PE=2 SV=1 |  |  |  |  |  |  |  |  |
| $83-5 | | 3 | 1 | 3.05% | 38668.5 | 9 | tr\|Q8IUB0\|Q8IUB0_HUMAN CTCL tumor antigen HD-CL-08 OS=Homo sapiens PE=2 SV=1 |  |  |  |  |  |  |  |  |
| $83-6 | | 3 | 1 | 2.38% | 50140.7 | 9.1 | tr\|Q6IPS9\|Q6IPS9_HUMAN Elongation factor 1-alpha OS=Homo sapiens GN=EEF1A1 PE=2 SV=1 |  |  |  |  |  |  |  |  |
| $83-7 | | 3 | 1 | 2.58% | 46269.1 | 9.1 | tr\|Q9NZS6\|Q9NZS6_HUMAN Elongation factor 1-alpha (Fragment) OS=Homo sapiens PE=2 SV=1 |  |  |  |  |  |  |  |  |
| $83-8 | | 3 | 1 | 2.38% | 50122.7 | 9.1 | tr\|Q6IPN6\|Q6IPN6_HUMAN Elongation factor 1-alpha OS=Homo sapiens GN=EEF1A1 PE=2 SV=1 |  |  |  |  |  |  |  |  |
| $83-9 | | 3 | 1 | 3.24% | 36911.1 | 7.8 | tr\|Q59GP5\|Q59GP5_HUMAN Eukaryotic translation elongation factor 1 alpha 2 variant (Fragment) OS=Homo sapiens PE=2 SV=1 |  |  |  |  |  |  |  |  |
| $83-10 | | 3 | 1 | 2.38% | 50200.8 | 9.1 | tr\|A8K9C4\|A8K9C4_HUMAN Elongation factor 1-alpha OS=Homo sapiens PE=2 SV=1 |  |  |  |  |  |  |  |  |
| $83-11 | | 3 | 1 | 2.49% | 47883.1 | 9.1 | tr\|A0A087WVQ9\|A0A087WVQ9_HUMAN Elongation factor 1-alpha 1 OS=Homo sapiens GN=EEF1A1 PE=1 SV=1 |  |  |  |  |  |  |  |  |
| $83-12 | | 3 | 1 | 4.40% | 26487.6 | 9.4 | tr\|Q8TBL1\|Q8TBL1_HUMAN Putative uncharacterized protein (Fragment) OS=Homo sapiens PE=2 SV=1 |  |  |  |  |  |  |  |  |
| $83-13 | | 3 | 1 | 4.44% | 26316.4 | 9.4 | tr\|Q96C29\|Q96C29_HUMAN Putative uncharacterized protein (Fragment) OS=Homo sapiens PE=2 SV=1 |  |  |  |  |  |  |  |  |
| $83-14 | | 3 | 1 | 2.38% | 50184.7 | 9.1 | tr\|Q6IPT9\|Q6IPT9_HUMAN Elongation factor 1-alpha OS=Homo sapiens GN=EEF1A1 PE=2 SV=1 |  |  |  |  |  |  |  |  |
| $83-15 | | 3 | 1 | 4.38% | 26616.7 | 9.4 | tr\|Q6P082\|Q6P082_HUMAN EEF1A1 protein (Fragment) OS=Homo sapiens GN=EEF1A1 PE=2 SV=1 |  |  |  |  |  |  |  |  |
| $83-16 | | 3 | 1 | 2.38% | 50112.6 | 9.1 | tr\|Q53G85\|Q53G85_HUMAN Elongation factor 1-alpha (Fragment) OS=Homo sapiens PE=2 SV=1 |  |  |  |  |  |  |  |  |
| $83-17 | | 3 | 1 | 2.76% | 43024.5 | 8.9 | tr\|Q96RE1\|Q96RE1_HUMAN Elongation factor 1-alpha OS=Homo sapiens GN=EEF1A1L14 PE=2 SV=1 |  |  |  |  |  |  |  |  |
| $83-18 | | 3 | 1 | 3.57% | 33242.2 | 9.1 | tr\|Q504Z0\|Q504Z0_HUMAN EEF1A1 protein OS=Homo sapiens GN=EEF1A1 PE=2 SV=1 |  |  |  |  |  |  |  |  |
| $83-19 | | 3 | 1 | 3.57% | 33189.2 | 8.9 | tr\|B4E2C5\|B4E2C5_HUMAN cDNA FLJ54032, highly similar to Elongation factor 1-alpha 1 OS=Homo sapiens PE=2 SV=1 |  |  |  |  |  |  |  |  |
| $83-20 | | 3 | 1 | 2.38% | 50110.7 | 9.1 | tr\|Q53GA1\|Q53GA1_HUMAN Elongation factor 1-alpha (Fragment) OS=Homo sapiens PE=2 SV=1 |  |  |  |  |  |  |  |  |
| $83-21 | | 3 | 1 | 3.01% | 39256.2 | 9 | tr\|Q53HR1\|Q53HR1_HUMAN Elongation factor 1-alpha (Fragment) OS=Homo sapiens PE=2 SV=1 |  |  |  |  |  |  |  |  |
| $83-22 | | 3 | 1 | 2.38% | 50110.7 | 9.1 | tr\|Q53GE9\|Q53GE9_HUMAN Elongation factor 1-alpha (Fragment) OS=Homo sapiens PE=2 SV=1 |  |  |  |  |  |  |  |  |
| $83-23 | | 3 | 1 | 2.38% | 50124.7 | 9.1 | tr\|Q53HR5\|Q53HR5_HUMAN Elongation factor 1-alpha (Fragment) OS=Homo sapiens PE=2 SV=1 |  |  |  |  |  |  |  |  |
| $83-24 | | 3 | 1 | 2.38% | 50198.7 | 9 | tr\|Q53HQ7\|Q53HQ7_HUMAN Elongation factor 1-alpha (Fragment) OS=Homo sapiens PE=2 SV=1 |  |  |  |  |  |  |  |  |
| $83-25 | | 3 | 1 | 2.78% | 42596.1 | 9.1 | tr\|B4DNE0\|B4DNE0_HUMAN cDNA FLJ52573, highly similar to Elongation factor 1-alpha 1 OS=Homo sapiens PE=2 SV=1 |  |  |  |  |  |  |  |  |
| $83-26 | | 3 | 1 | 2.54% | 46894 | 9.2 | tr\|Q53G89\|Q53G89_HUMAN Elongation factor 1-alpha (Fragment) OS=Homo sapiens PE=2 SV=1 |  |  |  |  |  |  |  |  |
| $83-27 | | 3 | 1 | 3.79% | 31059.8 | 9.2 | tr\|Q6P4C9\|Q6P4C9_HUMAN EEF1A1 protein (Fragment) OS=Homo sapiens GN=EEF1A1 PE=2 SV=1 |  |  |  |  |  |  |  |  |
| $83-28 | | 3 | 1 | 3.83% | 30700.4 | 9.3 | tr\|Q96CD8\|Q96CD8_HUMAN Putative uncharacterized protein (Fragment) OS=Homo sapiens PE=2 SV=1 |  |  |  |  |  |  |  |  |
| $83-29 | | 3 | 1 | 4.85% | 24196.2 | 9.6 | tr\|Q14222\|Q14222_HUMAN EEF1A protein (Fragment) OS=Homo sapiens GN=EEF1A PE=2 SV=1 |  |  |  |  |  |  |  |  |
| $83-30 | | 3 | 1 | 2.38% | 50141.6 | 9 | tr\|Q53HM9\|Q53HM9_HUMAN Elongation factor 1-alpha (Fragment) OS=Homo sapiens PE=2 SV=1 |  |  |  |  |  |  |  |  |
|  | R16062_1_CTGF,9925 | K.IGGIGTVPVGR.V | 1026.2149 | -0.7001 | 2 | 1 | 2.4377 | 0.2407 | 863.5 | 1 | 15\|20 | 9.75 | 1 | 30 |  |
|  | R16062_1_CTGF,9957 | K.IGGIGTVPVGR.V | 1026.2149 | 0.1699 | 2 | 1 | 2.9037 | 0.4563 | 685.8 | 1 | 13\|20 | 9.75 | 1 | 30 |  |
|  | R16062_1_CTGF,9969 | K.IGGIGTVPVGR.V | 1026.2149 | 0.4229 | 2 | 1 | 2.7786 | 0.4505 | 883.5 | 1 | 14\|20 | 9.75 | 1 | 30 |  |
| $84-1 | | 3 | 1 | 2.96% | 63367.7 | 6.6 | sp\|P21589\|5NTD_HUMAN 5'-nucleotidase OS=Homo sapiens GN=NT5E PE=1 SV=1 |  |  |  |  |  |  |  |  |
| $84-2 | | 3 | 1 | 6.44% | 28841.3 | 7.7 | tr\|Q96B60\|Q96B60_HUMAN 5'-nucleotidase OS=Homo sapiens GN=NT5E PE=1 SV=1 |  |  |  |  |  |  |  |  |
| $84-3 | | 3 | 1 | 2.96% | 63337.7 | 6.6 | tr\|Q53Z63\|Q53Z63_HUMAN 5'-nucleotidase OS=Homo sapiens GN=NT5E PE=2 SV=1 |  |  |  |  |  |  |  |  |
| $84-4 | | 3 | 1 | 2.96% | 63395.7 | 6.6 | tr\|B2RBH2\|B2RBH2_HUMAN cDNA, FLJ95508, highly similar to Homo sapiens 5'-nucleotidase, ecto (CD73) (NT5E), mRNA OS=Homo sapiens PE=2 SV=1 |  |  |  |  |  |  |  |  |
| $84-5 | | 3 | 1 | 2.96% | 63307.6 | 6.6 | tr\|Q6NZX3\|Q6NZX3_HUMAN 5'-nucleotidase, ecto (CD73) OS=Homo sapiens GN=NT5E PE=2 SV=1 |  |  |  |  |  |  |  |  |
|  | R16062_1_CTGF,15696 | K.VLPVGDEVVGIVGYTSK.E | 1732.9988 | -0.6752 | 2 | 1 | 2.36 | 0.4502 | 344 | 1 | 14\|32 | 4.37 | 1 | 5 |  |
|  | R16062_1_CTGF,15743 | K.VLPVGDEVVGIVGYTSK.E | 1732.9988 | 0.7698 | 2 | 1 | 2.8846 | 0.4784 | 611.8 | 1 | 18\|32 | 4.37 | 1 | 5 |  |
|  | R16062_1_CTGF,15800 | K.VLPVGDEVVGIVGYTSK.E | 1732.9988 | 0.0588 | 2 | 1 | 2.3548 | 0.3799 | 311.5 | 1 | 15\|32 | 4.37 | 1 | 5 |  |
| $85-1 | | 3 | 1 | 5.45% | 18897.8 | 10 | sp\|P46783\|RS10_HUMAN 40S ribosomal protein S10 OS=Homo sapiens GN=RPS10 PE=1 SV=1 |  |  |  |  |  |  |  |  |
| $85-2 | | 3 | 1 | 5.23% | 19868.1 | 9.9 | tr\|F6U211\|F6U211_HUMAN 40S ribosomal protein S10 OS=Homo sapiens GN=RPS10 PE=4 SV=1 |  |  |  |  |  |  |  |  |
| $85-3 | | 3 | 1 | 5.17% | 19859.9 | 9.9 | tr\|Q59GE4\|Q59GE4_HUMAN Ribosomal protein S10 variant (Fragment) OS=Homo sapiens PE=4 SV=1 |  |  |  |  |  |  |  |  |
| $85-4 | | 3 | 1 | 3.15% | 32596.3 | 9.2 | tr\|S4R435\|S4R435_HUMAN Protein RPS10-NUDT3 (Fragment) OS=Homo sapiens GN=RPS10-NUDT3 PE=3 SV=1 |  |  |  |  |  |  |  |  |
|  | R16062_1_CTGF,19418 | R.IAIYELLFK.E | 1110.3702 | -0.9028 | 2 | 1 | 2.6929 | 0.3661 | 1327.1 | 1 | 14\|16 | 6 | 1 | 4 |  |
|  | R16062_1_CTGF,19434 | R.IAIYELLFK.E | 1110.3702 | 0.3532 | 2 | 1 | 2.9531 | 0.51 | 1282.4 | 1 | 14\|16 | 6 | 1 | 4 |  |
|  | R16062_1_CTGF,19455 | R.IAIYELLFK.E | 1110.3702 | 0.2592 | 2 | 1 | 2.6658 | 0.524 | 932.5 | 1 | 13\|16 | 6 | 1 | 4 |  |
| $86-1 | | 2 | 1 | 2.00% | 50135.7 | 4.9 | sp\|Q71U36\|TBA1A_HUMAN Tubulin alpha-1A chain OS=Homo sapiens GN=TUBA1A PE=1 SV=1 |  |  |  |  |  |  |  |  |
| $86-2 | | 2 | 1 | 2.00% | 50151.7 | 4.9 | sp\|P68363\|TBA1B_HUMAN Tubulin alpha-1B chain OS=Homo sapiens GN=TUBA1B PE=1 SV=1 |  |  |  |  |  |  |  |  |
| $86-3 | | 2 | 1 | 2.00% | 49895.4 | 5 | sp\|Q9BQE3\|TBA1C_HUMAN Tubulin alpha-1C chain OS=Homo sapiens GN=TUBA1C PE=1 SV=1 |  |  |  |  |  |  |  |  |
| $86-4 | | 2 | 1 | 6.92% | 14401.3 | 5.3 | tr\|F8VS66\|F8VS66_HUMAN Tubulin alpha-1C chain OS=Homo sapiens GN=TUBA1C PE=4 SV=1 |  |  |  |  |  |  |  |  |
| $86-5 | | 2 | 1 | 2.16% | 46241.3 | 5 | tr\|B3KPS3\|B3KPS3_HUMAN cDNA FLJ32131 fis, clone PEBLM2000267, highly similar to Tubulin alpha-ubiquitous chain OS=Homo sapiens PE=2 SV=1 |  |  |  |  |  |  |  |  |
| $86-6 | | 2 | 1 | 1.73% | 57730.3 | 4.9 | tr\|F5H5D3\|F5H5D3_HUMAN Tubulin alpha-1C chain OS=Homo sapiens GN=TUBA1C PE=1 SV=1 |  |  |  |  |  |  |  |  |
| $86-7 | | 2 | 1 | 4.11% | 24221.2 | 5.1 | tr\|F8VQQ4\|F8VQQ4_HUMAN Tubulin alpha-1A chain (Fragment) OS=Homo sapiens GN=TUBA1A PE=1 SV=1 |  |  |  |  |  |  |  |  |
| $86-8 | | 2 | 1 | 8.04% | 12219.6 | 5.4 | tr\|F8VRZ4\|F8VRZ4_HUMAN Tubulin alpha-1A chain (Fragment) OS=Homo sapiens GN=TUBA1A PE=4 SV=1 |  |  |  |  |  |  |  |  |
| $86-9 | | 2 | 1 | 1.73% | 57760.3 | 4.9 | tr\|B7Z1K5\|B7Z1K5_HUMAN cDNA FLJ55956, highly similar to Tubulin alpha-6 chain OS=Homo sapiens PE=2 SV=1 |  |  |  |  |  |  |  |  |
| $86-10 | | 2 | 1 | 2.34% | 42729.6 | 5.1 | tr\|B4DQK4\|B4DQK4_HUMAN cDNA FLJ53743, highly similar to Tubulin alpha-3 chain OS=Homo sapiens PE=2 SV=1 |  |  |  |  |  |  |  |  |
| $86-11 | | 2 | 1 | 2.77% | 36648.6 | 8.2 | tr\|Q8N532\|Q8N532_HUMAN TUBA1C protein OS=Homo sapiens GN=TUBA1C PE=2 SV=1 |  |  |  |  |  |  |  |  |
| $86-12 | | 2 | 1 | 2.00% | 49823.3 | 5 | tr\|Q53GA7\|Q53GA7_HUMAN Tubulin alpha 6 variant (Fragment) OS=Homo sapiens PE=2 SV=1 |  |  |  |  |  |  |  |  |
| $86-13 | | 2 | 1 | 2.16% | 46341.4 | 5 | tr\|B3KT06\|B3KT06_HUMAN cDNA FLJ37398 fis, clone BRAMY2027467, highly similar to Tubulin alpha-ubiquitous chain OS=Homo sapiens PE=2 SV=1 |  |  |  |  |  |  |  |  |
| $86-14 | | 2 | 1 | 3.66% | 27465 | 5 | tr\|F8VVB9\|F8VVB9_HUMAN Tubulin alpha-1B chain (Fragment) OS=Homo sapiens GN=TUBA1B PE=1 SV=6 |  |  |  |  |  |  |  |  |
| $86-15 | | 2 | 1 | 2.00% | 50135.7 | 4.9 | tr\|A8JZY9\|A8JZY9_HUMAN cDNA FLJ78587 OS=Homo sapiens PE=2 SV=1 |  |  |  |  |  |  |  |  |
|  | R16062_1_CTGF,15757 | K.EIIDLVLDR.I | 1086.2639 | -0.3031 | 2 | 1 | 2.4856 | 0.3961 | 580.7 | 1 | 12\|16 | 4.03 | 1 | 15 |  |
|  | R16062_1_CTGF,15787 | K.EIIDLVLDR.I | 1086.2639 | 0.2599 | 2 | 1 | 2.3549 | 0.4344 | 914.4 | 1 | 14\|16 | 4.03 | 1 | 15 |  |
| $87-1 | | 2 | 1 | 4.49% | 36638.4 | 5.7 | sp\|P07195\|LDHB_HUMAN L-lactate dehydrogenase B chain OS=Homo sapiens GN=LDHB PE=1 SV=2 |  |  |  |  |  |  |  |  |
| $87-2 | | 2 | 1 | 4.49% | 36638.4 | 5.7 | tr\|Q5U077\|Q5U077_HUMAN L-lactate dehydrogenase OS=Homo sapiens GN=LDHB PE=2 SV=1 |  |  |  |  |  |  |  |  |
| $87-3 | | 2 | 1 | 11.03% | 14734.3 | 5.4 | tr\|C9J7H8\|C9J7H8_HUMAN L-lactate dehydrogenase B chain (Fragment) OS=Homo sapiens GN=LDHB PE=1 SV=6 |  |  |  |  |  |  |  |  |
| $87-4 | | 2 | 1 | 14.71% | 10806.6 | 5 | tr\|F5H793\|F5H793_HUMAN L-lactate dehydrogenase B chain (Fragment) OS=Homo sapiens GN=LDHB PE=1 SV=1 |  |  |  |  |  |  |  |  |
| $87-5 | | 2 | 1 | 6.47% | 25218 | 5.5 | tr\|A8MW50\|A8MW50_HUMAN L-lactate dehydrogenase (Fragment) OS=Homo sapiens GN=LDHB PE=1 SV=1 |  |  |  |  |  |  |  |  |
|  | R16062_1_CTGF,19390 | K.SLADELALVDVLEDK.L | 1630.8185 | 1.3405 | 2 | 1 | 3.1443 | 0.5297 | 876.3 | 1 | 16\|28 | 3.77 | 1 | 5 |  |
|  | R16062_1_CTGF,19410 | K.SLADELALVDVLEDK.L | 1630.8185 | 0.4495 | 2 | 1 | 2.3937 | 0.321 | 653.4 | 1 | 15\|28 | 3.77 | 1 | 5 |  |
| $88-1 | | 2 | 1 | 11.43% | 15054.1 | 8.4 | sp\|P07737\|PROF1_HUMAN Profilin-1 OS=Homo sapiens GN=PFN1 PE=1 SV=2 |  |  |  |  |  |  |  |  |
| $88-2 | | 2 | 1 | 9.70% | 17516.8 | 11 | tr\|I3L3D5\|I3L3D5_HUMAN Profilin-1 (Fragment) OS=Homo sapiens GN=PFN1 PE=1 SV=1 |  |  |  |  |  |  |  |  |
|  | R16062_1_CTGF,17088 | K.TFVNITPAEVGVLVGK.D | 1644.9364 | 0.3714 | 2 | 1 | 2.7372 | 0.3235 | 440 | 1 | 14\|30 | 5.66 | 1 | 2 |  |
|  | R16062_1_CTGF,17137 | K.TFVNITPAEVGVLVGK.D | 1644.9364 | 1.3714 | 2 | 1 | 3.1574 | 0.5006 | 756.1 | 1 | 16\|30 | 5.66 | 1 | 2 |  |
| $89-1 | | 2 | 1 | 7.35% | 22876.4 | 9.7 | sp\|P46782\|RS5_HUMAN 40S ribosomal protein S5 OS=Homo sapiens GN=RPS5 PE=1 SV=4 |  |  |  |  |  |  |  |  |
| $89-2 | | 2 | 1 | 7.35% | 22876.4 | 9.7 | tr\|A0A024R4Q8\|A0A024R4Q8_HUMAN Ribosomal protein S5, isoform CRA_a OS=Homo sapiens GN=RPS5 PE=3 SV=1 |  |  |  |  |  |  |  |  |
| $89-3 | | 2 | 1 | 6.67% | 25333.2 | 9.8 | tr\|M0R0R2\|M0R0R2_HUMAN 40S ribosomal protein S5 OS=Homo sapiens GN=RPS5 PE=1 SV=1 |  |  |  |  |  |  |  |  |
| $89-4 | | 2 | 1 | 7.50% | 22390.8 | 9.6 | tr\|M0R0F0\|M0R0F0_HUMAN 40S ribosomal protein S5 (Fragment) OS=Homo sapiens GN=RPS5 PE=1 SV=1 |  |  |  |  |  |  |  |  |
| $89-5 | | 2 | 1 | 11.19% | 14762.8 | 8.7 | tr\|M0QZN2\|M0QZN2_HUMAN 40S ribosomal protein S5 OS=Homo sapiens GN=RPS5 PE=1 SV=1 |  |  |  |  |  |  |  |  |
| $89-6 | | 2 | 1 | 7.35% | 22964.5 | 9.8 | tr\|Q53G25\|Q53G25_HUMAN Ribosomal protein S5 variant (Fragment) OS=Homo sapiens PE=2 SV=1 |  |  |  |  |  |  |  |  |
|  | R16062_1_CTGF,19331 | K.TIAECLADELINAAK.G | 1632.8311 | 0.0961 | 2 | 1 | 2.9634 | 0.3972 | 1211.9 | 1 | 19\|28 | 4.14 | 1 | 6 |  |
|  | R16062_1_CTGF,19352 | K.TIAECLADELINAAK.G | 1632.8311 | 0.9761 | 2 | 1 | 3.5125 | 0.3376 | 1017.8 | 1 | 17\|28 | 4.14 | 1 | 6 |  |
| $90-1 | | 2 | 1 | 2.55% | 80118.7 | 5.8 | sp\|P00736\|C1R_HUMAN Complement C1r subcomponent OS=Homo sapiens GN=C1R PE=1 SV=2 |  |  |  |  |  |  |  |  |
| $90-2 | | 2 | 1 | 2.50% | 81889.7 | 6 | tr\|B4DPQ0\|B4DPQ0_HUMAN Complement C1r subcomponent OS=Homo sapiens GN=C1R PE=1 SV=1 |  |  |  |  |  |  |  |  |
| $90-3 | | 2 | 1 | 4.79% | 42956.8 | 5.8 | tr\|D3DUT5\|D3DUT5_HUMAN Complement component 1, r subcomponent, isoform CRA_a OS=Homo sapiens GN=C1R PE=4 SV=1 |  |  |  |  |  |  |  |  |
| $90-4 | | 2 | 1 | 2.55% | 80300 | 5.9 | tr\|Q53HU9\|Q53HU9_HUMAN Complement component 1, r subcomponent variant (Fragment) OS=Homo sapiens PE=2 SV=1 |  |  |  |  |  |  |  |  |
| $90-5 | | 2 | 1 | 2.55% | 80198.9 | 6 | tr\|A8K5J8\|A8K5J8_HUMAN cDNA FLJ75066, highly similar to Homo sapiens complement component 1, r subcomponent (C1R), mRNA OS=Homo sapiens PE=2 SV=1 |  |  |  |  |  |  |  |  |
| $90-6 | | 2 | 1 | 2.68% | 76613.7 | 5.6 | tr\|F5H2D0\|F5H2D0_HUMAN Complement C1r subcomponent OS=Homo sapiens GN=C1R PE=1 SV=3 |  |  |  |  |  |  |  |  |
| $90-7 | | 2 | 1 | 2.55% | 80241 | 6 | tr\|Q53HT9\|Q53HT9_HUMAN Complement component 1, r subcomponent variant (Fragment) OS=Homo sapiens PE=2 SV=1 |  |  |  |  |  |  |  |  |
| $90-8 | | 2 | 1 | 3.01% | 68538.3 | 5.7 | tr\|B4E1B0\|B4E1B0_HUMAN cDNA FLJ54318, highly similar to Complement C1r subcomponent (EC 3.4.21.41) OS=Homo sapiens PE=2 SV=1 |  |  |  |  |  |  |  |  |
|  | R16062_1_CTGF,18438 | K.TLDEFTIIQNLQPQYQFR.D | 2255.5145 | 0.7145 | 2 | 1 | 2.3636 | 0.2831 | 258.6 | 2 | 12\|34 | 4.37 | 1 | 8 |  |
|  | R16062_1_CTGF,18455 | K.TLDEFTIIQNLQPQYQFR.D | 2255.5145 | 1.2505 | 2 | 1 | 2.6692 | 0.4986 | 699.3 | 1 | 18\|34 | 4.37 | 1 | 8 |  |
| $91-1 | | 2 | 1 | 7.28% | 22119.3 | 7.1 | sp\|P30043\|BLVRB_HUMAN Flavin reductase (NADPH) OS=Homo sapiens GN=BLVRB PE=1 SV=3 |  |  |  |  |  |  |  |  |
| $91-2 | | 2 | 1 | 9.20% | 17160.4 | 6.3 | tr\|M0R192\|M0R192_HUMAN Flavin reductase (NADPH) OS=Homo sapiens GN=BLVRB PE=1 SV=1 |  |  |  |  |  |  |  |  |
| $91-3 | | 2 | 1 | 7.28% | 22119.3 | 7.1 | tr\|V9HWI1\|V9HWI1_HUMAN Epididymis secretory protein Li 10 OS=Homo sapiens GN=HEL-S-10 PE=2 SV=1 |  |  |  |  |  |  |  |  |
| $91-4 | | 2 | 1 | 9.74% | 15669.7 | 5.2 | tr\|M0QZL1\|M0QZL1_HUMAN Flavin reductase (NADPH) OS=Homo sapiens GN=BLVRB PE=1 SV=1 |  |  |  |  |  |  |  |  |
|  | R16062_1_CTGF,16584 | K.TVAGQDAVIVLLGTR.N | 1513.7644 | 0.5114 | 2 | 1 | 2.7038 | 0.5234 | 717.7 | 1 | 14\|28 | 5.5 | 1 | 4 |  |
|  | R16062_1_CTGF,16597 | K.TVAGQDAVIVLLGTR.N | 1513.7644 | 0.3824 | 2 | 1 | 2.518 | 0.4323 | 492.9 | 1 | 12\|28 | 5.5 | 1 | 4 |  |
| $92-1 | | 2 | 1 | 5.76% | 32854.1 | 4.8 | sp\|P08865\|RSSA_HUMAN 40S ribosomal protein SA OS=Homo sapiens GN=RPSA PE=1 SV=4 |  |  |  |  |  |  |  |  |
| $92-2 | | 2 | 1 | 5.67% | 33313.7 | 4.8 | tr\|A0A0C4DG17\|A0A0C4DG17_HUMAN 40S ribosomal protein SA OS=Homo sapiens GN=RPSA PE=1 SV=1 |  |  |  |  |  |  |  |  |
| $92-3 | | 2 | 1 | 8.54% | 22014.4 | 9 | tr\|A0A024R7P5\|A0A024R7P5_HUMAN Similar to Laminin receptor 1, isoform CRA_a OS=Homo sapiens GN=LOC388524 PE=3 SV=1 |  |  |  |  |  |  |  |  |
| $92-4 | | 2 | 1 | 5.76% | 32909.1 | 4.8 | tr\|A6NE09\|A6NE09_HUMAN 40S ribosomal protein SA OS=Homo sapiens GN=RPSAP58 PE=1 SV=1 |  |  |  |  |  |  |  |  |
| $92-5 | | 2 | 1 | 5.76% | 32854.1 | 4.8 | tr\|A0A024R2P0\|A0A024R2P0_HUMAN 40S ribosomal protein SA OS=Homo sapiens GN=RPSA PE=3 SV=1 |  |  |  |  |  |  |  |  |
| $92-6 | | 2 | 1 | 6.46% | 29404.6 | 5.2 | tr\|C9J9K3\|C9J9K3_HUMAN 40S ribosomal protein SA (Fragment) OS=Homo sapiens GN=RPSA PE=1 SV=6 |  |  |  |  |  |  |  |  |
| $92-7 | | 2 | 1 | 5.76% | 32996.2 | 4.8 | tr\|Q96RS2\|Q96RS2_HUMAN 40S ribosomal protein SA OS=Homo sapiens GN=RPSA PE=2 SV=1 |  |  |  |  |  |  |  |  |
| $92-8 | | 2 | 1 | 14.66% | 12647.6 | 9.4 | tr\|F8WD59\|F8WD59_HUMAN 40S ribosomal protein SA (Fragment) OS=Homo sapiens GN=RPSA PE=1 SV=2 |  |  |  |  |  |  |  |  |
|  | R16062_1_CTGF,13761 | R.AIVAIENPADVSVISSR.N | 1741.9674 | -0.2946 | 2 | 1 | 2.7457 | 0.4431 | 396.8 | 1 | 17\|32 | 4.37 | 1 | 8 |  |
|  | R16062_1_CTGF,13800 | R.AIVAIENPADVSVISSR.N | 1741.9674 | -0.2406 | 2 | 1 | 2.7117 | 0.4814 | 529.8 | 1 | 17\|32 | 4.37 | 1 | 8 |  |
| $93-1 | | 2 | 1 | 2.03% | 84740.2 | 5.9 | tr\|D3DTX7\|D3DTX7_HUMAN Collagen, type I, alpha 1, isoform CRA_a OS=Homo sapiens GN=COL1A1 PE=4 SV=1 |  |  |  |  |  |  |  |  |
|  | R16062_1_CTGF,8524 | R.GETGPAGPAGPVGPVGAR.G | 1547.6978 | 0.3258 | 2 | 1 | 2.2418 | 0.2315 | 666.7 | 2 | 17\|34 | 6 | 1 | 1 |  |
|  | R16062_1_CTGF,8564 | R.GETGPAGPAGPVGPVGAR.G | 1547.6978 | 0.6358 | 2 | 1 | 2.5932 | 0.2777 | 822.7 | 1 | 18\|34 | 6 | 1 | 1 |  |
| $94-1 | | 2 | 1 | 2.29% | 50118.9 | 6.3 | sp\|P26641\|EF1G_HUMAN Elongation factor 1-gamma OS=Homo sapiens GN=EEF1G PE=1 SV=3 |  |  |  |  |  |  |  |  |
| $94-2 | | 2 | 1 | 2.29% | 50118.9 | 6.3 | tr\|Q53YD7\|Q53YD7_HUMAN EEF1G protein OS=Homo sapiens GN=EEF1G PE=2 SV=1 |  |  |  |  |  |  |  |  |
| $94-3 | | 2 | 1 | 4.81% | 22925.2 | 8.9 | tr\|B4DUK7\|B4DUK7_HUMAN cDNA FLJ59991, highly similar to Elongation factor 1-gamma OS=Homo sapiens PE=2 SV=1 |  |  |  |  |  |  |  |  |
| $94-4 | | 2 | 1 | 4.85% | 24104.3 | 6.5 | tr\|B4DUP0\|B4DUP0_HUMAN cDNA FLJ59433, highly similar to Elongation factor 1-gamma OS=Homo sapiens PE=2 SV=1 |  |  |  |  |  |  |  |  |
|  | R16062_1_CTGF,18090 | R.ILGLLDAYLK.T | 1119.3786 | 0.3936 | 2 | 1 | 2.3749 | 0.1777 | 463.8 | 4 | 11\|18 | 5.84 | 1 | 4 |  |
|  | R16062_1_CTGF,18098 | R.ILGLLDAYLK.T | 1119.3786 | 0.0966 | 2 | 1 | 2.3305 | 0.2456 | 690.3 | 1 | 14\|18 | 5.84 | 1 | 4 |  |
| $95-1 | | 2 | 1 | 3.52% | 32708.7 | 4.7 | sp\|P09493\|TPM1_HUMAN Tropomyosin alpha-1 chain OS=Homo sapiens GN=TPM1 PE=1 SV=2 |  |  |  |  |  |  |  |  |
| $95-2 | | 2 | 1 | 3.52% | 32850.9 | 4.7 | sp\|P07951\|TPM2_HUMAN Tropomyosin beta chain OS=Homo sapiens GN=TPM2 PE=1 SV=1 |  |  |  |  |  |  |  |  |
| $95-3 | | 2 | 1 | 3.51% | 32950.1 | 4.7 | sp\|P06753\|TPM3_HUMAN Tropomyosin alpha-3 chain OS=Homo sapiens GN=TPM3 PE=1 SV=2 |  |  |  |  |  |  |  |  |
| $95-4 | | 2 | 1 | 4.03% | 28522 | 4.7 | sp\|P67936\|TPM4_HUMAN Tropomyosin alpha-4 chain OS=Homo sapiens GN=TPM4 PE=1 SV=3 |  |  |  |  |  |  |  |  |
| $95-5 | | 2 | 1 | 3.52% | 32876.1 | 4.7 | tr\|D9YZV5\|D9YZV5_HUMAN Tropomyosin 1 (Alpha) isoform 4 OS=Homo sapiens GN=TPM1 PE=3 SV=1 |  |  |  |  |  |  |  |  |
| $95-6 | | 2 | 1 | 4.03% | 28608 | 4.8 | tr\|B7Z722\|B7Z722_HUMAN Tropomyosin 1 (Alpha), isoform CRA_i OS=Homo sapiens GN=TPM1 PE=2 SV=1 |  |  |  |  |  |  |  |  |
| $95-7 | | 2 | 1 | 4.03% | 28747.3 | 4.8 | tr\|H7BYY1\|H7BYY1_HUMAN Tropomyosin 1 (Alpha), isoform CRA_m OS=Homo sapiens GN=TPM1 PE=1 SV=1 |  |  |  |  |  |  |  |  |
| $95-8 | | 2 | 1 | 3.64% | 31753.1 | 4.9 | tr\|B7Z596\|B7Z596_HUMAN Tropomyosin alpha-1 chain OS=Homo sapiens GN=TPM1 PE=1 SV=1 |  |  |  |  |  |  |  |  |
| $95-9 | | 2 | 1 | 4.03% | 28522 | 4.7 | tr\|V9HW56\|V9HW56_HUMAN Epididymis secretory protein Li 108 OS=Homo sapiens GN=HEL-S-108 PE=2 SV=1 |  |  |  |  |  |  |  |  |
| $95-10 | | 2 | 1 | 3.07% | 37453.1 | 4.7 | tr\|Q6ZN40\|Q6ZN40_HUMAN Tropomyosin 1 (Alpha), isoform CRA_f OS=Homo sapiens GN=TPM1 PE=1 SV=1 |  |  |  |  |  |  |  |  |
| $95-11 | | 2 | 1 | 4.31% | 27175.7 | 4.7 | tr\|Q5HYB6\|Q5HYB6_HUMAN Epididymis luminal protein 189 OS=Homo sapiens GN=DKFZp686J1372 PE=1 SV=1 |  |  |  |  |  |  |  |  |
| $95-12 | | 2 | 1 | 3.52% | 32866 | 4.7 | tr\|A0A0K0K1I0\|A0A0K0K1I0_HUMAN Epididymis secretory protein Li 265 OS=Homo sapiens GN=HEL-S-265 PE=2 SV=1 |  |  |  |  |  |  |  |  |
| $95-13 | | 2 | 1 | 3.30% | 34402.5 | 5.4 | tr\|Q59GR8\|Q59GR8_HUMAN TPM1 protein variant (Fragment) OS=Homo sapiens PE=2 SV=1 |  |  |  |  |  |  |  |  |
| $95-14 | | 2 | 1 | 5.59% | 20621.1 | 4.6 | tr\|K7ENT6\|K7ENT6_HUMAN Tropomyosin alpha-4 chain (Fragment) OS=Homo sapiens GN=TPM4 PE=1 SV=1 |  |  |  |  |  |  |  |  |
| $95-15 | | 2 | 1 | 3.52% | 32677.7 | 4.7 | tr\|A0A024R5W6\|A0A024R5W6_HUMAN Tropomyosin 1 (Alpha), isoform CRA_a OS=Homo sapiens GN=TPM1 PE=3 SV=1 |  |  |  |  |  |  |  |  |
| $95-16 | | 2 | 1 | 3.52% | 33026.1 | 4.7 | tr\|A7XZE4\|A7XZE4_HUMAN Beta tropomyosin isoform OS=Homo sapiens GN=TPM2b PE=2 SV=1 |  |  |  |  |  |  |  |  |
| $95-17 | | 2 | 1 | 10.53% | 11017.3 | 4.8 | tr\|D6R904\|D6R904_HUMAN Tropomyosin alpha-3 chain OS=Homo sapiens GN=TPM3 PE=1 SV=1 |  |  |  |  |  |  |  |  |
| $95-18 | | 2 | 1 | 3.51% | 33222.7 | 4.7 | tr\|J3KN67\|J3KN67_HUMAN Tropomyosin alpha-3 chain OS=Homo sapiens GN=TPM3 PE=1 SV=1 |  |  |  |  |  |  |  |  |
| $95-19 | | 2 | 1 | 5.88% | 19306.7 | 4.8 | tr\|K7ERG3\|K7ERG3_HUMAN Tropomyosin alpha-4 chain (Fragment) OS=Homo sapiens GN=TPM4 PE=1 SV=1 |  |  |  |  |  |  |  |  |
| $95-20 | | 2 | 1 | 4.03% | 28580 | 4.7 | tr\|H0YK48\|H0YK48_HUMAN Tropomyosin alpha-1 chain OS=Homo sapiens GN=TPM1 PE=1 SV=1 |  |  |  |  |  |  |  |  |
| $95-21 | | 2 | 1 | 6.33% | 17833 | 4.8 | tr\|B4DTK3\|B4DTK3_HUMAN cDNA FLJ57891, highly similar to Tropomyosin beta chain OS=Homo sapiens PE=2 SV=1 |  |  |  |  |  |  |  |  |
| $95-22 | | 2 | 1 | 14.71% | 8014.11 | 4.8 | tr\|H0YL80\|H0YL80_HUMAN Tropomyosin alpha-1 chain (Fragment) OS=Homo sapiens GN=TPM1 PE=1 SV=1 |  |  |  |  |  |  |  |  |
| $95-23 | | 2 | 1 | 1.38% | 82419.7 | 4.9 | tr\|M1VPF4\|M1VPF4_HUMAN Tyrosine-protein kinase receptor OS=Homo sapiens GN=TPM3-ROS1 PE=2 SV=1 |  |  |  |  |  |  |  |  |
| $95-24 | | 2 | 1 | 4.08% | 28385.7 | 4.7 | tr\|D9YZV7\|D9YZV7_HUMAN Tropomyosin 1 (Alpha) isoform 6 OS=Homo sapiens GN=TPM1 PE=3 SV=1 |  |  |  |  |  |  |  |  |
| $95-25 | | 2 | 1 | 5.32% | 21833.6 | 4.8 | tr\|B4DGC2\|B4DGC2_HUMAN cDNA FLJ56690, highly similar to Tropomyosin beta chain OS=Homo sapiens PE=2 SV=1 |  |  |  |  |  |  |  |  |
| $95-26 | | 2 | 1 | 4.12% | 28420.3 | 4.9 | tr\|Q15657\|Q15657_HUMAN Tropomyosin isoform OS=Homo sapiens PE=2 SV=1 |  |  |  |  |  |  |  |  |
| $95-27 | | 2 | 1 | 3.11% | 36747.3 | 4.7 | tr\|Q5TCU8\|Q5TCU8_HUMAN Tropomyosin beta chain OS=Homo sapiens GN=TPM2 PE=1 SV=1 |  |  |  |  |  |  |  |  |
| $95-28 | | 2 | 1 | 4.41% | 26420.9 | 4.8 | tr\|A0A087WWU8\|A0A087WWU8_HUMAN Tropomyosin alpha-3 chain OS=Homo sapiens GN=TPM3 PE=1 SV=1 |  |  |  |  |  |  |  |  |
| $95-29 | | 2 | 1 | 3.52% | 32990 | 4.6 | tr\|V9HW25\|V9HW25_HUMAN Epididymis secretory protein Li 273 OS=Homo sapiens GN=HEL-S-273 PE=2 SV=1 |  |  |  |  |  |  |  |  |
| $95-30 | | 2 | 1 | 4.93% | 23754.6 | 4.7 | tr\|Q8TCG4\|Q8TCG4_HUMAN TPMsk1 (Fragment) OS=Homo sapiens GN=TPM1 PE=2 SV=1 |  |  |  |  |  |  |  |  |
| $95-31 | | 2 | 1 | 4.48% | 25470.5 | 4.6 | tr\|H0YNC7\|H0YNC7_HUMAN Tropomyosin alpha-1 chain (Fragment) OS=Homo sapiens GN=TPM1 PE=1 SV=1 |  |  |  |  |  |  |  |  |
| $95-32 | | 2 | 1 | 4.08% | 28508.9 | 4.8 | tr\|Q1ZYL5\|Q1ZYL5_HUMAN Tropomyosin 1 alpha variant 6 OS=Homo sapiens PE=2 SV=1 |  |  |  |  |  |  |  |  |
| $95-33 | | 2 | 1 | 4.08% | 28525 | 4.8 | tr\|F5H7S3\|F5H7S3_HUMAN Tropomyosin alpha-1 chain OS=Homo sapiens GN=TPM1 PE=1 SV=2 |  |  |  |  |  |  |  |  |
| $95-34 | | 2 | 1 | 3.77% | 30380.9 | 4.6 | tr\|H0YL52\|H0YL52_HUMAN Tropomyosin alpha-1 chain (Fragment) OS=Homo sapiens GN=TPM1 PE=1 SV=1 |  |  |  |  |  |  |  |  |
| $95-35 | | 2 | 1 | 4.37% | 26221.4 | 4.6 | tr\|B4DVY2\|B4DVY2_HUMAN cDNA FLJ54184, highly similar to Tropomyosin alpha-4 chain OS=Homo sapiens PE=2 SV=1 |  |  |  |  |  |  |  |  |
| $95-36 | | 2 | 1 | 7.25% | 15849.1 | 5 | tr\|K7EP68\|K7EP68_HUMAN Tropomyosin alpha-4 chain (Fragment) OS=Homo sapiens GN=TPM4 PE=1 SV=1 |  |  |  |  |  |  |  |  |
| $95-37 | | 2 | 1 | 4.03% | 29017.8 | 4.7 | tr\|B2RDE1\|B2RDE1_HUMAN cDNA, FLJ96568, highly similar to Homo sapiens tropomyosin 3 (TPM3), mRNA OS=Homo sapiens PE=2 SV=1 |  |  |  |  |  |  |  |  |
| $95-38 | | 2 | 1 | 3.52% | 32708.7 | 4.7 | tr\|D9YZV4\|D9YZV4_HUMAN Tropomyosin 1 (Alpha) isoform 1 OS=Homo sapiens GN=TPM1 PE=3 SV=1 |  |  |  |  |  |  |  |  |
| $95-39 | | 2 | 1 | 3.52% | 32814.8 | 4.6 | tr\|Q5TCU3\|Q5TCU3_HUMAN Tropomyosin beta chain OS=Homo sapiens GN=TPM2 PE=1 SV=1 |  |  |  |  |  |  |  |  |
| $95-40 | | 2 | 1 | 6.49% | 17634.7 | 4.7 | tr\|B4DTB1\|B4DTB1_HUMAN cDNA FLJ52936, weakly similar to Tropomyosin alpha-4 chain OS=Homo sapiens PE=2 SV=1 |  |  |  |  |  |  |  |  |
| $95-41 | | 2 | 1 | 4.61% | 24883.9 | 4.7 | tr\|C9IZA2\|C9IZA2_HUMAN cDNA FLJ26372 fis, clone HRT06233 OS=Homo sapiens PE=2 SV=2 |  |  |  |  |  |  |  |  |
| $95-42 | | 2 | 1 | 7.04% | 16365.5 | 4.7 | tr\|H0YKX5\|H0YKX5_HUMAN Tropomyosin alpha-1 chain (Fragment) OS=Homo sapiens GN=TPM1 PE=1 SV=1 |  |  |  |  |  |  |  |  |
| $95-43 | | 2 | 1 | 6.37% | 17904 | 5 | tr\|H0YKP3\|H0YKP3_HUMAN Tropomyosin alpha-1 chain OS=Homo sapiens GN=TPM1 PE=1 SV=1 |  |  |  |  |  |  |  |  |
|  | R16062_1_CTGF,12116 | R.IQLVEEELDR.A | 1244.3766 | -0.8114 | 2 | 1 | 2.2305 | 0.1975 | 958.2 | 1 | 14\|18 | 4 | 1 | 43 |  |
|  | R16062_1_CTGF,12126 | R.IQLVEEELDR.A | 1244.3766 | 0.2336 | 2 | 1 | 2.2682 | 0.2653 | 582.6 | 2 | 11\|18 | 4 | 1 | 43 |  |
| $96-1 | | 2 | 1 | 2.03% | 61175.3 | 6.3 | sp\|Q9H223\|EHD4_HUMAN EH domain-containing protein 4 OS=Homo sapiens GN=EHD4 PE=1 SV=1 |  |  |  |  |  |  |  |  |
| $96-2 | | 2 | 1 | 2.03% | 61175.3 | 6.3 | tr\|A0A024R9N6\|A0A024R9N6_HUMAN EH-domain containing 4, isoform CRA_a OS=Homo sapiens GN=EHD4 PE=4 SV=1 |  |  |  |  |  |  |  |  |
| $96-3 | | 2 | 1 | 2.04% | 61113.4 | 8.4 | tr\|A0A087WUA5\|A0A087WUA5_HUMAN EH domain-containing protein 4 OS=Homo sapiens GN=EHD4 PE=1 SV=1 |  |  |  |  |  |  |  |  |
| $96-4 | | 2 | 1 | 2.03% | 61156.2 | 6.2 | tr\|A8K9B9\|A8K9B9_HUMAN cDNA FLJ77391, highly similar to Homo sapiens EH-domain containing 4 (EHD4), mRNA OS=Homo sapiens PE=2 SV=1 |  |  |  |  |  |  |  |  |
|  | R16062_1_CTGF,15439 | R.LFEAEAQDLFR.D | 1339.4781 | -0.5019 | 2 | 1 | 2.5336 | 0.3449 | 650.4 | 1 | 13\|20 | 4.14 | 1 | 4 |  |
|  | R16062_1_CTGF,15488 | R.LFEAEAQDLFR.D | 1339.4781 | 1.8381 | 2 | 1 | 2.2782 | 0.201 | 731.5 | 1 | 13\|20 | 4.14 | 1 | 4 |  |
| $97-1 | | 2 | 1 | 8.57% | 20019.7 | 5.5 | sp\|P02792\|FRIL_HUMAN Ferritin light chain OS=Homo sapiens GN=FTL PE=1 SV=2 |  |  |  |  |  |  |  |  |
| $97-2 | | 2 | 1 | 16.30% | 10345.9 | 5.6 | tr\|B1Q3B3\|B1Q3B3_HUMAN Ferritin (Fragment) OS=Homo sapiens GN=FTL PE=3 SV=1 |  |  |  |  |  |  |  |  |
|  | R16062_1_CTGF,16440 | R.LGGPEAGLGEYLFER.L | 1608.7764 | -0.6356 | 2 | 1 | 3.2209 | 0.5582 | 751 | 1 | 17\|28 | 4.25 | 1 | 2 |  |
|  | R16062_1_CTGF,16571 | R.LGGPEAGLGEYLFER.L | 1608.7764 | -0.2186 | 2 | 1 | 2.2455 | 0.4321 | 859.3 | 1 | 16\|28 | 4.25 | 1 | 2 |  |
| $98-1 | | 2 | 1 | 0.42% | 280739 | 5.7 | sp\|P21333\|FLNA_HUMAN Filamin-A OS=Homo sapiens GN=FLNA PE=1 SV=4 |  |  |  |  |  |  |  |  |
| $98-2 | | 2 | 1 | 0.42% | 278164 | 5.5 | sp\|O75369\|FLNB_HUMAN Filamin-B OS=Homo sapiens GN=FLNB PE=1 SV=2 |  |  |  |  |  |  |  |  |
| $98-3 | | 2 | 1 | 0.40% | 291022 | 5.7 | sp\|Q14315\|FLNC_HUMAN Filamin-C OS=Homo sapiens GN=FLNC PE=1 SV=3 |  |  |  |  |  |  |  |  |
| $98-4 | | 2 | 1 | 0.42% | 277505 | 5.7 | tr\|Q60FE6\|Q60FE6_HUMAN Filamin A OS=Homo sapiens GN=FLNA PE=2 SV=1 |  |  |  |  |  |  |  |  |
| $98-5 | | 2 | 1 | 0.42% | 280490 | 5.6 | tr\|A0A024R321\|A0A024R321_HUMAN Filamin B, beta (Actin binding protein 278), isoform CRA_a OS=Homo sapiens GN=FLNB PE=4 SV=1 |  |  |  |  |  |  |  |  |
| $98-6 | | 2 | 1 | 0.42% | 278226 | 5.7 | tr\|Q60FE5\|Q60FE5_HUMAN Filamin A OS=Homo sapiens GN=FLNA PE=1 SV=1 |  |  |  |  |  |  |  |  |
| $98-7 | | 2 | 1 | 0.48% | 245851 | 5.7 | tr\|A0A087WWY3\|A0A087WWY3_HUMAN Filamin-A OS=Homo sapiens GN=FLNA PE=1 SV=1 |  |  |  |  |  |  |  |  |
| $98-8 | | 2 | 1 | 0.42% | 276550 | 5.7 | tr\|Q5HY54\|Q5HY54_HUMAN Filamin-A OS=Homo sapiens GN=FLNA PE=1 SV=1 |  |  |  |  |  |  |  |  |
| $98-9 | | 2 | 1 | 1.82% | 66591.5 | 9.1 | tr\|F8WE98\|F8WE98_HUMAN Filamin-A (Fragment) OS=Homo sapiens GN=FLNA PE=1 SV=2 |  |  |  |  |  |  |  |  |
| $98-10 | | 2 | 1 | 0.48% | 245867 | 5.7 | tr\|A6NDY9\|A6NDY9_HUMAN Filamin A OS=Homo sapiens GN=FLNA PE=2 SV=4 |  |  |  |  |  |  |  |  |
|  | R16062_1_CTGF,19454 | R.LIALLEVLSQK.K | 1227.5186 | 0.0956 | 2 | 1 | 3.4039 | 0.4752 | 1256 | 1 | 15\|20 | 6 | 1 | 10 |  |
|  | R16062_1_CTGF,19475 | R.LIALLEVLSQK.K | 1227.5186 | 1.1476 | 2 | 1 | 2.5437 | 0.3533 | 627.4 | 1 | 13\|20 | 6 | 1 | 10 |  |
| $99-1 | | 2 | 1 | 7.09% | 29079.9 | 6.9 | sp\|Q96G25\|MED8_HUMAN Mediator of RNA polymerase II transcription subunit 8 OS=Homo sapiens GN=MED8 PE=1 SV=2 |  |  |  |  |  |  |  |  |
|  | R16062_1_CTGF,13679 | R.NQVIIPLVLSPDRDEDLMR.Q | 2224.5669 | -0.8501 | 2 | 1 | 2.4172 | 0.3241 | 422.5 | 2 | 13\|36 | 4.23 | 1 | 1 |  |
|  | R16062_1_CTGF,13731 | R.NQVIIPLVLSPDRDEDLMR.Q | 2224.5669 | -1.1031 | 2 | 1 | 2.3711 | 0.3029 | 710.4 | 1 | 15\|36 | 4.23 | 1 | 1 |  |
| $100-1 | | 2 | 1 | 1.79% | 68976.1 | 6.5 | sp\|Q7Z5L7\|PODN_HUMAN Podocan OS=Homo sapiens GN=PODN PE=1 SV=2 |  |  |  |  |  |  |  |  |
| $100-2 | | 2 | 1 | 2.74% | 45733.5 | 8.6 | tr\|B2RA94\|B2RA94_HUMAN cDNA, FLJ94779 OS=Homo sapiens PE=2 SV=1 |  |  |  |  |  |  |  |  |
| $100-3 | | 2 | 1 | 3.06% | 40671.9 | 9.9 | tr\|B4DUY6\|B4DUY6_HUMAN cDNA FLJ54652, highly similar to Homo sapiens podocan (PODN), mRNA OS=Homo sapiens PE=2 SV=1 |  |  |  |  |  |  |  |  |
|  | R16062_1_CTGF,11376 | R.SVDANVLTPIR.S | 1185.3556 | -0.7104 | 2 | 1 | 2.4392 | 0.1833 | 752.9 | 1 | 14\|20 | 5.55 | 1 | 3 |  |
|  | R16062_1_CTGF,11421 | R.SVDANVLTPIR.S | 1185.3556 | 0.3386 | 2 | 1 | 2.4508 | 0.2074 | 875.4 | 1 | 14\|20 | 5.55 | 1 | 3 |  |
| $101-1 | | 2 | 1 | 2.89% | 59750.7 | 9.2 | sp\|P25705\|ATPA_HUMAN ATP synthase subunit alpha, mitochondrial OS=Homo sapiens GN=ATP5A1 PE=1 SV=1 |  |  |  |  |  |  |  |  |
| $101-2 | | 2 | 1 | 2.89% | 59750.7 | 9.2 | tr\|V9HW26\|V9HW26_HUMAN ATP synthase subunit alpha OS=Homo sapiens GN=HEL-S-123m PE=2 SV=1 |  |  |  |  |  |  |  |  |
| $101-3 | | 2 | 1 | 11.11% | 15267.6 | 5.5 | tr\|K7EJP1\|K7EJP1_HUMAN ATP synthase subunit alpha, mitochondrial (Fragment) OS=Homo sapiens GN=ATP5A1 PE=1 SV=6 |  |  |  |  |  |  |  |  |
| $101-4 | | 2 | 1 | 9.58% | 17700.4 | 5.6 | tr\|B4DGW3\|B4DGW3_HUMAN cDNA FLJ54625, highly similar to ATP synthase subunit alpha, mitochondrial (EC 3.6.3.14) OS=Homo sapiens PE=2 SV=1 |  |  |  |  |  |  |  |  |
| $101-5 | | 2 | 1 | 14.41% | 11715.3 | 4.5 | tr\|K7EQH4\|K7EQH4_HUMAN ATP synthase subunit alpha, mitochondrial (Fragment) OS=Homo sapiens GN=ATP5A1 PE=1 SV=1 |  |  |  |  |  |  |  |  |
| $101-6 | | 2 | 1 | 7.73% | 22199.6 | 8.7 | tr\|K7EK77\|K7EK77_HUMAN ATP synthase subunit alpha, mitochondrial (Fragment) OS=Homo sapiens GN=ATP5A1 PE=1 SV=1 |  |  |  |  |  |  |  |  |
| $101-7 | | 2 | 1 | 7.80% | 21589.9 | 9.7 | tr\|K7ERX7\|K7ERX7_HUMAN ATP synthase subunit alpha, mitochondrial (Fragment) OS=Homo sapiens GN=ATP5A1 PE=1 SV=1 |  |  |  |  |  |  |  |  |
|  | R16062_1_CTGF,15763 | R.TGAIVDVPVGEELLGR.V | 1625.8484 | -0.9806 | 2 | 1 | 2.3388 | 0.3479 | 356.2 | 1 | 13\|30 | 4.14 | 1 | 7 |  |
|  | R16062_1_CTGF,15829 | R.TGAIVDVPVGEELLGR.V | 1625.8484 | -0.5986 | 2 | 1 | 2.952 | 0.3722 | 316.5 | 1 | 14\|30 | 4.14 | 1 | 7 |  |
| $102-1 | | 2 | 1 | 3.18% | 48121.4 | 5 | sp\|Q15084\|PDIA6_HUMAN Protein disulfide-isomerase A6 OS=Homo sapiens GN=PDIA6 PE=1 SV=1 |  |  |  |  |  |  |  |  |
|  | R16062_1_CTGF,18355 | R.TGEAIVDAALSALR.Q | 1387.5642 | 1.3282 | 2 | 1 | 2.667 | 0.3959 | 1710.7 | 1 | 19\|26 | 4.37 | 1 | 1 |  |
|  | R16062_1_CTGF,18384 | R.TGEAIVDAALSALR.Q | 1387.5642 | 0.4132 | 2 | 1 | 3.1512 | 0.4405 | 1609.8 | 1 | 20\|26 | 4.37 | 1 | 1 |  |
| $103-1 | | 2 | 1 | 0.86% | 229000 | 5.4 | sp\|P35580\|MYH10_HUMAN Myosin-10 OS=Homo sapiens GN=MYH10 PE=1 SV=3 |  |  |  |  |  |  |  |  |
| $103-2 | | 2 | 1 | 0.86% | 227340 | 5.4 | sp\|P35749\|MYH11_HUMAN Myosin-11 OS=Homo sapiens GN=MYH11 PE=1 SV=3 |  |  |  |  |  |  |  |  |
| $103-3 | | 2 | 1 | 0.87% | 226533 | 5.5 | sp\|P35579\|MYH9_HUMAN Myosin-9 OS=Homo sapiens GN=MYH9 PE=1 SV=4 |  |  |  |  |  |  |  |  |
| $103-4 | | 2 | 1 | 0.84% | 234237 | 5.6 | tr\|B1PS43\|B1PS43_HUMAN Myosin heavy chain 11 smooth muscle isoform OS=Homo sapiens GN=MYH11 PE=1 SV=1 |  |  |  |  |  |  |  |  |
| $103-5 | | 2 | 1 | 2.89% | 68244.5 | 5.2 | tr\|O14729\|O14729_HUMAN Smooth muscle myosin heavy chain SM2 (Fragment) OS=Homo sapiens PE=2 SV=1 |  |  |  |  |  |  |  |  |
| $103-6 | | 2 | 1 | 2.73% | 72006.4 | 5.1 | tr\|O14794\|O14794_HUMAN Smooth muscle myosin heavy chain SM1 (Fragment) OS=Homo sapiens PE=2 SV=1 |  |  |  |  |  |  |  |  |
| $103-7 | | 2 | 1 | 0.87% | 226533 | 5.5 | tr\|A0A024R1N1\|A0A024R1N1_HUMAN Myosin, heavy polypeptide 9, non-muscle, isoform CRA_a OS=Homo sapiens GN=MYH9 PE=4 SV=1 |  |  |  |  |  |  |  |  |
| $103-8 | | 2 | 1 | 3.41% | 57373.1 | 5.1 | tr\|Q66K75\|Q66K75_HUMAN MYH11 protein (Fragment) OS=Homo sapiens GN=MYH11 PE=2 SV=1 |  |  |  |  |  |  |  |  |
| $103-9 | | 2 | 1 | 0.86% | 227340 | 5.4 | tr\|A0A024QZJ4\|A0A024QZJ4_HUMAN Myosin, heavy polypeptide 11, smooth muscle, isoform CRA_a OS=Homo sapiens GN=MYH11 PE=4 SV=1 |  |  |  |  |  |  |  |  |
| $103-10 | | 2 | 1 | 0.88% | 223578 | 5.4 | tr\|A0A024QZJ6\|A0A024QZJ6_HUMAN Myosin, heavy polypeptide 11, smooth muscle, isoform CRA_b OS=Homo sapiens GN=MYH11 PE=4 SV=1 |  |  |  |  |  |  |  |  |
| $103-11 | | 2 | 1 | 3.02% | 65770.2 | 9 | tr\|Q6ZNL4\|Q6ZNL4_HUMAN FLJ00279 protein (Fragment) OS=Homo sapiens GN=FLJ00279 PE=2 SV=1 |  |  |  |  |  |  |  |  |
|  | R16062_1_CTGF,16132 | R.TQLEELEDELQATEDAK.L | 1963.0435 | 0.5295 | 2 | 1 | 3.5681 | 0.4676 | 1949.9 | 1 | 22\|32 | 3.71 | 1 | 11 |  |
|  | R16062_1_CTGF,16184 | R.TQLEELEDELQATEDAK.L | 1963.0435 | 1.1765 | 2 | 1 | 3.5149 | 0.5671 | 1289.3 | 1 | 18\|32 | 3.71 | 1 | 11 |  |
| $104-1 | | 2 | 1 | 2.65% | 51511.6 | 5.4 | sp\|P02679\|FIBG_HUMAN Fibrinogen gamma chain OS=Homo sapiens GN=FGG PE=1 SV=3 |  |  |  |  |  |  |  |  |
| $104-2 | | 2 | 1 | 3.59% | 37738 | 5.9 | tr\|D3DP16\|D3DP16_HUMAN Fibrinogen gamma chain, isoform CRA_a OS=Homo sapiens GN=FGG PE=4 SV=1 |  |  |  |  |  |  |  |  |
| $104-3 | | 2 | 1 | 2.60% | 52337.6 | 5.4 | tr\|C9JC84\|C9JC84_HUMAN Fibrinogen gamma chain OS=Homo sapiens GN=FGG PE=1 SV=1 |  |  |  |  |  |  |  |  |
| $104-4 | | 2 | 1 | 10.08% | 13546.5 | 6.7 | tr\|C9JPQ9\|C9JPQ9_HUMAN Fibrinogen gamma chain (Fragment) OS=Homo sapiens GN=FGG PE=1 SV=1 |  |  |  |  |  |  |  |  |
| $104-5 | | 2 | 1 | 3.01% | 45092.4 | 5.8 | tr\|Q7Z664\|Q7Z664_HUMAN Putative uncharacterized protein DKFZp779N0926 (Fragment) OS=Homo sapiens GN=DKFZp779N0926 PE=2 SV=1 |  |  |  |  |  |  |  |  |
| $104-6 | | 2 | 1 | 2.70% | 50322.4 | 5.7 | tr\|C9JEU5\|C9JEU5_HUMAN Fibrinogen gamma chain OS=Homo sapiens GN=FGG PE=1 SV=1 |  |  |  |  |  |  |  |  |
| $104-7 | | 2 | 1 | 9.76% | 13988 | 6.7 | tr\|C9JU00\|C9JU00_HUMAN Fibrinogen gamma chain (Fragment) OS=Homo sapiens GN=FGG PE=1 SV=1 |  |  |  |  |  |  |  |  |
|  | R16062_1_CTGF,9226 | R.YLQEIYNSNNQK.I | 1514.6214 | -0.7586 | 2 | 1 | 2.4347 | 0.2127 | 514.6 | 1 | 13\|22 | 6 | 1 | 7 |  |
|  | R16062_1_CTGF,9258 | R.YLQEIYNSNNQK.I | 1514.6214 | 0.3564 | 2 | 1 | 2.6057 | 0.31 | 565.2 | 1 | 13\|22 | 6 | 1 | 7 |  |
| $105-1 | | 1 | 1 | 16.51% | 11775.1 | 8.7 | sp\|P01620\|KV302_HUMAN Ig kappa chain V-III region SIE OS=Homo sapiens PE=1 SV=1 |  |  |  |  |  |  |  |  |
| $105-2 | | 1 | 1 | 16.51% | 11788.2 | 8.7 | sp\|P01622\|KV304_HUMAN Ig kappa chain V-III region Ti OS=Homo sapiens PE=1 SV=1 |  |  |  |  |  |  |  |  |
| $105-3 | | 1 | 1 | 16.51% | 11746.2 | 9.1 | sp\|P01623\|KV305_HUMAN Ig kappa chain V-III region WOL OS=Homo sapiens PE=1 SV=1 |  |  |  |  |  |  |  |  |
| $105-4 | | 1 | 1 | 16.51% | 11830.4 | 9.3 | sp\|P04206\|KV307_HUMAN Ig kappa chain V-III region GOL OS=Homo sapiens PE=1 SV=1 |  |  |  |  |  |  |  |  |
| $105-5 | | 1 | 1 | 16.82% | 11605.9 | 8.1 | tr\|G3GAU4\|G3GAU4_HUMAN Anti-H1N1 influenza HA kappa chain variable region (Fragment) OS=Homo sapiens PE=2 SV=1 |  |  |  |  |  |  |  |  |
| $105-6 | | 1 | 1 | 16.51% | 11646.1 | 8.6 | tr\|Q9UL78\|Q9UL78_HUMAN Myosin-reactive immunoglobulin light chain variable region (Fragment) OS=Homo sapiens PE=2 SV=1 |  |  |  |  |  |  |  |  |
| $105-7 | | 1 | 1 | 16.51% | 11888.3 | 9.2 | tr\|A2NB46\|A2NB46_HUMAN Cold agglutinin FS-2 L-chain (Fragment) OS=Homo sapiens PE=2 SV=1 |  |  |  |  |  |  |  |  |
|  | R16062_1_CTGF,13316 | -.EIVLTQSPGTLSLSPGER.A | 1885.1086 | 0.6236 | 2 | 1 | 2.9033 | 0.1407 | 808.7 | 1 | 16\|34 | 4.53 | 1 | 7 |  |
| $106-1 | | 1 | 1 | 13.19% | 11334.9 | 13 | tr\|L8E8D0\|L8E8D0_HUMAN Alternative protein ATRX OS=Homo sapiens GN=ATRX PE=4 SV=1 |  |  |  |  |  |  |  |  |
|  | R16062_1_CTGF,17870 | -.M*NSGPEQGLQRK.Q | 1361.5103 | 1.4703 | 2 | 1 | 2.2452 | 0.166 | 838 | 1 | 14\|22 | 8.5 | 1 | 1 |  |
| $107-1 | | 1 | 1 | 1.71% | 69413 | 5.9 | sp\|P15311\|EZRI_HUMAN Ezrin OS=Homo sapiens GN=EZR PE=1 SV=4 |  |  |  |  |  |  |  |  |
| $107-2 | | 1 | 1 | 1.73% | 67820.2 | 6.1 | sp\|P26038\|MOES_HUMAN Moesin OS=Homo sapiens GN=MSN PE=1 SV=3 |  |  |  |  |  |  |  |  |
| $107-3 | | 1 | 1 | 1.72% | 68564 | 6 | sp\|P35241\|RADI_HUMAN Radixin OS=Homo sapiens GN=RDX PE=1 SV=1 |  |  |  |  |  |  |  |  |
| $107-4 | | 1 | 1 | 3.04% | 38827 | 9.4 | tr\|Q6PJT4\|Q6PJT4_HUMAN MSN protein (Fragment) OS=Homo sapiens GN=MSN PE=2 SV=1 |  |  |  |  |  |  |  |  |
| $107-5 | | 1 | 1 | 1.72% | 68564 | 6 | tr\|B0YJ88\|B0YJ88_HUMAN Radixin OS=Homo sapiens GN=RDX PE=2 SV=1 |  |  |  |  |  |  |  |  |
| $107-6 | | 1 | 1 | 1.73% | 67820.2 | 6.1 | tr\|V9HWC0\|V9HWC0_HUMAN Epididymis luminal protein 70 OS=Homo sapiens GN=HEL70 PE=2 SV=1 |  |  |  |  |  |  |  |  |
| $107-7 | | 1 | 1 | 1.17% | 98948.7 | 5.8 | tr\|J7M2B1\|J7M2B1_HUMAN Tyrosine-protein kinase receptor OS=Homo sapiens GN=EZR-ROS1 PE=2 SV=1 |  |  |  |  |  |  |  |  |
| $107-8 | | 1 | 1 | 1.71% | 69401 | 5.9 | tr\|B2R6J2\|B2R6J2_HUMAN cDNA, FLJ92973, highly similar to Homo sapiens villin 2 (ezrin) (VIL2), mRNA OS=Homo sapiens PE=2 SV=1 |  |  |  |  |  |  |  |  |
| $107-9 | | 1 | 1 | 1.71% | 69241.8 | 5.9 | tr\|Q6NUR7\|Q6NUR7_HUMAN Ezrin OS=Homo sapiens GN=EZR PE=2 SV=1 |  |  |  |  |  |  |  |  |
| $107-10 | | 1 | 1 | 1.71% | 69371.9 | 5.9 | tr\|E7EQR4\|E7EQR4_HUMAN Ezrin OS=Homo sapiens GN=EZR PE=1 SV=3 |  |  |  |  |  |  |  |  |
| $107-11 | | 1 | 1 | 6.29% | 19014 | 9.2 | tr\|Q9UJZ6\|Q9UJZ6_HUMAN Ezrin (Fragment) OS=Homo sapiens PE=2 SV=1 |  |  |  |  |  |  |  |  |
| $107-12 | | 1 | 1 | 6.58% | 18196.2 | 9.7 | tr\|Q9UJZ2\|Q9UJZ2_HUMAN Cytovillin 2 (Fragment) OS=Homo sapiens GN=VIL2 PE=2 SV=1 |  |  |  |  |  |  |  |  |
| $107-13 | | 1 | 1 | 2.33% | 51267.1 | 5.8 | tr\|B7Z437\|B7Z437_HUMAN cDNA FLJ53435, highly similar to Ezrin OS=Homo sapiens PE=2 SV=1 |  |  |  |  |  |  |  |  |
| $107-14 | | 1 | 1 | 2.92% | 40725.2 | 9.4 | tr\|Q05CU6\|Q05CU6_HUMAN RDX protein (Fragment) OS=Homo sapiens GN=RDX PE=2 SV=1 |  |  |  |  |  |  |  |  |
| $107-15 | | 1 | 1 | 6.41% | 19042.2 | 9.5 | tr\|Q9UJZ8\|Q9UJZ8_HUMAN Ezrin (Fragment) OS=Homo sapiens PE=2 SV=1 |  |  |  |  |  |  |  |  |
| $107-16 | | 1 | 1 | 1.81% | 65569.5 | 5.7 | tr\|B7Z5V2\|B7Z5V2_HUMAN cDNA FLJ54141, highly similar to Ezrin OS=Homo sapiens PE=2 SV=1 |  |  |  |  |  |  |  |  |
| $107-17 | | 1 | 1 | 2.95% | 40396.9 | 9.5 | tr\|Q6PKD3\|Q6PKD3_HUMAN RDX protein (Fragment) OS=Homo sapiens GN=RDX PE=2 SV=1 |  |  |  |  |  |  |  |  |
| $107-18 | | 1 | 1 | 7.09% | 15469.8 | 9.3 | tr\|Q9UJU1\|Q9UJU1_HUMAN Cytovillin 2 (Fragment) OS=Homo sapiens GN=VIL2 PE=2 SV=1 |  |  |  |  |  |  |  |  |
| $107-19 | | 1 | 1 | 6.17% | 19328.5 | 9.4 | tr\|Q9UK20\|Q9UK20_HUMAN Ezrin (Fragment) OS=Homo sapiens PE=2 SV=2 |  |  |  |  |  |  |  |  |
| $107-20 | | 1 | 1 | 2.24% | 52650.7 | 5.9 | tr\|B7Z2S7\|B7Z2S7_HUMAN cDNA FLJ58499, highly similar to Radixin OS=Homo sapiens PE=2 SV=1 |  |  |  |  |  |  |  |  |
| $107-21 | | 1 | 1 | 1.71% | 69399 | 5.9 | tr\|V9HW42\|V9HW42_HUMAN Epididymis secretory protein Li 105 OS=Homo sapiens GN=HEL-S-105 PE=2 SV=1 |  |  |  |  |  |  |  |  |
|  | R16062_1_CTGF,14498 | K.APDFVFYAPR.L | 1183.3398 | 0.4338 | 2 | 1 | 2.496 | 0.411 | 756.6 | 1 | 13\|18 | 5.88 | 1 | 21 |  |
| $108-1 | | 1 | 1 | 0.88% | 117176 | 9.2 | sp\|Q86WZ0\|HEAT4_HUMAN HEAT repeat-containing protein 4 OS=Homo sapiens GN=HEATR4 PE=2 SV=2 |  |  |  |  |  |  |  |  |
|  | R16062_1_CTGF,11389 | K.DLLTHKILK.L | 1081.3336 | -0.8284 | 2 | 1 | 2.3037 | 0.1336 | 626.9 | 9 | 11\|16 | 8.6 | 1 | 1 |  |
| $109-1 | | 1 | 1 | 7.80% | 22677.7 | 5.9 | sp\|P62820\|RAB1A_HUMAN Ras-related protein Rab-1A OS=Homo sapiens GN=RAB1A PE=1 SV=3 |  |  |  |  |  |  |  |  |
| $109-2 | | 1 | 1 | 7.96% | 22171.1 | 5.6 | sp\|Q9H0U4\|RAB1B_HUMAN Ras-related protein Rab-1B OS=Homo sapiens GN=RAB1B PE=1 SV=1 |  |  |  |  |  |  |  |  |
| $109-3 | | 1 | 1 | 7.96% | 22016.9 | 5.3 | sp\|Q92928\|RAB1C_HUMAN Putative Ras-related protein Rab-1C OS=Homo sapiens GN=RAB1C PE=5 SV=2 |  |  |  |  |  |  |  |  |
| $109-4 | | 1 | 1 | 7.80% | 22677.7 | 5.9 | tr\|Q5U0I6\|Q5U0I6_HUMAN H.sapiens ras-related Hrab1A protein OS=Homo sapiens GN=RAB1A PE=2 SV=1 |  |  |  |  |  |  |  |  |
| $109-5 | | 1 | 1 | 7.96% | 22198.1 | 5.6 | tr\|Q6FIG4\|Q6FIG4_HUMAN RAB1B protein OS=Homo sapiens GN=RAB1B PE=2 SV=1 |  |  |  |  |  |  |  |  |
| $109-6 | | 1 | 1 | 17.02% | 9878.19 | 8.8 | tr\|Q96RD8\|Q96RD8_HUMAN GTP binding protein Rab1a (Fragment) OS=Homo sapiens PE=4 SV=1 |  |  |  |  |  |  |  |  |
| $109-7 | | 1 | 1 | 9.25% | 18989.7 | 6.3 | tr\|B7Z8M7\|B7Z8M7_HUMAN cDNA FLJ57768, highly similar to Ras-related protein Rab-1A OS=Homo sapiens PE=2 SV=1 |  |  |  |  |  |  |  |  |
| $109-8 | | 1 | 1 | 9.25% | 19018.5 | 7.6 | tr\|E7END7\|E7END7_HUMAN Ras-related protein Rab-1A OS=Homo sapiens GN=RAB1A PE=1 SV=1 |  |  |  |  |  |  |  |  |
| $109-9 | | 1 | 1 | 9.47% | 18483.1 | 5.6 | tr\|E9PLD0\|E9PLD0_HUMAN Ras-related protein Rab-1B OS=Homo sapiens GN=RAB1B PE=1 SV=1 |  |  |  |  |  |  |  |  |
|  | R16062_1_CTGF,18524 | K.EFADSLGIPFLETSAK.N | 1725.92 | -1.035 | 2 | 1 | 2.2999 | 0.38 | 288.1 | 1 | 13\|30 | 4.14 | 1 | 9 |  |
| $110-1 | | 1 | 1 | 2.92% | 68304.2 | 5.4 | sp\|P38606\|VATA_HUMAN V-type proton ATPase catalytic subunit A OS=Homo sapiens GN=ATP6V1A PE=1 SV=2 |  |  |  |  |  |  |  |  |
| $110-2 | | 1 | 1 | 5.39% | 37751.2 | 5.3 | tr\|B7Z2V6\|B7Z2V6_HUMAN cDNA FLJ53889, highly similar to Vacuolar ATP synthase catalytic subunit A, ubiquitous isoform (EC 3.6.3.14) OS=Homo sapiens PE=2 SV=1 |  |  |  |  |  |  |  |  |
|  | R16062_1_CTGF,20471 | K.EILQEEEDLAEIVQLVGK.A | 2056.2999 | 0.5729 | 2 | 1 | 3.3523 | 0.5564 | 1141.2 | 1 | 19\|34 | 3.83 | 1 | 2 |  |
| $111-1 | | 1 | 1 | 0.79% | 115386 | 6.6 | sp\|Q9BVI0\|PHF20_HUMAN PHD finger protein 20 OS=Homo sapiens GN=PHF20 PE=1 SV=2 |  |  |  |  |  |  |  |  |
| $111-2 | | 1 | 1 | 1.47% | 62051.8 | 9.1 | tr\|Q5JWZ0\|Q5JWZ0_HUMAN PHD finger protein 20 (Fragment) OS=Homo sapiens GN=PHF20 PE=1 SV=1 |  |  |  |  |  |  |  |  |
| $111-3 | | 1 | 1 | 1.46% | 62820.9 | 9.3 | tr\|Q5JXL1\|Q5JXL1_HUMAN PHD finger protein 20 (Fragment) OS=Homo sapiens GN=PHF20 PE=1 SV=2 |  |  |  |  |  |  |  |  |
| $111-4 | | 1 | 1 | 1.25% | 72559.5 | 8.9 | tr\|A0A0A0MT13\|A0A0A0MT13_HUMAN PHD finger protein 20 OS=Homo sapiens GN=PHF20 PE=1 SV=1 |  |  |  |  |  |  |  |  |
| $111-5 | | 1 | 1 | 1.45% | 62933 | 9.3 | tr\|Q7Z5E2\|Q7Z5E2_HUMAN Medulloblastoma antigen MU-MB-50.72 (Fragment) OS=Homo sapiens PE=2 SV=1 |  |  |  |  |  |  |  |  |
| $111-6 | | 1 | 1 | 5.19% | 17739.9 | 9.5 | tr\|B0QYY0\|B0QYY0_HUMAN PHD finger protein 20 (Fragment) OS=Homo sapiens GN=PHF20 PE=1 SV=1 |  |  |  |  |  |  |  |  |
|  | R16062_1_CTGF,9971 | K.ENISENDR.E | 976.9681 | -0.0259 | 2 | 1 | 3.0119 | 0.1607 | 727.5 | 1 | 13\|14 | 4.14 | 1 | 6 |  |
| $112-1 | | 1 | 1 | 11.27% | 15945 | 5.5 | sp\|Q14019\|COTL1_HUMAN Coactosin-like protein OS=Homo sapiens GN=COTL1 PE=1 SV=3 |  |  |  |  |  |  |  |  |
| $112-2 | | 1 | 1 | 21.92% | 8224.3 | 8 | tr\|H3BT58\|H3BT58_HUMAN Coactosin-like protein OS=Homo sapiens GN=COTL1 PE=1 SV=1 |  |  |  |  |  |  |  |  |
|  | R16062_1_CTGF,20198 | K.FALITWIGENVSGLQR.A | 1805.07 | -0.415 | 2 | 1 | 2.3673 | 0.2083 | 308.7 | 11 | 10\|30 | 6 | 1 | 2 |  |
| $113-1 | | 1 | 1 | 1.86% | 67209.1 | 5.5 | sp\|P32456\|GBP2_HUMAN Guanylate-binding protein 2 OS=Homo sapiens GN=GBP2 PE=1 SV=3 |  |  |  |  |  |  |  |  |
| $113-2 | | 1 | 1 | 2.29% | 54034.9 | 5.6 | tr\|Q8TCE5\|Q8TCE5_HUMAN GBP2 protein OS=Homo sapiens GN=GBP2 PE=2 SV=1 |  |  |  |  |  |  |  |  |
|  | R16062_1_CTGF,11522 | K.GQLVVNPEALK.I | 1168.3681 | -1.0169 | 2 | 1 | 2.2408 | 0.2492 | 948 | 1 | 14\|20 | 6 | 1 | 2 |  |
| $114-1 | | 1 | 1 | 3.94% | 38434.1 | 7.6 | sp\|Q14103\|HNRPD_HUMAN Heterogeneous nuclear ribonucleoprotein D0 OS=Homo sapiens GN=HNRNPD PE=1 SV=1 |  |  |  |  |  |  |  |  |
| $114-2 | | 1 | 1 | 4.58% | 32834.5 | 8.2 | tr\|A0A024RDF4\|A0A024RDF4_HUMAN Heterogeneous nuclear ribonucleoprotein D (AU-rich element RNA binding protein 1, 37kDa), isoform CRA_e OS=Homo sapiens GN=HNRPD PE=4 SV=1 |  |  |  |  |  |  |  |  |
| $114-3 | | 1 | 1 | 4.17% | 36271.9 | 8.1 | tr\|A0A024RDB4\|A0A024RDB4_HUMAN Heterogeneous nuclear ribonucleoprotein D (AU-rich element RNA binding protein 1, 37kDa), isoform CRA_c OS=Homo sapiens GN=HNRPD PE=4 SV=1 |  |  |  |  |  |  |  |  |
| $114-4 | | 1 | 1 | 3.94% | 38434.1 | 7.6 | tr\|A0A024RDF3\|A0A024RDF3_HUMAN Heterogeneous nuclear ribonucleoprotein D (AU-rich element RNA binding protein 1, 37kDa), isoform CRA_d OS=Homo sapiens GN=HNRPD PE=4 SV=1 |  |  |  |  |  |  |  |  |
| $114-5 | | 1 | 1 | 4.62% | 33612.1 | 8.6 | tr\|B4DTC3\|B4DTC3_HUMAN cDNA FLJ54150, highly similar to Heterogeneous nuclear ribonucleoprotein D0 OS=Homo sapiens PE=2 SV=1 |  |  |  |  |  |  |  |  |
| $114-6 | | 1 | 1 | 6.97% | 22962.4 | 9.4 | tr\|B4E0W4\|B4E0W4_HUMAN cDNA FLJ61020, highly similar to Heterogeneous nuclear ribonucleoprotein D0 OS=Homo sapiens PE=2 SV=1 |  |  |  |  |  |  |  |  |
| $114-7 | | 1 | 1 | 12.61% | 12553.5 | 8.6 | tr\|D6RF44\|D6RF44_HUMAN Heterogeneous nuclear ribonucleoprotein D0 (Fragment) OS=Homo sapiens GN=HNRNPD PE=1 SV=6 |  |  |  |  |  |  |  |  |
| $114-8 | | 1 | 1 | 4.90% | 31481.5 | 6.9 | tr\|Q12771\|Q12771_HUMAN P37 AUF1 OS=Homo sapiens PE=2 SV=1 |  |  |  |  |  |  |  |  |
| $114-9 | | 1 | 1 | 6.33% | 23076.5 | 5 | tr\|D6RAF8\|D6RAF8_HUMAN Heterogeneous nuclear ribonucleoprotein D0 (Fragment) OS=Homo sapiens GN=HNRNPD PE=1 SV=1 |  |  |  |  |  |  |  |  |
| $114-10 | | 1 | 1 | 5.38% | 29667.2 | 9.2 | tr\|H0Y8G5\|H0Y8G5_HUMAN Heterogeneous nuclear ribonucleoprotein D0 (Fragment) OS=Homo sapiens GN=HNRNPD PE=1 SV=6 |  |  |  |  |  |  |  |  |
| $114-11 | | 1 | 1 | 6.67% | 23811.2 | 9.6 | tr\|H0YA96\|H0YA96_HUMAN Heterogeneous nuclear ribonucleoprotein D0 (Fragment) OS=Homo sapiens GN=HNRNPD PE=1 SV=1 |  |  |  |  |  |  |  |  |
|  | R16062_1_CTGF,11798 | K.IFVGGLSPDTPEEK.I | 1489.6518 | 0.1058 | 2 | 1 | 2.2523 | 0.2409 | 591.8 | 2 | 14\|26 | 4.14 | 1 | 11 |  |
| $115-1 | | 1 | 1 | 2.28% | 77214.3 | 5.1 | sp\|P23142\|FBLN1_HUMAN Fibulin-1 OS=Homo sapiens GN=FBLN1 PE=1 SV=4 |  |  |  |  |  |  |  |  |
| $115-2 | | 1 | 1 | 2.50% | 70155.1 | 5.1 | tr\|B4DUV1\|B4DUV1_HUMAN Fibulin-1 OS=Homo sapiens PE=2 SV=1 |  |  |  |  |  |  |  |  |
| $115-3 | | 1 | 1 | 2.22% | 78329.5 | 5.2 | tr\|B1AHL2\|B1AHL2_HUMAN Fibulin-1 OS=Homo sapiens GN=FBLN1 PE=1 SV=1 |  |  |  |  |  |  |  |  |
| $115-4 | | 1 | 1 | 8.47% | 20470.9 | 5 | tr\|B1AHM9\|B1AHM9_HUMAN Fibulin-1 (Fragment) OS=Homo sapiens GN=FBLN1 PE=1 SV=1 |  |  |  |  |  |  |  |  |
| $115-5 | | 1 | 1 | 2.51% | 70577.7 | 5.6 | tr\|Q8NBH6\|Q8NBH6_HUMAN Fibulin-1 OS=Homo sapiens PE=2 SV=1 |  |  |  |  |  |  |  |  |
| $115-6 | | 1 | 1 | 8.74% | 20048.4 | 4.5 | tr\|H7C1M6\|H7C1M6_HUMAN Fibulin-1 (Fragment) OS=Homo sapiens GN=FBLN1 PE=1 SV=1 |  |  |  |  |  |  |  |  |
| $115-7 | | 1 | 1 | 11.51% | 15432.3 | 4.7 | tr\|B1AHM7\|B1AHM7_HUMAN Fibulin-1 (Fragment) OS=Homo sapiens GN=FBLN1 PE=1 SV=1 |  |  |  |  |  |  |  |  |
|  | R16062_1_CTGF,11968 | K.IIEVEEEQEDPYLNDR.C | 1992.0865 | 0.0625 | 2 | 1 | 2.3037 | 0.3695 | 615.3 | 1 | 14\|30 | 3.71 | 1 | 7 |  |
| $116-1 | | 1 | 1 | 7.47% | 26922.8 | 5.1 | sp\|O00299\|CLIC1_HUMAN Chloride intracellular channel protein 1 OS=Homo sapiens GN=CLIC1 PE=1 SV=4 |  |  |  |  |  |  |  |  |
| $116-2 | | 1 | 1 | 7.47% | 26922.8 | 5.1 | tr\|Q5SRT3\|Q5SRT3_HUMAN Chloride intracellular channel protein OS=Homo sapiens GN=CLIC1 PE=2 SV=2 |  |  |  |  |  |  |  |  |
| $116-3 | | 1 | 1 | 7.47% | 27014.9 | 5.1 | tr\|Q53FB0\|Q53FB0_HUMAN Chloride intracellular channel protein (Fragment) OS=Homo sapiens PE=2 SV=1 |  |  |  |  |  |  |  |  |
|  | R16062_1_CTGF,15983 | K.LAALNPESNTAGLDIFAK.F | 1846.074 | -0.88 | 2 | 1 | 2.6378 | 0.4311 | 426.8 | 1 | 15\|34 | 4.37 | 1 | 3 |  |
| $117-1 | | 1 | 1 | 2.19% | 59620.5 | 5.4 | sp\|P50990\|TCPQ_HUMAN T-complex protein 1 subunit theta OS=Homo sapiens GN=CCT8 PE=1 SV=4 |  |  |  |  |  |  |  |  |
| $117-2 | | 1 | 1 | 2.41% | 54106.2 | 5.2 | tr\|Q7Z759\|Q7Z759_HUMAN CCT8 protein OS=Homo sapiens GN=CCT8 PE=2 SV=1 |  |  |  |  |  |  |  |  |
| $117-3 | | 1 | 1 | 2.19% | 59650.5 | 5.4 | tr\|Q53HU0\|Q53HU0_HUMAN Chaperonin containing TCP1, subunit 8 (Theta) variant (Fragment) OS=Homo sapiens PE=2 SV=1 |  |  |  |  |  |  |  |  |
| $117-4 | | 1 | 1 | 3.72% | 35368.9 | 5.7 | tr\|H7C4C8\|H7C4C8_HUMAN T-complex protein 1 subunit theta (Fragment) OS=Homo sapiens GN=CCT8 PE=1 SV=1 |  |  |  |  |  |  |  |  |
|  | R16062_1_CTGF,14419 | K.LFVTNDAATILR.E | 1334.5462 | -0.9968 | 2 | 1 | 2.2633 | 0.238 | 909.4 | 1 | 13\|22 | 5.84 | 1 | 4 |  |
| $118-1 | | 1 | 1 | 3.26% | 37540.2 | 6.4 | sp\|P37837\|TALDO_HUMAN Transaldolase OS=Homo sapiens GN=TALDO1 PE=1 SV=2 |  |  |  |  |  |  |  |  |
| $118-2 | | 1 | 1 | 3.46% | 35328.8 | 9.1 | tr\|F2Z393\|F2Z393_HUMAN Transaldolase OS=Homo sapiens GN=TALDO1 PE=1 SV=1 |  |  |  |  |  |  |  |  |
|  | R16062_1_CTGF,12964 | K.LLGELLQDNAK.L | 1214.3937 | 0.9147 | 2 | 1 | 2.345 | 0.3044 | 870.1 | 2 | 13\|20 | 4.37 | 1 | 2 |  |
| $119-1 | | 1 | 1 | 2.74% | 51901.4 | 8.2 | sp\|Q01518\|CAP1_HUMAN Adenylyl cyclase-associated protein 1 OS=Homo sapiens GN=CAP1 PE=1 SV=5 |  |  |  |  |  |  |  |  |
| $119-2 | | 1 | 1 | 2.74% | 51673 | 8.1 | tr\|D3DPU2\|D3DPU2_HUMAN Adenylyl cyclase-associated protein OS=Homo sapiens GN=CAP1 PE=3 SV=1 |  |  |  |  |  |  |  |  |
| $119-3 | | 1 | 1 | 3.00% | 47390.2 | 8.4 | tr\|B4DNW7\|B4DNW7_HUMAN Adenylyl cyclase-associated protein OS=Homo sapiens PE=2 SV=1 |  |  |  |  |  |  |  |  |
| $119-4 | | 1 | 1 | 2.74% | 51614.9 | 8.3 | tr\|B2RDY9\|B2RDY9_HUMAN Adenylyl cyclase-associated protein OS=Homo sapiens PE=2 SV=1 |  |  |  |  |  |  |  |  |
| $119-5 | | 1 | 1 | 2.88% | 49080.1 | 8.3 | tr\|B4DI38\|B4DI38_HUMAN Adenylyl cyclase-associated protein OS=Homo sapiens PE=2 SV=1 |  |  |  |  |  |  |  |  |
| $119-6 | | 1 | 1 | 6.40% | 22734 | 6.7 | tr\|Q5T0R3\|Q5T0R3_HUMAN Adenylyl cyclase-associated protein (Fragment) OS=Homo sapiens GN=CAP1 PE=1 SV=1 |  |  |  |  |  |  |  |  |
| $119-7 | | 1 | 1 | 6.47% | 22531.8 | 6.7 | tr\|Q5T0R4\|Q5T0R4_HUMAN Adenylyl cyclase-associated protein (Fragment) OS=Homo sapiens GN=CAP1 PE=1 SV=6 |  |  |  |  |  |  |  |  |
| $119-8 | | 1 | 1 | 4.96% | 28634.8 | 7.1 | tr\|Q5T0R9\|Q5T0R9_HUMAN Adenylyl cyclase-associated protein (Fragment) OS=Homo sapiens GN=CAP1 PE=1 SV=6 |  |  |  |  |  |  |  |  |
| $119-9 | | 1 | 1 | 7.47% | 19202 | 6.5 | tr\|Q5T0R7\|Q5T0R7_HUMAN Adenylyl cyclase-associated protein (Fragment) OS=Homo sapiens GN=CAP1 PE=1 SV=1 |  |  |  |  |  |  |  |  |
| $119-10 | | 1 | 1 | 6.25% | 23248.6 | 6.7 | tr\|Q5T0R2\|Q5T0R2_HUMAN Adenylyl cyclase-associated protein (Fragment) OS=Homo sapiens GN=CAP1 PE=1 SV=1 |  |  |  |  |  |  |  |  |
| $119-11 | | 1 | 1 | 7.39% | 19416.2 | 6.2 | tr\|Q5T0R6\|Q5T0R6_HUMAN Adenylyl cyclase-associated protein (Fragment) OS=Homo sapiens GN=CAP1 PE=1 SV=1 |  |  |  |  |  |  |  |  |
| $119-12 | | 1 | 1 | 3.24% | 43706 | 8.7 | tr\|B4DNY3\|B4DNY3_HUMAN Adenylyl cyclase-associated protein OS=Homo sapiens PE=2 SV=1 |  |  |  |  |  |  |  |  |
| $119-13 | | 1 | 1 | 6.05% | 23930.4 | 7.8 | tr\|Q5T0R1\|Q5T0R1_HUMAN Adenylyl cyclase-associated protein (Fragment) OS=Homo sapiens GN=CAP1 PE=1 SV=1 |  |  |  |  |  |  |  |  |
| $119-14 | | 1 | 1 | 7.26% | 19809.7 | 7 | tr\|Q5T0R5\|Q5T0R5_HUMAN Adenylyl cyclase-associated protein (Fragment) OS=Homo sapiens GN=CAP1 PE=1 SV=6 |  |  |  |  |  |  |  |  |
|  | R16062_1_CTGF,15383 | K.LSDLLAPISEQIK.E | 1427.6683 | 0.5263 | 2 | 1 | 2.6395 | 0.4823 | 555.3 | 1 | 14\|24 | 4.37 | 1 | 14 |  |
| $120-1 | | 1 | 1 | 2.11% | 89322 | 5.1 | sp\|P55072\|TERA_HUMAN Transitional endoplasmic reticulum ATPase OS=Homo sapiens GN=VCP PE=1 SV=4 |  |  |  |  |  |  |  |  |
| $120-2 | | 1 | 1 | 2.11% | 89322 | 5.1 | tr\|V9HW80\|V9HW80_HUMAN Epididymis luminal protein 220 OS=Homo sapiens GN=HEL-S-70 PE=2 SV=1 |  |  |  |  |  |  |  |  |
| $120-3 | | 1 | 1 | 2.64% | 71065.9 | 4.9 | tr\|Q96IF9\|Q96IF9_HUMAN VCP protein (Fragment) OS=Homo sapiens GN=VCP PE=2 SV=2 |  |  |  |  |  |  |  |  |
| $120-4 | | 1 | 1 | 5.54% | 34391.8 | 6.1 | tr\|Q9HAP1\|Q9HAP1_HUMAN Valosin-containing protein (Fragment) OS=Homo sapiens GN=VCP PE=2 SV=1 |  |  |  |  |  |  |  |  |
|  | R16062_1_CTGF,21002 | K.NAPAIIFIDELDAIAPK.R | 1812.0992 | -0.2768 | 2 | 1 | 2.2796 | 0.3553 | 635.9 | 1 | 17\|32 | 4.03 | 1 | 4 |  |
| $121-1 | | 1 | 1 | 3.94% | 29978.5 | 9.1 | sp\|Q4G0S7\|CC152_HUMAN Coiled-coil domain-containing protein 152 OS=Homo sapiens GN=CCDC152 PE=2 SV=3 |  |  |  |  |  |  |  |  |
| $121-2 | | 1 | 1 | 3.94% | 29978.5 | 9.1 | tr\|A0A024R043\|A0A024R043_HUMAN HCG2006161, isoform CRA_a OS=Homo sapiens GN=hCG_2006161 PE=4 SV=1 |  |  |  |  |  |  |  |  |
|  | R16062_1_CTGF,15506 | K.NNILDIQLEK.S | 1200.367 | 1.022 | 2 | 1 | 2.264 | 0.1509 | 699.2 | 1 | 12\|18 | 4.37 | 1 | 2 |  |
| $122-1 | | 1 | 1 | 5.02% | 35882.7 | 5.8 | sp\|P09525\|ANXA4_HUMAN Annexin A4 OS=Homo sapiens GN=ANXA4 PE=1 SV=4 |  |  |  |  |  |  |  |  |
| $122-2 | | 1 | 1 | 4.98% | 36085 | 5.8 | tr\|Q6LES2\|Q6LES2_HUMAN Annexin (Fragment) OS=Homo sapiens GN=ANXA4 PE=2 SV=1 |  |  |  |  |  |  |  |  |
| $122-3 | | 1 | 1 | 5.35% | 33551.9 | 5.6 | tr\|Q6P452\|Q6P452_HUMAN Annexin OS=Homo sapiens GN=ANXA4 PE=1 SV=1 |  |  |  |  |  |  |  |  |
| $122-4 | | 1 | 1 | 7.11% | 25421 | 6 | tr\|Q59FK3\|Q59FK3_HUMAN Annexin (Fragment) OS=Homo sapiens PE=2 SV=1 |  |  |  |  |  |  |  |  |
| $122-5 | | 1 | 1 | 14.29% | 12747.7 | 8.5 | tr\|Q6MZI0\|Q6MZI0_HUMAN Annexin (Fragment) OS=Homo sapiens GN=DKFZp686H02120 PE=2 SV=1 |  |  |  |  |  |  |  |  |
| $122-6 | | 1 | 1 | 5.28% | 34459.2 | 5.7 | tr\|B4DE02\|B4DE02_HUMAN Annexin OS=Homo sapiens PE=2 SV=1 |  |  |  |  |  |  |  |  |
| $122-7 | | 1 | 1 | 4.98% | 36057 | 5.7 | tr\|V9HW59\|V9HW59_HUMAN Annexin OS=Homo sapiens GN=HEL-S-274 PE=2 SV=1 |  |  |  |  |  |  |  |  |
| $122-8 | | 1 | 1 | 5.35% | 33614 | 5.6 | tr\|B4DDZ4\|B4DDZ4_HUMAN Annexin OS=Homo sapiens PE=2 SV=1 |  |  |  |  |  |  |  |  |
|  | R16062_1_CTGF,19443 | K.SETSGSFEDALLAIVK.C | 1667.8389 | -0.4241 | 2 | 1 | 3.025 | 0.4147 | 679.5 | 1 | 14\|30 | 4.14 | 1 | 8 |  |
| $123-1 | | 1 | 1 | 2.76% | 57563.8 | 7.3 | sp\|Q9Y285\|SYFA_HUMAN Phenylalanine--tRNA ligase alpha subunit OS=Homo sapiens GN=FARSA PE=1 SV=3 |  |  |  |  |  |  |  |  |
| $123-2 | | 1 | 1 | 2.76% | 57563.8 | 7.3 | tr\|Q6IBR2\|Q6IBR2_HUMAN FARSLA protein OS=Homo sapiens GN=FARSLA PE=2 SV=1 |  |  |  |  |  |  |  |  |
| $123-3 | | 1 | 1 | 8.33% | 18388.8 | 5.4 | tr\|K7EK06\|K7EK06_HUMAN Phenylalanine--tRNA ligase alpha subunit (Fragment) OS=Homo sapiens GN=FARSA PE=1 SV=6 |  |  |  |  |  |  |  |  |
| $123-4 | | 1 | 1 | 5.96% | 26036.6 | 6.2 | tr\|K7ER16\|K7ER16_HUMAN Phenylalanine--tRNA ligase alpha subunit OS=Homo sapiens GN=FARSA PE=1 SV=1 |  |  |  |  |  |  |  |  |
| $123-5 | | 1 | 1 | 2.55% | 62395.4 | 7.4 | tr\|K7ER00\|K7ER00_HUMAN Phenylalanine--tRNA ligase alpha subunit OS=Homo sapiens GN=FARSA PE=1 SV=1 |  |  |  |  |  |  |  |  |
|  | R16062_1_CTGF,19618 | K.SLQALGEVIEAELR.S | 1528.7327 | 1.4567 | 2 | 1 | 2.5605 | 0.3018 | 611.4 | 1 | 14\|26 | 4.25 | 1 | 5 |  |
| $124-1 | | 1 | 1 | 4.25% | 50582.8 | 5 | sp\|P31150\|GDIA_HUMAN Rab GDP dissociation inhibitor alpha OS=Homo sapiens GN=GDI1 PE=1 SV=2 |  |  |  |  |  |  |  |  |
| $124-2 | | 1 | 1 | 4.27% | 50663.3 | 6.1 | sp\|P50395\|GDIB_HUMAN Rab GDP dissociation inhibitor beta OS=Homo sapiens GN=GDI2 PE=1 SV=2 |  |  |  |  |  |  |  |  |
| $124-3 | | 1 | 1 | 4.27% | 50663.3 | 6.1 | tr\|Q6IAT1\|Q6IAT1_HUMAN Epididymis secretory sperm binding protein Li 46e OS=Homo sapiens GN=GDI2 PE=2 SV=1 |  |  |  |  |  |  |  |  |
| $124-4 | | 1 | 1 | 4.23% | 51154 | 8.4 | tr\|B4DLV7\|B4DLV7_HUMAN cDNA FLJ60299, highly similar to Rab GDP dissociation inhibitor beta OS=Homo sapiens PE=2 SV=1 |  |  |  |  |  |  |  |  |
| $124-5 | | 1 | 1 | 7.22% | 29856.3 | 6.2 | tr\|Q5SX87\|Q5SX87_HUMAN Rab GDP dissociation inhibitor beta (Fragment) OS=Homo sapiens GN=GDI2 PE=1 SV=1 |  |  |  |  |  |  |  |  |
| $124-6 | | 1 | 1 | 4.56% | 46908.5 | 4.9 | tr\|B4DHX4\|B4DHX4_HUMAN cDNA FLJ52902, highly similar to Rab GDP dissociation inhibitor alpha OS=Homo sapiens PE=2 SV=1 |  |  |  |  |  |  |  |  |
| $124-7 | | 1 | 1 | 9.36% | 23114.9 | 9.2 | tr\|Q5SX91\|Q5SX91_HUMAN Rab GDP dissociation inhibitor beta (Fragment) OS=Homo sapiens GN=GDI2 PE=1 SV=1 |  |  |  |  |  |  |  |  |
|  | R16062_1_CTGF,19280 | K.SPYLYPLYGLGELPQGFAR.L | 2142.441 | 1.392 | 2 | 1 | 2.7578 | 0.5377 | 745.3 | 1 | 17\|36 | 5.72 | 1 | 7 |  |
| $125-1 | | 1 | 1 | 7.72% | 28082.4 | 4.8 | sp\|P31946\|1433B_HUMAN 14-3-3 protein beta/alpha OS=Homo sapiens GN=YWHAB PE=1 SV=3 |  |  |  |  |  |  |  |  |
| $125-2 | | 1 | 1 | 7.72% | 28082.4 | 4.8 | tr\|V9HWD6\|V9HWD6_HUMAN Epididymis secretory protein Li 1 OS=Homo sapiens GN=HEL-S-1 PE=2 SV=1 |  |  |  |  |  |  |  |  |
|  | R16062_1_CTGF,19096 | K.TAFDEAIAELDTLNEESYK.D | 2160.2772 | -0.4278 | 2 | 1 | 2.9805 | 0.5524 | 359 | 1 | 14\|36 | 3.77 | 1 | 2 |  |
| $126-1 | | 1 | 1 | 12.50% | 14728.3 | 9.9 | sp\|P62987\|RL40_HUMAN Ubiquitin-60S ribosomal protein L40 OS=Homo sapiens GN=UBA52 PE=1 SV=2 |  |  |  |  |  |  |  |  |
| $126-2 | | 1 | 1 | 10.26% | 17964.9 | 9.7 | sp\|P62979\|RS27A_HUMAN Ubiquitin-40S ribosomal protein S27a OS=Homo sapiens GN=RPS27A PE=1 SV=2 |  |  |  |  |  |  |  |  |
| $126-3 | | 1 | 1 | 6.99% | 25761.6 | 6.9 | sp\|P0CG47\|UBB_HUMAN Polyubiquitin-B OS=Homo sapiens GN=UBB PE=1 SV=1 |  |  |  |  |  |  |  |  |
| $126-4 | | 1 | 1 | 2.34% | 77038.4 | 7.2 | sp\|P0CG48\|UBC_HUMAN Polyubiquitin-C OS=Homo sapiens GN=UBC PE=1 SV=3 |  |  |  |  |  |  |  |  |
| $126-5 | | 1 | 1 | 12.50% | 14728.3 | 9.9 | tr\|Q3MIH3\|Q3MIH3_HUMAN Ubiquitin A-52 residue ribosomal protein fusion product 1 OS=Homo sapiens GN=UBA52 PE=2 SV=1 |  |  |  |  |  |  |  |  |
| $126-6 | | 1 | 1 | 10.46% | 17214.8 | 6.8 | tr\|B4DV12\|B4DV12_HUMAN Polyubiquitin-B OS=Homo sapiens GN=UBB PE=1 SV=1 |  |  |  |  |  |  |  |  |
| $126-7 | | 1 | 1 | 3.00% | 59944.8 | 7.1 | tr\|L8B4I8\|L8B4I8_HUMAN Ubiquitin C OS=Homo sapiens GN=UbC PE=4 SV=1 |  |  |  |  |  |  |  |  |
| $126-8 | | 1 | 1 | 10.26% | 17964.9 | 9.7 | tr\|B2RDW1\|B2RDW1_HUMAN Epididymis luminal protein 112 OS=Homo sapiens GN=RPS27A PE=2 SV=1 |  |  |  |  |  |  |  |  |
| $126-9 | | 1 | 1 | 6.99% | 25757.6 | 6.9 | tr\|Q5PY61\|Q5PY61_HUMAN Polyubiquitin-C OS=Homo sapiens GN=UBC PE=1 SV=1 |  |  |  |  |  |  |  |  |
| $126-10 | | 1 | 1 | 5.25% | 34304.4 | 6.9 | tr\|Q96C32\|Q96C32_HUMAN Polyubiquitin-C OS=Homo sapiens GN=UBC PE=1 SV=1 |  |  |  |  |  |  |  |  |
| $126-11 | | 1 | 1 | 6.99% | 25761.6 | 6.9 | tr\|Q5U5U6\|Q5U5U6_HUMAN Epididymis secretory protein Li 50 OS=Homo sapiens GN=UBB PE=1 SV=1 |  |  |  |  |  |  |  |  |
| $126-12 | | 1 | 1 | 17.20% | 10469.1 | 8 | tr\|J3QS39\|J3QS39_HUMAN Polyubiquitin-B (Fragment) OS=Homo sapiens GN=UBB PE=1 SV=1 |  |  |  |  |  |  |  |  |
| $126-13 | | 1 | 1 | 25.40% | 7132.12 | 5.2 | tr\|M0R1V7\|M0R1V7_HUMAN Ubiquitin-60S ribosomal protein L40 (Fragment) OS=Homo sapiens GN=UBA52 PE=1 SV=1 |  |  |  |  |  |  |  |  |
| $126-14 | | 1 | 1 | 9.47% | 19015.9 | 7.9 | tr\|F5GXK7\|F5GXK7_HUMAN Polyubiquitin-C (Fragment) OS=Homo sapiens GN=UBC PE=1 SV=2 |  |  |  |  |  |  |  |  |
| $126-15 | | 1 | 1 | 2.63% | 68491.6 | 7.1 | tr\|L8B4R0\|L8B4R0_HUMAN Ubiquitin C OS=Homo sapiens GN=UbC PE=4 SV=1 |  |  |  |  |  |  |  |  |
| $126-16 | | 1 | 1 | 37.21% | 4853.57 | 5.1 | tr\|J3QSA3\|J3QSA3_HUMAN Polyubiquitin-B (Fragment) OS=Homo sapiens GN=UBB PE=1 SV=1 |  |  |  |  |  |  |  |  |
| $126-17 | | 1 | 1 | 7.77% | 23162.6 | 6.2 | tr\|J3QKN0\|J3QKN0_HUMAN Polyubiquitin-B (Fragment) OS=Homo sapiens GN=UBB PE=1 SV=1 |  |  |  |  |  |  |  |  |
| $126-18 | | 1 | 1 | 10.26% | 17904.8 | 9.7 | tr\|Q5RKT7\|Q5RKT7_HUMAN Ribosomal protein S27a OS=Homo sapiens GN=RPS27A PE=1 SV=1 |  |  |  |  |  |  |  |  |
| $126-19 | | 1 | 1 | 11.94% | 15032.2 | 5.5 | tr\|F5GYU3\|F5GYU3_HUMAN Polyubiquitin-C (Fragment) OS=Homo sapiens GN=UBC PE=1 SV=1 |  |  |  |  |  |  |  |  |
| $126-20 | | 1 | 1 | 6.69% | 26873 | 8.7 | tr\|Q9UFQ0\|Q9UFQ0_HUMAN Putative uncharacterized protein DKFZp434K0435 (Fragment) OS=Homo sapiens GN=DKFZp434K0435 PE=2 SV=1 |  |  |  |  |  |  |  |  |
| $126-21 | | 1 | 1 | 3.00% | 59900.8 | 7.1 | tr\|L8B196\|L8B196_HUMAN Ubiquitin C OS=Homo sapiens GN=UbC PE=4 SV=1 |  |  |  |  |  |  |  |  |
| $126-22 | | 1 | 1 | 7.14% | 25154 | 9.4 | tr\|J3QLP7\|J3QLP7_HUMAN Protein UBBP4 OS=Homo sapiens GN=UBBP4 PE=1 SV=1 |  |  |  |  |  |  |  |  |
| $126-23 | | 1 | 1 | 1.22% | 147335 | 7.7 | tr\|Q59EM9\|Q59EM9_HUMAN Ubiquitin C variant (Fragment) OS=Homo sapiens PE=2 SV=1 |  |  |  |  |  |  |  |  |
| $126-24 | | 1 | 1 | 26.23% | 6875.82 | 4.9 | tr\|F5GZ39\|F5GZ39_HUMAN Polyubiquitin-C (Fragment) OS=Homo sapiens GN=UBC PE=4 SV=1 |  |  |  |  |  |  |  |  |
| $126-25 | | 1 | 1 | 2.29% | 78698.4 | 7.8 | tr\|Q66K58\|Q66K58_HUMAN Uncharacterized protein (Fragment) OS=Homo sapiens PE=2 SV=1 |  |  |  |  |  |  |  |  |
| $126-26 | | 1 | 1 | 2.34% | 77012.3 | 7.2 | tr\|L8B4Z6\|L8B4Z6_HUMAN Ubiquitin C OS=Homo sapiens GN=UbC PE=4 SV=1 |  |  |  |  |  |  |  |  |
| $126-27 | | 1 | 1 | 6.99% | 25796.6 | 8.8 | tr\|J3QRK5\|J3QRK5_HUMAN Protein UBBP4 OS=Homo sapiens GN=UBBP4 PE=1 SV=1 |  |  |  |  |  |  |  |  |
|  | R16062_1_CTGF,13198 | K.TITLEVEPSDTIENVK.A | 1788.9744 | 0.4664 | 2 | 1 | 2.3362 | 0.4286 | 372.6 | 1 | 13\|30 | 4 | 1 | 27 |  |
| $127-1 | | 1 | 1 | 1.61% | 80829.6 | 8 | sp\|O75382\|TRIM3_HUMAN Tripartite motif-containing protein 3 OS=Homo sapiens GN=TRIM3 PE=1 SV=2 |  |  |  |  |  |  |  |  |
| $127-2 | | 1 | 1 | 1.62% | 80549.5 | 8.3 | tr\|B7Z5Y8\|B7Z5Y8_HUMAN cDNA FLJ56028, highly similar to Tripartite motif-containing protein 3 OS=Homo sapiens PE=2 SV=1 |  |  |  |  |  |  |  |  |
| $127-3 | | 1 | 1 | 2.12% | 62277.3 | 9.4 | tr\|Q6ZTE7\|Q6ZTE7_HUMAN cDNA FLJ44731 fis, clone BRACE3025719, highly similar to Homo sapiens ring finger protein 22 (RNF22) OS=Homo sapiens PE=2 SV=1 |  |  |  |  |  |  |  |  |
|  | R16062_1_CTGF,11872 | K.TKIGAGRLMGPK.G | 1229.5214 | 0.3764 | 2 | 1 | 2.3294 | 0.1472 | 329.9 | 9 | 12\|22 | 11.17 | 1 | 3 |  |
| $128-1 | | 1 | 1 | 3.69% | 42741.8 | 5.9 | sp\|P30740\|ILEU_HUMAN Leukocyte elastase inhibitor OS=Homo sapiens GN=SERPINB1 PE=1 SV=1 |  |  |  |  |  |  |  |  |
| $128-2 | | 1 | 1 | 3.69% | 42741.8 | 5.9 | tr\|V9HWH1\|V9HWH1_HUMAN Epididymis luminal protein 57 OS=Homo sapiens GN=HEL57 PE=2 SV=1 |  |  |  |  |  |  |  |  |
| $128-3 | | 1 | 1 | 4.11% | 38685.2 | 6.2 | tr\|B4E3A8\|B4E3A8_HUMAN cDNA FLJ53963, highly similar to Leukocyte elastase inhibitor OS=Homo sapiens PE=2 SV=1 |  |  |  |  |  |  |  |  |
|  | R16062_1_CTGF,18434 | K.TYNFLPEFLVSTQK.T | 1687.9166 | 0.4786 | 2 | 1 | 2.5252 | 0.3779 | 369 | 1 | 13\|26 | 5.66 | 1 | 3 |  |
| $129-1 | | 1 | 1 | 3.08% | 52657.9 | 9 | sp\|Q9UEW3\|MARCO_HUMAN Macrophage receptor MARCO OS=Homo sapiens GN=MARCO PE=1 SV=1 |  |  |  |  |  |  |  |  |
| $129-2 | | 1 | 1 | 3.08% | 52657.9 | 9 | tr\|Q4ZG40\|Q4ZG40_HUMAN Macrophage receptor with collagenous structure OS=Homo sapiens GN=MARCO PE=2 SV=1 |  |  |  |  |  |  |  |  |
| $129-3 | | 1 | 1 | 3.23% | 49733.5 | 9 | tr\|B4DLJ6\|B4DLJ6_HUMAN cDNA FLJ56989, highly similar to Macrophage receptor MARCO OS=Homo sapiens PE=2 SV=1 |  |  |  |  |  |  |  |  |
|  | R16062_1_CTGF,13254 | K.VGAGTGQIWLDNVQCR.G | 1774.9526 | 0.4626 | 2 | 1 | 2.4743 | 0.4796 | 576.7 | 1 | 14\|30 | 5.8 | 1 | 3 |  |
| $130-1 | | 1 | 1 | 0.35% | 515605 | 6.6 | sp\|P04114\|APOB_HUMAN Apolipoprotein B-100 OS=Homo sapiens GN=APOB PE=1 SV=2 |  |  |  |  |  |  |  |  |
| $130-2 | | 1 | 1 | 0.35% | 515558 | 6.6 | tr\|C0JYY2\|C0JYY2_HUMAN Apolipoprotein B (Including Ag(X) antigen) OS=Homo sapiens GN=APOB PE=4 SV=1 |  |  |  |  |  |  |  |  |
| $130-3 | | 1 | 1 | 0.37% | 489833 | 6.7 | tr\|A0A087WTM7\|A0A087WTM7_HUMAN Apolipoprotein B-100 OS=Homo sapiens GN=APOB PE=1 SV=1 |  |  |  |  |  |  |  |  |
| $130-4 | | 1 | 1 | 0.99% | 183579 | 6.3 | tr\|Q59HB3\|Q59HB3_HUMAN Apolipoprotein B variant (Fragment) OS=Homo sapiens PE=2 SV=1 |  |  |  |  |  |  |  |  |
| $130-5 | | 1 | 1 | 0.37% | 489819 | 6.7 | tr\|E1A689\|E1A689_HUMAN Mutant Apo B 100 OS=Homo sapiens PE=2 SV=1 |  |  |  |  |  |  |  |  |
|  | R16062_1_CTGF,18482 | K.VNWEEEAASGLLTSLK.D | 1747.9272 | 0.6332 | 2 | 1 | 2.486 | 0.3803 | 400.7 | 1 | 13\|30 | 4.25 | 1 | 5 |  |
| $131-1 | | 1 | 1 | 0.33% | 501320 | 5.6 | sp\|P08519\|APOA_HUMAN Apolipoprotein(a) OS=Homo sapiens GN=LPA PE=1 SV=1 |  |  |  |  |  |  |  |  |
| $131-2 | | 1 | 1 | 1.85% | 90569 | 7 | sp\|P00747\|PLMN_HUMAN Plasminogen OS=Homo sapiens GN=PLG PE=1 SV=2 |  |  |  |  |  |  |  |  |
| $131-3 | | 1 | 1 | 0.74% | 226516 | 5.7 | tr\|Q1HP67\|Q1HP67_HUMAN Lipoprotein, Lp(A) OS=Homo sapiens GN=LPA PE=1 SV=1 |  |  |  |  |  |  |  |  |
| $131-4 | | 1 | 1 | 7.14% | 23006.4 | 7.1 | tr\|Q68DS2\|Q68DS2_HUMAN Putative uncharacterized protein DKFZp779M0222 (Fragment) OS=Homo sapiens GN=DKFZp779M0222 PE=2 SV=1 |  |  |  |  |  |  |  |  |
| $131-5 | | 1 | 1 | 3.69% | 44900 | 7.4 | tr\|B4DPH4\|B4DPH4_HUMAN cDNA FLJ58778, highly similar to Plasminogen (EC 3.4.21.7) OS=Homo sapiens PE=2 SV=1 |  |  |  |  |  |  |  |  |
| $131-6 | | 1 | 1 | 1.85% | 90539 | 7 | tr\|B2R7F8\|B2R7F8_HUMAN Plasminogen OS=Homo sapiens PE=2 SV=1 |  |  |  |  |  |  |  |  |
|  | R16062_1_CTGF,16488 | K.YILQGVTSWGLGCAR.P | 1681.9103 | -0.5327 | 2 | 1 | 2.2342 | 0.2747 | 268.1 | 12 | 10\|28 | 8.22 | 1 | 6 |  |
| $132-1 | | 1 | 1 | 5.21% | 21450.1 | 8.8 | sp\|P63000\|RAC1_HUMAN Ras-related C3 botulinum toxin substrate 1 OS=Homo sapiens GN=RAC1 PE=1 SV=1 |  |  |  |  |  |  |  |  |
| $132-2 | | 1 | 1 | 5.21% | 21428.8 | 7.5 | sp\|P15153\|RAC2_HUMAN Ras-related C3 botulinum toxin substrate 2 OS=Homo sapiens GN=RAC2 PE=1 SV=1 |  |  |  |  |  |  |  |  |
| $132-3 | | 1 | 1 | 5.21% | 21378.9 | 8.4 | sp\|P60763\|RAC3_HUMAN Ras-related C3 botulinum toxin substrate 3 OS=Homo sapiens GN=RAC3 PE=1 SV=1 |  |  |  |  |  |  |  |  |
| $132-4 | | 1 | 1 | 5.41% | 20799.1 | 8.2 | tr\|B1AH80\|B1AH80_HUMAN Ras-related C3 botulinum toxin substrate 2 OS=Homo sapiens GN=RAC2 PE=1 SV=1 |  |  |  |  |  |  |  |  |
| $132-5 | | 1 | 1 | 6.76% | 16796.7 | 9.1 | tr\|A4D2P2\|A4D2P2_HUMAN Ras-related C3 botulinum toxin substrate 1 (Rho family, small GTP binding protein Rac1) OS=Homo sapiens GN=RAC1 PE=3 SV=1 |  |  |  |  |  |  |  |  |
| $132-6 | | 1 | 1 | 5.21% | 21428.8 | 7.5 | tr\|A0A024R1P2\|A0A024R1P2_HUMAN Ras-related C3 botulinum toxin substrate 2 (Rho family, small GTP binding protein Rac2), isoform CRA_a OS=Homo sapiens GN=RAC2 PE=3 SV=1 |  |  |  |  |  |  |  |  |
| $132-7 | | 1 | 1 | 4.74% | 23467.3 | 8.9 | tr\|A4D2P0\|A4D2P0_HUMAN Ras-related C3 botulinum toxin substrate 1 (Rho family, small GTP binding protein Rac1) OS=Homo sapiens GN=RAC1 PE=2 SV=1 |  |  |  |  |  |  |  |  |
| $132-8 | | 1 | 1 | 5.21% | 21450.1 | 8.8 | tr\|A4D2P1\|A4D2P1_HUMAN Ras-related C3 botulinum toxin substrate 1 (Rho family, small GTP binding protein Rac1) OS=Homo sapiens GN=RAC1 PE=2 SV=1 |  |  |  |  |  |  |  |  |
| $132-9 | | 1 | 1 | 6.02% | 18502.4 | 6.1 | tr\|B1AH78\|B1AH78_HUMAN Ras-related C3 botulinum toxin substrate 2 (Fragment) OS=Homo sapiens GN=RAC2 PE=1 SV=1 |  |  |  |  |  |  |  |  |
| $132-10 | | 1 | 1 | 7.35% | 15411.9 | 6.8 | tr\|J3QLK0\|J3QLK0_HUMAN Ras-related C3 botulinum toxin substrate 3 (Fragment) OS=Homo sapiens GN=RAC3 PE=1 SV=1 |  |  |  |  |  |  |  |  |
| $132-11 | | 1 | 1 | 7.69% | 14831.1 | 6.8 | tr\|J3KSC4\|J3KSC4_HUMAN Ras-related C3 botulinum toxin substrate 3 (Fragment) OS=Homo sapiens GN=RAC3 PE=1 SV=1 |  |  |  |  |  |  |  |  |
| $132-12 | | 1 | 1 | 6.76% | 16775.5 | 8.3 | tr\|B1AH77\|B1AH77_HUMAN Ras-related C3 botulinum toxin substrate 2 OS=Homo sapiens GN=RAC2 PE=1 SV=1 |  |  |  |  |  |  |  |  |
|  | R16062_1_CTGF,9477 | K.YLECSALTQR.G | 1241.3694 | 0.6274 | 2 | 1 | 2.2514 | 0.3464 | 1253.3 | 1 | 14\|18 | 5.99 | 1 | 12 |  |
| $133-1 | | 1 | 1 | 2.15% | 63173.5 | 5.4 | sp\|P02748\|CO9_HUMAN Complement component C9 OS=Homo sapiens GN=C9 PE=1 SV=2 |  |  |  |  |  |  |  |  |
| $133-2 | | 1 | 1 | 2.15% | 63203.6 | 5.4 | tr\|A0A024R035\|A0A024R035_HUMAN Complement component 9, isoform CRA_a OS=Homo sapiens GN=C9 PE=4 SV=1 |  |  |  |  |  |  |  |  |
|  | R16062_1_CTGF,15767 | R.AIEDYINEFSVR.K | 1456.5819 | 0.5669 | 2 | 1 | 2.7894 | 0.484 | 938.9 | 1 | 14\|22 | 4.14 | 1 | 2 |  |
| $134-1 | | 1 | 1 | 1.47% | 84138.1 | 5.8 | sp\|Q96TA1\|NIBL1_HUMAN Niban-like protein 1 OS=Homo sapiens GN=FAM129B PE=1 SV=3 |  |  |  |  |  |  |  |  |
| $134-2 | | 1 | 1 | 1.50% | 82683.6 | 5.8 | tr\|A0A024R872\|A0A024R872_HUMAN Chromosome 9 open reading frame 88, isoform CRA_a OS=Homo sapiens GN=C9orf88 PE=4 SV=1 |  |  |  |  |  |  |  |  |
| $134-3 | | 1 | 1 | 2.78% | 43823.7 | 5.3 | tr\|Q9H8K1\|Q9H8K1_HUMAN cDNA FLJ13518 fis, clone PLACE1005799 OS=Homo sapiens PE=2 SV=1 |  |  |  |  |  |  |  |  |
| $134-4 | | 1 | 1 | 3.36% | 35228.6 | 6.2 | tr\|Q2YD88\|Q2YD88_HUMAN FAM129B protein (Fragment) OS=Homo sapiens GN=FAM129B PE=2 SV=1 |  |  |  |  |  |  |  |  |
|  | R16062_1_CTGF,17627 | R.FQELIFEDFAR.F | 1415.5746 | 1.2416 | 2 | 1 | 2.4922 | 0.2049 | 981.8 | 1 | 14\|20 | 4.14 | 1 | 4 |  |
| $135-1 | | 1 | 1 | 1.76% | 101389 | 6.3 | sp\|P19827\|ITIH1_HUMAN Inter-alpha-trypsin inhibitor heavy chain H1 OS=Homo sapiens GN=ITIH1 PE=1 SV=3 |  |  |  |  |  |  |  |  |
| $135-2 | | 1 | 1 | 2.48% | 72119.3 | 7.3 | tr\|B7Z539\|B7Z539_HUMAN cDNA FLJ56954, highly similar to Inter-alpha-trypsin inhibitor heavy chain H1 OS=Homo sapiens PE=2 SV=1 |  |  |  |  |  |  |  |  |
| $135-3 | | 1 | 1 | 2.36% | 75508 | 6.5 | tr\|B7Z549\|B7Z549_HUMAN cDNA FLJ56821, highly similar to Inter-alpha-trypsin inhibitor heavy chain H1 OS=Homo sapiens PE=2 SV=1 |  |  |  |  |  |  |  |  |
| $135-4 | | 1 | 1 | 2.57% | 69526.6 | 5.7 | tr\|B7Z8B6\|B7Z8B6_HUMAN cDNA FLJ54395, highly similar to Inter-alpha-trypsin inhibitor heavy chain H1 OS=Homo sapiens PE=2 SV=1 |  |  |  |  |  |  |  |  |
|  | R16062_1_CTGF,15792 | R.GFSLDEATNLNGGLLR.G | 1677.8402 | -0.8658 | 2 | 1 | 2.2657 | 0.1566 | 325.3 | 8 | 12\|30 | 4.37 | 1 | 4 |  |
| $136-1 | | 1 | 1 | 2.16% | 122205 | 5.4 | sp\|P00450\|CERU_HUMAN Ceruloplasmin OS=Homo sapiens GN=CP PE=1 SV=1 |  |  |  |  |  |  |  |  |
| $136-2 | | 1 | 1 | 2.16% | 122205 | 5.4 | tr\|A5PL27\|A5PL27_HUMAN CP protein OS=Homo sapiens GN=CP PE=2 SV=1 |  |  |  |  |  |  |  |  |
| $136-3 | | 1 | 1 | 2.29% | 115472 | 5.4 | tr\|Q1L857\|Q1L857_HUMAN Ceruloplasmin (Fragment) OS=Homo sapiens PE=4 SV=1 |  |  |  |  |  |  |  |  |
| $136-4 | | 1 | 1 | 2.16% | 122172 | 5.5 | tr\|A8K5A4\|A8K5A4_HUMAN cDNA FLJ76826, highly similar to Homo sapiens ceruloplasmin (ferroxidase) (CP), mRNA OS=Homo sapiens PE=2 SV=1 |  |  |  |  |  |  |  |  |
| $136-5 | | 1 | 1 | 2.43% | 108820 | 5.5 | tr\|E9PFZ2\|E9PFZ2_HUMAN Ceruloplasmin OS=Homo sapiens GN=CP PE=1 SV=1 |  |  |  |  |  |  |  |  |
| $136-6 | | 1 | 1 | 2.70% | 97713.4 | 5.3 | tr\|H7C5R1\|H7C5R1_HUMAN Ceruloplasmin (Fragment) OS=Homo sapiens GN=CP PE=1 SV=1 |  |  |  |  |  |  |  |  |
| $136-7 | | 1 | 1 | 2.43% | 108822 | 5.5 | tr\|B7Z5Q2\|B7Z5Q2_HUMAN cDNA FLJ58075, highly similar to Ceruloplasmin (EC 1.16.3.1) OS=Homo sapiens PE=2 SV=1 |  |  |  |  |  |  |  |  |
| $136-8 | | 1 | 1 | 2.96% | 89045.8 | 5.5 | tr\|B3KTA8\|B3KTA8_HUMAN cDNA FLJ37971 fis, clone CTONG2009958, highly similar to CERULOPLASMIN (EC 1.16.3.1) OS=Homo sapiens PE=2 SV=1 |  |  |  |  |  |  |  |  |
|  | R16062_1_CTGF,20033 | R.HYYIAAEEIIWNYAPSGIDIFTK.E | 2716.0387 | 0.6077 | 3 | 1 | 3.8885 | 0.3579 | 1306.1 | 1 | 32\|88 | 4.65 | 1 | 8 |  |
| $137-1 | | 1 | 1 | 0.55% | 269767 | 5.8 | sp\|Q9Y490\|TLN1_HUMAN Talin-1 OS=Homo sapiens GN=TLN1 PE=1 SV=3 |  |  |  |  |  |  |  |  |
|  | R16062_1_CTGF,14870 | R.ILAQATSDLVNAIK.A | 1457.6976 | 0.1056 | 2 | 1 | 2.5377 | 0.3946 | 580.6 | 1 | 14\|26 | 5.84 | 1 | 1 |  |
| $138-1 | | 1 | 1 | 4.88% | 22782.6 | 6 | sp\|P04792\|HSPB1_HUMAN Heat shock protein beta-1 OS=Homo sapiens GN=HSPB1 PE=1 SV=2 |  |  |  |  |  |  |  |  |
| $138-2 | | 1 | 1 | 5.38% | 20406.3 | 9.3 | tr\|F8WE04\|F8WE04_HUMAN Heat shock protein beta-1 OS=Homo sapiens GN=HSPB1 PE=1 SV=1 |  |  |  |  |  |  |  |  |
| $138-3 | | 1 | 1 | 4.88% | 22782.6 | 6 | tr\|V9HW43\|V9HW43_HUMAN Epididymis secretory protein Li 102 OS=Homo sapiens GN=HEL-S-102 PE=2 SV=1 |  |  |  |  |  |  |  |  |
|  | R16062_1_CTGF,14863 | R.LFDQAFGLPR.L | 1164.3381 | 0.0931 | 2 | 1 | 2.437 | 0.2577 | 880.5 | 1 | 14\|18 | 5.84 | 1 | 3 |  |
| $139-1 | | 1 | 1 | 2.11% | 72691.6 | 7.1 | sp\|P31040\|SDHA_HUMAN Succinate dehydrogenase [ubiquinone] flavoprotein subunit, mitochondrial OS=Homo sapiens GN=SDHA PE=1 SV=2 |  |  |  |  |  |  |  |  |
| $139-2 | | 1 | 1 | 2.11% | 72691.6 | 7.1 | tr\|A0A024QZ30\|A0A024QZ30_HUMAN Succinate dehydrogenase [ubiquinone] flavoprotein subunit, mitochondrial OS=Homo sapiens GN=SDHA PE=3 SV=1 |  |  |  |  |  |  |  |  |
| $139-3 | | 1 | 1 | 2.70% | 56662.5 | 7.2 | tr\|A0A087X1I3\|A0A087X1I3_HUMAN Succinate dehydrogenase [ubiquinone] flavoprotein subunit, mitochondrial OS=Homo sapiens GN=SDHA PE=1 SV=1 |  |  |  |  |  |  |  |  |
| $139-4 | | 1 | 1 | 2.10% | 72688 | 7.3 | tr\|B4DYN5\|B4DYN5_HUMAN Succinate dehydrogenase [ubiquinone] flavoprotein subunit, mitochondrial OS=Homo sapiens PE=2 SV=1 |  |  |  |  |  |  |  |  |
| $139-5 | | 1 | 1 | 2.40% | 63567.1 | 6.8 | tr\|D6RFM5\|D6RFM5_HUMAN Succinate dehydrogenase [ubiquinone] flavoprotein subunit, mitochondrial OS=Homo sapiens GN=SDHA PE=1 SV=1 |  |  |  |  |  |  |  |  |
| $139-6 | | 1 | 1 | 2.55% | 60051.2 | 6.4 | tr\|Q0QF12\|Q0QF12_HUMAN Succinate dehydrogenase (quinone) (Fragment) OS=Homo sapiens GN=SDHA PE=2 SV=1 |  |  |  |  |  |  |  |  |
| $139-7 | | 1 | 1 | 3.19% | 48158 | 6.7 | tr\|B3KT34\|B3KT34_HUMAN cDNA FLJ37560 fis, clone BRCOC2000333, highly similar to Succinate dehydrogenase (ubiquinone) flavoprotein subunit, mitochondrial (EC 1.3.5.1) OS=Homo sapiens PE=2 SV=1 |  |  |  |  |  |  |  |  |
| $139-8 | | 1 | 1 | 11.11% | 13525.6 | 10 | tr\|H0Y8X1\|H0Y8X1_HUMAN Succinate dehydrogenase [ubiquinone] flavoprotein subunit, mitochondrial (Fragment) OS=Homo sapiens GN=SDHA PE=1 SV=1 |  |  |  |  |  |  |  |  |
|  | R16062_1_CTGF,20610 | R.LGANSLLDLVVFGR.A | 1474.7299 | 1.0179 | 2 | 1 | 2.3434 | 0.4897 | 493.1 | 1 | 12\|26 | 5.84 | 1 | 8 |  |
| $140-1 | | 1 | 1 | 5.13% | 43062.2 | 5.2 | sp\|Q12905\|ILF2_HUMAN Interleukin enhancer-binding factor 2 OS=Homo sapiens GN=ILF2 PE=1 SV=2 |  |  |  |  |  |  |  |  |
| $140-2 | | 1 | 1 | 5.68% | 38910.5 | 4.9 | tr\|B4DY09\|B4DY09_HUMAN Interleukin enhancer-binding factor 2 OS=Homo sapiens GN=ILF2 PE=1 SV=1 |  |  |  |  |  |  |  |  |
| $140-3 | | 1 | 1 | 5.13% | 43062.2 | 5.2 | tr\|F4ZW62\|F4ZW62_HUMAN NF45 OS=Homo sapiens PE=1 SV=1 |  |  |  |  |  |  |  |  |
| $140-4 | | 1 | 1 | 5.13% | 43004.2 | 5.3 | tr\|F4ZW63\|F4ZW63_HUMAN NF45 OS=Homo sapiens PE=2 SV=1 |  |  |  |  |  |  |  |  |
| $140-5 | | 1 | 1 | 13.33% | 16366.8 | 7.7 | tr\|A0A0A0MRL0\|A0A0A0MRL0_HUMAN Interleukin enhancer-binding factor 2 OS=Homo sapiens GN=ILF2 PE=1 SV=1 |  |  |  |  |  |  |  |  |
| $140-6 | | 1 | 1 | 17.39% | 12382.2 | 5.2 | tr\|X6R6Z1\|X6R6Z1_HUMAN Interleukin enhancer-binding factor 2 (Fragment) OS=Homo sapiens GN=ILF2 PE=1 SV=1 |  |  |  |  |  |  |  |  |
| $140-7 | | 1 | 1 | 5.13% | 43048.2 | 5.2 | tr\|Q53FG3\|Q53FG3_HUMAN Interleukin enhancer binding factor 2 variant (Fragment) OS=Homo sapiens PE=2 SV=1 |  |  |  |  |  |  |  |  |
|  | R16062_1_CTGF,16477 | R.NQDLAPNSAEQASILSLVTK.I | 2100.316 | 0.849 | 2 | 1 | 2.2825 | 0.3966 | 478.1 | 1 | 17\|38 | 4.37 | 1 | 7 |  |
| $141-1 | | 1 | 1 | 0.67% | 262624 | 5.5 | sp\|P02751\|FINC_HUMAN Fibronectin OS=Homo sapiens GN=FN1 PE=1 SV=4 |  |  |  |  |  |  |  |  |
| $141-2 | | 1 | 1 | 0.68% | 259211 | 5.5 | tr\|A0A024R462\|A0A024R462_HUMAN Fibronectin 1, isoform CRA_n OS=Homo sapiens GN=FN1 PE=4 SV=1 |  |  |  |  |  |  |  |  |
| $141-3 | | 1 | 1 | 2.61% | 66807 | 5.7 | tr\|A6YID2\|A6YID2_HUMAN Fibronectin splice variant A (Fragment) OS=Homo sapiens GN=FN1 PE=2 SV=1 |  |  |  |  |  |  |  |  |
| $141-4 | | 1 | 1 | 4.52% | 38655.9 | 9.7 | tr\|Q9UQS6\|Q9UQS6_HUMAN Fibronectin (Fragment) OS=Homo sapiens PE=2 SV=1 |  |  |  |  |  |  |  |  |
| $141-5 | | 1 | 1 | 3.06% | 56993.2 | 8.7 | tr\|A6YID4\|A6YID4_HUMAN Fibronectin splice variant C (Fragment) OS=Homo sapiens GN=FN1 PE=2 SV=1 |  |  |  |  |  |  |  |  |
| $141-6 | | 1 | 1 | 3.05% | 57031 | 5.4 | tr\|A6YID5\|A6YID5_HUMAN Fibronectin splice variant D (Fragment) OS=Homo sapiens GN=FN1 PE=2 SV=1 |  |  |  |  |  |  |  |  |
| $141-7 | | 1 | 1 | 1.55% | 113372 | 5.7 | tr\|Q6N084\|Q6N084_HUMAN Putative uncharacterized protein DKFZp686L11144 (Fragment) OS=Homo sapiens GN=DKFZp686L11144 PE=2 SV=1 |  |  |  |  |  |  |  |  |
| $141-8 | | 1 | 1 | 2.03% | 85622.8 | 5.3 | tr\|Q5CZ99\|Q5CZ99_HUMAN Putative uncharacterized protein DKFZp686I1370 (Fragment) OS=Homo sapiens GN=DKFZp686I1370 PE=2 SV=1 |  |  |  |  |  |  |  |  |
| $141-9 | | 1 | 1 | 2.48% | 70215.7 | 5.6 | tr\|A6YID6\|A6YID6_HUMAN Fibronectin splice variant E (Fragment) OS=Homo sapiens GN=FN1 PE=2 SV=1 |  |  |  |  |  |  |  |  |
| $141-10 | | 1 | 1 | 1.58% | 110884 | 5.8 | tr\|Q59G22\|Q59G22_HUMAN Fibronectin 1 variant (Fragment) OS=Homo sapiens PE=2 SV=1 |  |  |  |  |  |  |  |  |
| $141-11 | | 1 | 1 | 1.79% | 98155.7 | 5.9 | tr\|B4DU16\|B4DU16_HUMAN cDNA FLJ54550, highly similar to Homo sapiens fibronectin 1 (FN1), transcript variant 6, mRNA OS=Homo sapiens PE=2 SV=1 |  |  |  |  |  |  |  |  |
| $141-12 | | 1 | 1 | 1.45% | 120774 | 5.5 | tr\|H0Y7Z1\|H0Y7Z1_HUMAN Fibronectin (Fragment) OS=Homo sapiens GN=FN1 PE=1 SV=1 |  |  |  |  |  |  |  |  |
| $141-13 | | 1 | 1 | 1.58% | 111303 | 5.8 | tr\|B4DTK1\|B4DTK1_HUMAN cDNA FLJ53292, highly similar to Homo sapiens fibronectin 1 (FN1), transcript variant 5, mRNA OS=Homo sapiens PE=2 SV=1 |  |  |  |  |  |  |  |  |
| $141-14 | | 1 | 1 | 0.71% | 246698 | 5.7 | tr\|B7ZLE5\|B7ZLE5_HUMAN FN1 protein OS=Homo sapiens GN=FN1 PE=2 SV=1 |  |  |  |  |  |  |  |  |
| $141-15 | | 1 | 1 | 2.72% | 64092.9 | 6.1 | tr\|A6YID3\|A6YID3_HUMAN Fibronectin splice variant B (Fragment) OS=Homo sapiens GN=FN1 PE=2 SV=1 |  |  |  |  |  |  |  |  |
| $141-16 | | 1 | 1 | 4.22% | 41369.9 | 9.3 | tr\|O95617\|O95617_HUMAN Fibronectin (Fragment) OS=Homo sapiens PE=2 SV=1 |  |  |  |  |  |  |  |  |
| $141-17 | | 1 | 1 | 0.75% | 235427 | 5.5 | tr\|Q68CX6\|Q68CX6_HUMAN Putative uncharacterized protein DKFZp686O13149 OS=Homo sapiens GN=DKFZp686O13149 PE=2 SV=1 |  |  |  |  |  |  |  |  |
|  | R16062_1_CTGF,12807 | R.PAQGVVTTLENVSPPR.R | 1665.8726 | -0.1344 | 2 | 1 | 2.4481 | 0.4239 | 736.1 | 1 | 14\|30 | 6.43 | 1 | 17 |  |
| $142-1 | | 1 | 1 | 2.75% | 50642.9 | 8.7 | sp\|O60813\|PRA11_HUMAN PRAME family member 11 OS=Homo sapiens GN=PRAMEF11 PE=2 SV=3 |  |  |  |  |  |  |  |  |
| $142-2 | | 1 | 1 | 2.51% | 55353.6 | 9 | sp\|A6NGN4\|PRA25_HUMAN PRAME family member 25 OS=Homo sapiens GN=PRAMEF25 PE=3 SV=2 |  |  |  |  |  |  |  |  |
| $142-3 | | 1 | 1 | 3.14% | 44302.3 | 8.7 | sp\|H0Y7S4\|PRA26_HUMAN Putative PRAME family member 26 OS=Homo sapiens GN=PRAMEF26 PE=5 SV=2 |  |  |  |  |  |  |  |  |
| $142-4 | | 1 | 1 | 2.51% | 55466.8 | 8.7 | sp\|O60810\|PRAM4_HUMAN PRAME family member 4 OS=Homo sapiens GN=PRAMEF4 PE=2 SV=4 |  |  |  |  |  |  |  |  |
| $142-5 | | 1 | 1 | 2.51% | 55419.7 | 8.8 | sp\|Q5VWM5\|PRAM9_HUMAN PRAME family member 9/15 OS=Homo sapiens GN=PRAMEF9 PE=2 SV=1 |  |  |  |  |  |  |  |  |
| $142-6 | | 1 | 1 | 2.52% | 55160.4 | 8.8 | tr\|A0A087X198\|A0A087X198_HUMAN PRAME family member 9/15 OS=Homo sapiens GN=PRAMEF15 PE=4 SV=1 |  |  |  |  |  |  |  |  |
| $142-7 | | 1 | 1 | 2.51% | 55343.7 | 8.8 | tr\|A0A087WW85\|A0A087WW85_HUMAN PRAME family member 11 OS=Homo sapiens GN=PRAMEF11 PE=4 SV=2 |  |  |  |  |  |  |  |  |
| $142-8 | | 1 | 1 | 2.51% | 55352.6 | 8.9 | tr\|A0A087X1J8\|A0A087X1J8_HUMAN PRAME family member 25 OS=Homo sapiens GN=PRAMEF26 PE=4 SV=1 |  |  |  |  |  |  |  |  |
| $142-9 | | 1 | 1 | 2.52% | 55094.3 | 8.9 | tr\|A0A087WUW3\|A0A087WUW3_HUMAN Putative PRAME family member 26 OS=Homo sapiens GN=PRAMEF26 PE=4 SV=1 |  |  |  |  |  |  |  |  |
| $142-10 | | 1 | 1 | 2.51% | 55438.8 | 8.7 | tr\|A0A0J9YWX7\|A0A0J9YWX7_HUMAN PRAME family member 4 OS=Homo sapiens GN=PRAMEF4 PE=4 SV=1 |  |  |  |  |  |  |  |  |
| $142-11 | | 1 | 1 | 2.51% | 55340.6 | 8.7 | tr\|B7ZMC2\|B7ZMC2_HUMAN PRAMEF11 protein OS=Homo sapiens GN=PRAMEF11 PE=2 SV=1 |  |  |  |  |  |  |  |  |
| $142-12 | | 1 | 1 | 2.51% | 55399.7 | 8.8 | tr\|B7ZW04\|B7ZW04_HUMAN PRAME family member 9 OS=Homo sapiens GN=PRAMEF9 PE=2 SV=1 |  |  |  |  |  |  |  |  |
| $142-13 | | 1 | 1 | 2.75% | 50601.9 | 9 | tr\|A0A0G2JN56\|A0A0G2JN56_HUMAN Putative PRAME family member 26 OS=Homo sapiens GN=PRAMEF26 PE=4 SV=1 |  |  |  |  |  |  |  |  |
|  | R16062_1_CTGF,15462 | R.QPLTVFVELWLK.N | 1473.7835 | -1.3145 | 2 | 1 | 2.2354 | 0.1596 | 231.2 | 19 | 9\|22 | 6 | 1 | 13 |  |
| $143-1 | | 1 | 1 | 1.40% | 95338.2 | 6.4 | sp\|P13639\|EF2_HUMAN Elongation factor 2 OS=Homo sapiens GN=EEF2 PE=1 SV=4 |  |  |  |  |  |  |  |  |
| $143-2 | | 1 | 1 | 2.38% | 55669.5 | 6.3 | tr\|B4DRE8\|B4DRE8_HUMAN cDNA FLJ58164, highly similar to Elongation factor 2 OS=Homo sapiens PE=2 SV=1 |  |  |  |  |  |  |  |  |
| $143-3 | | 1 | 1 | 2.06% | 64780.7 | 8.8 | tr\|Q6PK56\|Q6PK56_HUMAN EEF2 protein (Fragment) OS=Homo sapiens GN=EEF2 PE=2 SV=1 |  |  |  |  |  |  |  |  |
| $143-4 | | 1 | 1 | 3.10% | 42934.7 | 5.9 | tr\|B4DMC6\|B4DMC6_HUMAN cDNA FLJ60696, highly similar to Elongation factor 2 OS=Homo sapiens PE=2 SV=1 |  |  |  |  |  |  |  |  |
|  | R16062_1_CTGF,19271 | R.TFCQLILDPIFK.V | 1495.7809 | 0.5169 | 2 | 1 | 2.5668 | 0.2803 | 538.8 | 1 | 13\|22 | 5.5 | 1 | 4 |  |
| $144-1 | | 1 | 1 | 2.56% | 62261.3 | 7.2 | sp\|P36269\|GGT5_HUMAN Gamma-glutamyltransferase 5 OS=Homo sapiens GN=GGT5 PE=1 SV=2 |  |  |  |  |  |  |  |  |
| $144-2 | | 1 | 1 | 2.99% | 53799.4 | 6.9 | tr\|B4DND4\|B4DND4_HUMAN cDNA FLJ50588, highly similar to Gamma-glutamyltransferase 5 (EC 2.3.2.2) OS=Homo sapiens PE=2 SV=1 |  |  |  |  |  |  |  |  |
| $144-3 | | 1 | 1 | 2.94% | 53813.5 | 7.2 | tr\|B4DEL3\|B4DEL3_HUMAN cDNA FLJ50454, moderately similar to Gamma-glutamyltransferase 5 (EC2.3.2.2) OS=Homo sapiens PE=2 SV=1 |  |  |  |  |  |  |  |  |
| $144-4 | | 1 | 1 | 6.82% | 23144.6 | 8.9 | tr\|H7C1X2\|H7C1X2_HUMAN Gamma-glutamyltransferase 5 (Fragment) OS=Homo sapiens GN=GGT5 PE=1 SV=1 |  |  |  |  |  |  |  |  |
|  | R16062_1_CTGF,16703 | R.TGIILNNELLDLCER.C | 1774.0028 | 1.9848 | 2 | 1 | 2.9251 | 0.4743 | 895.8 | 1 | 15\|28 | 4.14 | 1 | 4 |  |
| $145-1 | | 1 | 1 | 5.91% | 57116.5 | 4.8 | sp\|P07237\|PDIA1_HUMAN Protein disulfide-isomerase OS=Homo sapiens GN=P4HB PE=1 SV=3 |  |  |  |  |  |  |  |  |
| $145-2 | | 1 | 1 | 5.91% | 57116.5 | 4.8 | tr\|A0A024R8S5\|A0A024R8S5_HUMAN Protein disulfide-isomerase OS=Homo sapiens PE=3 SV=1 |  |  |  |  |  |  |  |  |
| $145-3 | | 1 | 1 | 12.40% | 26680.3 | 5.1 | tr\|B4DJS0\|B4DJS0_HUMAN cDNA FLJ56766, highly similar to Protein disulfide-isomerase (EC5.3.4.1) OS=Homo sapiens PE=2 SV=1 |  |  |  |  |  |  |  |  |
| $145-4 | | 1 | 1 | 6.10% | 55381.4 | 4.7 | tr\|B4DNL5\|B4DNL5_HUMAN Protein disulfide-isomerase OS=Homo sapiens PE=2 SV=1 |  |  |  |  |  |  |  |  |
| $145-5 | | 1 | 1 | 19.23% | 16876 | 4.6 | tr\|I3L3P5\|I3L3P5_HUMAN Protein disulfide-isomerase (Fragment) OS=Homo sapiens GN=P4HB PE=1 SV=1 |  |  |  |  |  |  |  |  |
| $145-6 | | 1 | 1 | 14.42% | 23022.2 | 4.9 | tr\|I3L312\|I3L312_HUMAN Protein disulfide-isomerase (Fragment) OS=Homo sapiens GN=P4HB PE=1 SV=2 |  |  |  |  |  |  |  |  |
|  | R16062_1_CTGF,22054 | R.TGPAATTLPDGAAAESLVESSEVAVIGFFK.D | 2937.2473 | 1.4743 | 3 | 1 | 3.7862 | 0.4743 | 558.7 | 1 | 25\|116 | 4 | 1 | 6 |  |
| $146-1 | | 1 | 1 | 2.80% | 52602.5 | 6.3 | sp\|P01008\|ANT3_HUMAN Antithrombin-III OS=Homo sapiens GN=SERPINC1 PE=1 SV=1 |  |  |  |  |  |  |  |  |
| $146-2 | | 1 | 1 | 14.29% | 10493.3 | 4.6 | tr\|Q9UE54\|Q9UE54_HUMAN Gene for antithrombin-III (aa 262-353) OS=Homo sapiens PE=4 SV=1 |  |  |  |  |  |  |  |  |
| $146-3 | | 1 | 1 | 2.80% | 52542.4 | 6.3 | tr\|A0A0K0Q2Z1\|A0A0K0Q2Z1_HUMAN Serpin peptidase inhibitor clade C member 1 OS=Homo sapiens GN=SERPINC1 PE=2 SV=1 |  |  |  |  |  |  |  |  |
| $146-4 | | 1 | 1 | 2.80% | 52602.5 | 6.3 | tr\|A0A024R944\|A0A024R944_HUMAN Serpin peptidase inhibitor, clade C (Antithrombin), member 1, isoform CRA_a OS=Homo sapiens GN=SERPINC1 PE=3 SV=1 |  |  |  |  |  |  |  |  |
|  | R16062_1_CTGF,15354 | R.VAEGTQVLELPFK.G | 1431.6586 | 0.6316 | 2 | 1 | 2.3822 | 0.403 | 755.5 | 1 | 12\|24 | 4.53 | 1 | 4 |  |
|  |  |  |  |  |  |  |  |  |  |  |  |  |  |  |  |
